# Catalytic Performance of Immobilized Sulfuric Acid on Silica Gel for *N*-Formylation of Amines with Triethyl Orthoformate

**DOI:** 10.3390/molecules27134213

**Published:** 2022-06-30

**Authors:** Sodeeq Aderotimi Salami, Xavier Siwe-Noundou, Rui W. M. Krause

**Affiliations:** 1 Department of Chemistry, Rhodes University, Grahamstown 6140, South Africa; 2 Department of Pharmaceutical Sciences, School of Pharmacy, Sefako Makgatho Health Sciences University, P.O Box 218, Pretoria 0204, South Africa

**Keywords:** *N*-formylation, amines, immobilized sulfuric acid, silica gel, triethyl orthoformate

## Abstract

In the search for convenient, green, and practical catalytic methods for the current interest in organic synthesis, a simple, green, and highly efficient protocol for *N*-formylation of various amines was carried out in the presence of immobilized sulfuric acid on silica gel (H_2_SO_4_–SiO_2_). All reactions were performed in refluxing triethyl orthoformate (65 °C). The product formamides were obtained with high-to-excellent yields within 4 min to 2 h. The current approach is advantageous, due to its short reaction time and high yields. The catalyst is recyclable with no significant loss in catalytic efficiency.

## 1. Introduction

**Figure 1:**
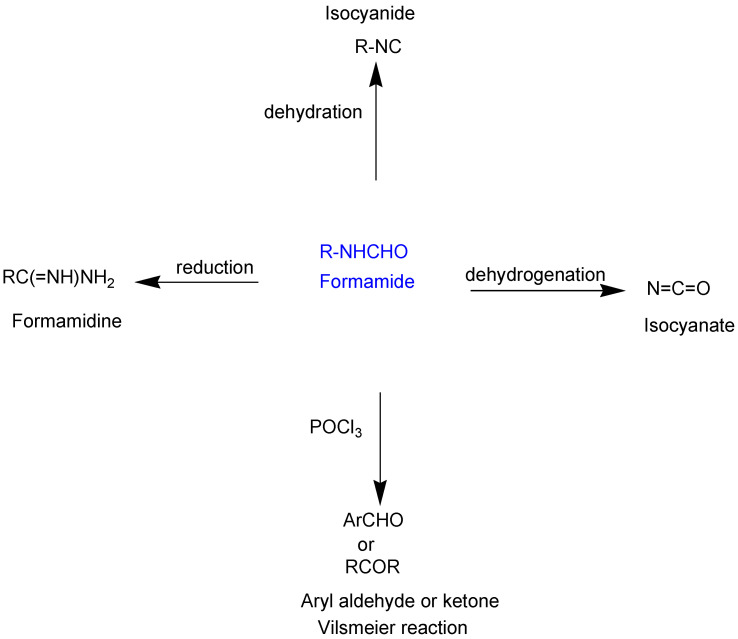
Schematic representation depicting *N*-formamide as versatile synthetic reagent.

A fascinating trend in the synthesis of widely used organic molecules is the focus on green chemistry, including efficient reactions and the use of ecologically friendly reagents [1]. The use of silica gel as an effective catalyst in chemical processes has attracted much attention in recent years. The formylation of amines is a crucial process in organic chemistry, owing to the widespread application of *N*-formyl amine derivatives in industry and in biologically active compounds, such as fluoroquinolones, substituted imidazoles, 1,2-dihydroquinolines, and nitrogen-bridged heterocycles, among others [2]. *N*-formyl amine derivatives have also been used as reagents in Vilsmeier formylation reactions as amino acid-protecting groups [3] and in the synthesis of several other important derivatives, such as formamidines [4], isocyanates [5], and nitriles [6] (Figure 1).

Despite the fact that there are a variety of reagents for *N*-formylation of amines, the synthesis of formamides utilizing triethyl orthoformate as a formylating agent is still popular [1]. The reaction of ethyl orthoformate with aniline to afford *N,N*′-diphenylformamidine was initially reported in 1869 by Wichelhaus [7]. Subsequently, Claisen synthesized ethyl *N*-phenylformimidate in low yields from the same reactants, but under slightly different experimental conditions [8]. Swaringen and colleagues went on to show that the reaction of *N*-alkylanilines with orthoformates in the absence of a catalyst or with hydrochloric/acetic acid produced orthoamides in low yields [8]. These few examples demonstrate one of the major drawbacks of this system, namely, the low yield. Meanwhile, when *p*-toluenesulfonic acid was employed as a catalyst, high yields of *N*-alkylformanilides and *N,N*-dialkylanilines were generated, but the reactions still often required high temperatures and prolonged reaction times. For example, Swaringen and co-workers demonstrated the synthesis of *N*-ethyl formamides from the reaction of amines with triethyl orthoformate in the presence of H_2_SO_4_, but under severe conditions (temperature above 140 °C) [9].

Various other formylating agents have been reported, including chloral [10], acetic formic anhydride [11], formic acid [12], ammonium formate [13], formate esters [14], polymer-supported formate [15], ethyl formate [16], triethyl orthoformate [1,2], aldehydes and methanol [17], carbon monoxide [18], and carbon dioxide [19]. However, these also tend to suffer from similar problems of long reaction times (hours to days), variable or low yields, and harsh conditions (or expensive catalyst systems).

Several catalysts have been employed for the formylation of amines, including silica-supported sulfuric acid [20], H_2_SO_4_/NaHSO_4_-activated charcoal [21], K-F alumina [22], Amberlite IR 120 [23], ZnO [24], nano-CeO_2_ [25], nano-MgO [26], natrolite zeolite [27], indium metal [28], sulfated titania [29], and sulfated tungstate [30], among others (Table 1).

In the absence of a catalyst or promoter, *N*-formylation of amines is a sluggish reaction that usually requires unique reaction conditions or long time frames for completion [25]. However, some of these methods have quite a number of limitations, including harsh reaction conditions, the need for expensive metal catalysts or organocatalysts, and long reaction time frames. Thus, for organic transformations, the development of a safe, benign, environmentally friendly, high-yield, quick-reaction, and recyclable catalyst for *N*-formylation of amines remains extremely desirable [3].

**Figure 2:**
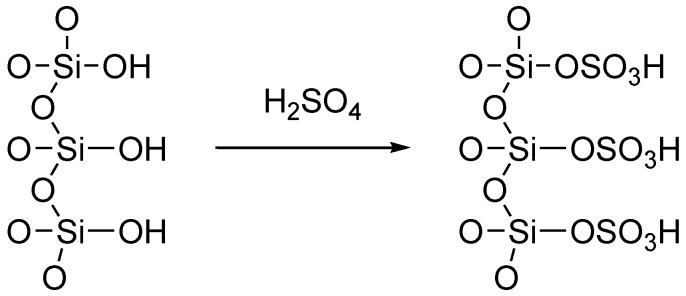
Immobilized sulfuric acid on silica gel.

In the last few years, H_2_SO_4_–SiO_2_ (Table 2) has demonstrated significant promise as a cost-effective and easily retrievable solid catalyst for driving a variety of essential organic reactions in solvent-free environments. H_2_SO_4_–SiO_2_ is appealing for industrial usage because of its high catalytic activity, operational simplicity, and recyclability. There are two types of functional groups on the silica surface: siloxane (Si–O–Si) and silanol (Si–OH). Thus, silica gel modification can occur through the reaction of a specific molecule with either the siloxane (nucleophilic substitution at the Si) or silanol (direct reaction with the hydroxyl group) functions, though it is widely accepted that the reaction with the silanol function is the most common modification pathway (Figure 2) [47,48]. The notion of employing H_2_SO_4_–SiO_2_ as a transamidation catalyst was inspired by Rasheed et al. [20]. We became interested in employing the same catalyst to build a generic formylation with triethyl orthoformate. To the best of our knowledge, no reports of H_2_SO_4_–SiO_2_-catalyzed formylation with triethyl orthoformate have been published, and so for the first time, we present findings in this regard.

## 2. Results and Discussion

**Figure 3:**
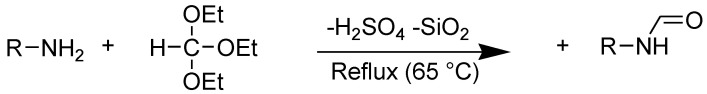
*N*-formylation of amines with triethyl orthoformate.

Initially, the reaction of aniline with triethyl orthoformate was chosen as the model reaction (Figure 3). During the optimization of reaction parameters, it was observed that aniline reacted smoothly with triethyl orthoformate, providing the desired product with a good yield (96%) within a short period of time (Table 3).

In order to generalize the protocol for the formylation of sterically hindered amines, the reaction was optimized with respect to temperature and molar ratio. The temperature was raised to 65 °C and was observed to be quite sufficient to carry out the reaction with an optimum yield of the desired product (Table 3). It was observed that the need for an excess of triethyl orthoformate was no longer required, as a 1:3 molar ratio of amine to triethyl orthoformate was sufficient to yield the desired product (Table 3, entry 3).

We next explored the impact of immobilized sulfuric acid on silica gel stoichiometry on the outcome of the reaction (Table 4). We observed that excess H_2_SO_4_–SiO_2_ was not beneficial for faster conversion. Conversely, a lower amount of H_2_SO_4_–SiO_2_ led to substantially slower conversion. The background reaction (used as a model) was also measured in the absence of H_2_SO_4_–SiO_2_, confirming its vital role.

In general, the reaction proceeded efficiently, with various amines reacting with triethyl orthoformate to produce the corresponding *N*-formylated product with good-to-excellent yield within a very short time. Aliphatic and aromatic primary amines underwent smooth *N*-formylation and gave the product in 70–96% yields (Table 5).

Aniline with electron-donating groups provided an excellent yield of 65–96% with triethyl orthoformate. The halogen (F, Cl, Br, I)-containing anilines provided good yields, ranging from 73% to 96%, of corresponding products. Similarly, electron-withdrawing groups were found to react smoothly under the optimized reaction conditions and demonstrate good yields of desired products (85–96%). Generally, under these optimized reaction conditions, various functional groups were tolerated. However, finding a general method for generating amide bonds will surely benefit the drug discovery process. In general, the formylation of aryl/heteroaryl amines (electron-neutral, -rich, -deficient), aliphatic, and cyclic secondary amines afforded the formylation products in excellent yields (70–96%). Interestingly, sterically hindered aryl amines, such as products **6**, **7**, **10**, **11**, **16**, **17**, and **33**–**38**, were found to react smoothly under the optimized reaction conditions, demonstrating good yields of desired products. Less reactive hetero aromatics, such as **42**–**51** and **56**, produced the product with a surprisingly high yield (77–90%) and a longer reaction time (35–60 min). When secondary amines **52**–**54** were employed, the reaction was somehow slow, providing a good yield of products in 1 h (Table 5). NMR spectral data of all synthesized compounds are available in the Supplementary Materials (S1–S56).

## 3. Reusability of Catalyst

The reusability of the catalytic system was explored. The catalyst was separated by simple filtration and washed with ethyl acetate after the reaction was completed, and it was reused for two consecutive cycles within the same time frame (4 min), with a slight decrease in catalytic activity (9–13%) (Table 6).

In order to demonstrate the efficiency and versatility of the H_2_SO_4_–SiO_2_ system, we compared the result of *N*-formylation of aniline with other protocols that have been published based on reaction times and yields (Table 7). The results showed that the other approaches required longer reaction times for efficient conversion than for the present protocol. Therefore, on this basis, the present protocol is more efficient or comparable with other methodologies.

**Figure 4:**
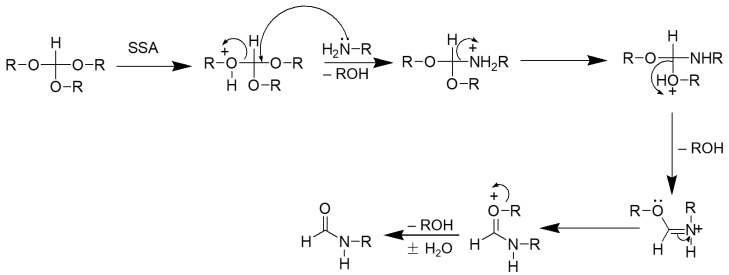
Proposed mechanism for *N*-formylation of amines with triethyl orthoformate.

Even though we have yet to prove the mechanism of our reaction in an experimental manner, Figure 4 suggests a possible explanation. The first step is the activation of the electrophilic carbon of triethyl orthoformate by the sulfonic group of H_2_SO_4_–SiO_2_, which led to the formation of a cationic intermediate. The cationic intermediate reacted with amine nucleophiles, which, on further elimination of ethanol, furnished the desired formylated product.

While 1,8-difformamido-naphthalein (**38**) and 3-formamido-1,2,4-triazole-5-thiol (**53**) are new derivatives and were characterized by one- and two-dimensional NMR analysis and high-resolution mass spectroscopy, all other products are known compounds and were identified by melting point, IR, ^1^H NMR, and ^13^C NMR spectroscopy. The synthesis of formamides was confirmed by IR spectra, which revealed two distinct absorption bands between 3300 and 3400 cm^−1^ (secondary NH) and 1640 and 1680 cm^−1^ (*N*-formyl, C=O).

Furthermore, formamide molecules have both a conformational stereogenic axis and a configurational stereogenic centre. These molecules take on two distinct *syn* and *anti*-conformational diastereomers as a result of restricted rotation around the Ar–N bond [50]. The ^1^H and ^13^C NMR spectra of most of the synthesized formamides at 25 °C were consistent with the presence of two rotamers. Only one rotamer was observed for the compounds **8**, **14**, **27**, **45** and **46**.

**Figure 5:**
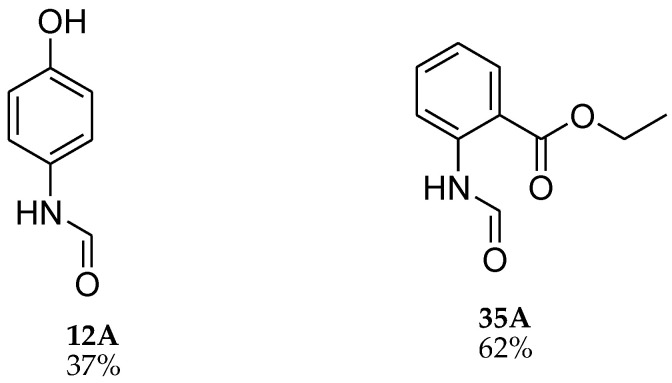
Yield of isolated conformers **12A** and **35A**.

During the purification of compounds **12** and **35**, two products appeared as partially separated spots on thin-layer chromatography (TLC) plates. Using normal silica gel chromatography, these compounds were identified as A and B rotamer pairs. After purifying compounds **12** and **35**, pure rotamers **12A** and **35A** were isolated (Figure 5). **12A** and **35A** were the only pure isomers that could be isolated, while **12B** and **35B** were always contaminated to some degree by **12A** and **35A**, respectively. The fact that we were able to isolate rotamers A and B at room temperature and characterize them using basic spectroscopic techniques astounded us. This occurrence may be viewed as a specific form of atropisomerism, because atropisomers are stereoisomers with restricted rotation around a single bond where the rotational barrier is high enough to allow isolation of the isomeric species [51].

## 4. Materials and Methods

A PerkinElmer Spectrum 100 FT-IR Spectrometer (Valencia, CA, USA) was used for the FT-IR analysis. The IR spectra were obtained by the attenuated total reflection (ATR) method. For each experiment, 16 scans were performed in the frequency range from 650 to 4000 cm^−1^. Melting points of all the compounds were determined using a Koffler hot-stage apparatus and were uncorrected. NMR spectra were recorded on a Bruker Advance III 400 spectrometer (Rheinstetten, Germany) using CDCl_3_ or DMSO-d_6_ as a solvent with tetramethyl silane used as internal standard. LC-MS/MS data were recorded on a Bruker Compact quadrupole time of flight (QToF) mass spectrometer (Bremen, Germany). Raw mass spectrometry data were processed using MZmine software (version 2.38) (San Diego, CA, USA). Solvents and chemicals used were of analytical grade, purchased from Sigma Aldrich (St. Louis, MO, USA) and used without further purification. The purity determination of the starting materials and reaction monitoring were performed by thin-layer chromatography (TLC) on Merck silica gel G F254plates (Duren, Germany).

### 4.1. Preparation of Sulfuric Acid Adsorbed on Silica Gel (H_2_SO_4_–SiO_2_)

The preparation of H_2_SO_4_–SiO_2_ was carried out by following the reported procedure [52]. To a suspension of silica gel (29.5 g, 230–400 mesh size) in EtOAc (60 mL), H_2_SO_4_ (1.5 g, 15.5 mmol, 0.8 mL of a 98% aq. solution of H_2_SO_4_) was added and the mixture was stirred magnetically for 30 min at room temperature. EtOAc was removed under reduced pressure (rotary evaporator) and the residue was heated at 100 °C for 72 h under vacuum to afford H_2_SO_4_–SiO_2_ as a free-flowing powder.

### 4.2. A General Procedure for N-Formylation of Amines with Triethyl Orthoformate Promoted by Immobilized H_2_SO_4_ on Silica Gel

To a mixture of aniline (0.548 mL, 6 mmol) and triethyl orthoformate (24 mmol), the immobilized H_2_SO_4_ on silica gel (1.2 g) was then added and the reaction mixture was stirred under reflux conditions (65 °C). Progress of the reaction was monitored by TLC. After completion of the reaction, the mixture was diluted with EtOAc (20 mL), filtered, water (30 mL) was added, the solution was extracted with EtOAc, and the combined organic layers were dried over anhydrous Na_2_SO_4_ and concentrated. The residue was subjected to column chromatography and eluted with (EtOAc–Pet Ether (3:1)) to afford the product in high yields.

## 5. Conclusions

We have developed a simple, green, and highly efficient protocol for *N*-formylation of various amines in the presence of immobilized sulfuric acid on silica gel, with excellent yields and remarkably simple and environmentally benign processes. The approach is compatible with a wide range of aromatic, heteroaromatic, aliphatic, and cyclic/acyclic primary or secondary amines. The H_2_SO_4_–SiO_2_ catalytic system described here is a good complement to previously reported protocols, due to its ease of manipulation, low cost, and benign nature. We are optimistic that, with this approach, we will be able to develop the biologically relevant heterocyclic ring system more efficiently. This protocol is generic, and it will undoubtedly offer value to the growing area of organic synthesis.

## Figures and Tables

**Table 1:** Catalysts in combination with formylating agents employed for the formylation of various amines.

Entry	Catalyst	Formylating Agent	Reaction Condition	Time	Yield %	Reference
1	Sodium formate	Formic acid	Solvent free	>8 h		[31]
2	Amberlite IR-120	Formic acid	Microwave irradiation	2 min	90–97	[23]
3	Molecular iodine (I_2_)	Formic acid	Solvent free	2 h	60–99	[32]
4	Thiamine hydrochloride	Formic acid	Solvent free		88–96	[33]
5	Fe_2_O_3_-Hap-SO_3_H	Formic acid	Solvent free	15–60 min	95–99	[34]
6	Sulfated tungstate	Formic acid	Solvent free	10–45 min	85–95	[35]
7	CDMT II	Formic acid	Microwave irradiation	3–6 min	64–94	[36]
8	Amidine and Guanidine	Methyl formate	Solvent free	1–96 h	65–98	[37]
9	TBD-based ionic liquids	Formic acid	Solvent free	10–35 min	75–98	[38]
10	Indium	Formic acid	Solvent free	1.5–24 h	70–98	[28]
11	ZnO	Formic acid	Solvent free	10–720 min	65–99	[24]
12	ZnCl_2_	Formic acid	Solvent free	10–900 min	60–98	[39]
13	TiO_2_-P25 or TiO_2_-SO_4_^2−^	Formic acid	Solvent free	30–45 min	40–99	[29]
14	FSG-HF(N(SO_2_C_8_F_11_)_2_)_4_	Formic acid	Solvent free	1–4 h	60–88	[40]
15	Iridium	Paraformaldehyde	Reflux in H_2_O	5–10 h	41–91	[41]
16	Silver and gold surfaces	Formaldehyde	Solvent free	6 h	75–97	[42]
17	Gold nanoparticles (Au/Al_2_O_3_ or Au/NiO)	Methanol	Reflux in H_2_O	4 h	72–97	[43]
18	Ruthenium N-heterocyclic catalyst (Ru-NHC)	Methanol	Reflux in toluene (125 °C)	12–24 h	27–99	[44]
19	Copper salt (CuCl_2_.H_2_O)	Methanol	Solvent free	45–90 min	63–80	[45]
20	Ionic liquid catalyzed formylation	CO	Reflux in methanol (140 °C)	4 h	42–99	[18]
21	Inorganic ligand-supported chromium (III) catalyst (NH_4_)_3_[CrMo6O18(OH)_6_]	Methanol	Reflux in H_2_O_2_ (80 °C)	12 h	60–99	[46]
22	Lipase	Ethyl formate	Reflux in THF at room temperature	1–8 h	29–99	[14]
23	No catalyst	Triethyl orthoformate in water	Ultrasound irradiation	3 h	35–88	[1]
24	Catalyst free	Ammonium formate	Solvent free	5 min–24 h	43–98	[13]

**Table 2:** Silica-supported Brønsted acids as catalyst for the formylation of various amines.

Entry	Catalyst	Formylation Agent	Reaction Condition	Time	Yield %	Reference
1	HClO_4_^−^–SiO_2_	Formic acid	Solvent free	15–90 min	70–96	[25]
2	Fe_3_O_4_@SiO_2_–APTES-TFA	1,3-dicarbonyl compound	Solvent free	n/a	68–98	[34]
3	H_2_SO_4_–SiO_2_	Formic acid	Solvent free	4–46 min	65–99	[20]
4	H_2_SO_4_–SiO_2_	*N,N*-dimethyl amide	Solvent free	6–12 h	75–95	[25]

n/a: not applicable.

**Table 3:** Optimization of reaction parameters for *N*-formylation of amines with triethyl orthoformate (TEOF).

Entry	Reaction Condition	Time	Yield
1	Aniline (1 mmol)/TEOF (1 mmol), SIS (0.2 g)	10 min	44%
2	Aniline (1 mmol)/TEOF (2 mmol), SIS (0.2 g)	6 min	66%
3	Aniline (1 mmol)/TEOF (3 mmol), SIS (0.2 g)	4 min	96%
4	Aniline (1 mmol)/TEOF (4 mmol), SIS (0.2 g)	4 min	90%

**Table 4:** *N*-formylation of aniline under different catalytic conditions.

Entry	Catalytic Condition	Time	Yield
1	Aniline (1 mmol)/TEOF (3 mmol) without catalyst at 65 °C	3 h	traces
2	Aniline (1 mmol)/TEOF (3 mmol), SIS (0.1 g), 65 °C	5 min	78%
3	Aniline (1 mmol)/TEOF (3 mmol), SIS (0.2 g), 65 °C	4 min	96%
4	Aniline (1 mmol)/TEOF (3 mmol), SIS (0.3 g), 65 °C	4 min	88%
5	Aniline (1 mmol)/TEOF (3 mmol), SIS (0.4 g), 65 °C	6 min	71%
6	Aniline (1 mmol)/TEOF (3 mmol), SIS (0.5 g), 65 °C	6 min	64%

**Table 5:** *N*-formylation of amines using triethyl orthoformate in the presence of immobilized sulfuric acid on silica gel.

Entry	Amines	Time (Min)	Product	Yield (%)
1	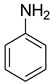	4	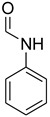	96
2	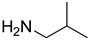	4	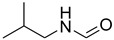	81
3	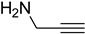	4	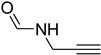	78
4	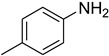	9	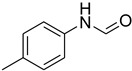	95
5	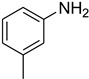	4	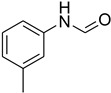	90
6	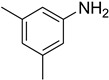	4	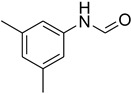	97
7	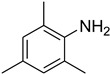	10	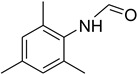	83
8	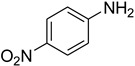	10	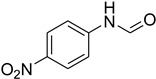	97
9	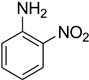	10	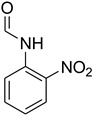	90
10	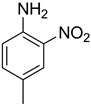	10	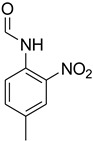	96
11	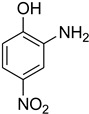	15	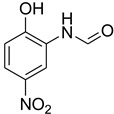	90
12	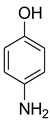	13	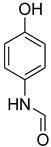	75
13	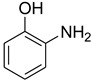	13	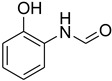	81
14	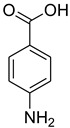	5	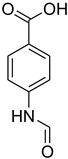	86
15	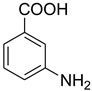	5	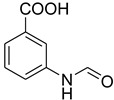	94
16	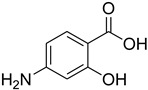	20	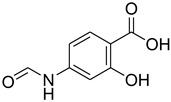	75
17	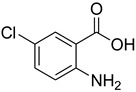	12	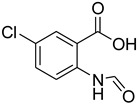	73
18	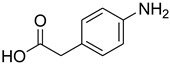	20	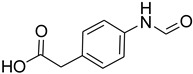	85
19	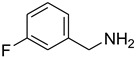	6	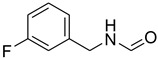	97
20	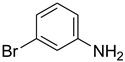	6	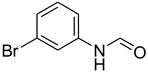	78
21	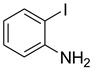	5	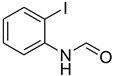	94
22	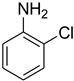	6	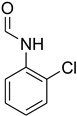	78
23	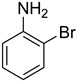	6	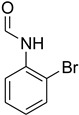	84
24	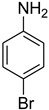	5	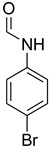	81
25	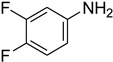	10	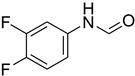	56
26	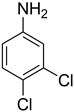	10	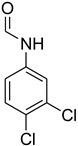	81
27	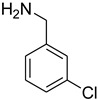	12	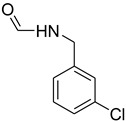	82
28	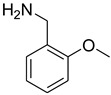	15	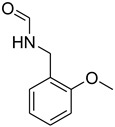	85
29	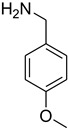	15	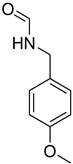	96
30	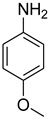	8	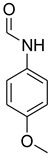	93
31	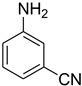	6	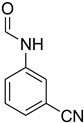	94
32	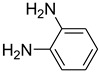	20	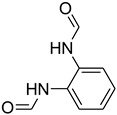	96
33	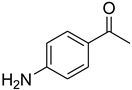	18	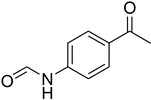	95
34	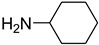	5	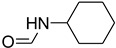	86
35	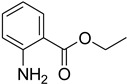	12	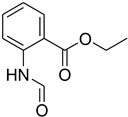	93
36	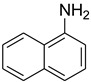	12	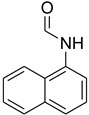	98
37	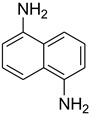	15	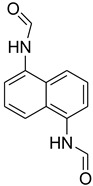	80
38	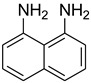	20	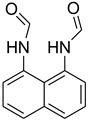	91
39	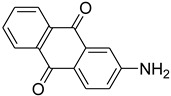	24	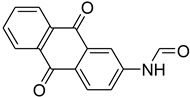	93
40	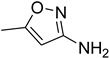	15	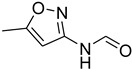	95
41	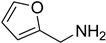	13	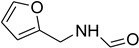	92
42	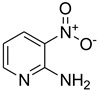	25	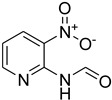	77
43	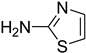	30	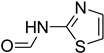	67
44	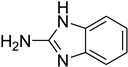	54	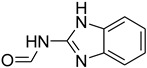	76
45	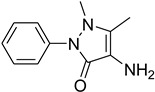	45	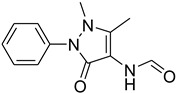	79
46	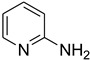	45	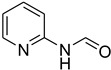	71
47	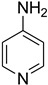	60	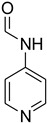	94
48	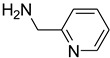	50	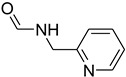	94
49	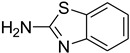	40	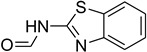	87
50	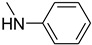	40	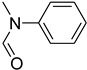	78
51	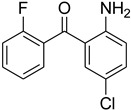	50	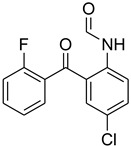	73
52	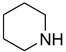	40	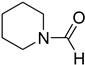	85
53	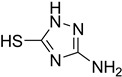	40	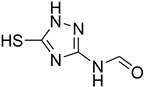	75
54	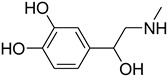	60	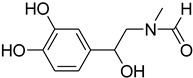	85
55	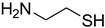	35	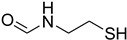	93
56	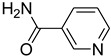	60	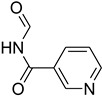	93

**Table 6:** Efficiency of the recycled SIS in the *N*-formylation of aniline.

Entry	Turn	Yield %
1	1	96
2	2	89
3	3	83

**Table 7:** Comparison of efficiency of various conditions in the *N*-formylation of aniline.

Entry	Conditions	Time	Yield	References
1	Triethyl orthoformate in H_2_O under ultrasound irradiation.	3 h	88%	[1]
2	Solid-supported formate, DMSO, 70–80 °C	4 h	60%	[15]
3	SSA, HCOOH, 50–60 °C, solvent-free	7 min	99%	[49]
4	SA on activated charcoal, ethylformate, 54 °C	4 min	95%	[21]
5	Triethyl orthoformate in H_2_O under neutral condition. Microwave irradiation, 90 °C	2 h	87%	[2]
6	SIS, triethyl orthoformate, 60–65 °C, solvent-free	3 min	96%	Present protocol

## Data Availability

Original data from experiments are available from the authors.

## Supplementary Materials

The following supporting information can be downloaded at: https://www.mdpi.com/article/10.3390/molecules27134213/s1: Figure S1–S56: NMR spectral data of synthesized compounds. References [53,54,55,56,57,58] are cited in the Supplementary Materials.

## Sample Availability

Samples of the compounds are available from the authors.

## References

[1] Habibi D., Sahebekhtiari H., Nasrollahzadeh M., Taghipour A. (2013). A Very Simple, Highly Efficient and Catalyst-free Procedure for the N-Formylation of Amines Using Triethyl orthoformate in Water Under Ultrasound-irradiation. Lett. Org. Chem..

[2] Kaboudin B., Khodamorady M. (2010). Organic reactions in water: A practical and convenient method for the N-formylation of amines in water. Synlett.

[3] Khatri C.K., Chaturbhuj G.U. (2017). Sulfated polyborate-catalyzed N-formylation of amines: A rapid, green and efficient protocol. J. Iran. Chem. Soc..

[4] Han Y., Cai L. (1997). An efficient and convenient synthesis of formamidines. Tetrahedron Lett..

[5] Gould-Fogerite S., Mannino R.J. (1997). Protein or peptide-cochleate vaccines and methods of immunizing using the same.

[6] Pravin P., Maryam A.M., Alexander D. (2020). Isocyanide 2.0. Green Chem..

[7] Roberts R.M., Vogt P.J. (1956). Ortho esters, imidic esters and amidines: N-alkylformanilides from alkyl orthoformates and primary aromatic amines; Rearrangement of alkyl N-arylformimidates. J. Am. Chem. Soc..

[8] de la Mare P.B.D. (1949). Kinetics of thermal addition of halogens to olefinic compounds. Q. Rev. Chem. Soc..

[9] Swaringen R.A., Eaddy J.F., Henderson T.R. (1980). Reaction of Ortho Esters with Secondary Amines. J. Org. Chem..

[10] Blicke F.F., Lu C.-J. (1952). Formylation of Amines with Chloral and Reduction of the N-Formyl Derivatives with Lithium Aluminum Hydride. J. Am. Chem. Soc..

[11] Kiho T., Yoshida I., Katsuragawa M., Sakushima M., Usui S., Ukai S. (1994). Polysaccharides in Fungi. XXXIV. A Polysaccharide from the Fruiting Bodies of Amanita muscaria and the Antitumor Activity of Its Carboxymethylated Product. Biol. Pharm. Bull..

[12] Jung S.H., Ahn J.H., Park S.K., Choi J.K. (2002). A practical and convenient procedure for the N-formylation of amines using formic acid. Bull. Korean Chem. Soc..

[13] Ganapati Reddy P., Kishore Kumar G.D., Baskaran S. (2000). A convenient method for the N-formylation of secondary amines and anilines using ammonium formate. Tetrahedron Lett..

[14] Rupesh Patre E., Sanjib Mal A., Pankaj R., Nilkanth R., Sujit Ghorai K., Sudhindra Deshpande H., Myriem Qacemi E.I., Smejkal T., Pal S., Manjunath B.N. (2017). First report on bio-catalytic N-formylation of amines using ethyl formate. Chem. Commun..

[15] Desai B., Danks T.N., Wagner G. (2005). Thermal and microwave-assisted N-formylation using solid-supported reagents. Tetrahedron Lett..

[16] Dhake K.P., Tambade P.J., Singhal R.S., Bhanage B.M. (2011). An efficient, catalyst- and solvent-free N-formylation of aromatic and aliphatic amines. Green Chem. Lett. Rev..

[17] Das B., Krishnaiah M., Balasubramanyam P., Veeranjaneyulu B., Nandan Kumar D. (2008). A remarkably simple N-formylation of anilines using polyethylene glycol. Tetrahedron Lett..

[18] Noh H.W., An Y., Lee S., Jung J., Son S.U., Jang H.Y. (2019). Metal-free Carbon Monoxide (CO) Capture and Utilization: Formylation of Amines. Adv. Synth. Catal..

[19] Zhang L., Han Z., Zhao X., Wang Z., Ding K. (2015). Highly efficient ruthenium-catalyzed N-formylation of amines with H_2_ and CO_2_. Angew. Chem. Int. Ed..

[20] Rasheed S., Rao D.N., Reddy A.S., Shankar R., Das P. (2015). Sulphuric acid immobilized on silica gel (H2SO4-SiO2) as an eco-friendly catalyst for transamidation. RSC Adv..

[21] Zeynizadeh B., Abdollahi M. (2016). The immobilized NaHSO_4_·H_2_O on activated charcoal: A highly efficient promoter system for N-formylation of amines with ethyl formate. Curr. Chem. Lett..

[22] Das V.K., Devi R.R., Raul P.K., Thakur A.J. (2012). Nano rod-shaped and reusable basic Al_2_O_3_ catalyst for N-formylation of amines under solvent-free conditions: A novel, practical and convenient NOSE’ approach. Green Chem..

[23] Madthukur Bhojegowd M.R., Nizam A., Pasha M.A. (2010). Amberlite IR-120: A reusable catalyst for N-formylation of amines with formic acid using microwaves. Cuihua Xuebao/Chin. J. Catal..

[24] Hosseini-sarvari M., Sharghi H. (2006). ZnO as a New Catalyst for N-Formylation of Amines under Solvent-Free Conditions. Tetrahedron.

[25] Zeynizadeh B. (2017). Catalytic Performance. J. Chem. Soc. Pak..

[26] Nasrollahzadeh M., Motahharifar N., Sajjadi M., Aghbolagh A.M., Shokouhimehr M., Varma R.S. (2019). Recent advances in N -formylation of amines and nitroarenes using efficient (nano)catalysts in eco-friendly media. Green Chem..

[27] Bahari S., Sajadi S.M. (2012). Natrolite zeolite: A natural and reusable catalyst for one-pot synthesis of α-aminophosphonates under solvent-free conditions. Arab. J. Chem..

[28] Kim J.G., Jang D.O. (2010). Indium-catalyzed N-formylation of amines under solvent-free conditions. Synlett.

[29] Krishnakumar B., Swaminathan M. (2011). A convenient method for the N-formylation of amines at room temperature using TiO2-P25 or sulfated titania. J. Mol. Catal. A Chem..

[30] Veer S.D., Pathare S.P., Akamanchi K.G. (2016). Sulfated tungstate catalyzed hydration of alkynes. Ark..

[31] Thirunarayanan G., Muthuvel I., Sathiyendiran V. (2015). Spectral LFER studies in some N-(substituted phenyl) formamides. Ann. Chem..

[32] Kim J.G., Jang D.O. (2010). Facile and highly efficient N-formylation of amines using a catalytic amount of iodine under solvent-free conditions. Synlett.

[33] Lei M., Ma L., Hu L. (2010). A convenient one-pot synthesis of formamide derivatives using thiamine hydrochloride as a novel catalyst. Tetrahedron Lett..

[34] Jafarzadeh M., Soleimani E., Norouzi P., Adnan R., Sepahvand H. (2015). Preparation of trifluoroacetic acid-immobilized Fe_3_O_4_@SiO_2_-APTES nanocatalyst for synthesis of quinolines. J. Fluor. Chem..

[35] Pathare S.P., Sawant R.V., Akamanchi K.G. (2012). Sulfated tungstate catalyzed highly accelerated N-formylation. Tetrahedron Lett..

[36] De Luca L., Giacomelli G., Porcheddu A., Salaris M. (2004). A new, simple procedure for the synthesis of formyl amides. Synlett.

[37] Deutsch J., Eckelt R., Köckritz A., Martin A. (2009). Catalytic reaction of methyl formate with amines to formamides. Tetrahedron.

[38] Baghbanian S.M., Farhang M. (2013). Protic [TBD][TFA] ionic liquid as a reusable and highly efficient catalyst for N-formylation of amines using formic acid under solvent-free condition. J. Mol. Liq..

[39] Chandra Shekhar A., Ravi Kumar A., Sathaiah G., Luke Paul V., Sridhar M., Shanthan Rao P. (2009). Facile N-formylation of amines using Lewis acids as novel catalysts. Tetrahedron Lett..

[40] Hong M., Xiao G. (2013). Hafnium(IV) bis(perfluorooctanesulfonyl)imide complex supported on fluorous silica gel catalyzed N-formylation of amines using aqueous formic acid. J. Fluor. Chem..

[41] Ourida S., Mark J.B., John B., James L., Stephen P.M., Pawel P., Robert J.W., Williams J.M.J. (2010). Iridium-catalyzed formylation of amines with paraformaldehyde. Tetrahedron Lett..

[42] Lundberg H. (2015). Group (IV) Metal-Catalyzed Direct Amidation: Synthesis and Mechanistic Considerations. Ph.D. Thesis.

[43] Ishida T., Haruta M. (2009). N-formylation of amines via the aerobic oxidation of methanol over supported gold nanoparticles. ChemSusChem.

[44] Ortega N., Richter C., Glorius F. (2013). N-Formylation of amines by methanol activation. Org. Lett..

[45] Tumma H., Nagaraju N., Reddy K.V. (2009). A facile method for the N-formylation of primary and secondary amines by liquid phase oxidation of methanol in the presence of hydrogen peroxide over basic copper hydroxyl salts. J. Mol. Catal. A Chem..

[46] Han Y., Zhikang W., Zheyu W., Yongyan Z., Shi R., Qixin Z., Jingjing W., Sheng H., Yongge W. (2019). N-formylation of amines using methanol as a potential formyl carrier by a reusable chromium catalyst. Commun. Chem..

[47] Kaur M., Sharma S., Bedi P.S. (2015). Silica supported Brönsted acids as catalyst in organic transformations: A comprehensive review. Cuihua Xuebao/Chinese J. Catal..

[48] Pramanik A., Bhar S. (2021). Silica-sulfuric acid and alumina-sulfuric acid: Versatile supported Brønsted acid catalysts. New J. Chem..

[49] Habibi D., Rahmani P., Akbaripanah Z. (2013). N-formylation of anilines with silica sulfuric acid under solvent free conditions. J. Org. Chem..

[50] Hu D.X., Grice P., Ley S.V. (2012). Rotamers or diastereomers? An overlooked NMR solution. J. Org. Chem..

[51] Lanyon-Hogg T., Ritzefeld M., Masumoto N., Magee A.I., Rzepa H.S., Tate E.W. (2015). Modulation of Amide Bond Rotamers in 5-Acyl-6,7-dihydrothieno [3,2-c]pyridines. J. Org. Chem..

[52] Habibi D., Nasrollahzadeh M., Sahebekhtiari H. (2013). Green synthesis of formamides using the Natrolite zeolite as a natural, efficient and recyclable catalyst. J. Mol. Catal. A Chem..

[53] Ma’mani L., Sheykhan M., Heydari A., Faraji M., Yamini Y. (2010). Sulfonic acid supported on hydroxyapatite-encapsulated-γ-Fe_2_O_3_ nanocrystallites as a magnetically Brønsted acid for N-formylation of amines. Appl. Catal. A Gen..

[54] Bose A.K., Ganguly S.N., Manhas M.S., Guha A., Pombo-Villars A. (2006). Microwave promoted energy-efficient N-formylation with aqueous formic acid. Tetrahedron Lett..

[55] Lygin A.V., De Meijere A. (2009). ortho-Lithiophenyl isocyanide: A versatile precursor for 3H-quinazolin-4-ones and 3H-quinazolin-4-thiones. Org. Lett..

[56] Landquist J.K. (1951). Synthetic antimalarials. Part XLVI. Some 4-[(dialkylaminoalkyl)amino] quinoline derivatives. J. Chem. Soc..

[57] Kim J.J., Park Y.D., Cho S.D., Kim H.K., Chung H.A., Lee S.G., Falck J.R., Yoon Y.J. (2004). Efficient N-arylation of pyridazin-3(2H)-ones. Tetrahedron Lett..

[58] Trost B.M. (1991). The Atom Economy—A Search for Synthetic Efficiency. Science.

