# Supplementary material for: Catalytic Performance of Immobilized Sulfuric Acid on Silica Gel for N-Formylation of Amines with Triethyl Orthoformate
Source: Molecules. 2022 Jun 30;27(13):4213. doi: 10.3390/molecules27134213 (PMC9268625; doi:10.3390/molecules27134213)
Supplement: Supplementary file 1 [file molecules-27-04213-s001.zip › molecules-1720771-supplementary.pdf]

Supplementary Materials

Catalytic Performance of Immobilized Sulfuric Acid on Silica Gel for N-Formylation of Amines with Triethyl Orthoformate

Sodeeq Aderotimi Salami [1], Xavier Siwe-Noundou [2] and Rui Werner Maçedo Krause [1,*]

1. Department of Chemistry, Rhodes University, Grahamstown 6140, South Africa.
2. Department of `Pharmaceutical Sciences, School of Pharmacy, Sefako Makgatho Health Sciences University, P.O Box 218 Pretoria, 0204, South Africa.

* Correspondence: sodeeqaderotimi@gmail.com; Tel.: +27 83 302 3511 (S.A.S.); xavier.siwenoundou@smu.ac.za; Tel +27 12 521 5647 (X.S.-N.); r.krause@ru.ac.za; Tel +27 46 603 7030 (R.W.M.K)

Abstract: In the search for convenient, green, and practical catalytic methods for the current interest in or-ganic synthesis, a simple, green, and highly efficient protocol for N-formylation of various amines was carried out in the presence of immobilized sulfuric acid on silica gel (H2SO4–SiO2). All reactions were performed in refluxing triethyl orthoformate (65 °C). The product formamides were obtained with high-to-excellent yields within 4 min to 2 h. The current approach is advantageous, due to its short reaction time and high yields. The catalyst is recyclable with no significant loss in catalytic efficiency.

Keywords: *N*-formylation; amines; immobilized sulfuric acid; silica gel; triethyl orthoformate.

Table of Contents

	Page
**1. Experimental**	**S2**
**1.1. Materials and methods**	**S2**
**1.2. Preparation of sulphuric acid adsorbed on silica gel (H_2SO_4–SiO_2)**	**S2**
**1.3. General procedure for the *N*-formylation reaction**	**S3**
**2. Spectral data of synthsized compounds**	**S32**
**3. Mass spectroscopy of the new derivatives 38 and 53**	**S94**
**4. Green Metrics S4**	**S95**

1. Experimental

1.1. Materials and Methods

A PerkinElmer Spectrum 100 FT-IR Spectrometer was used for the FT-IR analysis. The IR spectra were obtained by the attenuated total reflection (ATR) method. For each experiment, 16 scans were performed in the frequency range from 650 to 4000 cm^{-1}. Melting points of all the compounds were determined using a Koffler hot-stage apparatus and are uncorrected. NMR spectra were recorded on a Bruker AMX400 spectrometer using $CDCl_3$ or DMSO-d_6 as a solvent with tetramethylsilane used as internal standard. Solvents and chemicals used were of analytical grade, which were purchased from Sigma Aldrich, and used without further purification. The purity determination of the starting materials and reaction monitoring was performed by thin-layer chromatography (TLC) on Merck silica gel G F_{254}plates. All the products are known compounds and were identified by melting point and ^{1}H NMR spectroscopy except 1,8-difformamido naphthalein and 3-Formamido-1,2,4-triazole-5-thiol which are new derivatives. The analytical data were compared with the literature values. [1–10]

1.2. Preparation of Sulphuric Acid Adsorbed on Silica Gel (H_2SO_4–SiO_2)

The preparation of H_2SO_4–SiO_2 was carried out by following reported procedure.[1] To a suspension of silica gel (29.5 g, 230–400 mesh size) in EtOAc (60 mL) was added H_2SO_4 (1.5 g, 15.5 mmol, 0.8 mL of a 98% aq. solution of H_2SO_4) and the mixture was stirred magnetically for 30 min at room temperature. The EtOAc was removed under reduced pressure (rotary evaporator) and the residue was heated at 100 $^{\circ}$C for 72 h under vacuum to afford H_2SO_4–SiO_2 as a free-flowing powder.

1.3. A General Procedure for *N*-formylation of Amines with Triethyl Orthoformate Promoted by Immobilized H_2SO_4 on Silica Gel.

1.3.1. *N*-phenyl formamide

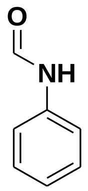

To a mixture of Aniline (0.548 mL, 6 mmol) and triethyl orthoformate (24 mmol). The immobilized H_2SO_4 on silica gel (1.2 g) was then added and the reaction mixture was stirred under reflux conditions (65 °C). Progress of the reaction was monitored by TLC. After completion of the reaction, the mixture was diluted with EtOAc (20 mL), filtered, water (30 mL) added, the solution extracted with EtOAc, and the combined organic layers were dried over anhydrous Na_2SO_4 and concentrated. The residue was subjected to column chromatography and eluted with (EtOAc-Pet Ether (3:1)) to afford *N*-Phenylformamide **(1)**. (0.52 g, 95%). Dark brown liquid. IR (NaCl) ν(cm^{-1}) 3349, 3213, 3150, 2896, 1700, 1686, 1530, 1436, 1350, 1261. Mixture of rotamers were observed.^{1}H NMR (400 MHz, $CDCl_3$) Major rotamer: −δ 8.45 (s, 1H), 8.22 (d, *J* = 2.0 Hz, 1H), 7.46 (dd, *J* = 8.5, 0.9 Hz, 2H), 7.12 – 6.95 (m, 4H). ^{13}C NMR (101 MHz, $CDCl_3$) δ 159.93, 137.09, 129.21, 124.80, 118.95. ^{1}H NMR (400 MHz, $CDCl_3$) Minor rotamer: −δ 9.15 (d, *J* = 10.0 Hz, 1H), 8.59 (d, *J* = 11.4 Hz, 1H), 7.46 (dd, *J* = 8.5, 0.9 Hz, 2H), 7.27 – 7.15 (m, 4H). ^{13}C NMR (101 MHz, $CDCl_3$) δ 163, 137, 129, 125.29, 120.

1.3.2. *N*-isobutylformamide

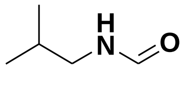

The experimental procedure described for the synthesis of *N*-phenyl formamide **1** was followed using isobutylamine (0.596 mL, 6 mmol) and triethyl orthoformate (24 mmol) and (1.2 g) of immobilized H_2SO_4 on silica gel. The mixture was subjected to purification and the eluted solvent (EtOAc) was then evaporated under reduced pressure to afford as a yellow oil *N*-isobutylformamide **(2)**. (0.48 g, 80.5%).

IR (NaCl) v(cm^{-1}) 3312 2951 1936 1653 1534 1463 1368. Mixture of rotamers were observed. ^{1}H NMR (400 MHz, Chloroform-*d*) Major rotamer: −δ 7.57 (s, 1H), 3.65 – 2.65 (m, 2H), 1.70 – 1.18 (m, 1H), 0.96 – 0.56 (m, 3H). ^{13}C NMR (101 MHz, $CDCl_3$) δ 159.91, 47, 35, 28, 20, 9. ^{1}H NMR (400 MHz, Chloroform-*d*) Minor rotamer: −δ 6.86 (s, 1H), 2.62 – 1.70 (m, 4H), 1.18 – 0.96 (m, 1H), ^{13}C NMR (101 MHz, $CDCl_3$) δ 152, 46, 30, 24, 19, 9.

13.3. *N*-(prop-2-ynyl)formamide

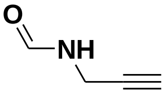

The experimental procedure described for the synthesis of *N*-phenyl formamide **1** was followed using proparglamine (0.36 mL, 6 mmol) and triethyl orthoformate (24 mmol) and (1.2 g) of immobilized H_2SO_4 on silica gel. The mixture was subjected to purification and the eluted solvent (EtOAc) was then evaporated under reduced pressure to afford a yellow oil *N*-(prop-2-ynyl)formamide **(3)** (0.28 g, 78%). IR (NaCl) v(cm^{-1}) 3390, 3047, 2923, 2356, 1645, 1524, 1379, 1235, 1031. Mixture of rotamers were observed. ^{1}H NMR (400 MHz, $CDCl_3$) Major rotamer: −δ 8.32 (s, 1H), 8.02 (s, 1H), 3.91 (s, 2H), 2.22 (s, 1H). ^{13}C NMR (101 MHz, $CDCl_3$) δ 161, 71, 27. Minor rotamer: ^{1}H NMR (400 MHz, $CDCl_3$) −δ 7.73 (d, J = 39.6 Hz, 1H), 5.12 (d, J = 207.9 Hz, 1H), 3.65 (s, 1H), 2.35 (s, 1H). ^{13}C NMR (101 MHz, $CDCl_3$) δ 165, 79, 31.

1.3.4. *N*-p-tolylformamide

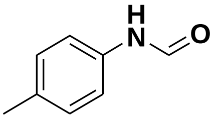

The experimental procedure described for the synthesis of *N*-phenyl formamide **1** was followed using p-toluidine (0.644 g, 6 mmol) and triethyl orthoformate (24 mmol) and (1.2 g) of immobilized H_2SO_4 on silica gel. The mixture was subjected to purification and the eluted solvent (EtOAc) was then evaporated under reduced pressure to afford a white solid *N*-p-tolylformamide **(4)** (0.61g 95%); mp 50–54 °C (lit. 50–54 °C)[2]; IR (NaCl) v(cm^{-1}) 3360, 2913, 2854, 1686, 1515, 1368, 1211. Mixture of rotamers were observed. ^{1}H NMR (400 MHz, $CDCl_3$) Major rotamer: −δ 9.19 (d, J = 10.7 Hz, 1H), 8.65

(d, J = 11.4 Hz, 1H), 7.46 (d, J = 8.4 Hz, 2H), 7.01 (d, J = 8.4 Hz, 2H), 2.31 (s, 3H). ^{13}C NMR (101 MHz, $CDCl_3$) δ 163, 134, 130, 120. Minor rotamer: ^{1}H NMR (400 MHz, $CDCl_3$) −δ 8.59 (s, 1H), 8.30 (d, J = 2.0 Hz, 1H), 7.13 (dd, J = 14.5, 8.3 Hz, 4H), 2.34 (s, 3H). ^{13}C NMR (101 MHz, $CDCl_3$) δ 159, 133, 129, 119.

1.3.5. *N*-m-tolylformamide

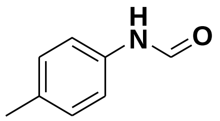

The experimental procedure described for the synthesis of *N*-phenyl formamide **1** was followed using m-toluidine (0.63 mL, 6 mmol) and triethyl orthoformate (24 mmol) and (1.2 g) of immobilized H_2SO_4 on silica gel. The mixture was subjected to purification and the eluted solvent (EtOAc) was then evaporated under reduced pressure to afford a brown liquid *N*-m-tolylformamide **(5)** (0.57 g, 90%). Mixture of rotamers were observed. ^{1}H NMR (400 MHz, $CDCl_3$) Major rotamer: δ 9.14 (d, J = 8.6 Hz, 1H), 8.60 (d, J = 11.4 Hz, 1H), 7.25 (d, J = 8.1 Hz, 1H), 7.10 (dd, J = 20.6, 8.0 Hz, 2H), 6.82 (t, J = 6.3 Hz, 3H), 2.23 (s, 3H). ^{13}C NMR (101 MHz, $CDCl_3$) −δ 163, 139, 136, 129, 125, 119, 115. Minor rotamer: ^{1}H NMR (400 MHz, $CDCl_3$) −δ 8.30 (s, 1H), 8.21 (d, J = 2.0 Hz, 1H), 7.31 (s, 1H), 7.14 – 7.05 (m, 2H), 6.89 (d, J = 7.6 Hz, 1H), 2.20 (s, 3H). ^{13}C NMR (101 MHz, $CDCl_3$) δ 160, 138, 128, 125, 120, 117.

1.3.6. *N*-(3,5-Dimethylphenyl)formamide

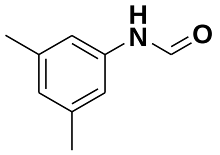

The experimental procedure described for the synthesis of *N*-phenyl formamide **1** was followed using 3,5-dimethyl aniline (0.748 mL, 6 mmol) and triethyl orthoformate (24 mmol) and (1.2 g) of immobilized H_2SO_4 on silica gel. The mixture was subjected to purification and the eluted solvent (EtOAc) was then evaporated under reduced pressure to afford a yellow solid *N*-(3,5-dimethyl) phenylformamide **(6)** (0.73 g, 97%) mp 168–170 °C (lit. 168–169 °C)[3]. IR (NaCl) v(cm^{-1}) 3321

3013.5 2918 1669 1601 1396 1282. Mixture of rotamers were observed. ^{1}H NMR (400 MHz, $CDCl_3$) Major rotamer: ^{1}H NMR (400 MHz, $CDCl_3$) −δ 8.41 (s, 1H), 7.57 (s, 1H), 7.35 (s, 1H), 6.78 (d, *J* = 7.1 Hz, 9H), 2.38 (s, 28H). ^{13}C NMR (101 MHz, $CDCl_3$) δ 163, 149, 139, 139, 117, 113, 21. Minor rotamer: ^{1}H NMR (400 MHz, $CDCl_3$) −δ 9.13 (s, 1H), 8.78 (d, *J* = 10.6 Hz, 1H), 8.29 (s, 1H), 6.88 (d, *J* = 14.4 Hz, 2H), 2.14 (s, 1H). ^{13}C NMR (101 MHz, $CDCl_3$) δ 159, 145, 139, 137, 126, 120, 118, 14.

1.3.7. *N*-mesitylformamide

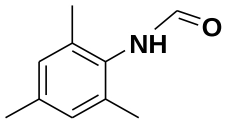

The experimental procedure described for the synthesis of *N*-phenyl formamide **1** was followed using 2,4,6-trimethylaniline (0.8 mL, 6 mmol) and triethyl orthoformate (24 mmol) and (1.2 g) of immobilized H_2SO_4 on silica gel. The mixture was subjected to purification and the eluted solvent (EtOAc) was then evaporated under reduced pressure to afford a white solid *N*-mesitylformamide **(7)** (0.66 g, 82.5%) mp 179–180 °C (lit.181–182 °C)[3]. IR (NaCl) *v*(cm^{-1}) 3389, 2970, 2915, 2868, 1653, 1532, 1489, 1391, 1254, 1012. Mixture of rotamers were observed ^{1}H NMR (400 MHz, $CDCl_3$) Major rotamer: −δ 8.31 (s, 1H), 6.91 (d, *J* = 21.0 Hz, 4H), 6.09 (s, 2H), 2.28 (d, *J* = 16.2 Hz, 12H), 2.21 (s, 2H). ^{13}C NMR (101 MHz, $CDCl_3$) δ 166, 139, 137, 134, 18, 17. ^{1}H NMR (400 MHz, $CDCl_3$) Minor rotamer: −δ 8.03 (d, *J* = 7.2 Hz, 1H), 7.94 (d, *J* = 9.9 Hz, 1H), 7.27 (s, 1H), 6.78 (s, 1H), 2.21 (s, 1H), 2.17 (d, *J* = 5.9 Hz, 4H). ^{13}C NMR (101 MHz, $CDCl_3$) δ 164, 131, 129, 129, 122, 21, 20.

1.3.8. *N*-(4-nitrophenyl) formamide

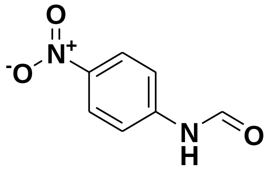

The experimental procedure described for the synthesis of *N*-propyl formamide **1** was followed using 4-nitroaniline (0.83 g, 6 mmol) and triethyl orthoformate (24 mmol) and (1.2 g) of immobilized H_2SO_4

on silica gel. The mixture was subjected to purification and the eluted solvent (EtOAc) was then evaporated under reduced pressure to afford yellow solid *N*-(4-nitrophenyl)formamide **(8)** (0.81 g, 97%) mp 196–198 °C (lit. 194–196 °C)[4]. IR (NaCl) *v*(cm^{-1}) 3481, 3343, 3116, 2946, 1732, 1624, 1581, 1444, 1275. ^{1}H NMR (400 MHz, DMSO-d_6) −δ 7.94 (d, *J* = 10.3 Hz, 1H), 6.71 (s, 1H), 6.58 (d, *J* = 10.3 Hz, 1H), 3.38 (s, 2H). ^{13}C NMR (101 MHz, DMSO -d_6) δ 155, 135, 126, 112.

1.3.9. *N*-(2-nitrophenyl) formamide

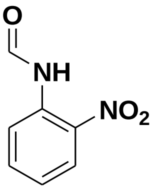

The experimental procedure described for the synthesis of *N*-phenyl formamide **1** was followed using 2-nitro aniline (0.83 g, 6 mmol) and triethyl orthoformate (24 mmol) and (1.2 g) of immobilized H_2SO_4 on silica gel. The mixture was subjected to purification and the eluted solvent (EtOAc) was then evaporated under reduced pressure to afford a yellow solid *N*-(2-nitrophenyl) formamide **(9)** (0.74 g, 90%) mp 175–177 °C (lit. 174–175 °C)[5]. IR (NaCl) *v*(cm^{-1}) 3470, 3341, 3275, 2946, 2360, 2126, 1715, 1684, 1489, 1246. Mixture of rotamers were observed. ^{1}H NMR (400 MHz, $CDCl_3$) Major rotamer: δ- 8.57 (s, 1H), 8.05 (dd, *J* = 8.6, 1.5 Hz, 1H), 7.36 – 7.28 (m, 1H), 6.80 (dd, *J* = 8.4, 1.2 Hz, 1H), 5.93 (s, 3H). ^{13}C NMR (101 MHz, $CDCl_3$) δ 159, 144, 135, 133, 126, 124, 118. Minor rotamer: ^{1}H NMR (400 MHz, $CDCl_3$) −δ 10.29 (s, 1H), 8.75 (d, *J* = 8.1 Hz, 1H), 8.19 (dd, *J* = 8.5, 1.5 Hz, 1H), 7.69 – 7.58 (m, 1H), 7.20 (t, *J* = 7.7 Hz, 1H), 6.65 (ddd, *J* = 8.4, 7.0, 1.3 Hz, 2H). ^{13}C NMR (101 MHz, $CDCl_3$) δ 159, 144, 136, 131, 125, 122, 116.

1.3.10. *N*-(4-methyl−2-nitrophenyl)formamide

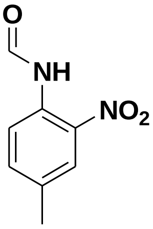

The experimental procedure described for the synthesis of *N*-phenyl formamide **1** was followed using 4-methyl-2-nitroaniline (0.9 g, 6 mmol) and triethyl orthoformate (24 mmol) and (1.2 g) of immobilized H_2SO_4 on silica gel. The mixture was subjected to purification and the eluted solvent (EtOAc) was then evaporated under reduced pressure to afford a reddish brown solid *N*-(4-methyl-2-nitrophenyl)formamide **(10)** (0.86 g, 96%) mp 124–126 °C (lit. 124–125 °C)[3]. IR (NaCl) ν(cm^{-1}) 3470, 3349, 3251, 2923, 1704, 1672, 1563, 1407, 1332, 1231, 1149. Mixture of rotamers were observed. ^{1}H NMR (400 MHz, $CDCl_3$) Major rotamer: −δ 10.17 (s, 1H), 8.62 (d, *J* = 8.5 Hz, 1H), 7.99 (s, 1H), 7.44 (d, *J* = 10.5 Hz, 1H), 6.71 (d, *J* = 8.5 Hz, 1H), 2.37 (s, 4H). ^{13}C NMR (101 MHz, $CDCl_3$) δ 159, 142, 136, 130, 124, 122, 20. Minor rotamer: ^{1}H NMR (400 MHz, $CDCl_3$) −δ 9.65 (s, 1H), 8.84 (s, 1H), 8.53 (s, 1H), 7.85 (s, 1H), 7.15 (d, *J* = 10.5 Hz, 1H), 2.23 (s, 1H). ^{13}C NMR (101 MHz, $CDCl_3$) δ 161, 153, 135, 131, 126, 118, 19.

1.3.11. *N*-(2-hydroxy-5-nitrophenyl) formamide

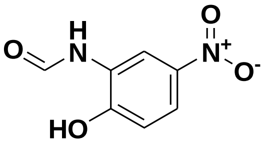

The experimental procedure described for the synthesis of *N*-phenyl formamide **1** was followed using 2-hydroxy-5-nitroaniline (0.925 g, 6 mmol) and triethyl orthoformate (24 mmol) and (1.2 g) of immobilized H_2SO_4 on silica gel. The mixture was subjected to purification and the eluted solvent (EtOAc) was then evaporated under reduced pressure to afford a yellow solid *N*-(2-hydroxy-5-nitrophenyl) formamide **(11)** (0.83 g, 90%) mp 116–118 °C. IR (NaCl) ν(cm^{-1}) 3118 2915 1632 1510.6 1349 1120.7. ^{1}H NMR (400 MHz, $CDCl_3$) −δ 8.70 (s, 1H), 8.35 (s, 1H), 8.27 (s, 1H), 7.72 (d, *J* = 10.3 Hz, 1H). ^{13}C NMR (101 MHz, $CDCl_3$) δ 155, 153, 145, 140, 122, 117, 111.

1.3.12. *N*-(4-hydroxyphenyl)formamide

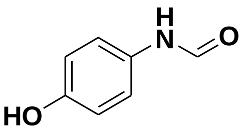

The experimental procedure described for the synthesis of *N*-phenyl formamide **1** was followed using 2-aminophenol. (0.654 g, 6 mmol) and triethyl orthoformate (24 mmol) and (1.2 g) of immobilized H_2SO_4 on silica gel. The mixture was subjected to purification and the eluted solvent (EtOAc/Pet-Ether (3:1)) was then evaporated under reduced pressure to afford a brown solid *N*-(4-hydroxyphenyl)formamide **(12)** (0.49 g, 75%) mp 136–138 °C (lit. 139 °C). The compound was identified by spectral comparison with literature data. IR (NaCl) v(cm^{-1}) 3308 3118 2970 2808 1720 1653 1505 1401 1244. Mixture of rotamers were observed.^{1}H NMR (400 MHz, DMSO-d_6). Major rotamer: −δ 9.25 (s, 1H), 8.34 (s, 1H), 7.37 (d, J = 8.8 Hz, 2H), 6.70 (d, J = 8.8 Hz, 2H), 3.36 (s, 1H). ^{13}C NMR (101 MHz, DMSO-d_6) δ 163, 153, 148, 130, 121, 115. ^{1}H NMR (400 MHz, DMSO-d_6). Minor rotamer: −δ 9.90 (s, 1H), 8.51 (d, J = 11.2 Hz, 1H), 6.99 (d, J = 8.7 Hz, 1H), 6.44 (d, J = 16.3 Hz, 1H). ^{13}C NMR (101 MHz, DMSO-d_6) δ 163, 153, 141, 130, 120, 115.

1.3.13. *N*-(2-Hydroxy phenyl) formamide

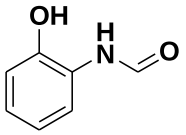

The experimental procedure described for the synthesis of *N*-phenyl formamide **1** was followed using 2-aminophenol. (0.654 g, 6 mmol) and triethyl orthoformate (24 mmol) and (1.2 g) of immobilized H_2SO_4 on silica gel. The mixture was subjected to purification and the eluted solvent (EtOAc/Pet-Ether) (3:1)) was then evaporated under reduced pressure to afford a white solid *N*-(2-Hydroxy phenyl) formamide **(13)** (0.53 g, 81%). mp 128–130 °C (lit. 129–131 °C) [4]. IR (NaCl) v(cm^{-1}) 3481, 3343, 3145, 2961, 1720, 1624, 1581, 1444, 1275. Mixture of rotamers were observed. ^{1}H NMR (400 MHz, $CDCl_3$) Major rotamer: −δ 8.20 (d, J = 1.7 Hz, 1H), 7.56 (s, 1H), 7.13 (dd, J = 7.9, 1.4 Hz, 1H), 7.08 (td, J = 7.9, 1.5 Hz, 1H), 6.95 (dd, J = 8.1, 1.2 Hz, 1H), 6.83 (td, J = 7.8, 1.3 Hz, 1H), 4.27 (s, 1H). ^{13}C NMR (101 MHz, $CDCl_3$) δ 159, 147, 127, 121, 120, 119. Minor rotamer: ^{1}H NMR (400 MHz, $CDCl_3$) −δ 8.61 (d, J = 11.6 Hz, 1H), 6.74 (d, J = 1.4 Hz, 1H), 6.73 – 6.68 (m, 11H), 6.66 – 6.58 (m, 7H), 2.46 (s, 6H). ^{13}C NMR (101 MHz, $CDCl_3$) δ 159, 143, 124, 120, 119.

1.3.14. 4-Formamidobenzoic acid

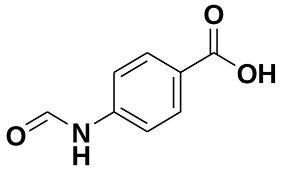

The experimental procedure described for the synthesis of *N*-phenyl formamide **1** was followed using 3-aminobenzoic acid (0.823 g, 6 mmol) and triethyl orthoformate (24 mmol) or and (1.2 g) of immobilized H_2SO_4 on silica gel. The mixture was subjected to purification and the eluted solvent (EtOAc) was then evaporated under reduced pressure to afford a white solid 4-formamidobenzoic acid **(14)** (0.71 g, 86%). mp 250–252 °C. IR (NaCl) v(cm^{-1}) 3457, 3363, 3128, 2874, 1655, 1596, 1416, 1287. ^{1}H NMR (400 MHz, DMSO-d_6) −δ 12.0 (s, 0H). 7.61 (d, *J* = 8.6 Hz, 1H), 6.53 (d, *J* = 8.7 Hz, 1H), 5.87 (s, 1H), 3.37 (s, 1H). ^{13}C NMR (101 MHz, DMSO-d_6) δ 167, 153, 131, 117, 112.

1.3.15. 3-Formamidobenzoic acid

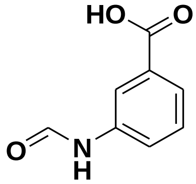

The experimental procedure described for the synthesis of *N*-phenyl formamide **1** was followed using 3-aminobenzoic acid (0.823 g, 6 mmol) and triethyl orthoformate (24 mmol) and (1.2 g) of immobilized H_2SO_4 on silica gel. The mixture was subjected to purification and the eluted solvent (Pet/EtOAc 3:1) was then evaporated under reduced pressure to afford 3-formamidobenzoic acid **(15)** (0.77 g, 94%). mp 155–158 °C. IR (NaCl) v(cm^{-1}) 3300, 3125, 2918, 2250, 1644, 1558, 1382, 1220. ^{1}H NMR (400 MHz, DMSO-d_6) −δ 13.00 (s, OH), 10.38 (s, 1H), 10.29 (d, *J* = 10.9 Hz, 1H), 8.85 (d, *J* = 10.9 Hz, 1H), 8.27 (d, *J* = 35.9 Hz, 3H), 7.80 (d, *J* = 7.9 Hz, 2H), 7.64 (d, *J* = 7.6 Hz, 2H), 7.46 (dt, *J* = 15.9, 8.6 Hz, 2H), 7.20 – 6.91 (m, 1H), 3.35 (s, 17H). ^{13}C NMR (101 MHz, DMSO-d_6) δ 167, 159, 138, 131, 129, 124, 123, 119.

1.3.16. 4-formamido-2-hydroxybenzoic acid

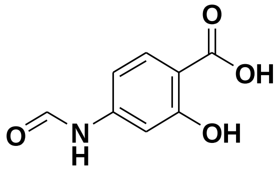

The experimental procedure described for the synthesis of *N*-propyl formamide **1** was followed using 4-amino salicylic acid (0.91 g, 6 mmol) and triethyl orthoformate (24 mmol) and (1.2 g) of immobilized H_2SO_4 on silica gel. The mixture was subjected to purification and the eluted solvent (Pet-Ether/EtOAc (1:3)) was then evaporated under reduced pressure to afford a yellow viscous liquid 4-formamido-2-hydroxybenzoic acid **(16)** (0.68 g, 75%). IR (NaCl) v(cm^{-1}) 3384, 3126, 2890, 1748, 1648, 1558, 1377, 1215. ^{1}H NMR (400 MHz, DMSO-d_6) −δ 7.41 (d, J = 8.6 Hz, 1H), 6.07 (dd, J = 8.6, 2.0 Hz, 1H), 5.96 (d, J = 2.0 Hz, 1H), 3.63 (s, 1H), 3.34 (s, 4H). ^{13}C NMR (101 MHz, DMSO-d_6) δ 172, 163, 155, 131, 106, 98.

1.3.17. 5-chloro-2-formamidobenzoic acid

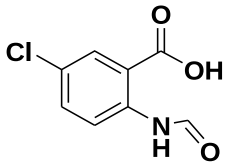

The experimental procedure described for the synthesis of *N*-phenyl formamide **1** was followed using 2-amino-5-chlorobenzoic acid (1.02 g, 6 mmol) and triethyl orthoformate (24 mmol) and (1.2 g) of immobilized H_2SO_4 on silica gel. The mixture was subjected to purification and the eluted solvent (EtOAc) was then evaporated under reduced pressure to afford a white solid 5-chloro-2-formamidobenzoic acid **(17)** (7.45 g, 73%) mp 204–206 °C (lit. 205–207 °C)[3]. IR (NaCl) v(cm^{-1}) 3469, 3351, 2808, 1667, 1586, 1472, 1201. Mixture of rotamers were observed. ^{1}H NMR (400 MHz, DMSO-d_6) Major rotamer: −δ 10.98 (s, 1H), 8.56 (d, J = 8.9 Hz, 1H), 7.92 (d, J = 2.6 Hz, 1H), 7.62 (d, J = 2.6 Hz, 2H), 6.78 (d, J = 8.9 Hz, 2H). ^{13}C NMR (101 MHz, DMSO-d_6) δ 169, 161, 150, 133, 130, 122, 118, 110. Minor rotamer: ^{1}H NMR (400 MHz, DMSO-d_6) −δ 10.49 (s, 1H), 8.98 (s, 1H), 8.51 (s, 1H), 7.66 (dd, J = 8.9, 2.5 Hz, 1H), 7.25 (dd, J = 8.9, 2.7 Hz, 1H). ^{13}C NMR (101 MHz, DMSO-d_6) δ 167, 161, 150, 138, 130, 127, 117.

1.3.18. 4-Formamido phenyl acetic acid

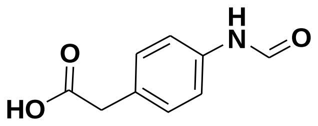

The experimental procedure described for the synthesis of *N*-phenyl formamide **1** was followed using 4-amino phenyl acetic acid (0.906 g, 6 mmol) and triethyl orthoformate (24 mmol) and (1.2 g) of immobilized H_2SO_4 on silica gel. The mixture was subjected to purification and the eluted solvent (EtOAc) was then evaporated under reduced pressure to afford a white solid 4-Formamido phenyl acetic acid **(18)** (0.77 g, 85%) mp 142–144 °C. Mixture of rotamers were observed. Major rotamer: ^{1}H NMR (400 MHz, MeOD) –δ 8.43 (s, 1H), 7.27 (d, *J* = 11.0 Hz, 6H), 6.98 (d, *J* = 8.6 Hz, 7H), 6.46 (d, *J* = 11.8 Hz, 1H). ^{13}C NMR (101 MHz, MeOD) –δ 174, 163, 136, 131, 129, 119, 115, 48, 47, 39. Minor rotamer: ^{1}H NMR (400 MHz, MeOD) –δ 7.98 (s, 1H), 7.02 (s, 1H), 6.98 6.86 (d, *J* = 11.0 Hz, 2H), 6.79 (d, *J* = 8.4 Hz, 1H). ^{13}C NMR (101 MHz, MeOD) δ 174, 160, 131, 130, 129, 118, 115, 48, 46, 39. m/z (ESI-HRMS) calculated for $C_9H_9NO_3$ $[M+H]^+$: 180.0582; found $[M+H]^+$: 180.0697.

1.3.19. *N*-(3-fluoro-phenyl) formamide

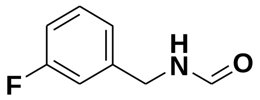

The experimental procedure described for the synthesis of *N*-phenyl formamide **1** was followed using 3-fluoro aniline (0.576 mL, 6 mmol) and triethyl orthoformate (24 mmol) and (1.2 g) of immobilized H_2SO_4 on silica gel. The mixture was subjected to purification and the eluted solvent (EtOAc/Pet (2:1)) was then evaporated under reduced pressure to afford 3-Fluorobenzylformamide **(19)** (0.56 g, 97%) mp 53–55 °C (lit. 53.9–54.6 °C)[6]. IR (NaCl) *v*(cm^{-1}) 3304, 3032, 2944, 1684, 1598, 1442, 1278, 1149, 861. Mixture of rotamers were observed. Major rotamer: ^{1}H NMR (400 MHz, $CDCl_3$) –δ 8.46 (s, 1H), 8.38 (s, 1H), 7.61 (s, 1H), 7.49 (d, *J* = 10.6 Hz, 1H), 7.35 – 7.22 (m, 1H), ^{13}C NMR (101 MHz, $CDCl_3$) δ 164, 159, 138, 131, 115, 112, 107. Minor rotamer: ^{1}H NMR (400 MHz, $CDCl_3$) –δ 8.71 (d, *J* = 11.2 Hz, 1H), 7.49 (d, *J* = 10.6 Hz, 1H), 7.19 (d, *J* = 8.3 Hz, 2H), 6.86 (dt, *J* = 18.1, 8.7 Hz, 3H). ^{13}C NMR (101 MHz, $CDCl_3$) δ 162, 138, 130, 114, 111, 106.

1.3.20. *N*-(3-bromo-phenyl) formamide

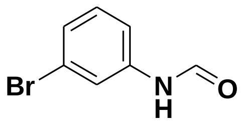

The experimental procedure described for the synthesis of *N*-phenyl formamide **1** was followed using 3-bromoaniline (0.6 mL, 6 mmol) and triethyl orthoformate (24 mmol) and (1.2 g) of immobilized H_2SO_4 on silica gel. The mixture was subjected to purification and the eluted solvent (EtOAc) was then evaporated under reduced pressure to afford a white solid *N*-(3-bromophenyl)formamide **(20)** (0.47 g, 78%) mp 100–103 °C. IR (NaCl) *v*(cm^{-1}) 3355, 2889, 1749, 1749, 1677, 1463, 1283. Mixture of rotamers were observed. Major rotamer: ^{1}H NMR (400 MHz, $CDCl_3$) −δ 9.07 (d, *J* = 11.0 Hz, 1H), 8.68 (d, *J* = 11.2 Hz, 1H), 7.79 (s, 2H), 7.44 (d, *J* = 8.1 Hz, 2H), 7.29 (d, *J* = 9.8 Hz, 2H), 7.15 (t, *J* = 8.1 Hz, 2H), 7.03 (d, *J* = 7.9 Hz, 1H), ^{13}C NMR (101 MHz, $CDCl_3$) δ 162, 138, 131, 128, 123, 118. Minor rotamer: ^{1}H NMR (400 MHz, $CDCl_3$) −δ 8.40 (s, 1H), 8.33 (s, 2H), 7.79 (s, 2H), 7.29 (d, *J* = 9.8 Hz, 2H), 7.25 – 7.17 (m, 3H), 7.03 (d, *J* = 7.9 Hz, 1H). ^{13}C NMR (101 MHz, $CDCl_3$) δ 159, 138, 130, 127, 121, 117.

1.3.21. *N*-(2-Iodo-phenyl) formamide

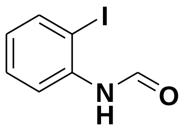

The experimental procedure described for the synthesis of *N*-phenyl formamide **1** was followed using 2-iodo aniline (0.7 g, 6 mmol) and triethyl orthoformate (24 mmol) and (1.2 g) of immobilized H_2SO_4 on silica gel. The mixture was subjected to purification and the eluted solvent (EtOAc/Pet (4:1)) was then evaporated under reduced pressure to afford a white solid *N*-(2-Iodo-phenyl) formamide **(21)** (0.66 g 94%) mp 116–118 °C (lit. 118 °C)[2]. IR (NaCl) *v*(cm^{-1}) 3313 2902 2647 1748 1658 1515 1434 1396 1201.5. ^{1}H NMR (400 MHz, $CDCl_3$)δ 8.55 (d, *J* = 7.8 Hz, 2H), 8.38 (s, 3H), 8.18 (d, *J* = 7.3 Hz, 3H), 7.73 (dd, *J* = 17.5, 7.0 Hz, 6H), 7.55 (d, *J* = 7.3 Hz, 12H), 7.48 (s, 2H), 7.18 (s, 1H), 6.85 (s, 2H), 6.79

(s, 3H), 6.69 (d, J = 7.2 Hz, 11H), 6.41 (s, 9H), 4.21 (s, 21H), 4.02 (s, 1H). ^{13}C NMR (101 MHz, $CDCl_3$) δ 162, 159, 146, 139, 139, 137, 129, 108.

1.3.22. *N*-(2-chlorophenyl)formamide

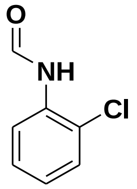

The experimental procedure described for the synthesis of *N*-phenyl formamide **1** was followed using 2-chlorooaniline (0.76 g, 6 mmol) and triethyl orthoformate (24 mmol) and (1.2 g) of immobilized H_2SO_4 on silica gel. The mixture was subjected to purification and the eluted solvent (EtOAc) was then evaporated under reduced pressure to afford a white solid *N*-(2-chlorophenyl)formamide **(22)** (0.69 g, 91%) mp 80–82 °C (lit. 81–82 °C)[3]. IR (NaCl) v(cm^{-1}) 3343, 3103, 3040, 2903, 1700, 1665, 1532, 1438, 1399, 1297, 1160, 1036. Mixture of rotamers were observed. ^{1}H NMR (400 MHz, $CDCl_3$) Major rotamer: −δ 8.50 (d, J = 1.4 Hz, 1H), 8.06 (s, 1H), 7.37 (dd, J = 8.0, 1.3 Hz, 1H), 7.24 (d, J = 1.1 Hz, 1H), 7.06 (td, J = 7.9, 1.4 Hz, 1H). ^{13}C NMR (101 MHz, $CDCl_3$) δ 159, 130, 129, 127, 125, 122, 119. Minor rotamer: ^{1}H NMR (400 MHz, $CDCl_3$) δ 8.70 (d, J = 11.1 Hz, 1H), 8.38 (d, J = 8.3 Hz, 1H), 7.42 (d, J = 8.0 Hz, 1H), 7.28 (d, J = 5.5 Hz, 1H), 7.13 (dt, J = 8.6, 4.4 Hz, 1H). ^{13}C NMR (101 MHz, $CDCl_3$) δ 162, 133, 128, 126, 124, 122.

1.3.23. *N*-(2-bromophenyl)formamide

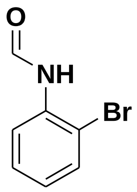

The experimental procedure described for the synthesis of *N*-phenyl formamide **1** was followed using 2-bromoaniline (1.03 g, 6 mmol) and triethyl orthoformate (24 mmol) and (1.2 g) of immobilized H_2SO_4 on silica gel. The mixture was subjected to purification and the eluted solvent (EtOAc) was then evaporated under reduced pressure to afford a white solid *N*-(2-bromophenyl)formamide **(23)** (0.87 g,

84%) mp 92–94 °C (lit. 93 °C)[7]. IR (NaCl) *v*(cm^{-1}) 3351, 2907, 1696, 1655, 1575, 1516, 1395, 1289, 1160, 1120. Mixture of rotamers were observed. ^{1}H NMR (400 MHz, $CDCl_3$) Major rotamer: −δ 8.39 (dd, *J* = 8.2, 1.3 Hz, 1H), 7.80 (s, 1H), 7.56 (dd, *J* = 8.1, 1.2 Hz, 1H), 7.02 (td, *J* = 7.9, 1.5 Hz, 1H). ^{13}C NMR (101 MHz, $CDCl_3$) −δ 159, 135, 132, 128, 126, 122, 113. Minor rotamer: ^{1}H NMR (400 MHz, $CDCl_3$) −δ 8.71 (d, *J* = 11.1 Hz, 1H), 8.39 (d, *J* = 9.5 Hz, 1H), 7.61 (d, *J* = 8.0 Hz, 1H), 7.33 (t, *J* = 7.8 Hz, 2H), 7.08 (t, *J* = 8.3 Hz, 1H). ^{13}C NMR (101 MHz, $CDCl_3$) δ 162, 135, 133, 129, 126, 119, 115.

1.3.24. *N*-(4-bromophenyl)formamide

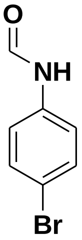

The experimental procedure described for the synthesis of *N*-phenyl formamide **1** was followed using 4-bromoaniline (0.8 mL, 6 mmol) and triethyl orthoformate (24 mmol) and (1.2 g) of immobilized H_2SO_4 on silica gel. The mixture was subjected to purification and the eluted solvent (EtOAc) was then evaporated under reduced pressure to afford a white solid *N*-(4-bromophenyl)formamide **(24)** (0.65g 81%) mp 115–117 °C (lit. 115–119 °C)[2];. IR (NaCl) *v*(cm^{-1}) 3355, 3185, 3114, 3052, 2864, 1747, 1651, 1586, 1395, 1307, 1250, 1067, 820. Mixture of rotamers were observed. ^{1}H NMR (400 MHz, $CDCl_3$) Major rotamer: −δ 8.36 (s, 1H), 8.06 (s, 1H), 7.83 (s, 1H), 7.46 (d, *J* = 8.8 Hz, 2H), 6.98 (d, *J* = 8.8 Hz, 2H). ^{13}C NMR (101 MHz, $CDCl_3$) −δ 171, 162, 159, 145, 135, 132, 121, 117, 116. Minor rotamer: ^{1}H NMR (400 MHz, $CDCl_3$) −δ 8.83 (d, *J* = 9.2 Hz, 1H), 8.63 (d, *J* = 11.3 Hz, 1H), 7.46 (d, *J* = 8.8 Hz, 2H), 7.21 (d, *J* = 11.9 Hz, 1H). ^{13}C NMR (101 MHz, $CDCl_3$) −δ 163, 145, 135, 131, 120, 118, 110.

1.3.25. *N*-(3,4-difluorophenyl) formamide

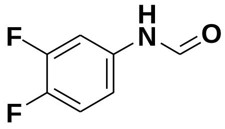

The experimental procedure described for the synthesis of *N*-phenylformamide **1** was followed using 3,4-difluoroaniline (0.77 g, 6 mmol) and triethyl orthoformate (24 mmol) and (1.2 g) of immobilized H_2SO_4 on silica gel. The mixture was subjected to purification and the eluted solvent (EtOAc) was then evaporated under reduced pressure to afford a white solid *N*-(3,4-difluorophenyl) formamide **(25)** (0.44 g, 56%). mp 165–170 °C. IR (NaCl) v(cm^{-1}) 3560, 2880, 1700, 1662, 1505, 1334, 1206. ^{1}H NMR (400 MHz, DMSO-d_6) −δ 7.97 (s, 1H), 7.16 – 7.04 (m, 2H), 6.93 (s, 2H), 6.75 (s, 2H). ^{13}C NMR (101 MHz, DMSO-d_6) δ 152, 149, 146, 118, 115, 108.

1.3.26. *N*-(3,4-dichlorophenyl)formamide

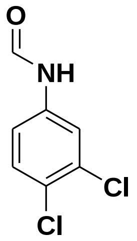

The experimental procedure described for the synthesis of *N*-phenyl formamide **1** was followed using 3,4-dichoro aniline (0.97 g, 6 mmol) and triethyl orthoformate (24 mmol) and (1.2 g) of immobilized H_2SO_4 on silica gel. The mixture was subjected to purification and the eluted solvent (EtOAc) was then evaporated under reduced pressure to afford a white solid *N*-(3,4-dichlorophenyl)formamide **(26)** (0.78g 81%) mp 105–107 °C (lit.107–108 °C)[8]. IR (NaCl) v(cm^{-1}) 3373, 3007, 2892, 1672, 1594, 1473, 1309, 1129, 812. Mixture of rotamers was observed. ^{1}H NMR (400 MHz, DMSO-d_6) Major rotamer: −δ 8.49 (d, J = 64.0 Hz, 1H), 8.29 (s, 1H), 7.49 – 7.30 (m, 2H). ^{13}C NMR (101 MHz, DMSO-d_6) δ 160, 138, 131, 126, 121, 119. Minor rotamer: ^{1}H NMR (400 MHz, DMSO-d_6) −δ 8.68 (d, J = 11.0 Hz, 1H), 7.88 (s, 1H), 8.08 (s, 1H), 7.06 (dd, J = 8.7, 2.2 Hz, 1H). ^{13}C NMR (101 MHz, DMSO-d_6) δ 162, 138, 132, 131, 127, 120, 118.

1.3.27. 3-Chlorobenzylformamide

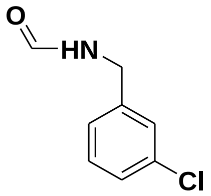

The experimental procedure described for the synthesis of *N*-phenyl formamide **1** was followed using 3-chlorobenzylamine (1.0 g, 6 mmol) and triethyl orthoformate (24 mmol) and (1.2 g) of immobilized H_2SO_4 on silica gel. The mixture was subjected to purification and the eluted solvent (EtOAc) was then evaporated under reduced pressure to afford a yellow viscous liquid 3-chlorobenzylformamide **(27)** (0.82 g, 82%). IR (NaCl) v(cm^{-1}) 3216, 2899, 2790, 2700, 2645, 1731, 1665, 1579, 1489, 1371, 1211, 1098, 773. ^{1}H NMR (400 MHz, DMSO-d_6) −δ 8.40 (s, 1H), 8.17 (s, 1H), 7.58 (s, 1H), 7.49 – 7.13 (m, 6H), 4.32 (d, J = 6.1 Hz, 1H), 4.05 (s, 1H). ^{13}C NMR (101 MHz, DMSO-d_6) δ 166, 161, 141, 137, 133, 130, 128, 128, 127, 127, 126, 41.

1.3.28. *N*-[(2-Methoxyphenyl)methyl]formamide

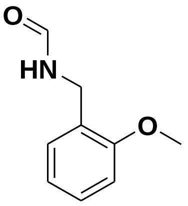

The experimental procedure described for the synthesis of *N*-phenyl formamide **1** was followed using 2-methoxybenzylamine (1.00 g, 6 mmol) and triethyl orthoformate (24 mmol) and (1.2 g) of immobilized H_2SO_4 on silica gel. The mixture was subjected to purification and the eluted solvent (EtOAc) was then evaporated under reduced pressure to afford a white solid *N*-(2-methoxybenzyl)formamide **(28)** (0.85 g, 85%) mp 78–80 °C. IR (NaCl) v(cm^{-1}) 3355, 2837, 2329, 2091, 1655, 1489, 1235, 1024. Mixture of rotamers were observed. ^{1}H NMR (400 MHz, $CDCl_3$) Major rotamer: −δ 8.14 (s, 1H), 8.11 (s, 1H), 7.25 (d, J = 7.4 Hz, 3H), 7.20 (d, J = 10.3 Hz, 2H), 6.95 – 6.83 (m, 6H), 4.45 (d, J = 6.0 Hz, 2H), 3.83 (s, 8H). ^{13}C NMR (101 MHz, $CDCl_3$) δ 161, 157, 129, 128, 125, 110, 55, 37. Minor rotamer: ^{1}H NMR (400 MHz, $CDCl_3$) −δ 8.85 (s, 1H), 7.28 (d, J = 2.4 Hz, 1H), 7.16 (d, J = 7.4 Hz, 1H), 7.03 – 6.94 (m, 1H), 6.75 (s, 1H), 4.31 (d, J = 4.7 Hz, 1H), 3.80 (s, 4H). ^{13}C NMR (101 MHz, $CDCl_3$) δ 165, 130, 129, 125, 120, 41.

1.3.29. *N*-(4-methoxybenzyl)formamide

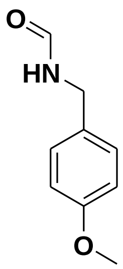

The experimental procedure described for the synthesis of *N*-phenyl formamide **1** was followed using 4-methoxybenzylamine (0.82 g, 6 mmol) and triethyl orthoformate (24 mmol) and (1.2 g) of immobilized H_2SO_4 on silica gel. The mixture was subjected to purification and the eluted solvent (EtOAc) was then evaporated under reduced pressure to afford a white solid *N*-(4-methoxybenzyl)formamide **(29)** (0.786 g, 96%). mp 77–79 °C (lit. 77 °C)[8]. IR (NaCl) ν(cm^{-1}) 3390, 2913, 2623, 2219, 1651, 1514, 1244. Mixture of rotamers was observed. ^{1}H NMR (400 MHz, $CDCl_3$) Major rotamer: −δ 8.14 (s, 1H), 7.17 (d, *J* = 8.7 Hz, 2H), 6.83 (d, *J* = 8.7 Hz, 2H), 6.64 (s, 1H), 4.34 (d, *J* = 5.9 Hz, 2H), 3.76 (s, 4H). ^{13}C NMR (101 MHz, $CDCl_3$) −δ 161, 129, 114, 55, 41. Minor rotamer: ^{1}H NMR (400 MHz, $CDCl_3$) −δ 8.07 (d, *J* = 11.9 Hz, 1H), 7.17 (d, *J* = 34.2 Hz, 1H), 6.87 (d, *J* = 8.3 Hz, 1H), 4.27 (d, *J* = 6.2 Hz, 1H), 3.78 (s, 2H). ^{13}C NMR (101 MHz, $CDCl_3$) −δ 159, 133, 130, 59, 45.

1.3.30. *N*-(4-methoxyphenyl)formamide

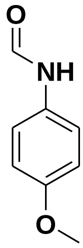

The experimental procedure described for the synthesis of *N*-phenyl formamide **1** was followed using p-anisidine (0.74 g, 6 mmol) and triethyl orthoformate (24 mmol) and (1.2 g) of immobilized H_2SO_4 on silica gel. The mixture was subjected to purification and the eluted solvent (EtOAc) was then evaporated under reduced pressure to afford a white solid *N*-(4-methoxyphenyl)formamide **(30)** (0.693 g, 94%) mp 77–79 °C (lit. 80–81 °C)[9]. IR (NaCl) ν(cm^{-1}) 3379, 3009, 2829, 1907, 1641, 1516, 1379, 1246, 1035. Mixture of rotamers were observed. ^{1}H NMR (400 MHz, $CDCl_3$) Major rotamer: −δ 8.57 (s, 1H), 8.19 (d, *J* = 34.9 Hz, 2H), 7.43 (d, *J* = 8.8 Hz, 1H), 6.81 (d, *J* = 8.8 Hz, 2H), 3.74 (s, 2H). ^{13}C NMR (101

MHz, $CDCl_3$) −δ 163, 159, 156, 129, 122, 114, 55. Minor rotamer: ^{1}H NMR (400 MHz, $CDCl_3$) −δ 9.06 (d, J = 9.5 Hz, 1H), 8.47 (d, J = 11.0 Hz, 1H), 7.01 (d, J = 8.7 Hz, 2H), 6.85 (d, J = 8.8 Hz, 2H), 3.77 (s, 3H). ^{13}C NMR (101 MHz, $CDCl_3$) −δ 171, 164, 157, 129, 121, 114, 60.

1.3.31. *N*-(3-cyanophenyl)formamide

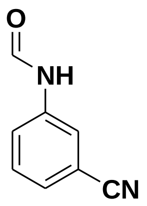

The experimental procedure described for the synthesis of *N*-phenyl formamide **1** was followed using 3-aminobenzo nitrile (0.71 g, 6 mmol) and triethyl orthoformate (24 mmol) and (1.2 g) of immobilized H_2SO_4 on silica gel. The mixture was subjected to purification and the eluted solvent (EtOAc) was then evaporated under reduced pressure to afford a white solid *N*-(3-cyanophenyl)formamide **(31)** (0.66 g, 93%) mp 115–117 °C. IR (NaCl) v(cm^{-1}) 3300, 3025, 2972, 2223, 1669, 1590, 1469, 1301. Mixture of rotamers were observed. ^{1}H NMR (400 MHz, DMSO-d_6) Major rotamer: - δ 10.32 (s, 1H), 10.17 (d, J = 10.6 Hz, 1H), 7.86 (s, 1H), 7.40 – 7.15 (m, 3H). ^{13}C NMR (101 MHz, DMSO-d_6) δ 160, 139, 130, 127, 123, 122, 118, 111. Minor rotamer: ^{1}H NMR (400 MHz, DMSO-d_6) −δ Major rotamer: 8.69 (d, J = 10.8 Hz, 1H), 8.15 (d, J = 1.5 Hz, 4H), 7.71 – 7.51 (m, 4H), 7.48 (s, 1H). ^{13}C NMR (101 MHz, DMSO-d_6) δ 162, 139, 130, 127, 122, 120, 112.

1.3.32. *N*-(2-formamidophenyl)formamide

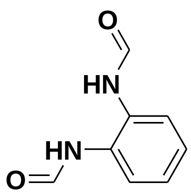

The experimental procedure described for the synthesis of *N*-phenyl formamide **1** was followed using o-phenelenediamine (0.65 g, 6 mmol) and triethyl orthoformate (24 mmol) and (1.2 g) of immobilized H_2SO_4 on silica gel. The mixture was subjected to purification and the eluted solvent (EtOAc/EtOH

(2:1)) was then evaporated under reduced pressure to afford *N*-(2-formamidophenyl)formamide **(32)** (0.64 g 96%). IR (NaCl) *v*(cm^{-1}) 3417, 2899, 1772, 1680, 1453, 1410, 1239. ^{1}H NMR (400 MHz, Acetone) - δ 8.69 (s, 1H), 8.44 (s, 1H), 7.84 (d, *J* = 66.2 Hz, 6H), 7.75 (s, 1H), 7.33 (d, *J* = 9.0 Hz, 2H). ^{13}C NMR (101 MHz, Acetone) δ 163, 159, 140, 134, 122, 114.

1.3.33. *N*-(4-acetyl phenyl) formamide

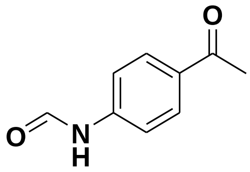

The experimental procedure described for the synthesis of *N*-phenyl formamide **1** was followed using 4-aceta aminophenone (0.8 g, 6 mmol) and triethyl orthoformate (24 mmol) and (1.2 g) of immobilized H_2SO_4 on silica gel. The mixture was subjected to purification and the eluted solvent (EtOAc) was then evaporated under reduced pressure to afford a yellow solid *N*-(4-acetyl phenyl) formamide **(33)** (0.76 g, 95%) 158–160 °C. IR (NaCl) *v*(cm^{-1}) 3293, 2884, 1669, 1662, 1582, 1529, 1268, 1058. Mixture of rotamers were observed. ^{1}H NMR (400 MHz, $CDCl_3$) Major rotamer: −δ 8.42 (s, 1H), 8.35 (s, 1H), 7.79 (d, *J* = 8.5 Hz, 3H), 6.64 (d, *J* = 8.6 Hz, 3H), 2.50 (s, 4H). ^{13}C NMR (101 MHz, $CDCl_3$) −δ 196, 159, 151, 141, 133, 130, 129, 119, 113, 25. Minor rotamer: ^{1}H NMR (400 MHz, $CDCl_3$) −δ 8.85 (d, *J* = 11.2 Hz, 1H), 8.59 (d, *J* = 9.4 Hz, 1H), 7.92 (d, *J* = 8.7 Hz, 5H), 7.68 (d, *J* = 8.7 Hz, 4H), 7.16 (d, *J* = 8.6 Hz, 2H), 2.57 (d, *J* = 3.2 Hz, 9H). ^{13}C NMR (101 MHz, $CDCl_3$) −δ 197, 161, 151, 141, 133, 130, 127, 117, 26.

1.3.34. *N*-(cyclohexyl)formamide

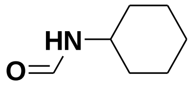

The experimental procedure described for the synthesis of *N*-phenyl formamide **1** was followed using cyclohexylamine (0.69 mL, 6 mmol) and triethyl orthoformate (24 mmol) and (1.2 g) of immobilized H_2SO_4 on silica gel. The mixture was subjected to purification and the eluted solvent (EtOAc/Pet ether

(3:1)) was then evaporated under reduced pressure to afford viscous colourless liquid *N*-(cyclohexyl)formamide **(34)** (0.60 g, 86%). IR (NaCl) *ν*(cm^{-1}) 3289, 2940, 2858, 2204, 1640, 1538, 1334. Mixture of rotamers were observed. ^{1}H NMR (400 MHz, $CDCl_3$) Major rotamer −δ 8.41 (s, 1H), 2.97 (s, 3H), 1.97 (d, *J* = 8.8 Hz, 6H), 1.72 (d, *J* = 11.8 Hz, 6H), 1.39 – 1.19 (m, 11H). ^{13}C NMR (101 MHz, $CDCl_3$) −δ 169, 77, 50, 31, 24. Minor rotamer ^{1}H NMR (400 MHz, $CDCl_3$) −δ 8.03 (s, 1H), 3.77 (s, 1H), 1.85 (d, *J* = 15.6 Hz, 2H), 1.58 (d, *J* = 12.2 Hz, 5H), 1.13 (dd, *J* = 25.0, 13.0 Hz, 11H). ^{13}C NMR (101 MHz, $CDCl_3$) −δ 161, 77, 48, 33, 25.

1.3.35. Ethyl 2-(formylamino)benzoate

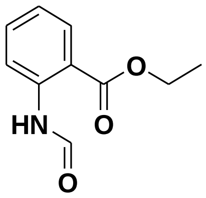

The experimental procedure described for the synthesis of *N*-phenyl formamide **1** was followed using ethyl 2-aminobenzoate (0.99 g, 6 mmol) and triethyl orthoformate (24 mmol) and (1.2 g) of immobilized H_2SO_4 on silica gel. The mixture was subjected to purification and the eluted solvent (EtOAc) was then evaporated under reduced pressure to afford a white solid ethyl 2-formamidobenzoate **(35)** (0.92g 93%) mp 58–60 °C (lit. 59 °C)[3]. IR (NaCl) *ν*(cm^{-1}) 3284, 2904, 1681, 1582, 1515, 1244. Mixture of rotamers were observed. ^{1}H NMR (400 MHz, $CDCl_3$) −δ 10.92 (s, 1H), 8.39 (s, 1H), 7.77 (d, *J* = 8.0 Hz, 1H), 7.13 (t, *J* = 8.3 Hz, 1H), 6.53 (t, *J* = 8.7 Hz, 2H), 5.46 (s, 2H), 4.24 – 4.16 (m, 2H), 1.26 (t, *J* = 7.2 Hz, 4H). ^{13}C NMR (101 MHz, $CDCl_3$) −δ 168, 159, 150, 140, 134, 131, 121, 116, 110, 60, 13. Minor rotamer: ^{1}H NMR (400 MHz, $CDCl_3$) δ 8.83 (d, *J* = 10.9 Hz, 1H), 8.61 (d, *J* = 8.4 Hz, 1H), 7.94 (d, *J* = 8.0 Hz, 1H), 7.42 (t, *J* = 7.7 Hz, 1H), 7.00 (t, *J* = 7.6 Hz, 1H), 4.35 – 4.21 (m, 8H), 1.30 (d, *J* = 7.1 Hz, 7H). ^{13}C NMR (101 MHz, $CDCl_3$) δ 168, 161, 146, 134, 130, 121, 115, 61, 29.

1.3.36. *N*-(naphthalen-1-yl)formamide

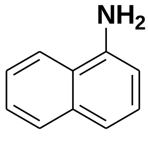

The experimental procedure described for the synthesis of *N*-phenyl formamide **1** was followed using 1-naphthylamine (0.86 g, 6 mmol) and triethyl orthoformate (24 mmol) and (1.2 g) of immobilized H_2SO_4 on silica gel. The mixture was subjected to purification and the eluted solvent (EtOAc) was then evaporated under reduced pressure to afford faint purple solid *N*-(naphthalen-1-yl)formamide **(36)** (0.84 g, 98%) mp 135–137 °C (lit. 137 °C)[3]. IR (NaCl) v(cm^{-1}) 3250, 3051, 2968, 1658, 1567, 1391, 1296. Mixture of rotamers were observed. ^{1}H NMR (400 MHz, $CDCl_3$) Major rotamer: −δ 8.30 (s, 1H), 8.17 (d, *J* = 7.0 Hz, 2H), 7.87 – 7.74 (m, 5H), 7.60 (d, *J* = 8.2 Hz, 2H), 7.50 – 7.35 (m, 10H), 6.75 (dd, *J* = 6.8, 1.5 Hz, 1H). ^{13}C NMR (101 MHz, $CDCl_3$) −δ 164, 142, 134, 128, 128, 127, 127, 126, 125, 124, 122, 121, 119, 110. Minor rotamer: ^{1}H NMR (400 MHz, $CDCl_3$) −δ 8.74 (s, 1H), 8.61 (d, *J* = 10.0 Hz, 1H), 8.55 (s, 1H), 7.86 (d, *J* = 8.4 Hz, 1H), 7.71 (t, *J* = 10.4 Hz, 2H), 7.27 (dt, *J* = 15.0, 7.7 Hz, 8H), 7.17 (d, *J* = 6.8 Hz, 6H). ^{13}C NMR (101 MHz, $CDCl_3$) −δ 159, 141, 134, 131, 128, 127, 126, 125, 124, 120, 119, 106.

1.3.37. *N*-(5-formamidonaphthalen-1-yl)formamide

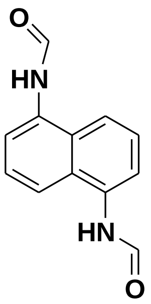

The experimental procedure described for the synthesis of *N*-phenyl formamide **1** was followed using 1,5-diaminonaphthalene (0.94 g, 6 mmol) and triethyl orthoformate (24 mmol) and (1.2 g) of immobilized H_2SO_4 on silica gel. The mixture was subjected to purification and the eluted solvent (EtOAc) was then evaporated under reduced pressure to afford a brown solid *N*-(5-formamidonaphthalen-1-yl)formamide **(37)** (0.60 g, 64%) mp 130–133 °C (lit.130–131 °C)[10]. IR (NaCl) v(cm^{-1}) 3224, 3005, 2903, 1641, 1528, 1395, 1262, 1215, 1149. Mixture of rotamers were observed. ^{1}H NMR (400 MHz, MeOD) Major rotamer: −δ 8.39 (d, *J* = 4.9 Hz, 1H), 7.84 (d, *J* = 8.7 Hz, 1H), 7.79 (d, *J* = 8.6 Hz, 1H), 7.33 – 7.26 (m, 4H), 6.76 (t, *J* = 7.8 Hz, 2H). ^{13}C NMR (101 MHz,

MeOD) −δ 161, 144, 131, 126, 123, 119, 109. Minor rotamer: ^{1}H NMR (400 MHz, MeOD) −δ 8.45 (s, 1H), 8.36 (s, 1H), 7.74 (d, *J* = 7.3 Hz, 1H), 7.10 (t, *J* = 7.8 Hz, 1H), 6.71 (d, *J* = 7.4 Hz, 1H). ^{13}C NMR (101 MHz, MeOD) −δ 165, 131, 127, 124, 120, 110.

1.3.38. *N*-(8-formamidonaphthalen-1-yl)formamide

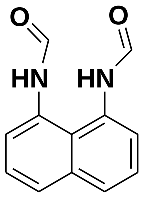

The experimental procedure described for the synthesis of *N*-phenyl formamide **1** was followed using 1,8-diaminonaphthalene (0.94 g, 6 mmol) and triethyl orthoformate (24 mmol) and (1.2 g) of immobilized H_2SO_4 on silica gel. The mixture was subjected to purification and the eluted solvent (EtOAc) was then evaporated under reduced pressure to afford a brown solid *N*-(8-formamidonaphthalen-1-yl)formamide **(38)** (0.86 g, 91%) mp 146–148 °C**.** IR (NaCl) *ν*(cm^{-1}) 3036, 2943, 2329, 1739, 1681, 1336, 1219. ^{1}H NMR (400 MHz, $CDCl_3$) −δ 7.46 (s, 1H), 7.44 (s, 1H), 7.09 – 7.00 (m, 5H), 6.46 (dd, *J* = 6.0, 2.2 Hz, 2H). ^{13}C NMR (101 MHz, $CDCl_3$) δ 159, 145, 137, 128, 120, 108. m/z (ESI-HRMS) calculated for $C_9H_9NO_3$ [M+Na]$^+$: 237.0639; found [M+Na]$^+$: 237.0698.

1.3.39. 2-Formamido anthraquinone

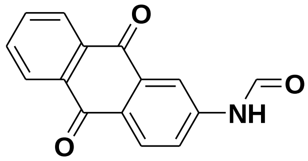

The experimental procedure described for the synthesis of *N*-phenyl formamide **1** was followed using 2-amino anthraquinone (1.34 g, 6 mmol) and triethyl orthoformate (24 mmol) and (1.2 g) of immobilized H_2SO_4 on silica gel. The mixture was subjected to purification and the eluted solvent (EtOAc/EtOH (1:2) was then evaporated under reduced pressure to afford an orange solid 2-formamido anthraquinone **(39)** (1.17 g, 98%) mp 306–308 °C. IR (NaCl) *ν*(cm^{-1}) 3249, 3108, 1706, 1667, 1589, 1538, 1287, 1240, 1177. ^{1}H NMR (400 MHz, $CDCl_3$) δ 8.52 (s, 1H), 8.35 (s, 1H), 8.34 (s, 1H), 8.33 – 8.31 (m, 3H), 8.30 (d, *J* = 1.7 Hz, 1H), 8.29 – 8.24 (m, 3H), 8.15 (d, *J* = 8.4 Hz, 2H), 7.81 (dd, *J* = 5.8,

3.3 Hz, 4H), 7.75 (td, J = 7.2, 1.6 Hz, 3H), 7.45 (d, J = 2.5 Hz, 2H). ^{13}C NMR (101 MHz, $CDCl_3$) δ 182.71, 181.29, 168.33, 135.43, 133.69, 132.33, 129.15, 127.07, 124.57.

1.3.40. *N*-(5-methylisoxazol-3-yl) formamide

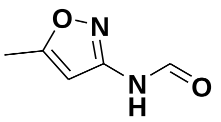

The experimental procedure described for the synthesis of *N*-phenylformamide **1** was followed using 3-amino-5-methylisoxazol (0.589 g, 6 mmol) and triethyl orthoformate (24 mmol) and (1.2 g) of immobilized H_2SO_4 on silica gel. The mixture was subjected to purification and the eluted solvent (Pet-ether/EtOAc (2:1)) was then evaporated under reduced pressure to afford a yellow solid *N*-(5-methylisoxazol-3-yl) formamide **(40)** (0.56 g, 95%) mp 120–122 °C. IR (NaCl) ν(cm^{-1}) 3213, 3151, 2918, 1686, 1548, 1396, 1249. Mixture of rotamers were observed. ^{1}H NMR (400 MHz, $CDCl_3$) Major rotamer: −δ 8.40 (s, 1H), 6.70 (s, 1H), 5.53 (s, 3H), 3.70 (s, 5H), 2.39 (s, 4H). ^{13}C NMR (101 MHz, $CDCl_3$) −δ 169, 164, 159, 96, 94, 13. Minor Rotamer: ^{1}H NMR (400 MHz, $CDCl_3$) −δ 9.69 (s, 2H), 8.80 (d, J = 11.0 Hz, 1H), 5.91 (s, 1H), 2.28 (s, 2H). ^{13}C NMR (101 MHz, $CDCl_3$) −δ 170, 162, 157, 97, 93, 12.

1.3.41. *N*-(Furan-2yl)methyl)formamide

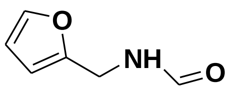

The experimental procedure described for the synthesis of *N*-phenyl formamide **1** was followed using furfurylamine (0.53 mL, 6 mmol) and triethyl orthoformate (24 mmol) and (1.2 g) of immobilized H_2SO_4 on silica gel. The mixture was subjected to purification and the eluted solvent (EtOAc) was then evaporated under reduced pressure to afford a brown liquid *N*-((furan-2-yl)methyl) formamide **(41)** (0.521 g, 98%). IR (NaCl) ν(cm^{-1}) 3375, 3044, 2919, 2853, 2333, 2083, 1661, 1504, 1383, 1199, 1012. Mixture of rotamers is observed. ^{1}H NMR (400 MHz, $CDCl_3$) Major rotamer: −δ 7.47 (s, 1H), 7.30 (d, J = 1.7 Hz, 1H), 7.26 (s, 1H), 6.19 (dd, J = 35.5, 2.0 Hz, 1H), 4.35 – 4.29 (m, 1H). ^{13}C NMR (101 MHz,

$CDCl_3$) −δ 161.56, 150.72, 142.28, 110.43, 34.89. Minor rotamer: ^{1}H NMR (400 MHz, $CDCl_3$) −δ 8.03 (s, 4H), 7.98 (d, *J* = 3.9 Hz, 1H), 7.00 (s, 1H), 6.26 (d, *J* = 1.7 Hz, 1H), 6.06 – 5.98 (m, 1H), 5.56 (d, *J* = 3.7 Hz, 1H), 4.28 – 4.22 (m, 1H). ^{13}C NMR (101 MHz, $CDCl_3$) −δ 165, 142, 128, 107, 38.

1.3.42. 2-Formamido-3-nitropyridine

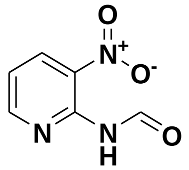

The experimental procedure described for the synthesis of *N*-Phenyl formamide **1** was followed using 2-amino-3-nitropyridine (0.84 g, 6 mmol) and triethyl orthoformate (24 mmol) or and (1.2 g) of immobilized H_2SO_4 on silica gel. The mixture was subjected to purification and the eluted solvent (EtOAc/Pet-Ether (4:1)) was then evaporated under reduced pressure to afford a yellow solid 2-Formamido-3-nitropyridine **(42)** (0.77 g, 92%). mp 140–141 °C. IR (NaCl) *v*(cm^{-1}) 3460, 3089, 2913, 2846, 2328, 1643, 1567, 1505, 1429, 1206. ^{1}H NMR (400 MHz, $CDCl_3$) δ 8.42 (dd, *J* = 8.3, 1.6 Hz, 1H), 8.35 (dd, *J* = 4.5, 1.6 Hz, 1H), 6.74 (dd, *J* = 8.3, 4.5 Hz, 1H), 1.75 (s, 1H). ^{13}C NMR (101 MHz, $CDCl_3$) δ 156, 153, 135, 128, 113.

1.3.43. *N*-(thiazol-2-yl)formamide

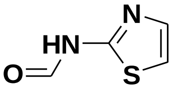

The experimental procedure described for the synthesis of *N*-Phenyl formamide **1** was followed using 2-aminothiazole (0.6 g, 6 mmol) and triethyl orthoformate (24 mmol) and (1.2 g) of immobilized H_2SO_4 on silica gel. The mixture was subjected to purification and the eluted solvent (EtOAc/EtOH (2:1)) was then evaporated under reduced pressure to afford a white solid *N*-(thiazol-2-yl)formamide **(43)** (0.40 g, 67%) mp 155–157 °C (lit.156–161 °C)[11]. IR (NaCl) *v*(cm^{-1}) 3400, 3267, 3087, 3014, 2809, 1700, 1645, 1520, 1172. ^{1}H NMR (400 MHz, Chloroform-*d*) −δ 8.62 (s, 1H), 7.47 (d, *J* = 3.6 Hz, 1H), 7.05 (d, *J* = 3.6 Hz, 1H), 6.54 (s, NH). ^{13}C NMR (101 MHz, $CDCl_3$) −δ 158, 146, 137, 114.

1.3.44. 2-Formamido benzyimidazole

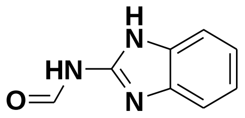

The experimental procedure described for the synthesis of *N*-phenyl formamide **1** was followed using 2-amino benzyimidazole (0.8 g, 6 mmol) and triethyl orthoformate (24 mmol) and (1.2 g) of immobilized H_2SO_4 on silica gel. The mixture was subjected to purification and the eluted solvent (EtOAc/EtOH1:2) was then evaporated under reduced pressure to afford a white solid 2-Formamido benzyimidazole **(44)** (0.63g 79%) mp 165 – 167 °C. IR (NaCl) v(cm^{-1}) 3567, 3065, 1694, 1538, 1475, 1377, 1326, 1193, 1036. ^{1}H NMR (400 MHz, DMSO-d_6) −δ 8.36 (s, 1H), 7.13 (dd, J = 5.8, 3.2 Hz, 5H), 6.90 (dd, J = 5.8, 3.2 Hz, 5H), 6.71 (s, 5H). ^{13}C NMR (101 MHz, DMSO-d_6) −δ 172, 165, 154, 136, 119, 110.

1.3.45. 4-formamido-antipyrine

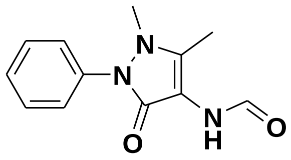

The experimental procedure described for the synthesis of *N*-phenyl formamide **1** was followed using 4-amino antipyrine (1.22 g, 6 mmol) and triethyl orthoformate (24 mmol) and (1.2 g) of immobilized H_2SO_4 on silica gel. The mixture was subjected to purification and the eluted solvent (EtOAc/EtOH (1:1)) was then evaporated under reduced pressure to afford a yellow solid 4-formamido-antipyrine **(45)** (0.93 g, 76%) mp 136–137 °C. IR (NaCl) v(cm^{-1}) 3359, 3157, 3054, 2873, 2501, 2157, 1687, 1640, 1303. ^{1}H NMR (400 MHz, $CDCl_3$) −δ 8.91 (s, 1H), 8.61 (s, 1H), 7.98 (d, J = 51.9 Hz, 3H), 7.68 – 6.98 (m, 13H), 2.97 (s, 5H), 2.05 (s, 5H). ^{13}C NMR (101 MHz, $CDCl_3$) −δ 166, 163, 162, 149, 133, 129, 125, 104, 77, 34, 11.

1.3.46. *N*-(Pyridine -2-yl) formamide

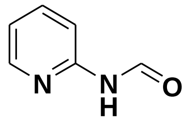

The experimental procedure described for the synthesis of *N*-phenyl formamide **1** was followed using 2-aminopyridine (0.56 g, 6 mmol) and triethyl orthoformate (24 mmol) and (1.2 g) of immobilized H_2SO_4 on silica gel. The mixture was subjected to purification and the eluted solvent (EtOAc/EtOH (1:1)) was then evaporated under reduced pressure to afford a white solid *N*-(Pyridine -2-yl) formamide **(46)** (0.44 g, 79%) mp 72–73 °C (lit. 71 °C)[9]. IR (NaCl) *v*(cm^{-1}) 3384, 3271, 3048, 2907, 2731, 2305, 2071, 1758, 1642, 1516, 1438, 1282. Mixture of rotamers were observed. ^{1}H NMR (400 MHz, MeOD) Major rotamer: −δ 9.31 (s, 1H), 8.29 (d, *J* = 3.8 Hz, 1H), 8.11 (d, *J* = 8.3 Hz, 1H), 7.81 – 7.66 (m, 2H). ^{13}C NMR (101 MHz, MeOD) −δ 164, 152, 149, 139, 121, 115. Minor rotamer: ^{1}H NMR (400 MHz, MeOD) −δ 8.39 (s, 1H), 8.25 (d, *J* = 4.0 Hz, 1H), 7.11 (dd, *J* = 12.3, 7.2 Hz, 2H), 6.93 (d, *J* = 8.1 Hz, 1H). ^{13}C NMR (101 MHz, MeOD) −δ 161, 151, 148, 139, 120, 112.

1.3.47. *N*-(Pyridine -4-yl) formamide

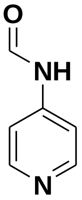

The experimental procedure described for the synthesis of *N*-phenyl formamide **1** was followed using 2-aminopyridine (0.56 g, 6 mmol) and triethyl orthoformate (24 mmol) and (1.2 g) of immobilized H_2SO_4 on silica gel. The mixture was subjected to purification and the eluted solvent (EtOAc/EtOH (1:1)) was then evaporated under reduced pressure to afford a yellow liquid *N*-(Pyridine-4-yl) formamide **(47)** (0.40 g, 71%). IR (NaCl) *v*(cm^{-1}) 3387, 3101, 1694, 1628, 1526, 1338, 1201. ^{1}H NMR (400 MHz, $CDCl_3$) −δ 10.73 (s, 1H), 10.11 (d, *J* = 7.3 Hz, 2H), 9.48 (s, 1H), 8.92 (d, *J* = 7.3 Hz, 2H). ^{13}C NMR (101 MHz, $CDCl_3$) −δ 173, 161, 141, 111.

1.3.48. *N*-((pyridine-2-yl)methyl)formamide

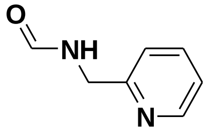

The experimental procedure described for the synthesis of *N*-phenyl formamide **1** was followed using 2-picolyl amine (0.65 mL, 6 mmol) and triethyl orthoformate (24 mmol) and (1.2 g) of immobilized H_2SO_4 on silica gel. The mixture was subjected to purification and the eluted solvent (EtOAc) was then evaporated under reduced pressure to afford a yellow oil *N*-((pyridine-2-yl)methyl)formamide **(48)** (0.61 g, 94%). IR (NaCl) v(cm^{-1}) 3263, 3044, 2927, 2853, 2333, 2094, 1668, 1594, 1532, 1379, 1231, 1153. Mixture of rotamers were observed. ^{1}H NMR (400 MHz, $CDCl_3$) Major rotamer: −δ 10.10 (d, *J* = 7.0 Hz, 1H), 8.46 (d, *J* = 4.8 Hz, 1H), 8.12 (s, 1H), 7.75 (td, *J* = 7.7, 1.5 Hz, 1H), 7.39 (d, *J* = 7.9 Hz, 1H), 7.29 – 7.25 (m, 1H), 4.55 (d, *J* = 6.0 Hz, 1H). ^{13}C NMR (101 MHz, $CDCl_3$) −δ 164.64, 162.24, 155.60, 147.55, 139.11, 123.38, 42.21. Minor rotamer: ^{1}H NMR (400 MHz, $CDCl_3$) −δ 8.50 (d, *J* = 5.3 Hz, 1H), 8.20 (s, 1H), 7.85 (s, 1H), 7.72 – 7.63 (m, 1H), 7.42 (d, *J* = 7.6 Hz, 1H), 7.24 – 7.18 (m, 1H), 4.76 (d, *J* = 6.1 Hz, 1H). ^{13}C NMR (101 MHz, $CDCl_3$) −δ 166.44, 156.66, 148.60, 137.79, 126.52, 121.95, 46.41.

1.3.49. 2-Formamido benzothiazole

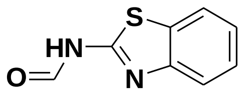

The experimental procedure described for the synthesis of *N*-phenyl formamide **1** was followed using 2-amino benzothiazole (0.90 g, 6 mmol) and triethyl orthoformate (24 mmol) and (1.2 g) of immobilized H_2SO_4 on silica gel. The mixture was subjected to purification and the eluted solvent (EtOAc) was then evaporated under reduced pressure to afford a white solid 2-formamido benzothiazole **(49)** (0.79 g, 87%) mp 74–76 °C. IR (NaCl) v(cm^{-1}) 3351, 3179, 2713, 2282, 1690, 1620, 1518, 1444. ^{1}H NMR (400 MHz, DMSO-d_6) −δ 8.13 (s, 1H), 7.58 (d, *J* = 7.8 Hz, 1H), 7.35 (d, *J* = 8.0 Hz, 1H), 7.19 (t, *J* = 7.7 Hz, 1H), 6.99 (t, *J* = 7.6 Hz, 1H), 6.19 (s, 4H). ^{13}C NMR (101 MHz, DMSO-d_6) −δ 168, 164, 152, 130, 126, 122, 121, 118.

1.3.50. *N*-methyl-*N*-phenylformamide

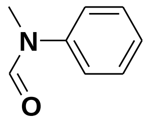

The experimental procedure described for the synthesis of *N*-phenyl formamide **1** was followed using N-methylaniline (0.65 mL, 6 mmol) and triethyl orthoformate (24 mmol) and (1.2 g) of immobilized H_2SO_4 on silica gel. The mixture was subjected to purification and the eluted solvent (EtOAc) was then evaporated under reduced pressure to afford *N*-methyl-*N*-phenylformamide **(50)** (0.48 g, 73%) mp 123–125 °C (lit. 124–125 °C) [12]. Mixture of rotamers were observed. ^{1}H NMR (400 MHz, $CDCl_3$) Major rotamer: −δ 8.39 (s, 1H), 7.32 (t, *J* = 7.8 Hz, 2H), 7.13 – 7.04 (m, 6H), 6.53 (dd, *J* = 8.6, 1.0 Hz, 3H), 2.74 (s, 5H). ^{13}C NMR (101 MHz, $CDCl_3$) −δ 162, 149, 128, 122, 112, 31. Minor rotamer: ^{1}H NMR (400 MHz, $CDCl_3$) −δ 8.26 (s, 1H), 7.23 – 7.16 (m, 1H), 6.66 – 6.59 (m, 2H), 3.23 (s, 3H). ^{13}C NMR (101 MHz, $CDCl_3$) −δ 162, 142, 129, 126, 117, 32.

1.3.51. *N*-[4-chloro-2-(2-fluorobenzoyl)phenyl]formamide

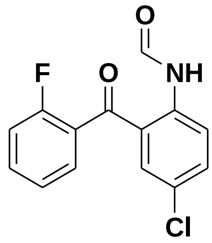

The experimental procedure described for the synthesis of *N*-phenyl formamide **1** was followed using 2-amino-5-chloro-2-fluoro benzophenone (1.5 g, 6 mmol) and triethyl orthoformate (24 mmol) and (1.2 g) of immobilized H_2SO_4 on silica gel. The mixture was subjected to purification and the eluted solvent (EtOAc) was then evaporated under reduced pressure to afford a yellow solid *N*-[4-chloro-2-(2-fluorobenzoyl)phenyl]formamide **(51)** (1.22 g, 81%) mp 79–81 °C. IR (NaCl) *v*(cm^{-1}) 3446 3341.4 2923 1640.5 1477.5 1215.8 1154. Mixture of rotamers were observed. ^{1}H NMR (400 MHz, $CDCl_3$) Major rotamer: −δ 8.64 (d, *J* = 9.0 Hz, 1H), 8.53 (s, 1H), 8.46 (d, *J* = 14.5 Hz, 1H), 7.96 (s, 1H), 7.58 (d, *J* = 6.6 Hz, 1H), 7.35 (td, *J* = 7.6, 1.6 Hz, 5H), 7.22 – 7.20 (m, 4H), 7.16 (t, *J* = 7.5 Hz, 6H), 6.90 (d, *J* = 9.4 Hz, 5H). 4.04 (q, *J* = 7.1 Hz, 1H), 3.89 (q, *J* = 7.1 Hz, 2H), 1.96 (s, 2H), 1.19 (dt, *J* = 18.1, 7.1 Hz, 3H). ^{13}C NMR (101 MHz, $CDCl_3$) −δ 194, 170, 160, 158, 145, 134, 132, 130, 126, 124, 120,

116, 64, 60, 21, 14. Minor rotamer:^{1}H NMR (400 MHz, $CDCl_3$) −δ 10.97 (s, 1H), 8.64 (d, *J* = 9.0 Hz, 1H), 8.11 (d, *J* = 8.6 Hz, 2H), 7.58 (d, *J* = 6.6 Hz, 4H), 7.46 – 7.38 (m, 7H), 7.22 – 7.19 (m, 10H), 7.07 (t, *J* = 9.1 Hz, 17H), 6.84 (d, *J* = 1.8 Hz, 3H) 4.14 (q, *J* = 7.1 Hz, 1H), 3.59 (q, *J* = 7.0 Hz, 1H), 1.93 (s, 1H), 1.14 – 1.06 (m, 5H). ^{13}C NMR (101 MHz, $CDCl_3$) −δ 188, 174, 161, 159, 151, 133, 131, 127, 123, 121, 118, 59, 20, 14.

1.3.52. Piperidine-1-carbaldehde

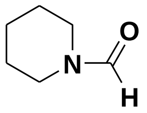

The experimental procedure described for the synthesis of *N*-phenyl formamide **1** was followed using piperidine (0.79 mL 6 mmol) and triethyl orthoformate (24 mmol) and (1.2 g) of immobilized H_2SO_4 on silica gel. The mixture was subjected to purification and the eluted solvent (EtOAc) was then evaporated under reduced pressure to afford a yellow oil Piperidine-1-carbaldehde **(52)** (0.67 mL, 85%). IR (NaCl) *v*(cm^{-1}) 3371, 2944, 2772, 2540, 1726, 1694, 1585, 1338. ^{1}H NMR (400 MHz, D_2O) −δ 8.38 (s, 1H), 3.15 – 2.98 (m, 4H), 1.69 (dt, *J* = 11.4, 5.8 Hz, 4H), 1.57 (dd, *J* = 11.0, 5.7 Hz, 2H). ^{13}C NMR (101 MHz, D_2O) −δ 170, 44, 22, 21.

1.3.53. 3-Formamido-1,2,4-triazole-5-thiol

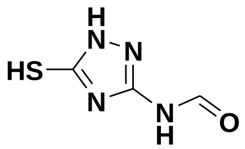

The experimental procedure described for the synthesis of *N*-phenyl formamide **1** was followed using piperidine (0.67 g, 6 mmol) and triethyl orthoformate (24 mmol) and (1.2 g) of immobilized H_2SO_4 on silica gel. The mixture was subjected to purification and the eluted solvent (EtOAc) was then evaporated under reduced pressure to afford a white solid 3-formamido-1,2,4-triazole **(53)** (0.60 g 90%) mp 198–200 °C (lit. 201–202 °C)[3]. IR (NaCl) *v*(cm^{-1}) 3398, 3316, 3191, 3050, 2725, 1636, 1593, 1209. Mixture of two rotamers were observed. ^{1}H NMR (400 MHz, DMSO-d_6) Major rotamer: −δ 8.93 (s,

1H), 7.95 (s, 1H), 7.47 (s, 2H), 5.84 (s, 4H). ^{13}C NMR (101 MHz, DMSO-d_6) −δ 163, 160, 144. Minor rotamer: ^{1}H NMR (400 MHz, DMSO-d_6) −δ 11.04 (s, 1H), 8.44 (s, 1H), 8.34 (s, 1H), 7.47 (s, 2H). ^{13}C NMR (101 MHz, DMSO) −δ 166, 156, 148. m/z (ESI-HRMS) calculated for $C_9H_9NO_3$ $[M+H]^+$: 145.0106; found $[M+H]^+$: 145.0218.

1.3.54. *N*-[2-(3,4-dihydroxyphenyl)-2-hydroxyethyl]-*N*-methylformamide

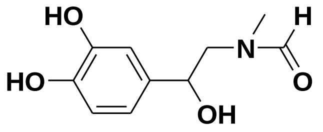

The experimental procedure described for the synthesis of *N*-phenyl formamide **1** was followed using (−)-Epinephrine (1.09 g, 6 mmol) and triethyl orthoformate (24 mmol) and (1.2 g) of immobilized H_2SO_4 on silica gel. The mixture was subjected to purification and the eluted solvent (EtOAc) was then evaporated under reduced pressure to afford a brown viscous liquid *N*-[2-(3,4-dihydroxyphenyl)-2-hydroxyethyl]-*N*-methylformamide **(54)** (0.816 g, 75%). IR (NaCl) $v(cm^{-1})$ 3245, 3003, 2787, 2137, 1734, 1636, 1538, 1334. ^{1}H NMR (400 MHz, D_2O) δ 8.17 (s, 1H), 6.84 – 6.62 (m, 2H), 6.59 (d, J = 7.9 Hz, 1H), 6.50 (d, J = 7.8 Hz, 1H), 4.71 (s, 9H), 2.99 (d, J = 8.1 Hz, 1H), 2.60 (s, 1H), 2.55 (s, 2H). ^{13}C NMR (101 MHz, D_2O) δ 168, 144, 132, 118, 116, 113, 68, 54, 32.

1.3.55. *N*-(2-mercaptoethyl)formamide

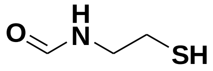

The experimental procedure described for the synthesis of *N*-phenyl formamide **1** was followed using cysteamine (0.46 g, 6 mmol) and triethyl orthoformate (24 mmol) and (1.2 g) of immobilized H_2SO_4 on silica gel. The mixture was subjected to purification and the eluted solvent (EtOAc) was then evaporated under reduced pressure to afford a yellowish liquid *N*-(2-mercaptoethyl)formamide **(55)** (0.39 g 85%). IR (NaCl) $v(cm^{-1}\ )$ 3363, 3148, 2783, 1671, 1593, 1393. ^{1}H NMR (400 MHz, D_2O) −δ 8.27 (s, 1H), 7.96 (s, 1H), 4.71 (s, 4H), 3.07 (t, J = 6.6 Hz, 1H), 2.71 (t, J = 6.6 Hz, 1H). ^{13}C NMR (101 MHz, D_2O) −δ 168, 164, 41, 21.

1.3.56. *N*-formylpyridine-3-carboxamide

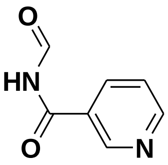

The experimental procedure described for the synthesis of *N*-phenyl formamide **1** was followed using nicotinamide (0.73 g, 6 mmol) and triethyl orthoformate (24 mmol) and (1.2 g) of immobilized H_2SO_4 on silica gel. The mixture was subjected to purification and the eluted solvent (EtOAc) was then evaporated under reduced pressure to afford a white solid *N*-formylpyridine-3-carboxamide **(56)** (0.68 g, 93%) mp 98–100 °C. IR (NaCl) ν(cm^{-1}) 3245, 3003, 2787, 2137, 1734, 1636, 1538, 1334. ^{1}H NMR (400 MHz, D_2O) −δ 8.65 (d, *J* = 1.9 Hz, 1H), 8.47 (d, *J* = 6.4 Hz, 1H), 8.27 (s, 1H), 8.02 (dd, *J* = 9.9, 1.8 Hz, 1H), 7.39 (dd, *J* = 8.0, 5.1 Hz, 1H), 4.70 (s, 4H). ^{13}C NMR (101 MHz, D_2O) −δ 169, 151, 146, 137, 128, 124.

2. NMR spectral data of synthesised compounds

2.1. *N*-Phenylformamide

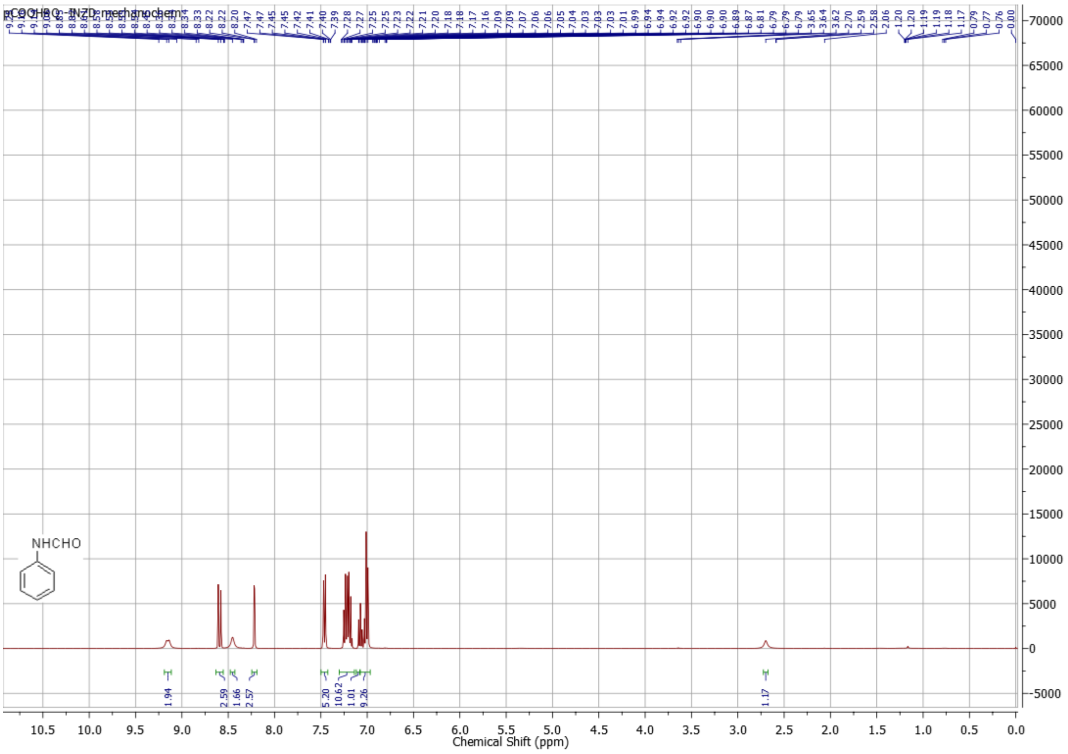

Figure S1.1 ^{1}H NMR (400 MHz, $CDCl_3$) spectrum

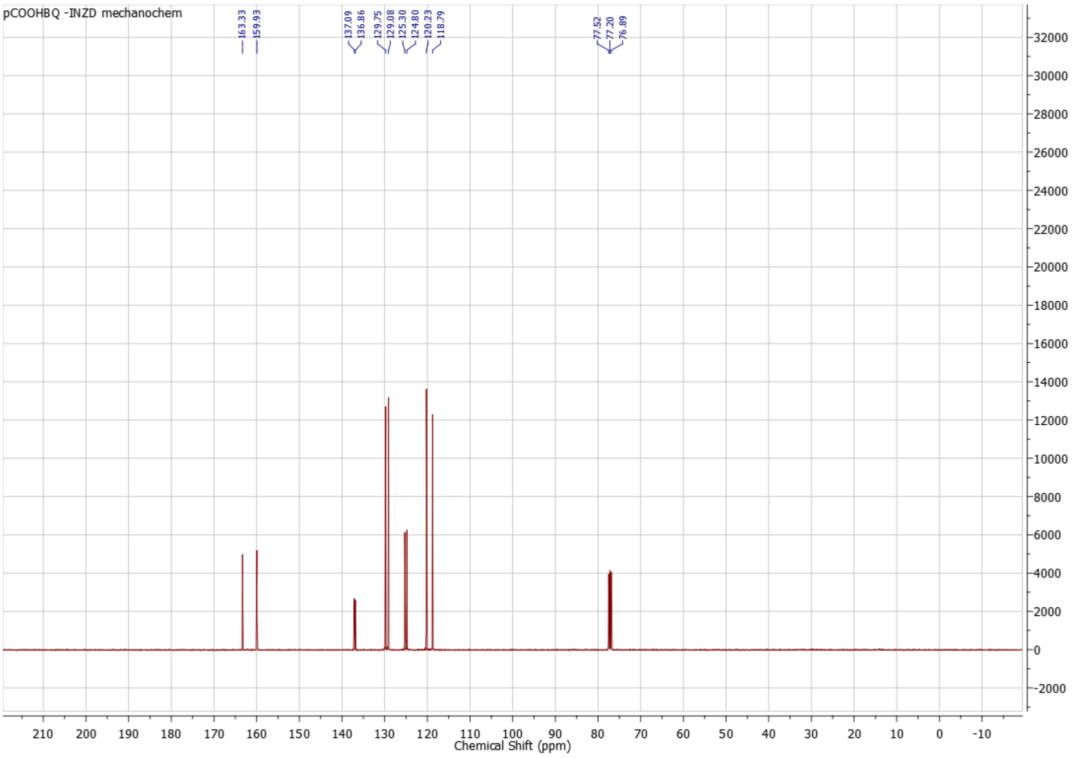

Figure S1.2. ^{13}C NMR (101 MHz, $CDCl_3$) spectrum

2.2. *N*-isobutylformamide

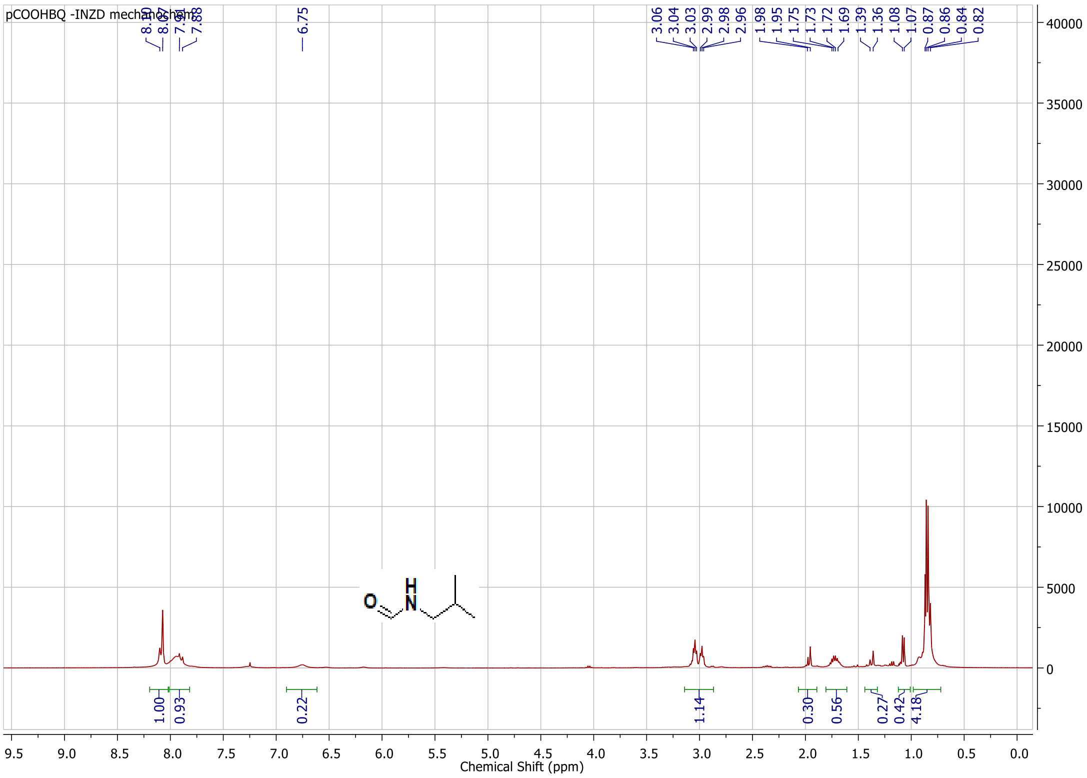

Figure S2.1 ^{1}H NMR (400 MHz, $CDCl_3$) spectrum

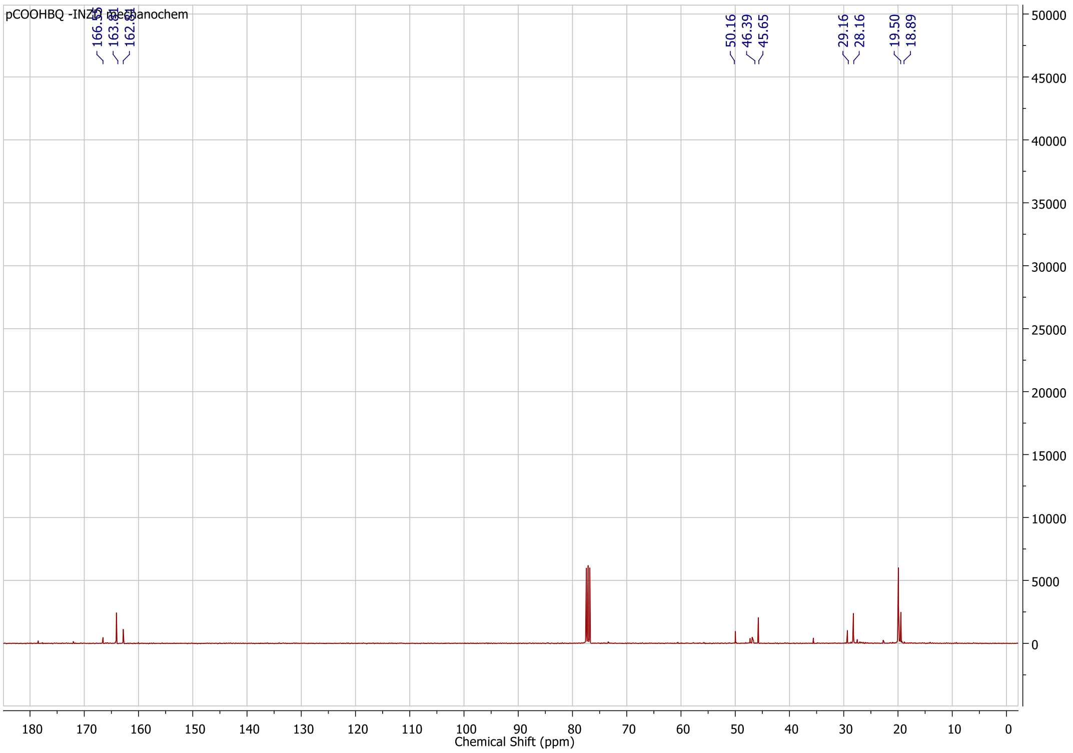

Figure S2.2. ^{13}C NMR (101 MHz, $CDCl_3$) spectrum

2.3. *N*-(prop-2-ynyl)formamide

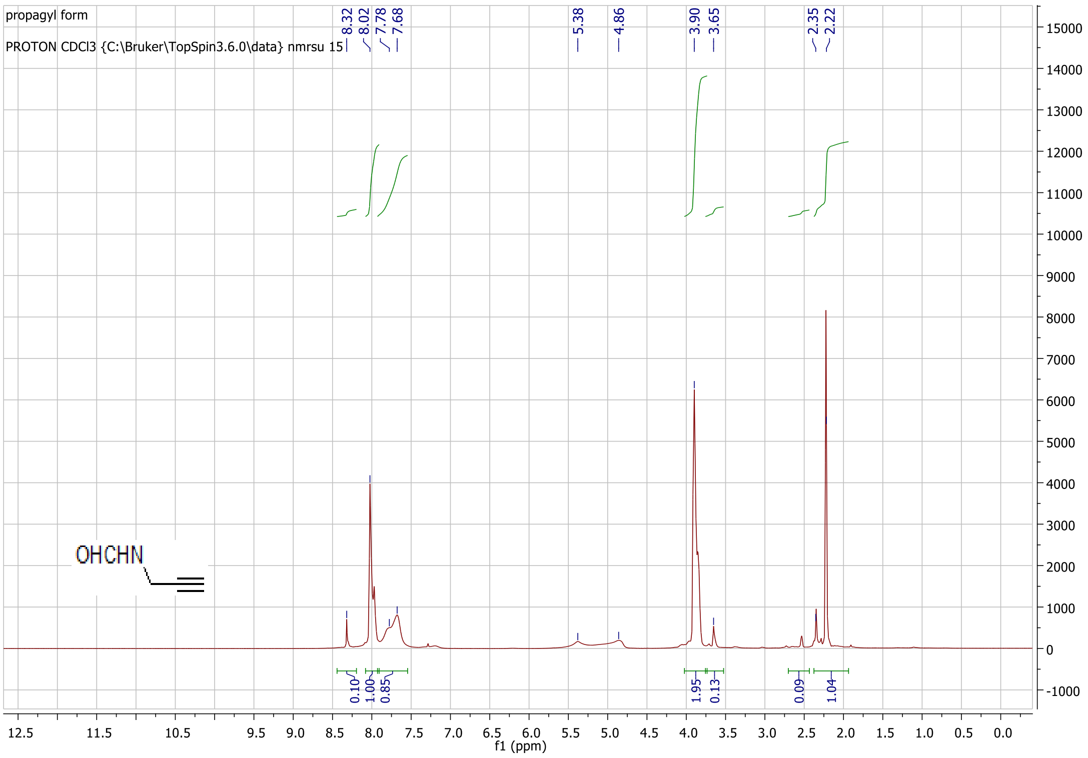

Figure S3.1. ^{1}H NMR (400 MHz, $CDCl_3$) spectrum

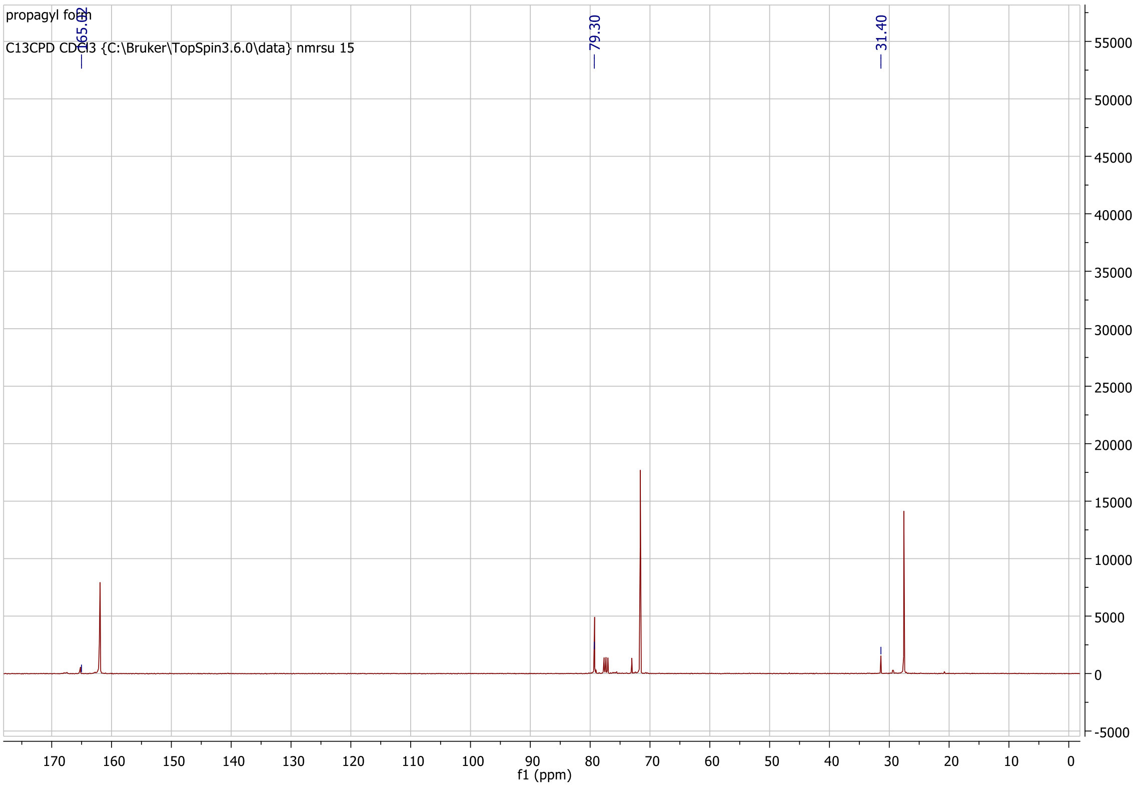

Figure S3.2. ^{13}C NMR (101 MHz, $CDCl_3$) spectrum

2.4. *N*-p-tolylformamide

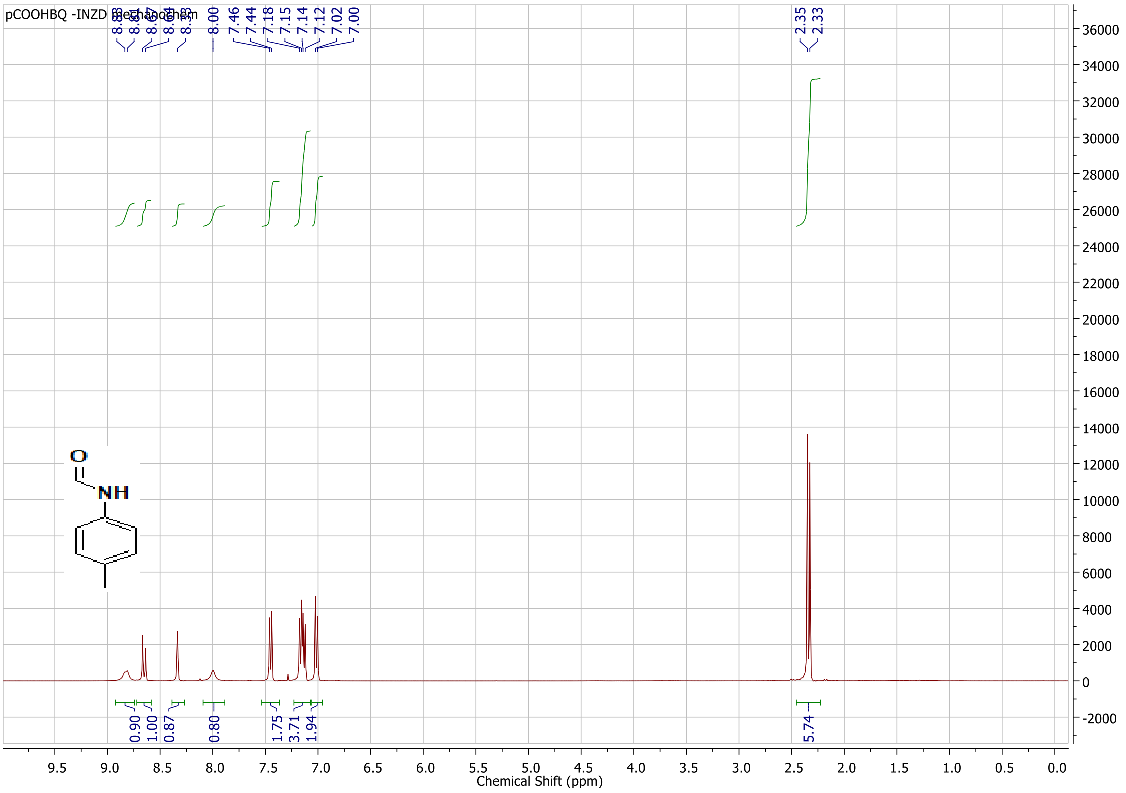

Figure S4.1. ^{1}H NMR (400 MHz, $CDCl_3$) spectrum

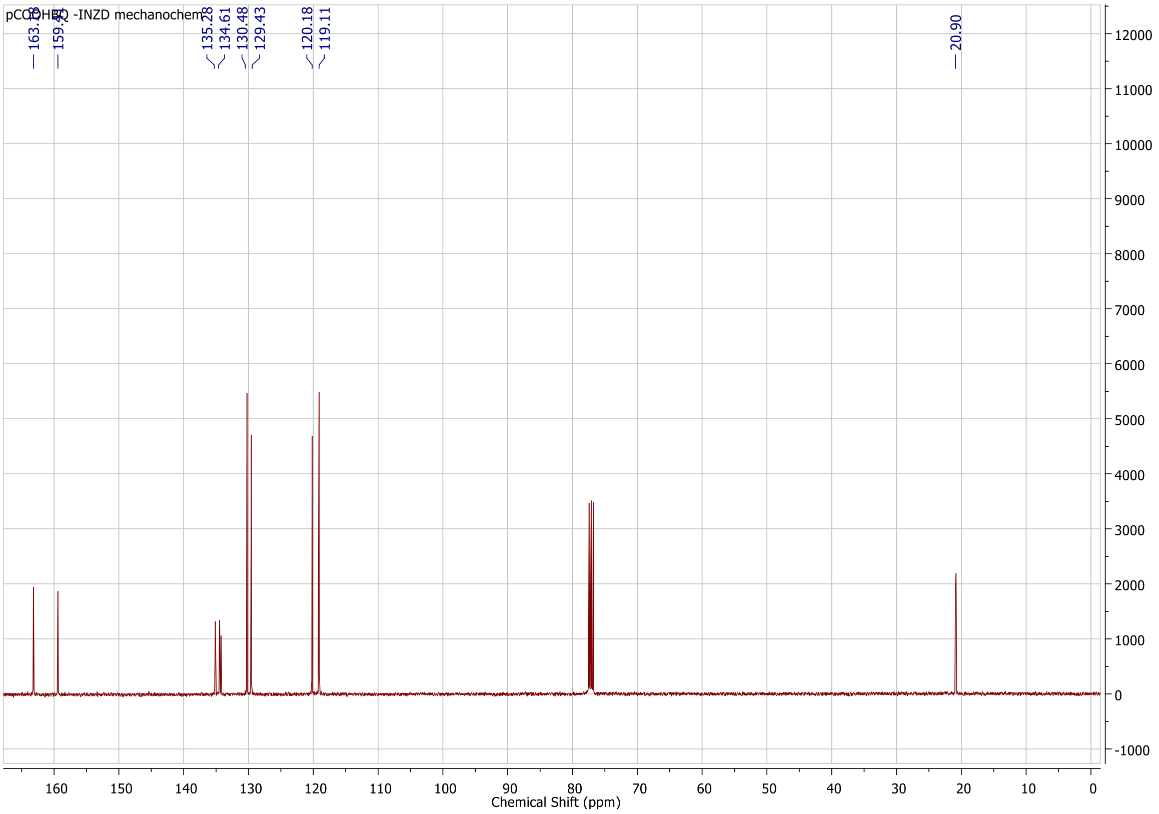

Figure S4.2. ^{13}C NMR (101 MHz, $CDCl_3$) spectrum

2.5. *N*-m-tolylformamide

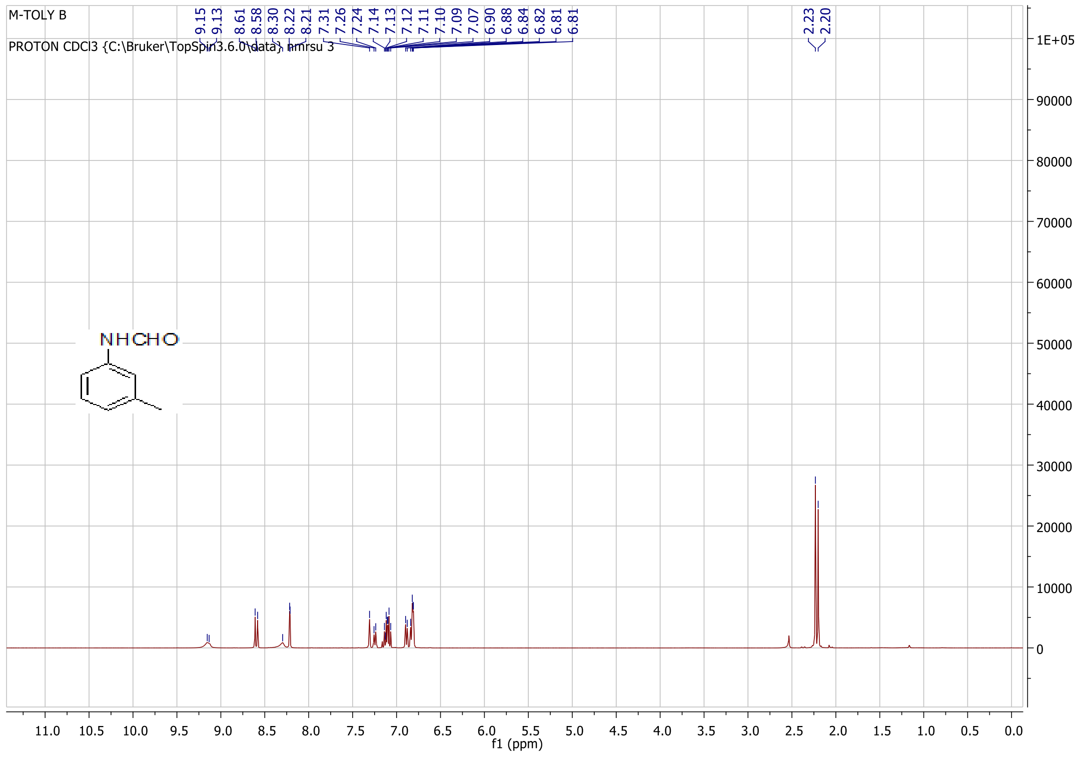

Figure S5.1 ^{1}H NMR (400 MHz, $CDCl_3$) spectrum

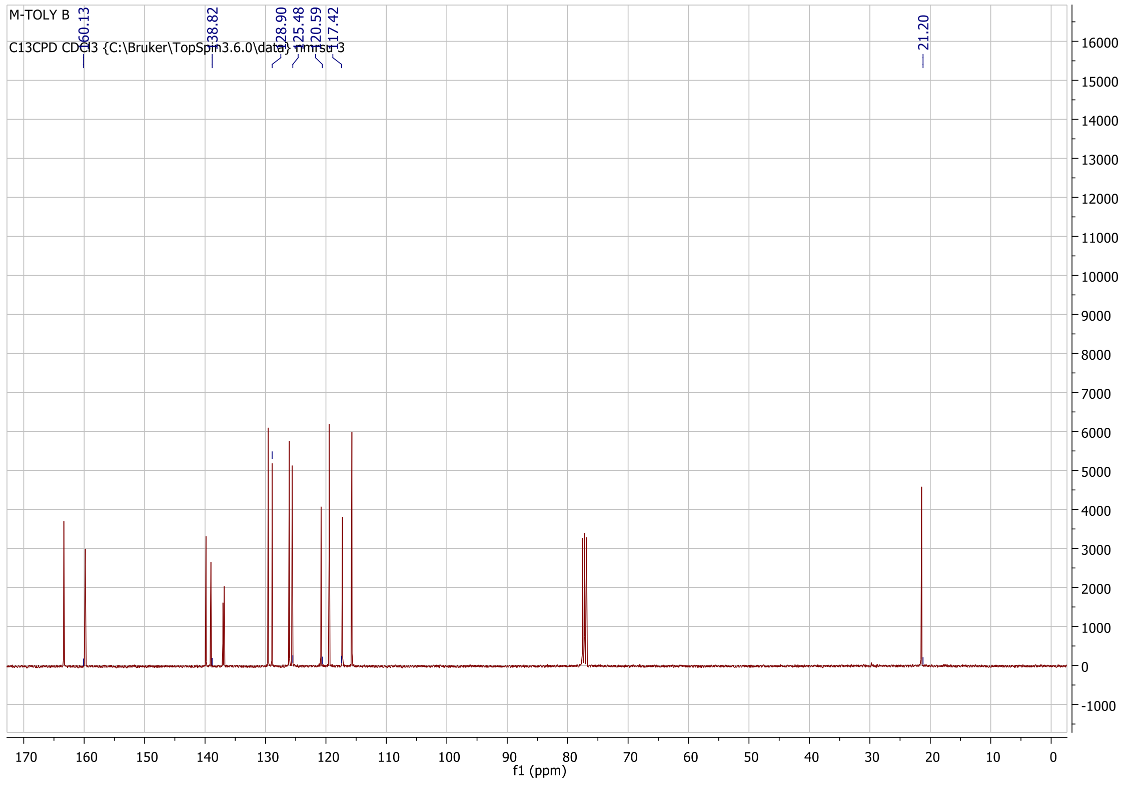

Figure S5.2 ^{13}C NMR (101 MHz, $CDCl_3$) spectrum

2.6. 3,5 dimethylphenyl formamide

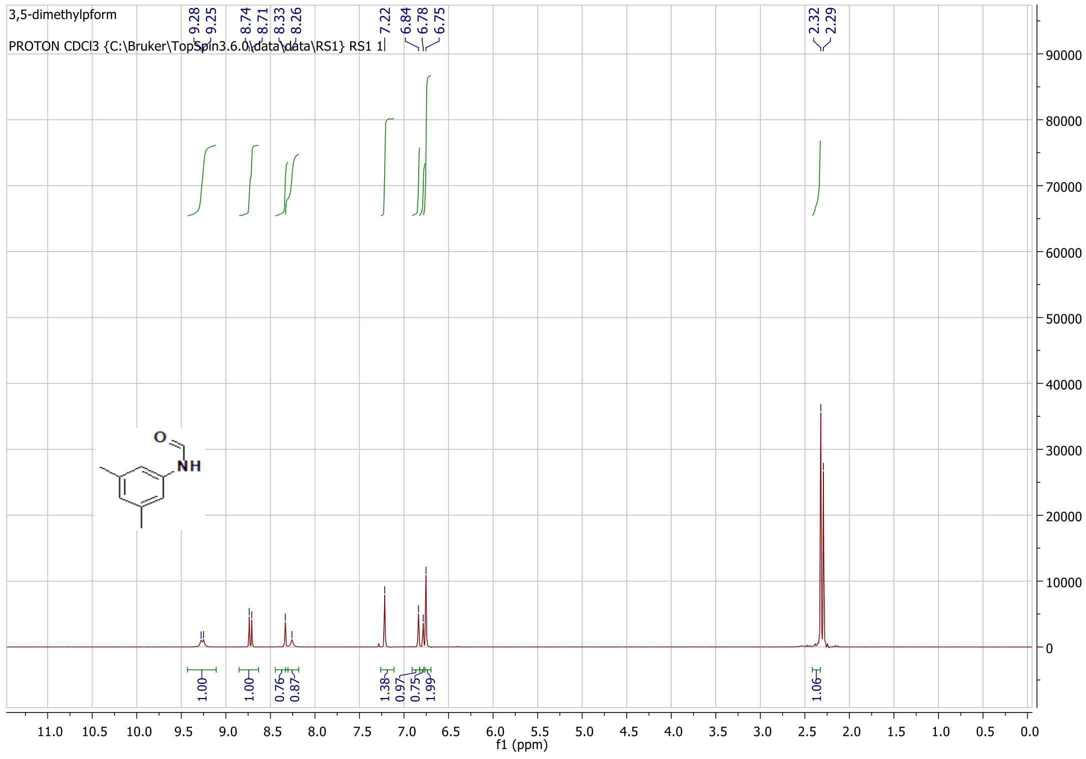

Figure S6.1 ^{1}H NMR (400 MHz, $CDCl_3$) spectrum

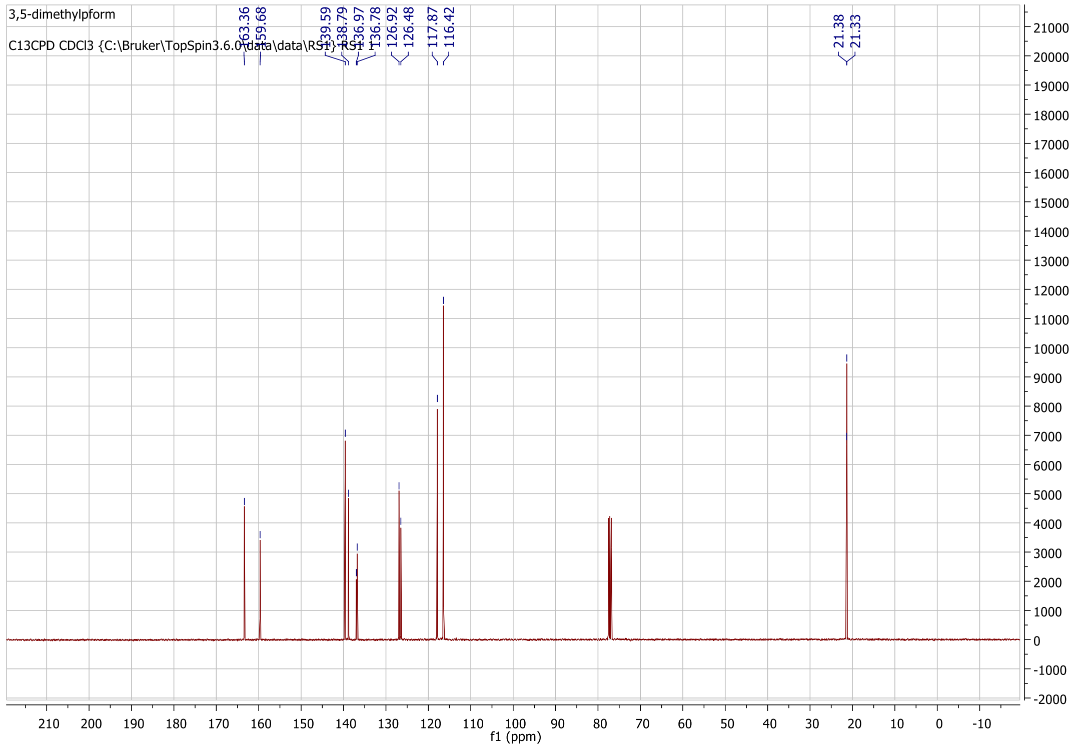

Figure S6.2. ^{13}C NMR (101 MHz, $CDCl_3$) spectrum

2.7. 2,4,6-trimethylphenylformamide

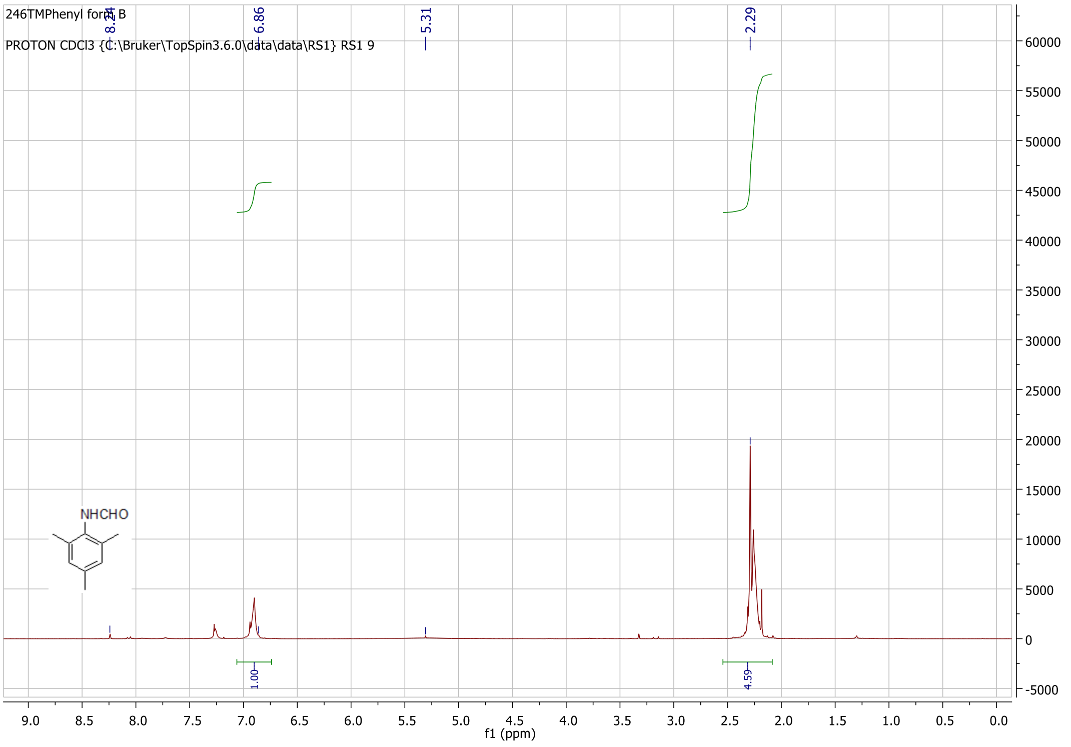

Figure S7.1. ^{1}H NMR (400 MHz, $CDCl_3$) spectrum

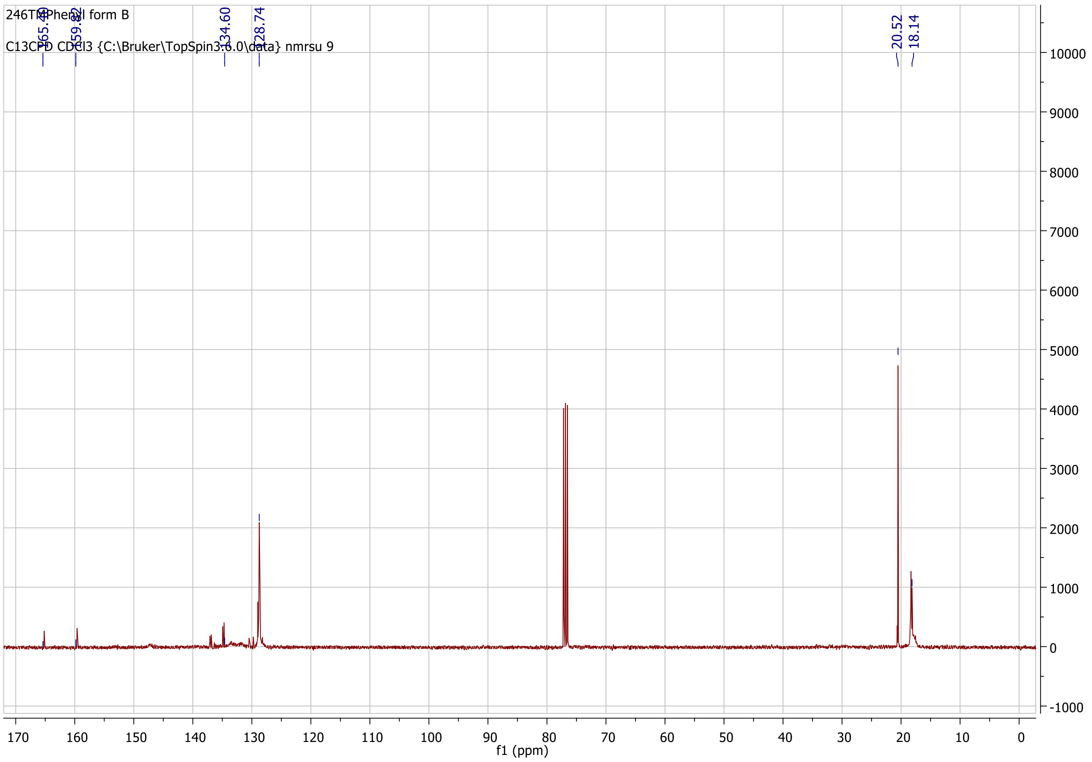

Figure S7.2. ^{13}C NMR (101 MHz, $CDCl_3$) spectrum

2.8. *N*-(4-nitrophenyl) formamide

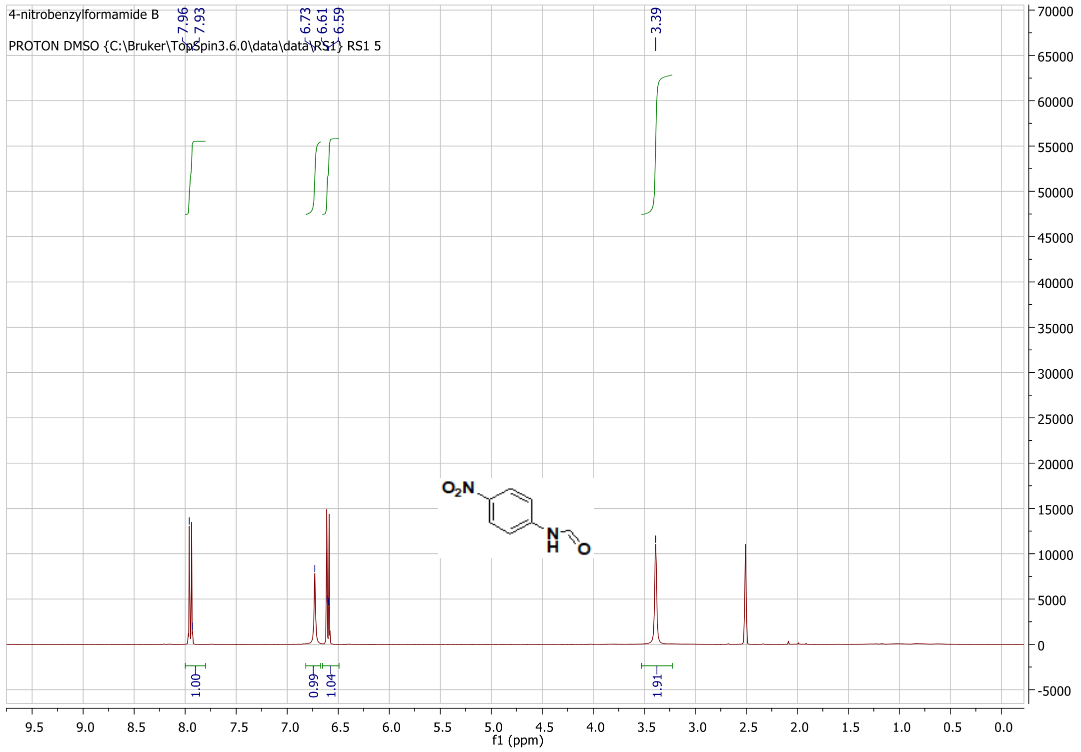

Figure S8.1. ^{1}H NMR (400 MHz, $CDCl_3$) spectrum

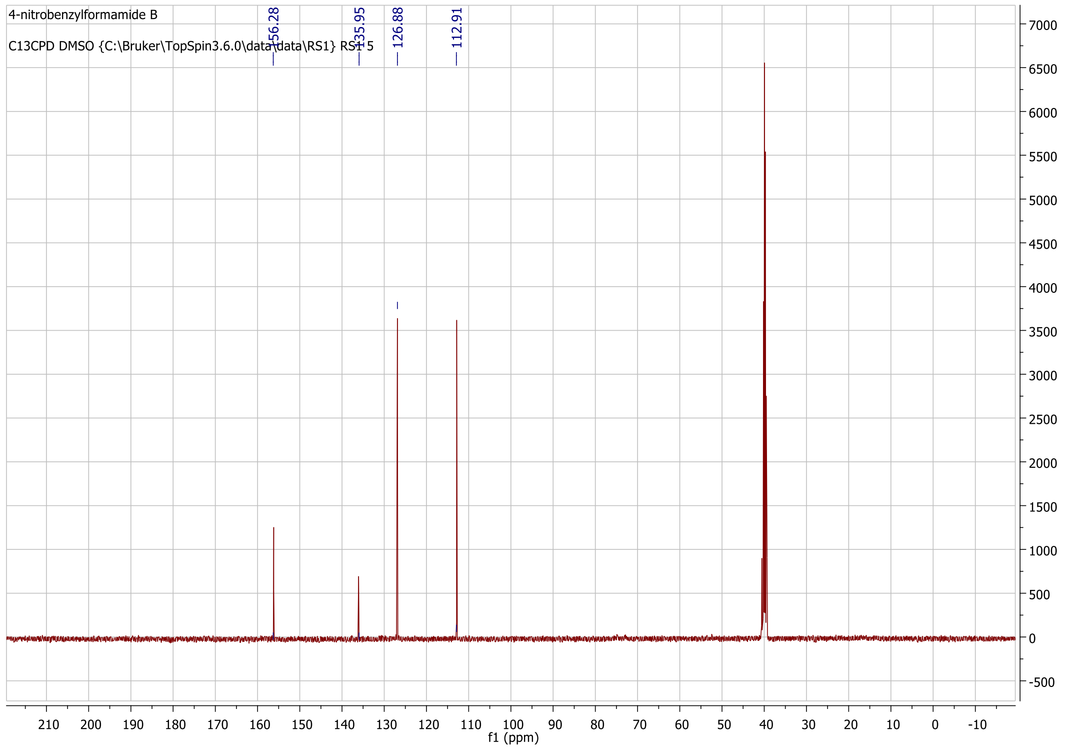

Figure S8.2. ^{13}C NMR (101 MHz, $CDCl_3$) spectrum

2.9. *N*-(2-nitrophenyl) formamide

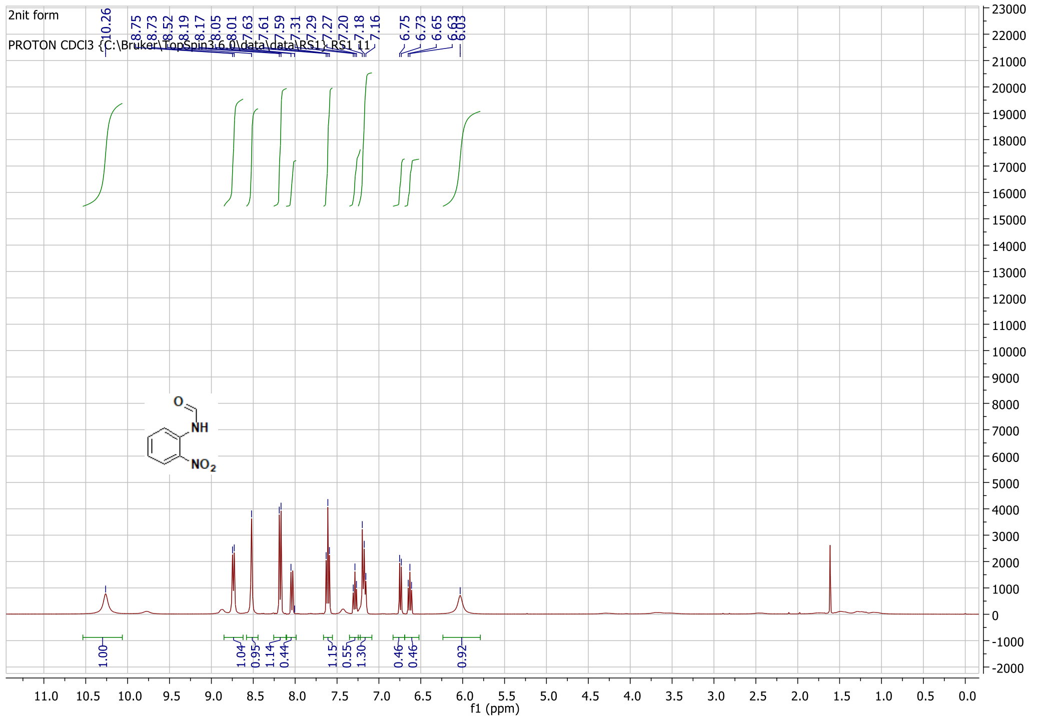

Figure S9.1. ^{1}H NMR (400 MHz, $CDCl_3$) spectrum

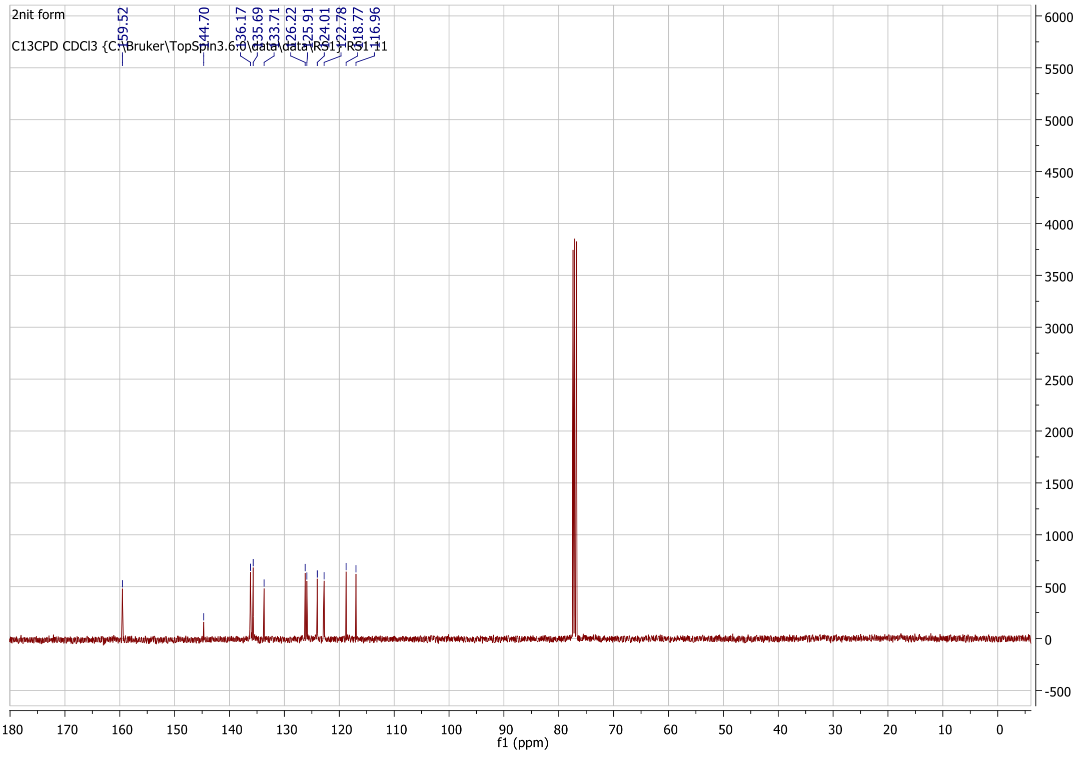

Figure S9.2. ^{13}C NMR (101 MHz, $CDCl_3$) spectrum

2.10. *N*-(4-methyl-2-nitrophenyl)formamide

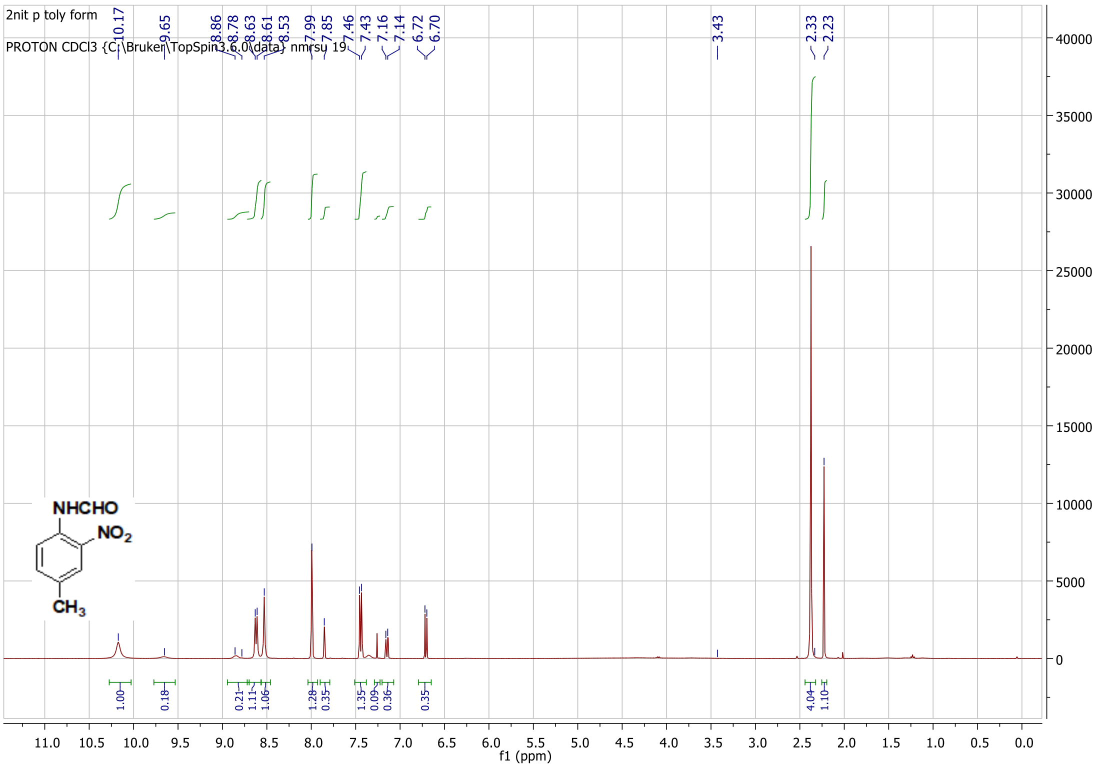

Figure S10.1 ^{1}H NMR (400 MHz, $CDCl_3$) spectrum

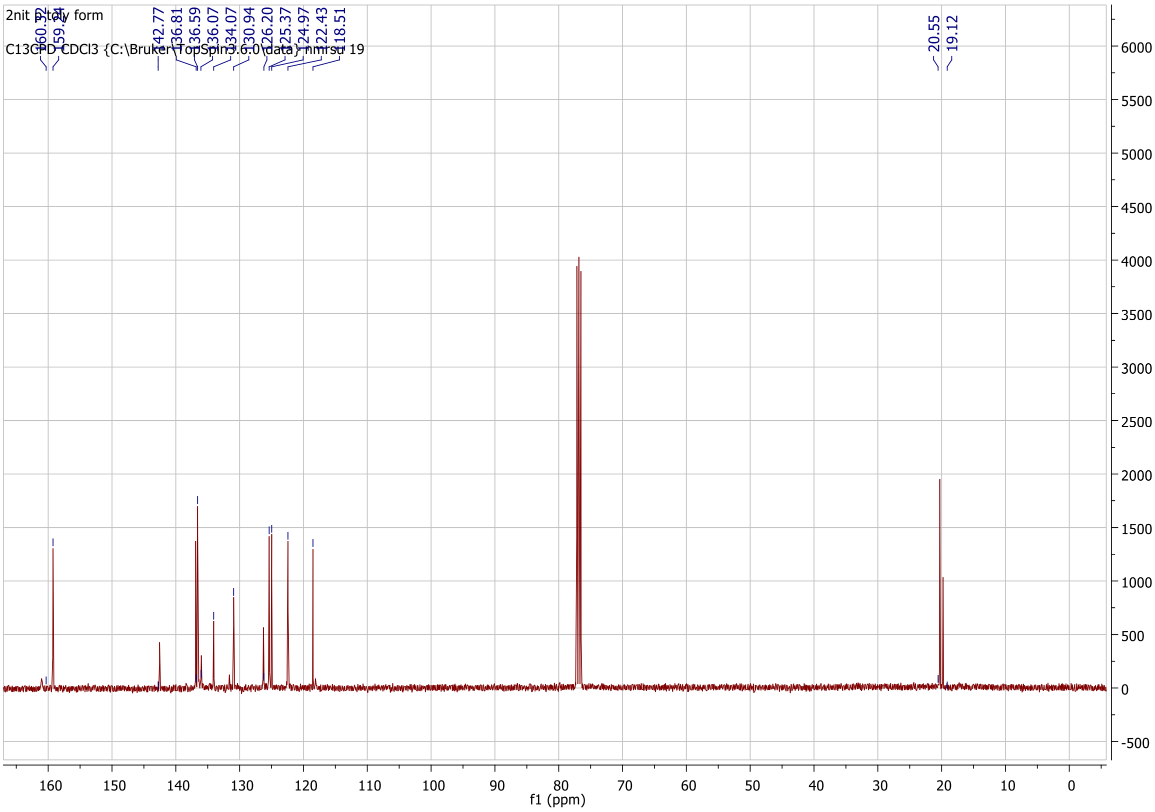

Figure S10.2. ^{13}C NMR (101 MHz, $CDCl_3$) spectrum

2.11. *N*-(2-hydroxy-5-nitrophenyl) formamide

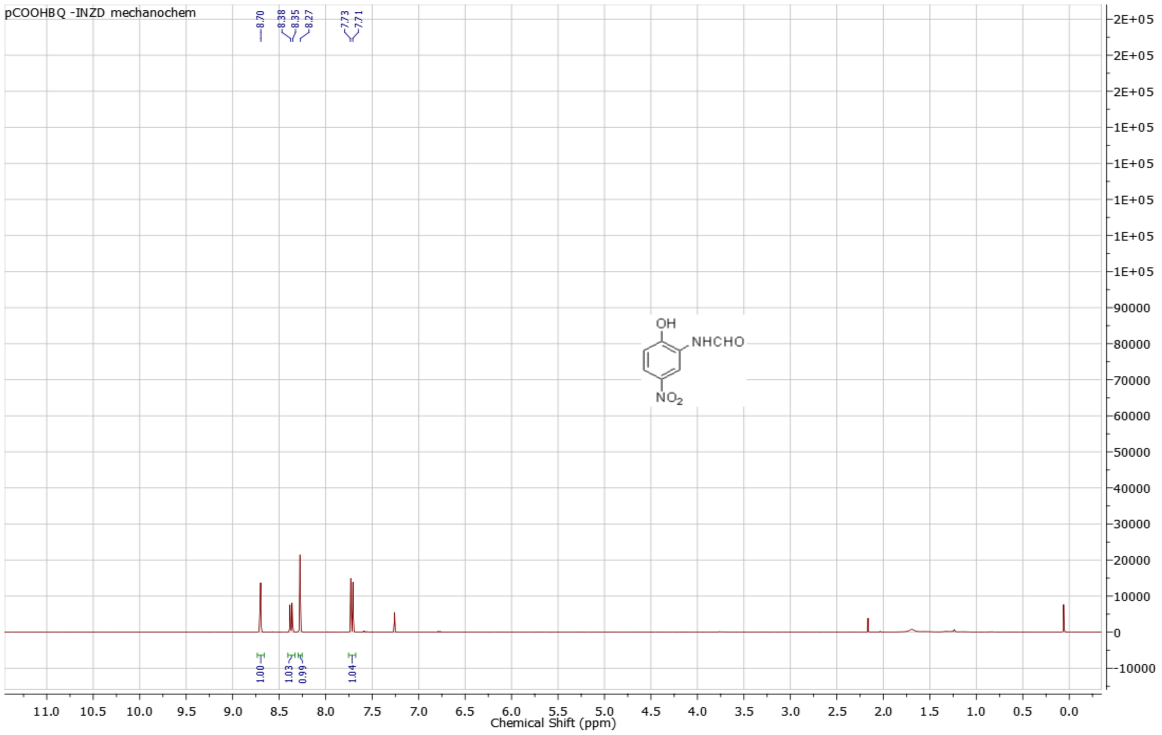

Figure S11.1. ^{1}H NMR (400 MHz, $CDCl_3$) spectrum

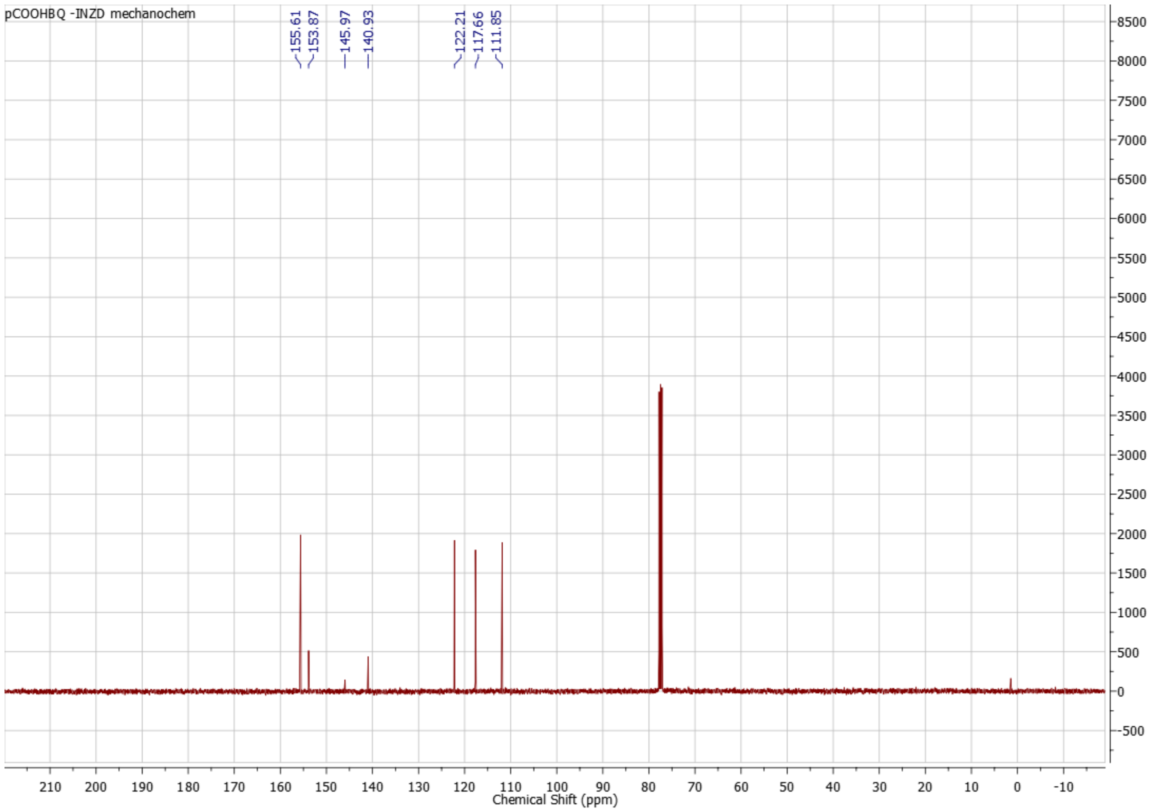

Figure S11.2. ^{13}C NMR (101 MHz, $CDCl_3$) spectrum

2.12. *N*-(4-hydroxyphenyl)formamide

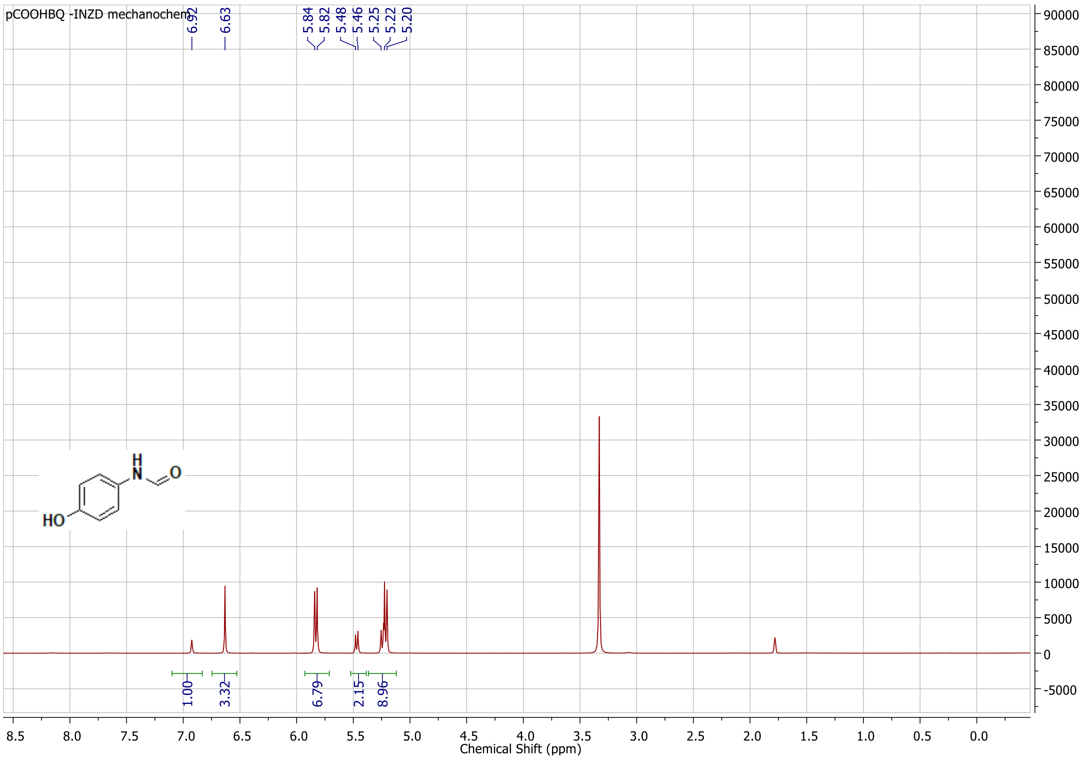

Figure S12.1. ^{1}H NMR (400 MHz, DMSO-d_6) spectrum

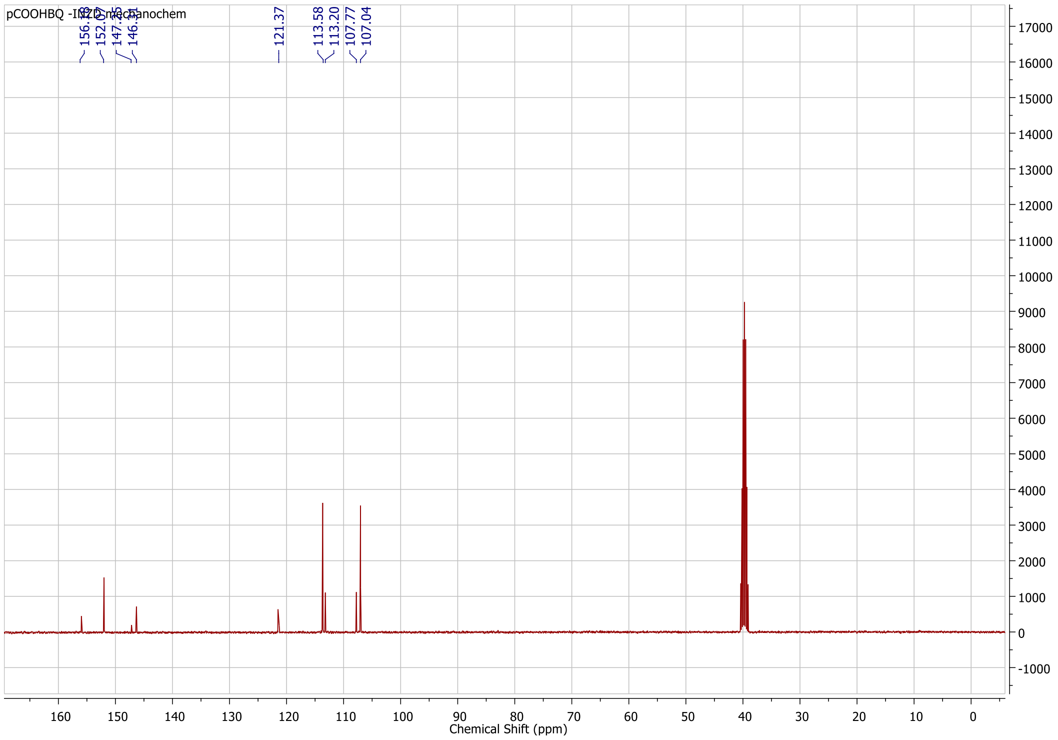

Figure S12.2. ^{13}C NMR (101 MHz, DMSO-d_6) spectrum

2.13. *N*-(2-hydroxyphenyl)formamide

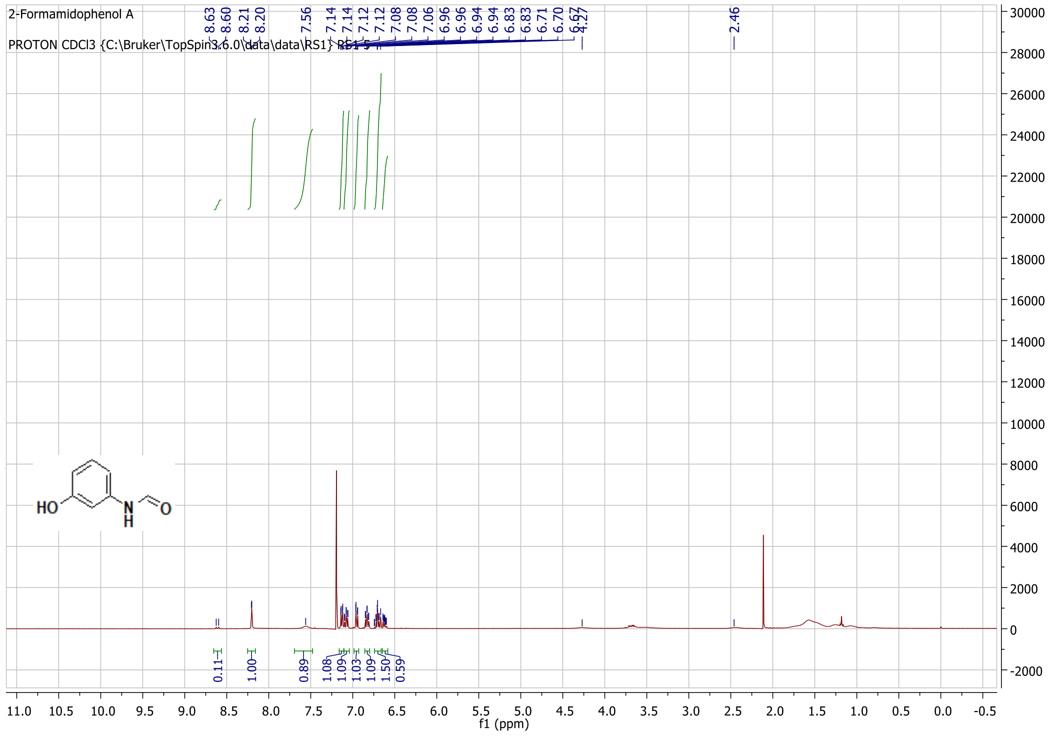

Figure S13.1. ^{1}H NMR (400 MHz, $CDCl_3$) spectrum

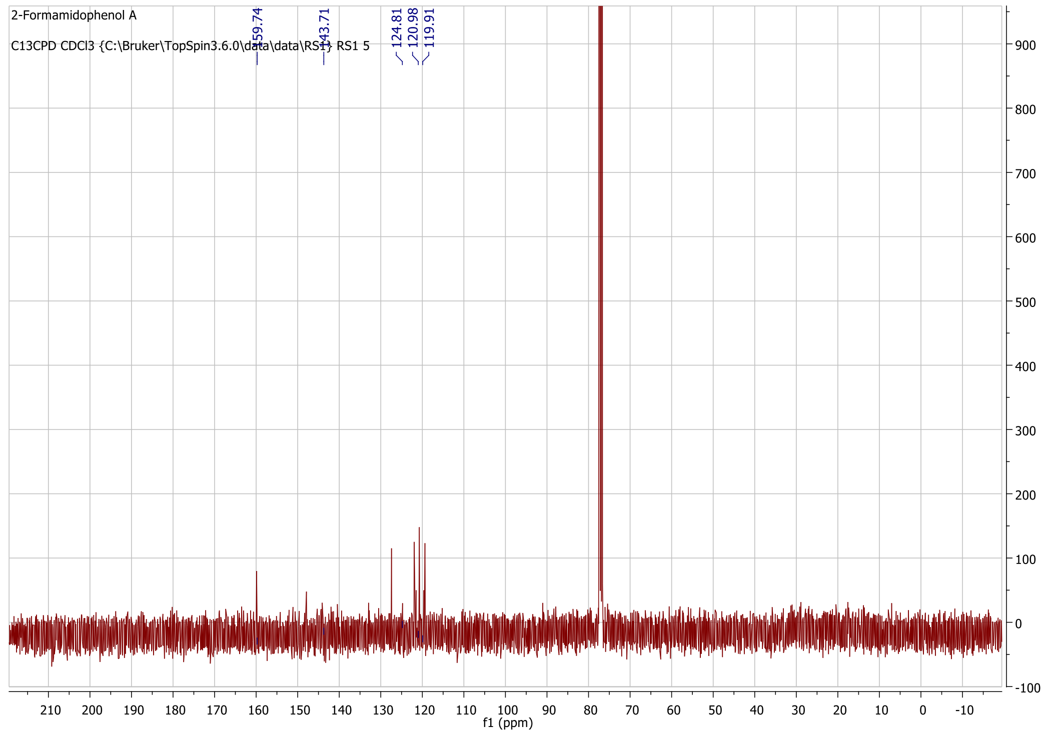

Figure S13.2. ^{13}C NMR (101 MHz, $CDCl_3$) spectrum

2.14. 4-Formamidobenzoic acid

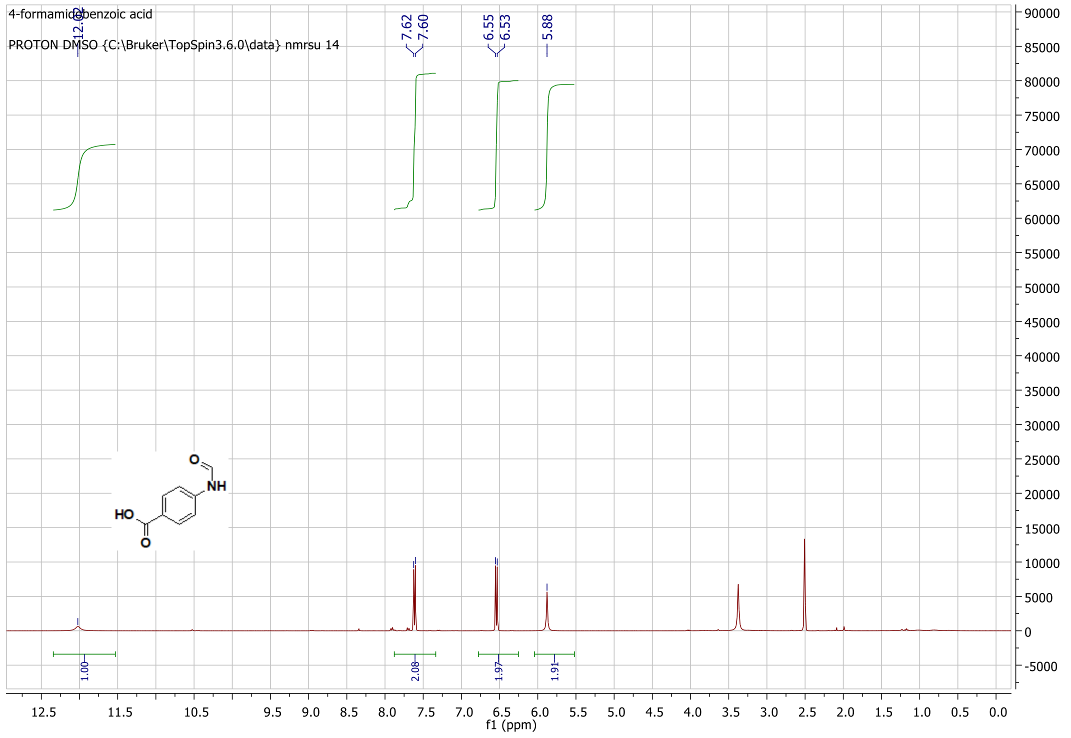

Figure S14.1 ^{1}H NMR (400 MHz, DMSO-d_6) spectrum

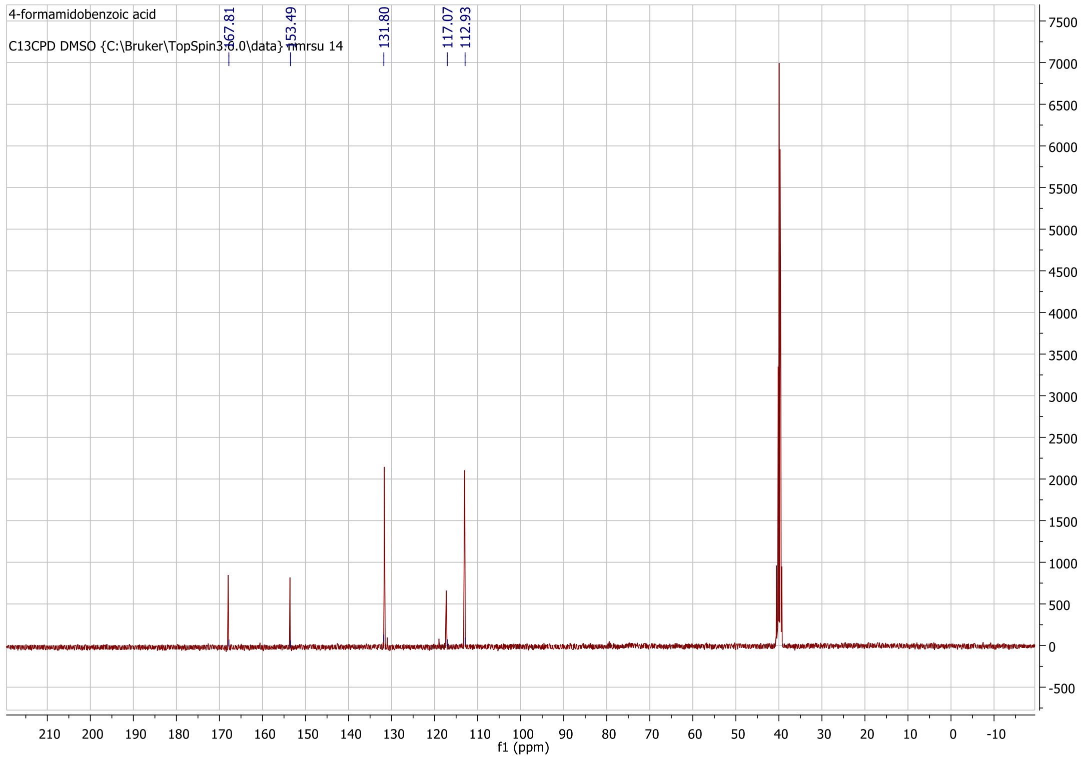

Figure S14.2 ^{13}C NMR (101 MHz, DMSO-d_6) spectrum

2.15. 3-Formamidobenzoic acid

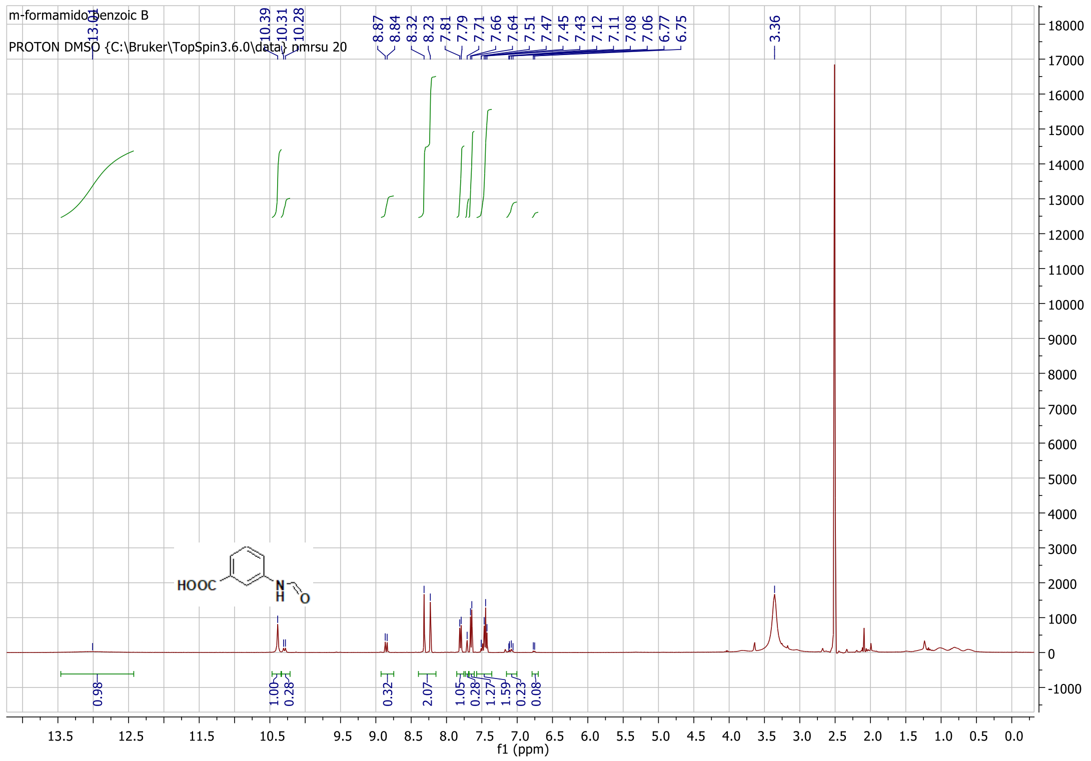

Figure S15.1. ^{1}H NMR (400 MHz, DMSO-d_6) spectrum

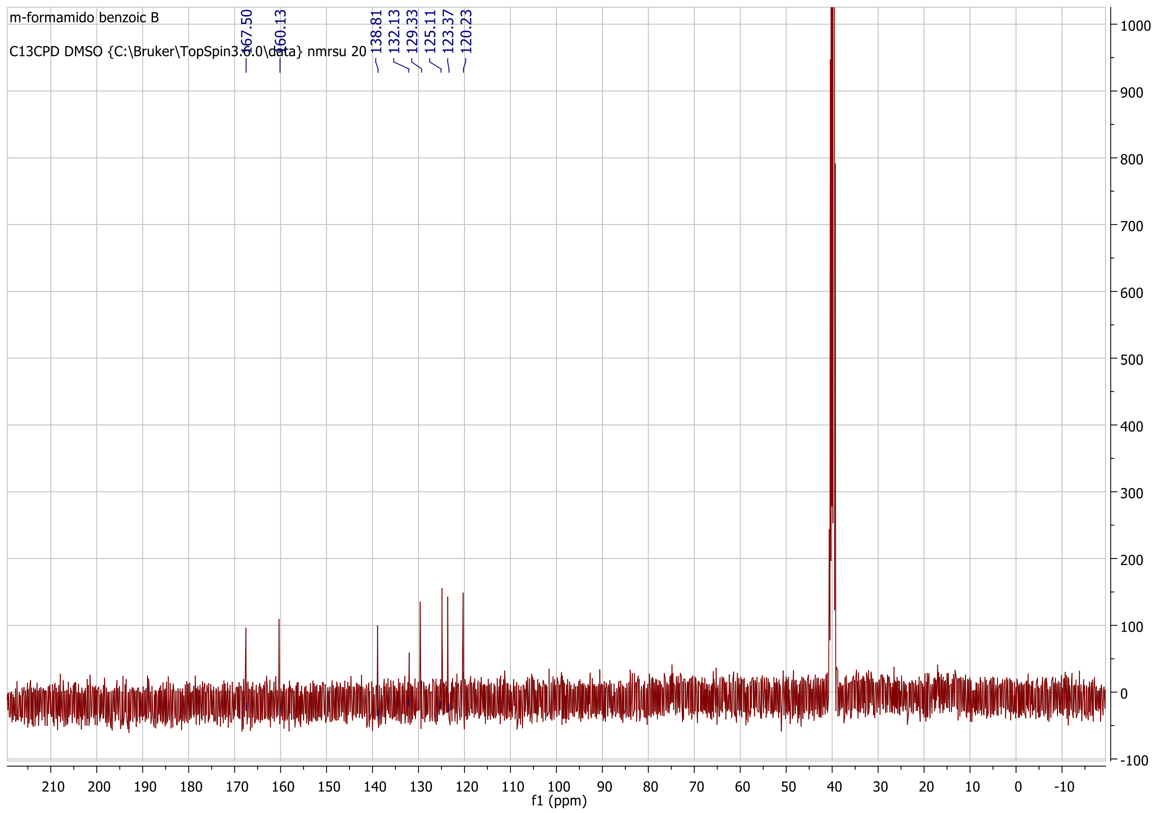

Figure S15.2. ^{13}C NMR (101 MHz, DMSO-d_6) spectrum

2.16. 4-formamido-2-hydroxybenzoic acid

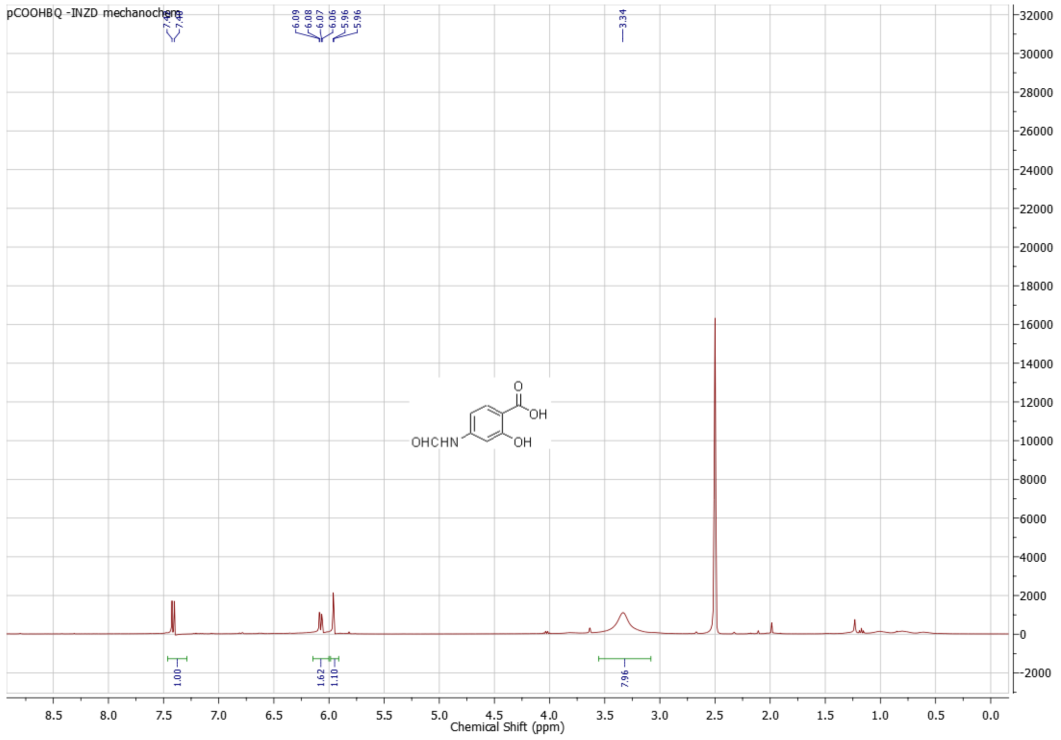

Figure S16.1. ^{1}H NMR (400 MHz, DMSO-d_6) spectrum

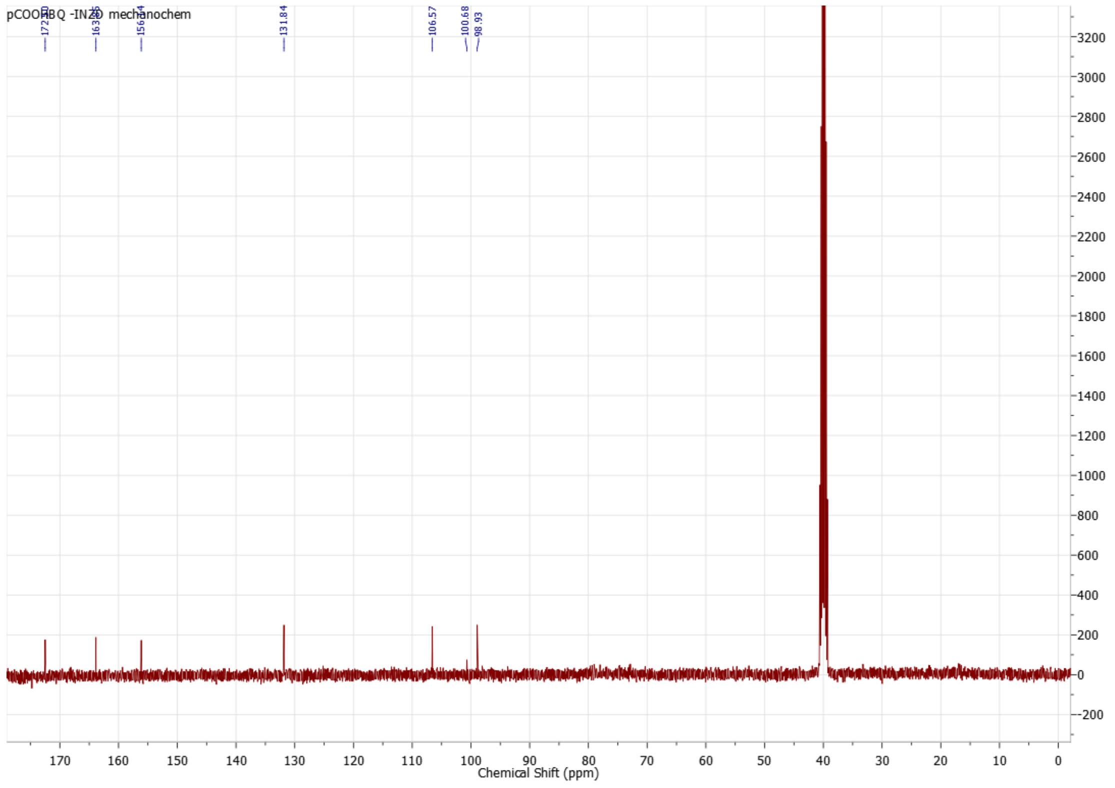

Figure S16.2. ^{13}C NMR (101 MHz, DMSO-d_6) spectrum

2.17. 5-chloro-2-formamidobenzoic acid

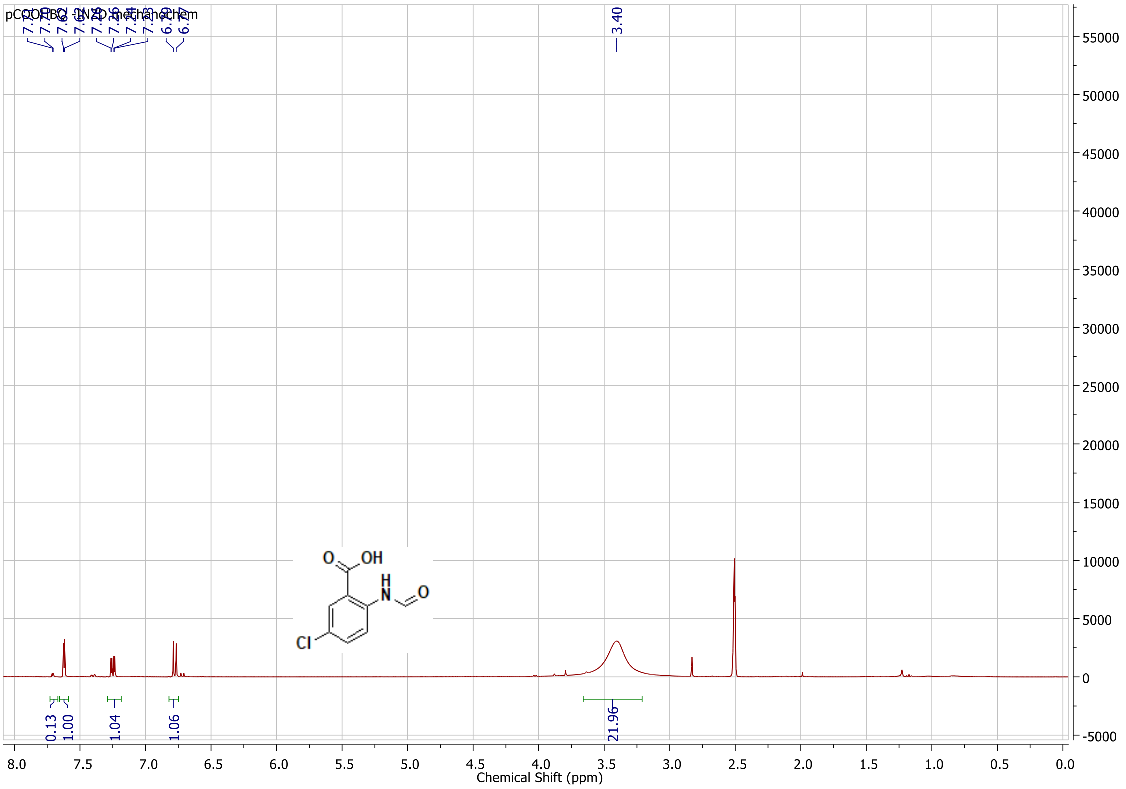

Figure S17.1. ^{1}H NMR (400 MHz, DMSO-d_6) spectrum

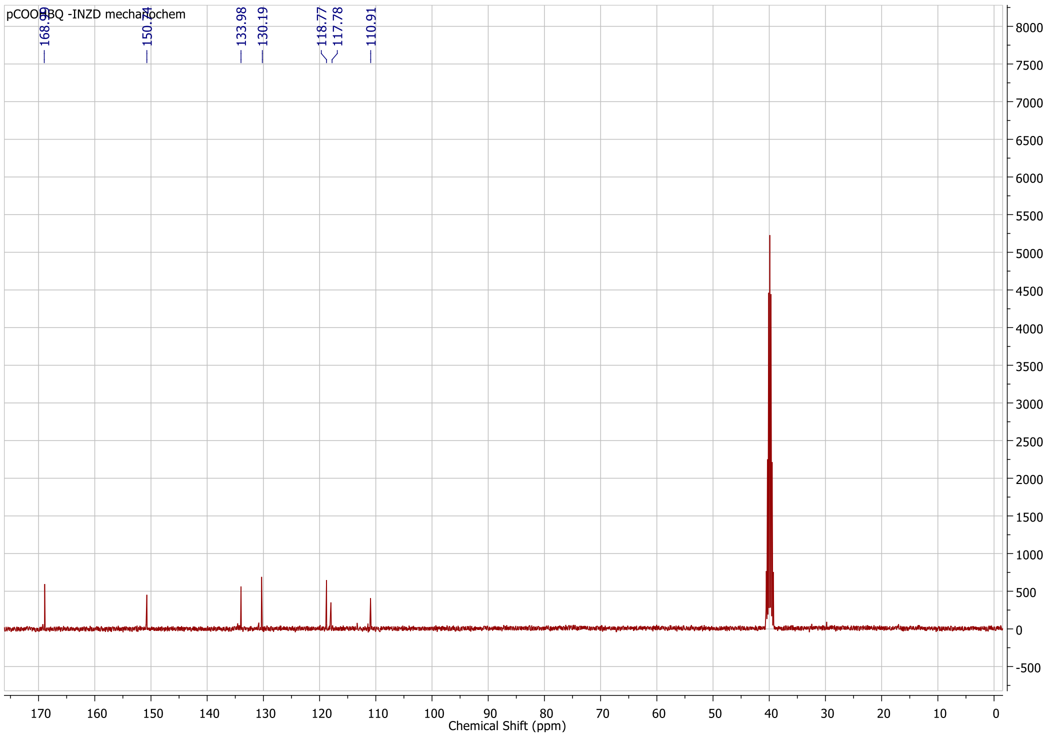

Figure S17.2. ^{13}C NMR (101 MHz, DMSO-d_6) spectrum

2.18. 4-Formamido phenyl acetic acid

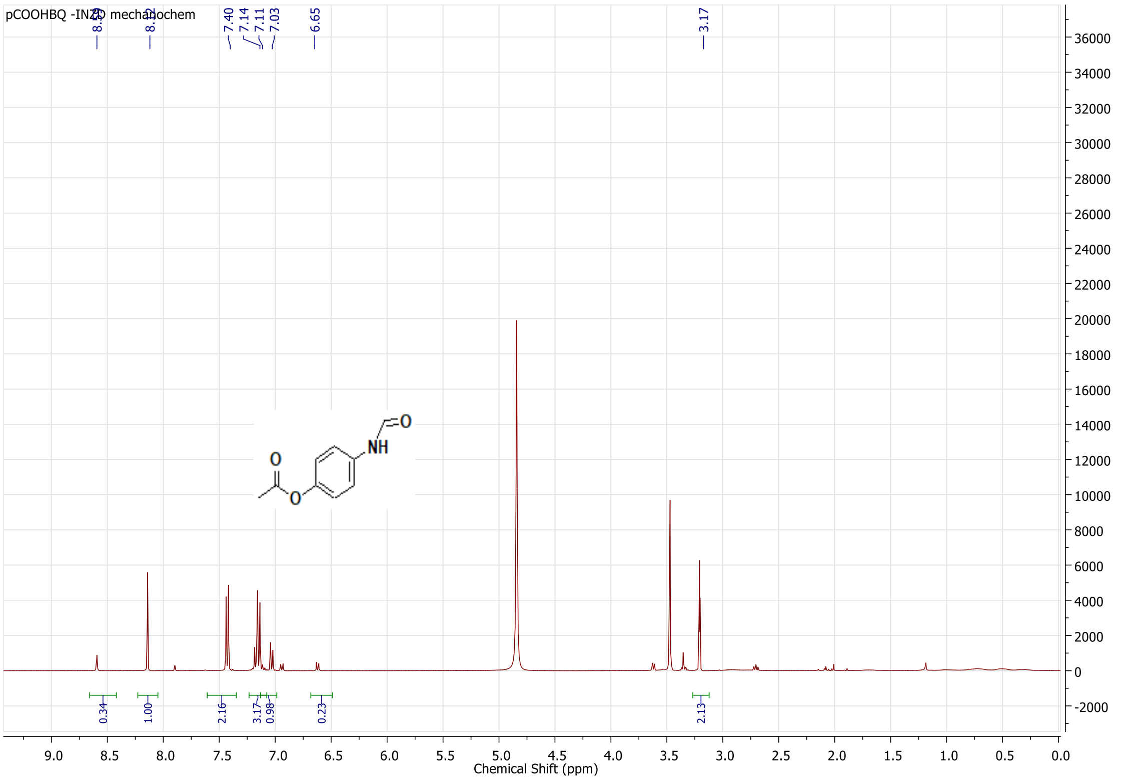

Figure S18.1 ^{1}H NMR (400 MHz, MeOD) spectrum

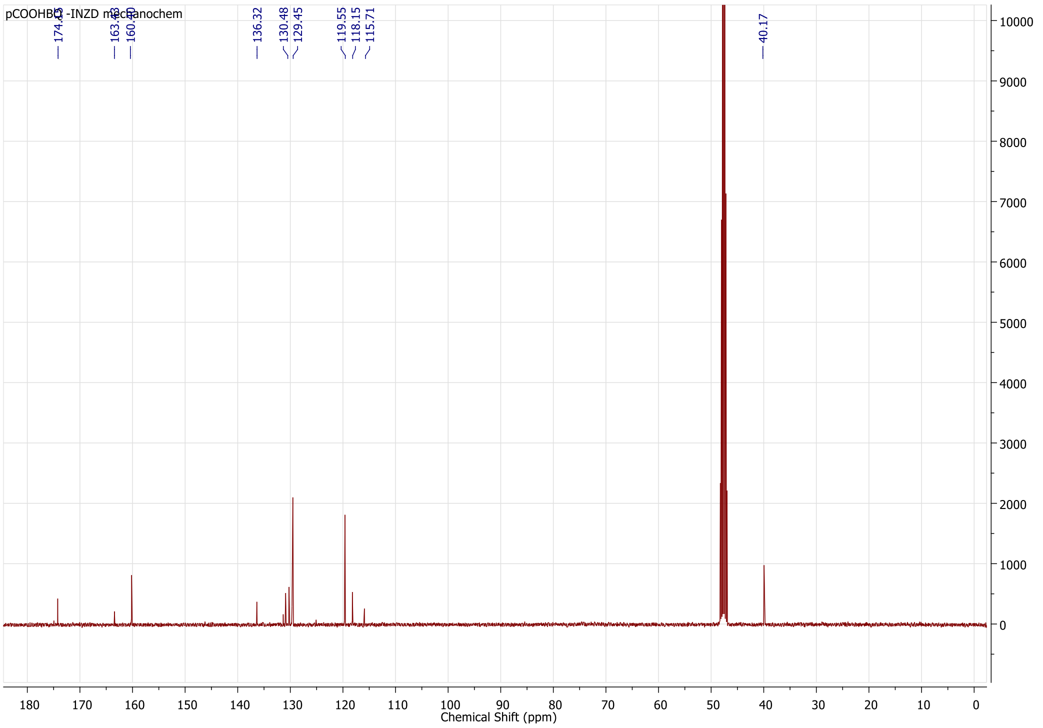

Figure S18.2. ^{13}C NMR (101 MHz, MeOD) spectrum

2.19. 3-Fluorophenylformamide

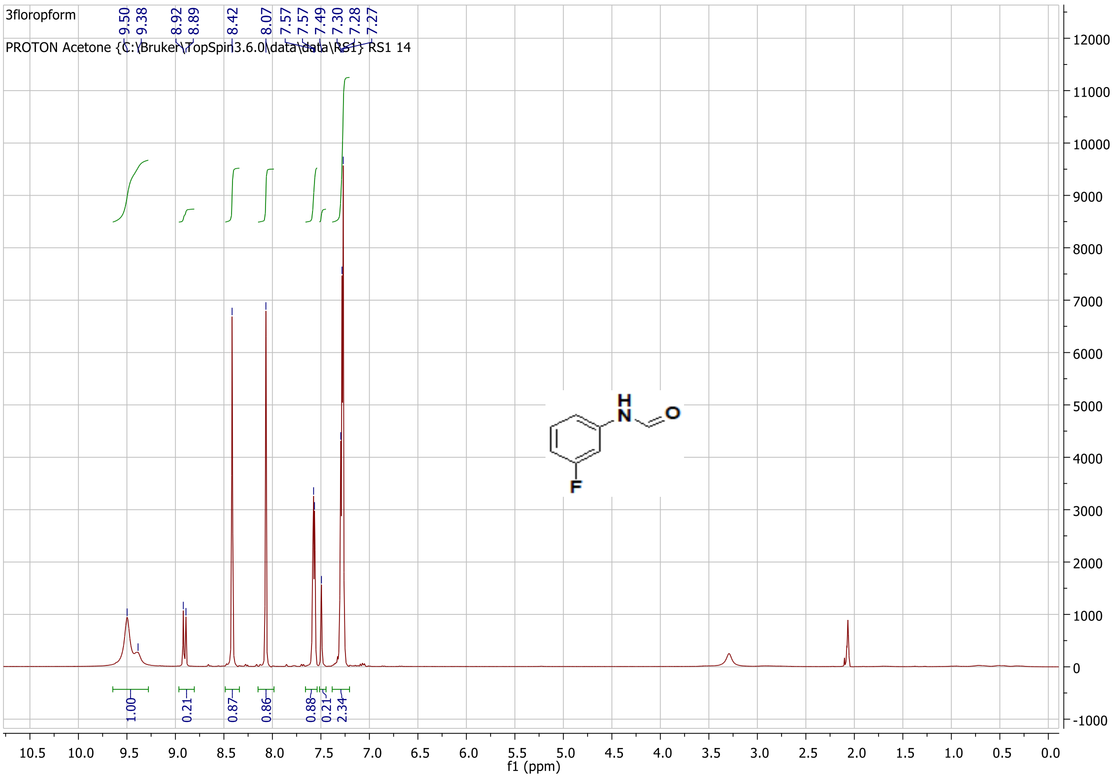

Figure S19.1. ^{1}H NMR (400 MHz, Acetone-d_6) spectrum

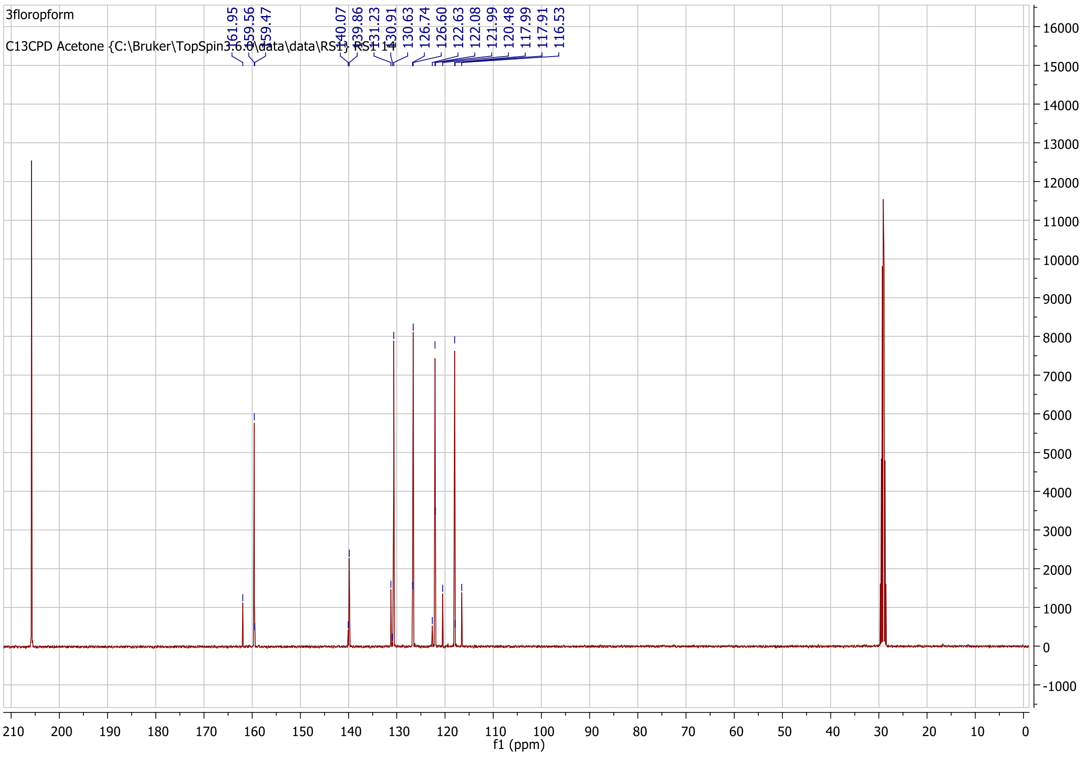

Figure S19.2 ^{13}C NMR spectrum (101 MHz, Acetone-d_6) spectrum

2.20. 3-Bromophenylformamide

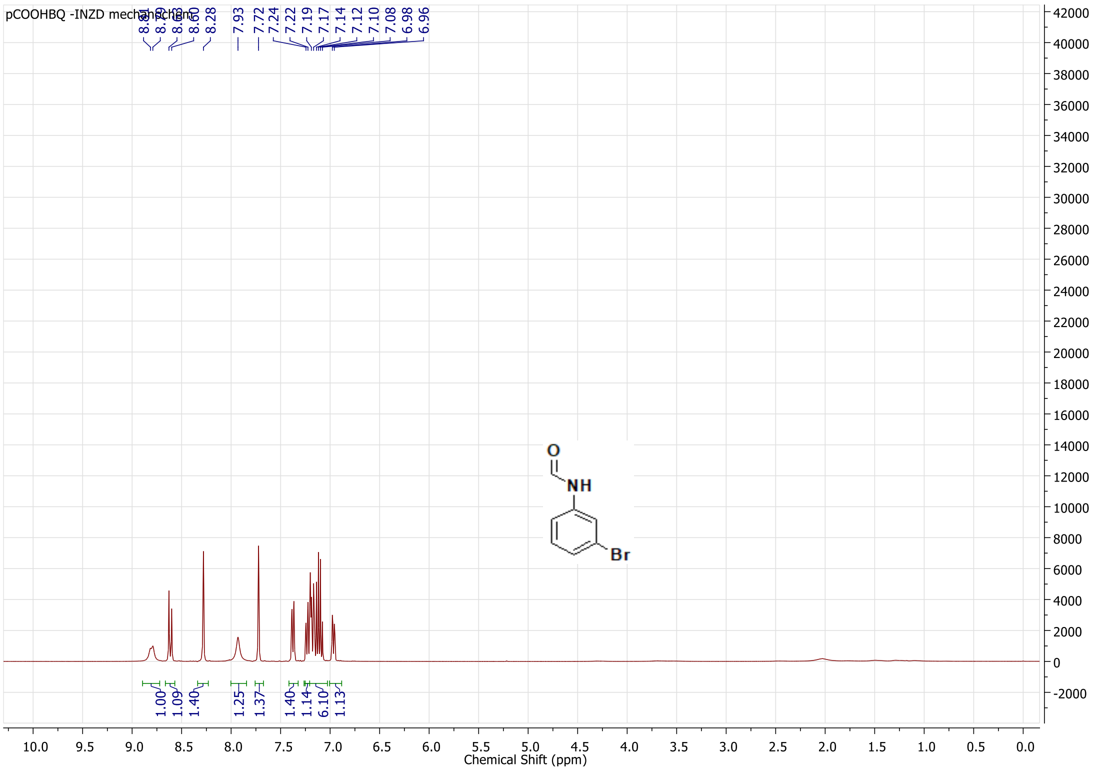

Figure S20.1. ^{1}H NMR (400 MHz, $CDCl_3$) spectrum

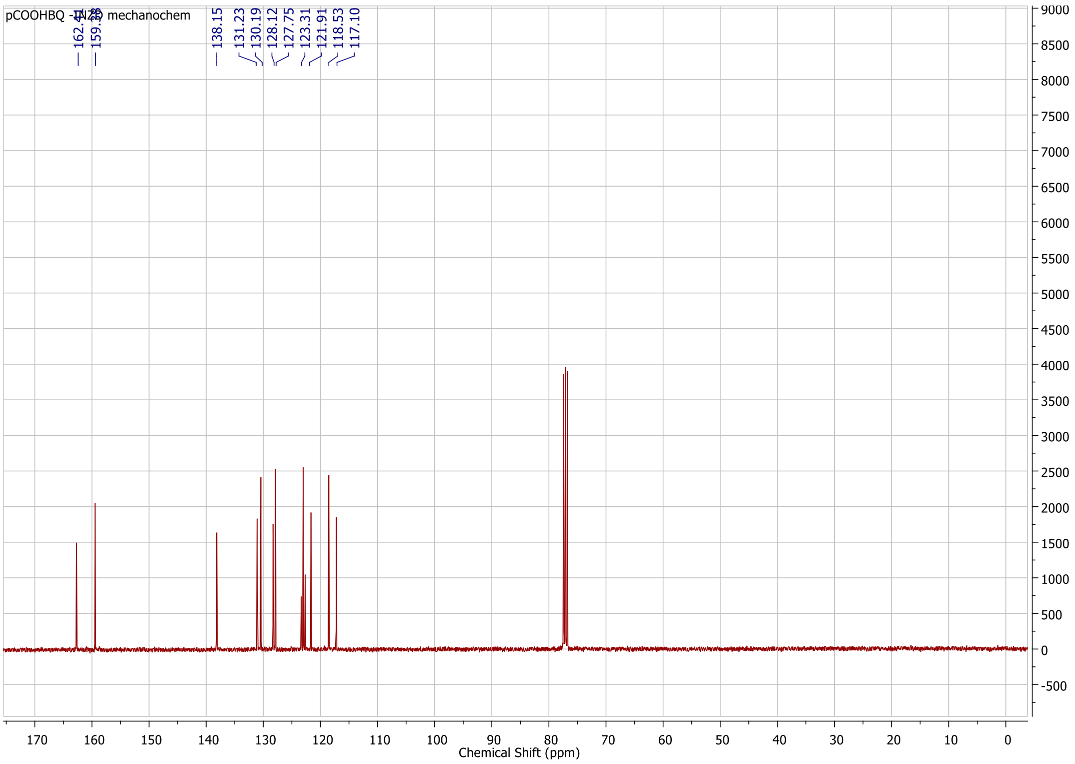

Figure S20.2 ^{13}C NMR (101 MHz, $CDCl_3$) spectrum

2.21. *N*-(2-Iodo-phenyl) formamide

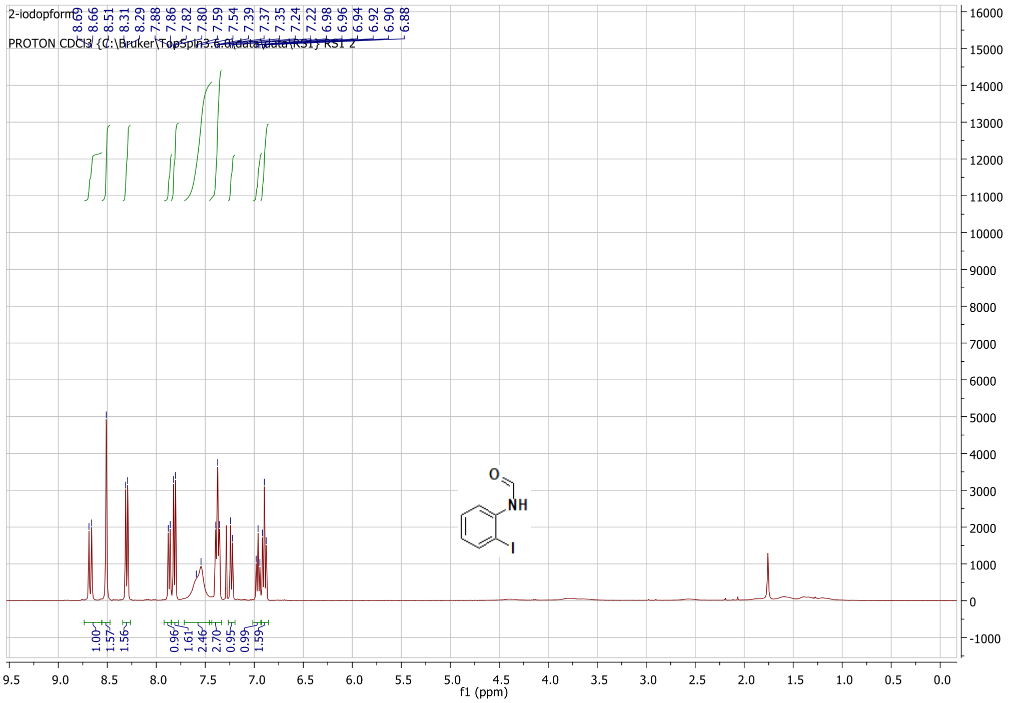

Figure S21.1 ^{1}H NMR (400 MHz, $CDCl_3$) spectrum

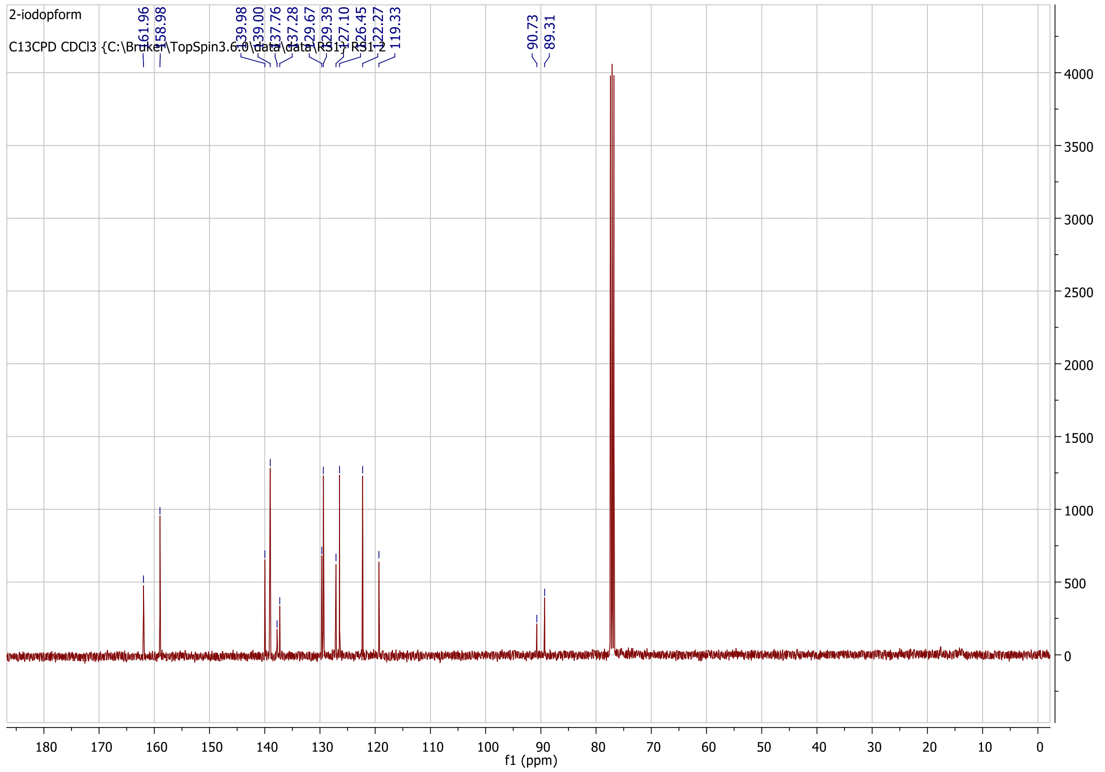

Figure S21.2. ^{13}C NMR (101 MHz, $CDCl_3$) spectrum

2.22. *N*-(2-chlorophenyl)formamide

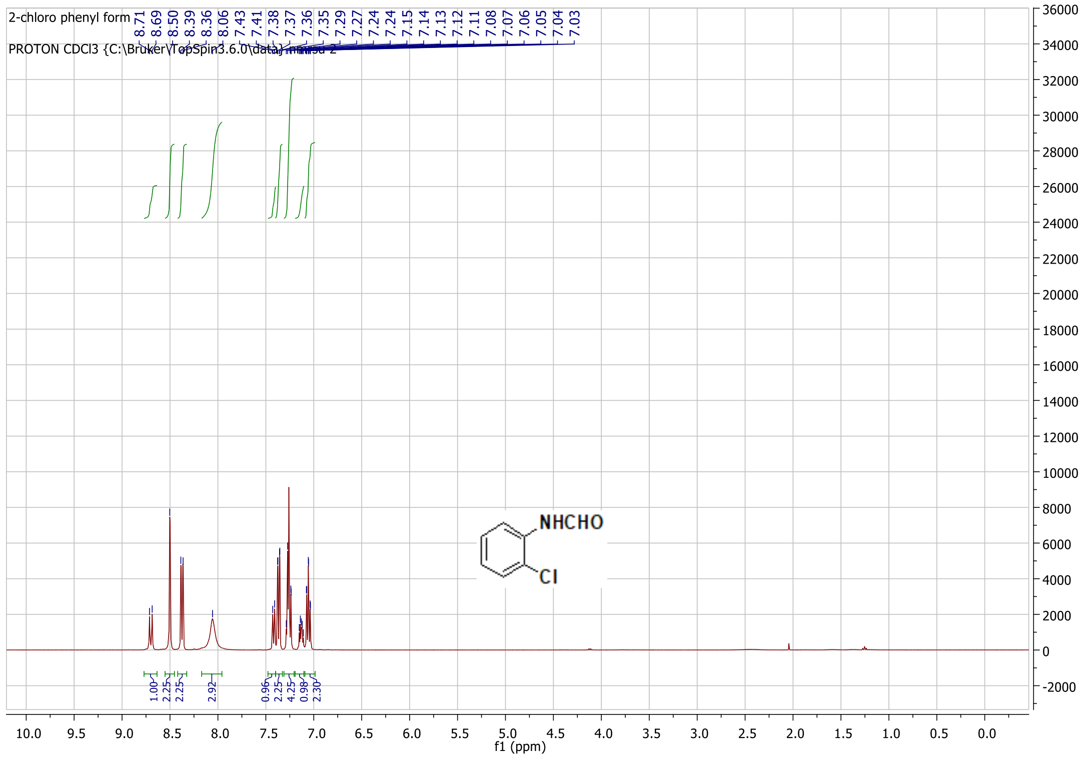

Figure S22.1 ^{1}H NMR (400 MHz, $CDCl_3$) spectrum

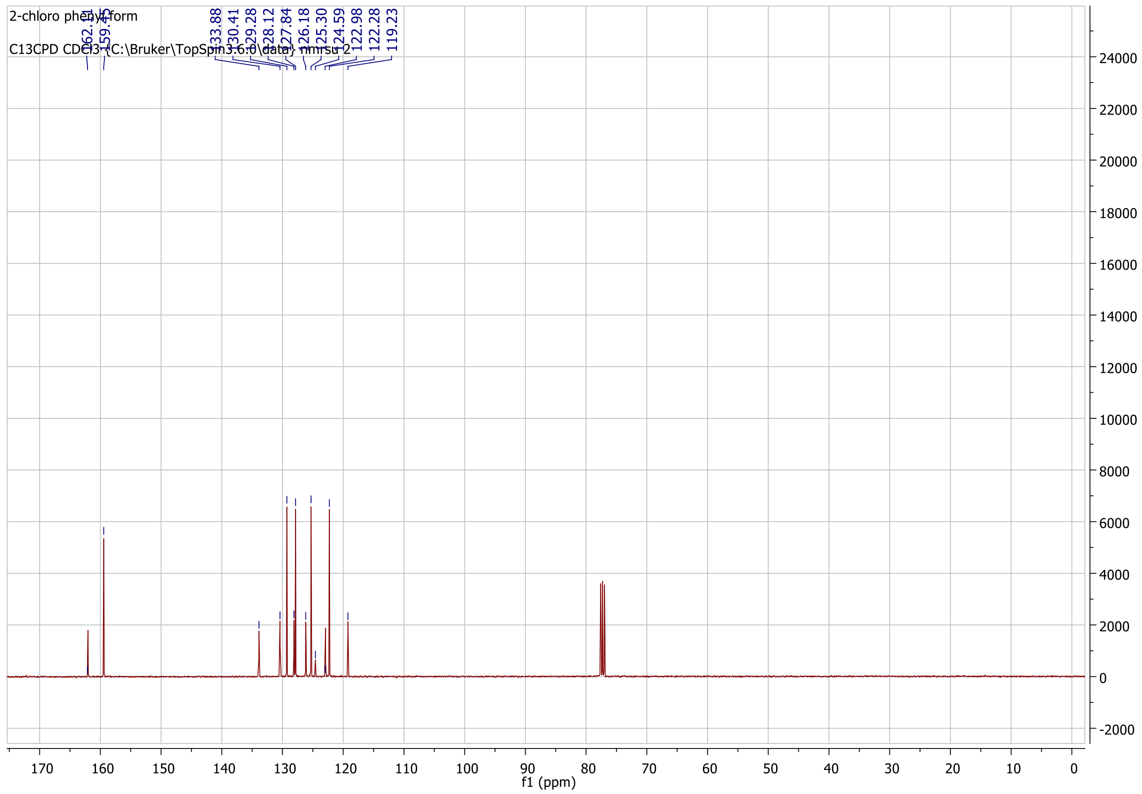

Figure S22.2. ^{13}C NMR (101 MHz, $CDCl_3$) spectrum

2.23. *N*-(2-bromophenyl)formamide

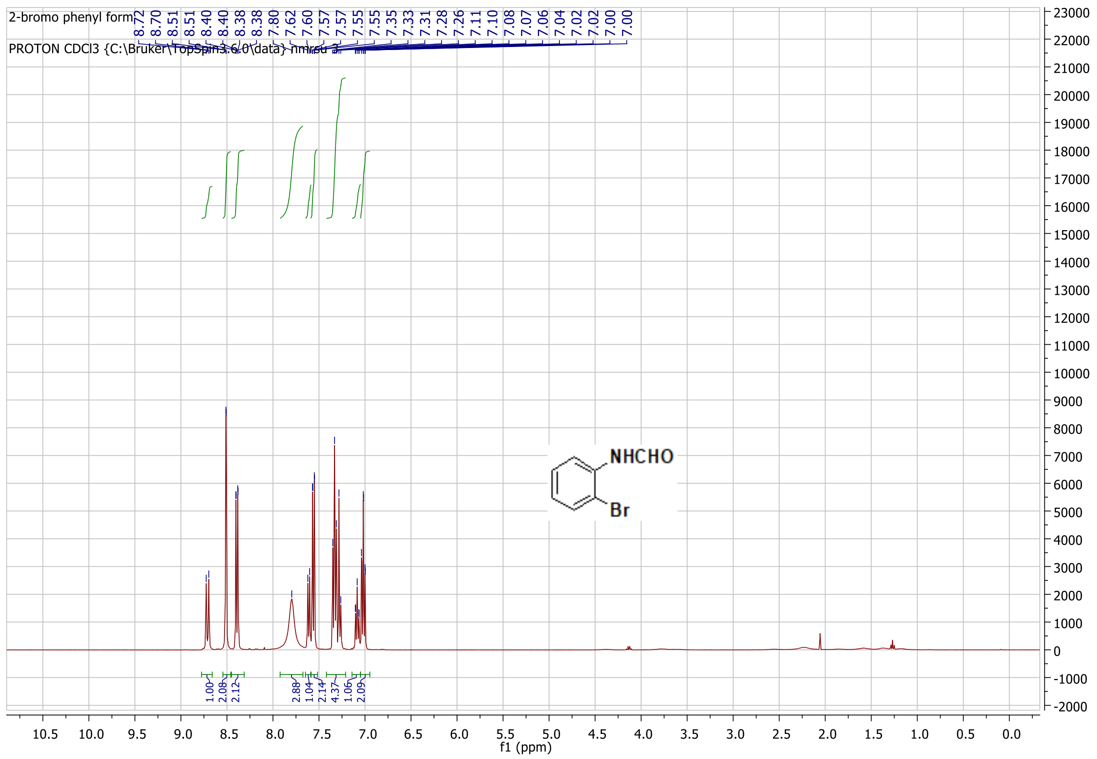

Figure S23.1 ^{1}H NMR (400 MHz, $CDCl_3$) spectrum

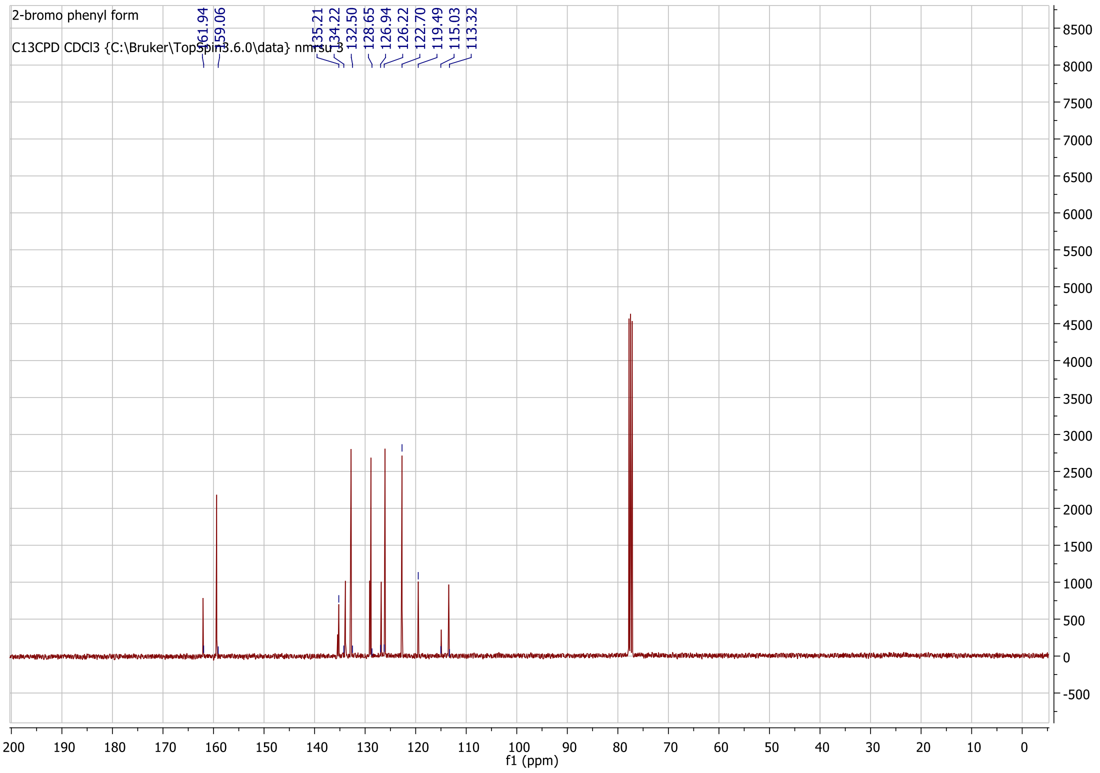

Figure S23.2. ^{13}C NMR (101 MHz, $CDCl_3$) spectrum

2.24. *N*-(4-bromophenyl)formamide

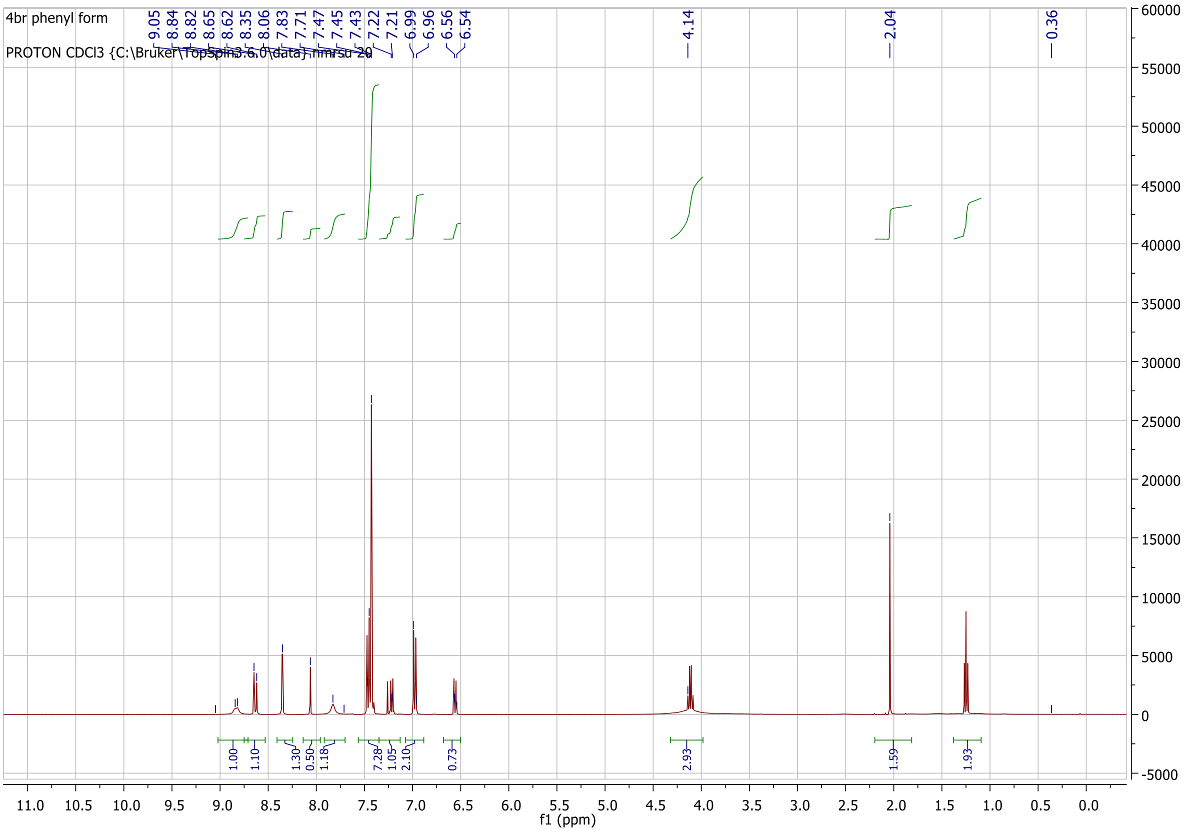

Figure S24.1. ^{1}H NMR (400 MHz, $CDCl_3$) spectrum

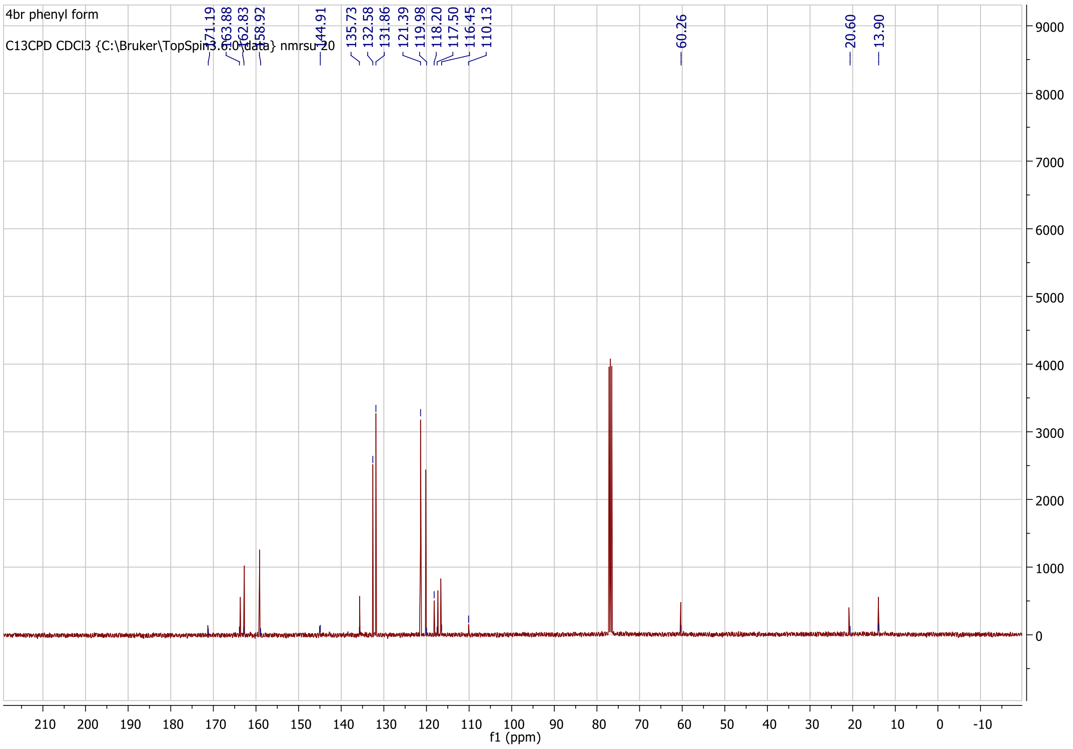

Figure S24.2 ^{13}C NMR (101 MHz, $CDCl_3$) spectrum

2.25. *N*-(3,4-difluorophenyl) formamide

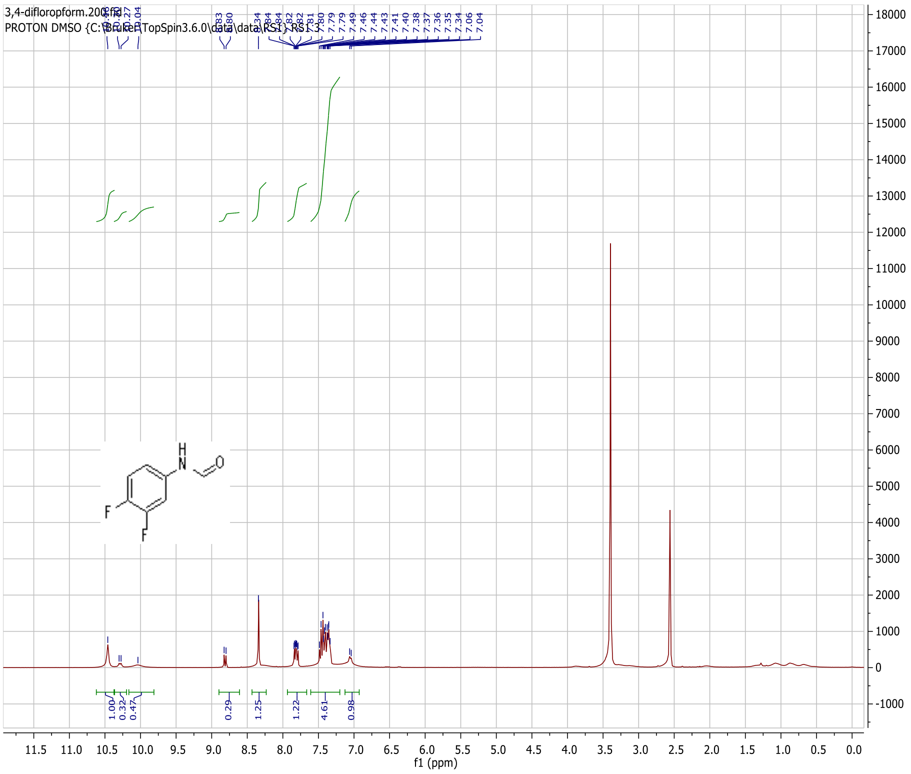

Figure S25.1 ^{1}H NMR (400 MHz, $CDCl_3$) spectrum

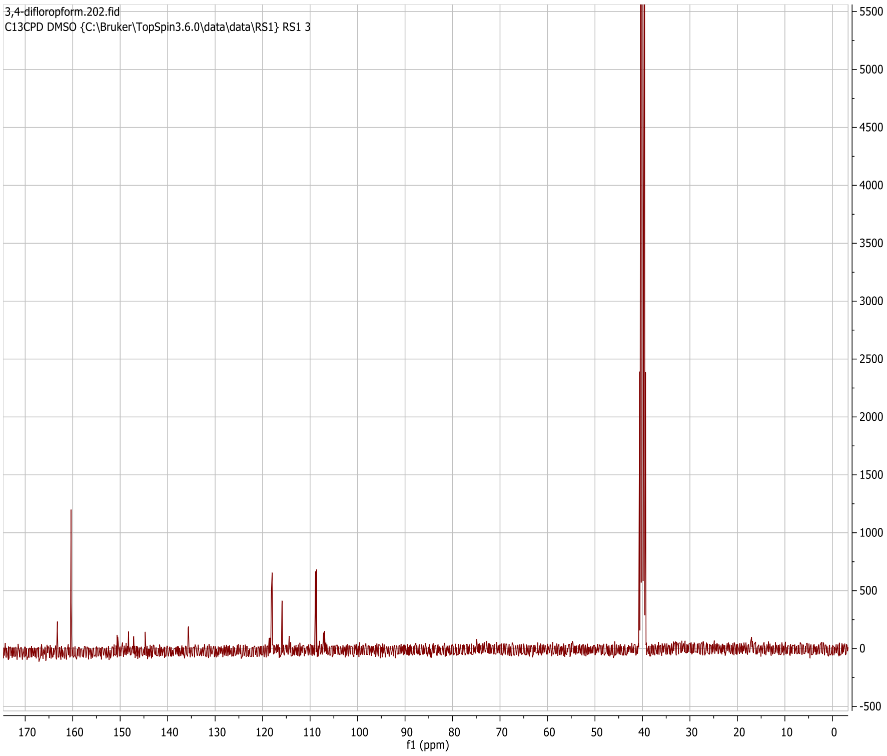

Figure S25.2. ^{13}C NMR (101 MHz, $CDCl_3$) spectrum

2.26. *N*-(3,4-dichlorophenyl)formamide

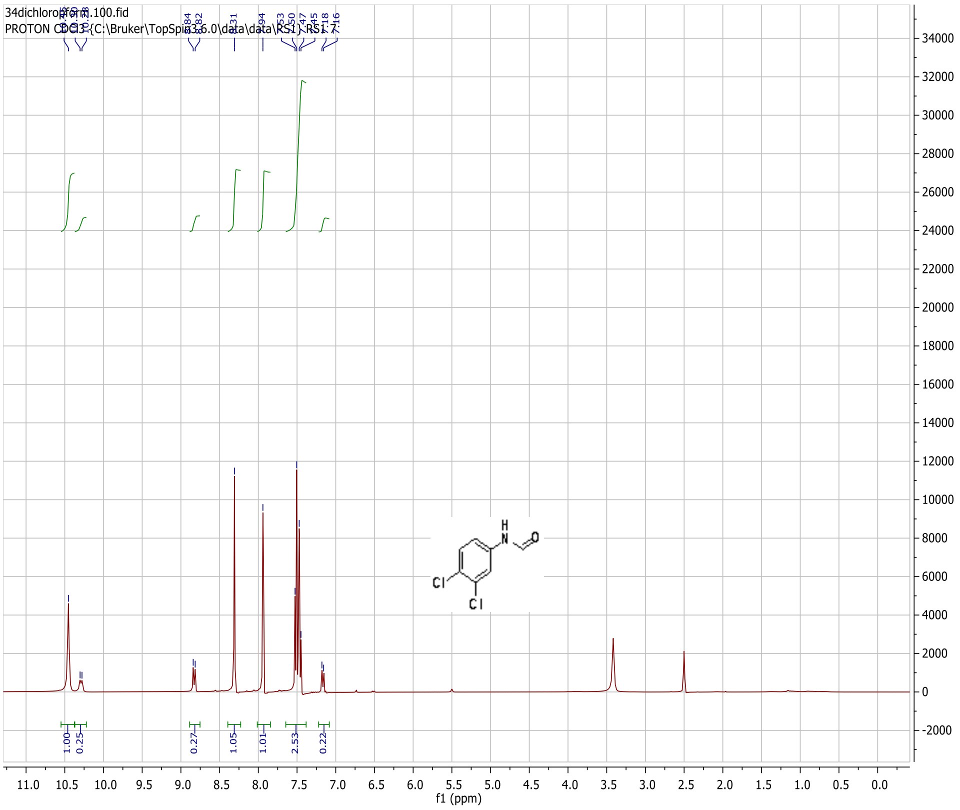

Figure S26.1 ^{1}H NMR (400 MHz, DMSO-d_6) spectrum

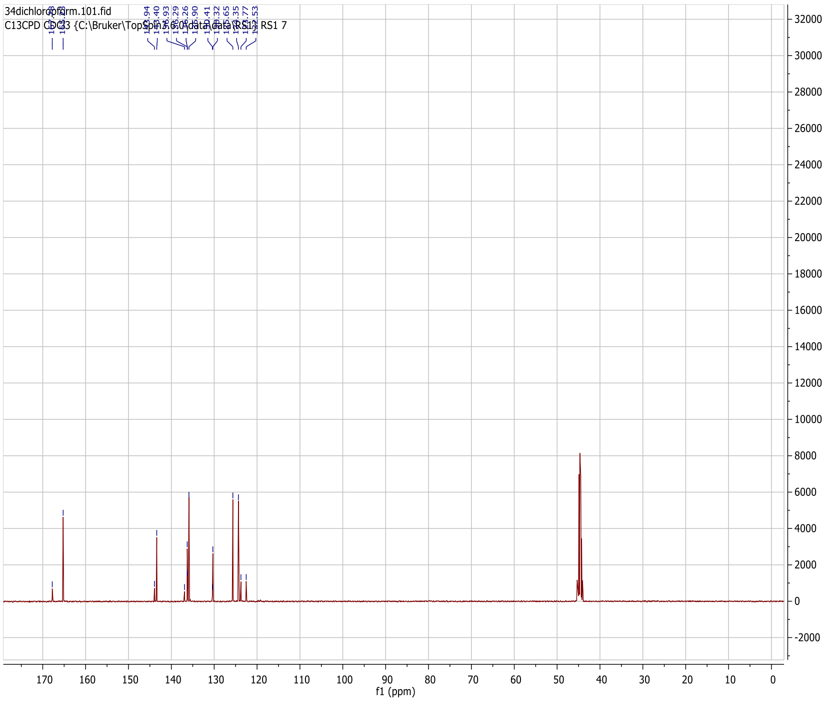

Figure S26.2 ^{13}C NMR (101 MHz, DMSO-d_6) spectrum

2.27. *N*-(3-chlorobenzyl)formamide

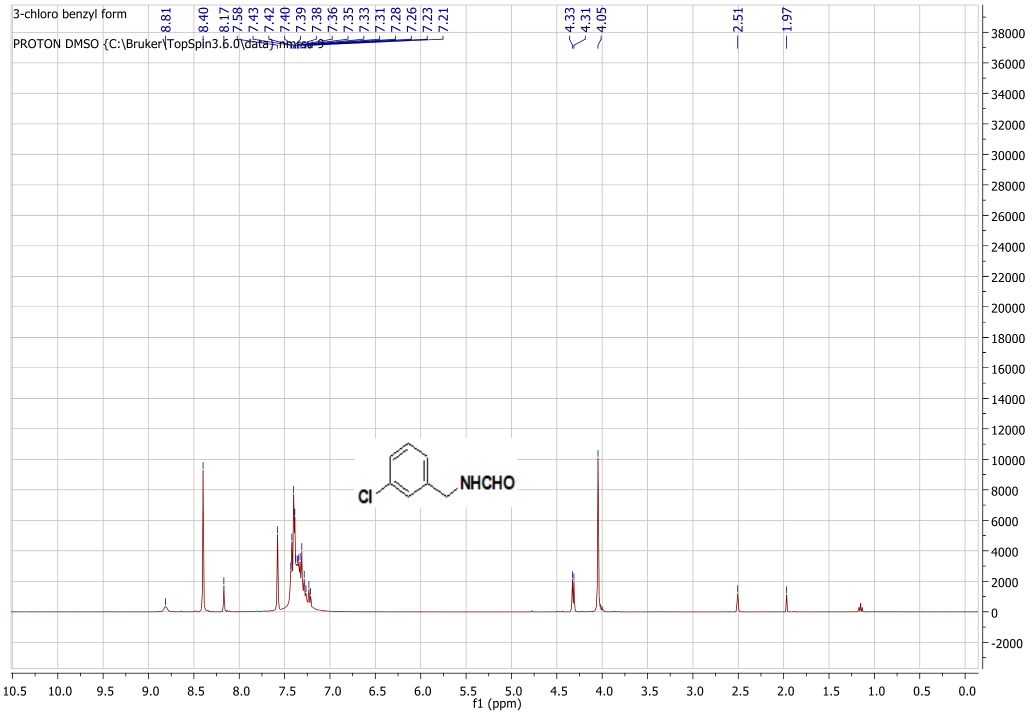

Figure S27.1 ^{1}H NMR (400 MHz, DMSO-d_6) spectrum

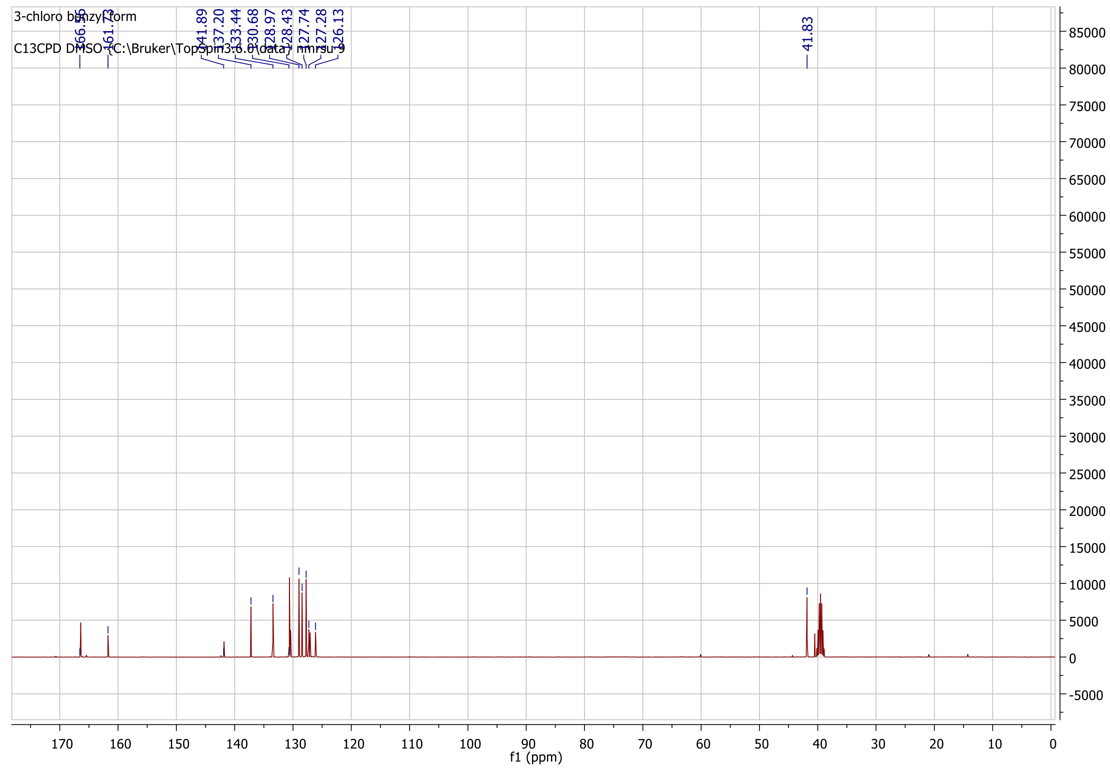

Figure S27.2. ^{13}C NMR (101 MHz, DMSO-d_6) spectrum

2.28. *N*-[(2-Methoxyphenyl)methyl]formamide

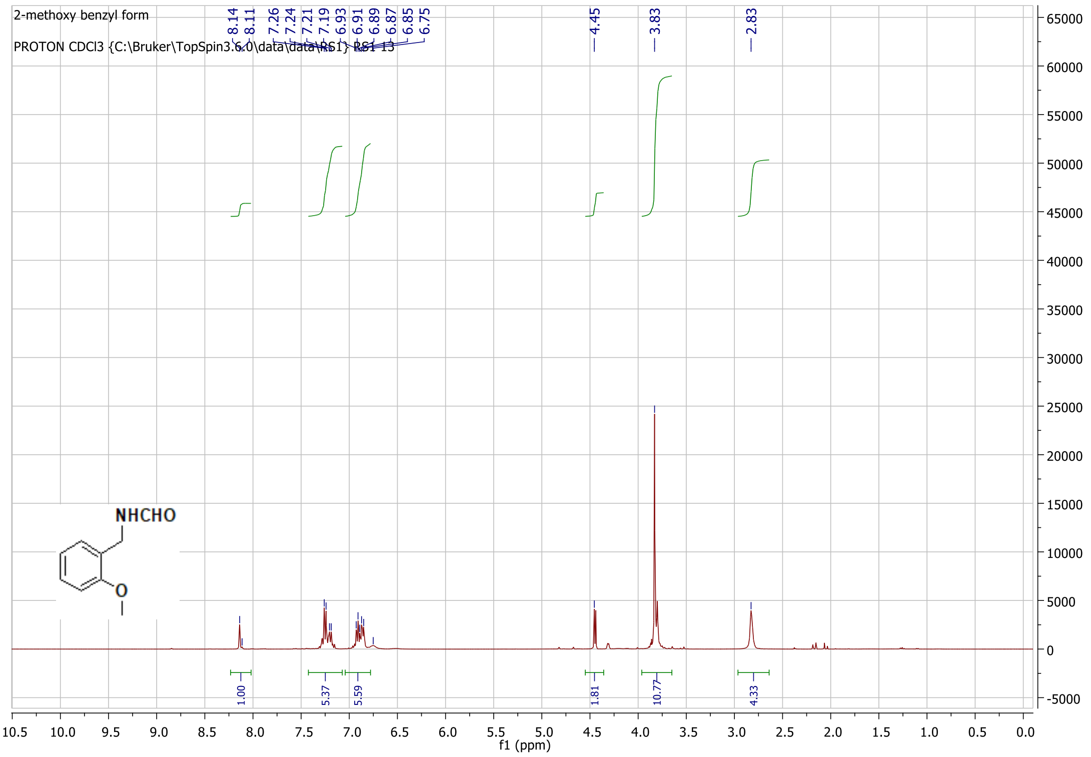

Figure S28.1 ^{1}H NMR (400 MHz, $CDCl_3$) spectrum

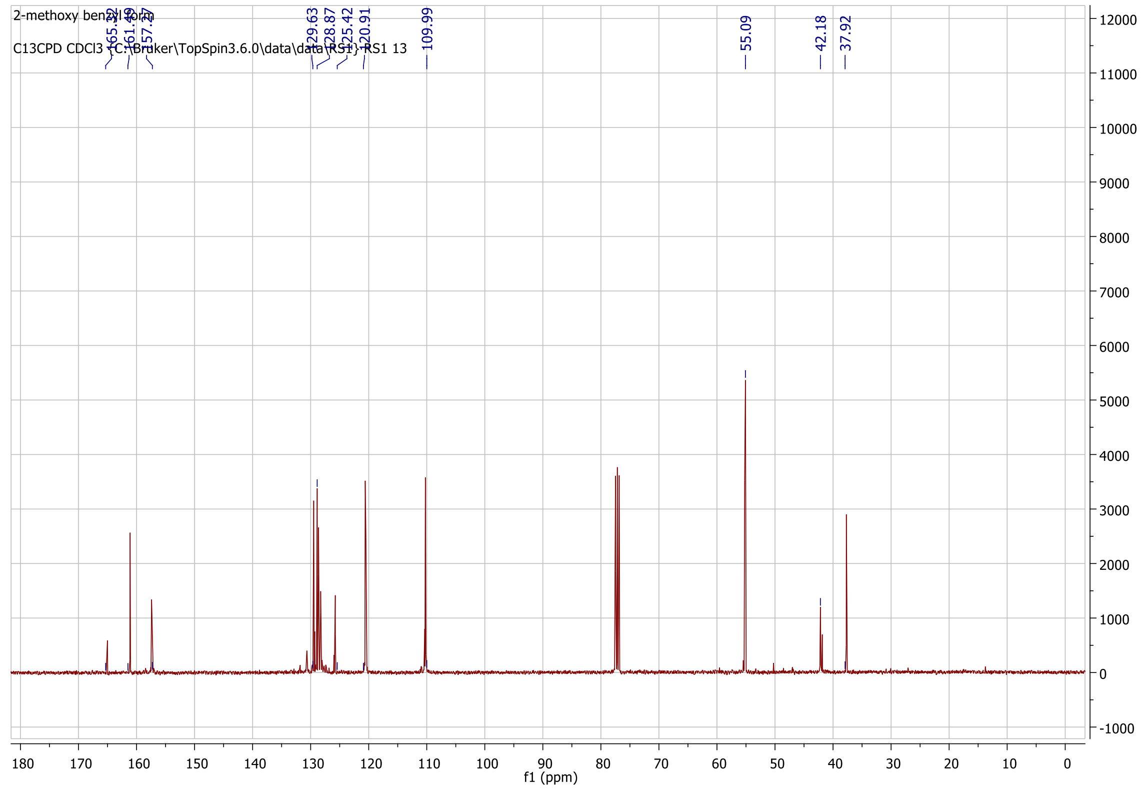

Figure S28.2 ^{13}C NMR (101 MHz, $CDCl_3$) spectrum

2.29. *N*-(4-methoxybenzyl)formamide

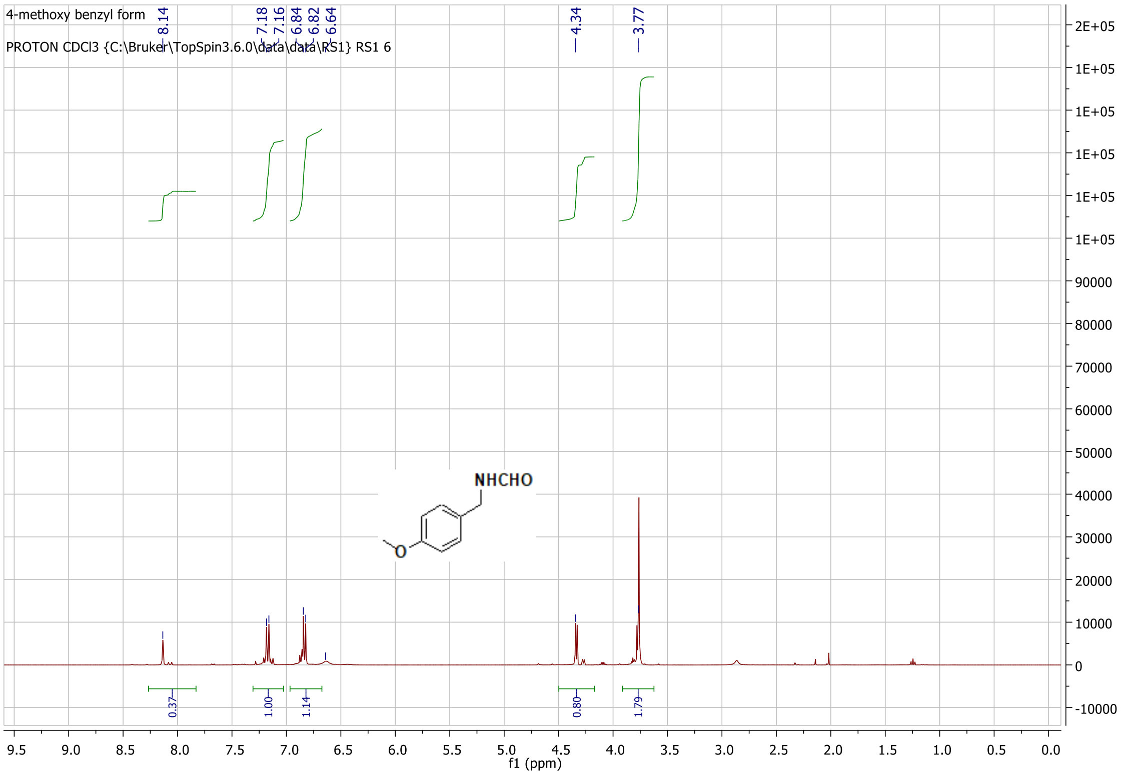

Figure S29.1 ^{1}H NMR (400 MHz, $CDCl_3$) spectrum

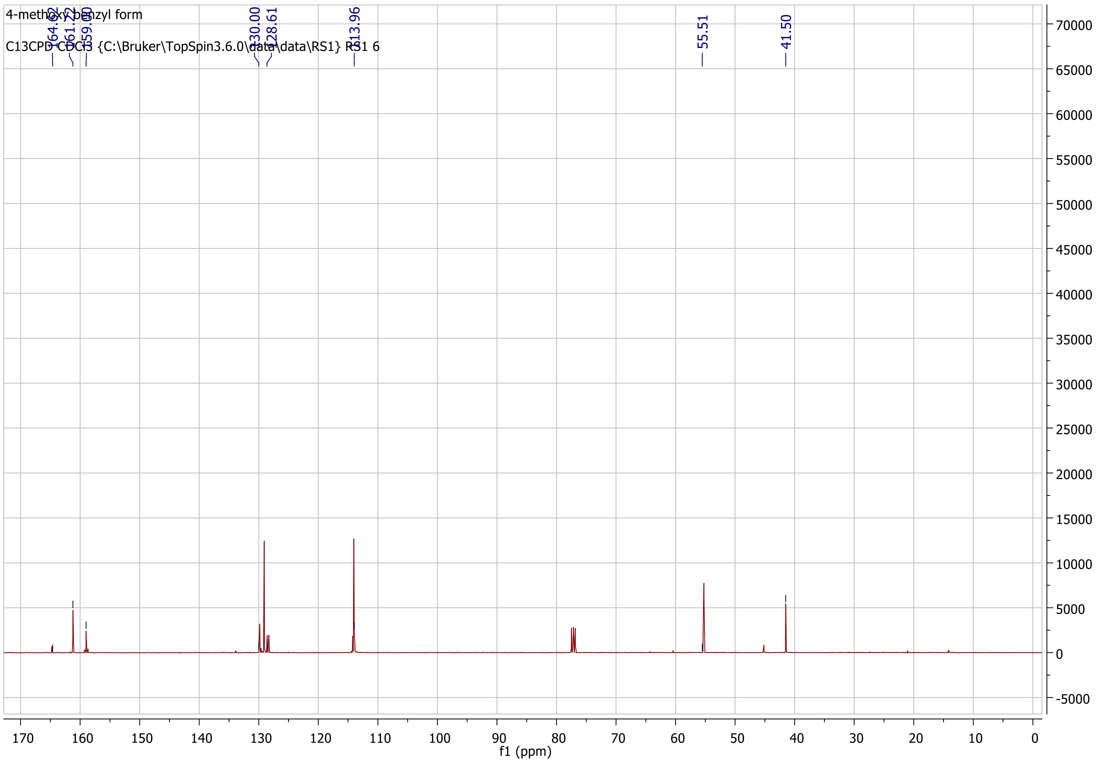

Figure S29.2. ^{13}C NMR (101 MHz, $CDCl_3$) spectrum

2.30. *N*-(4-methoxyphenyl)formamide

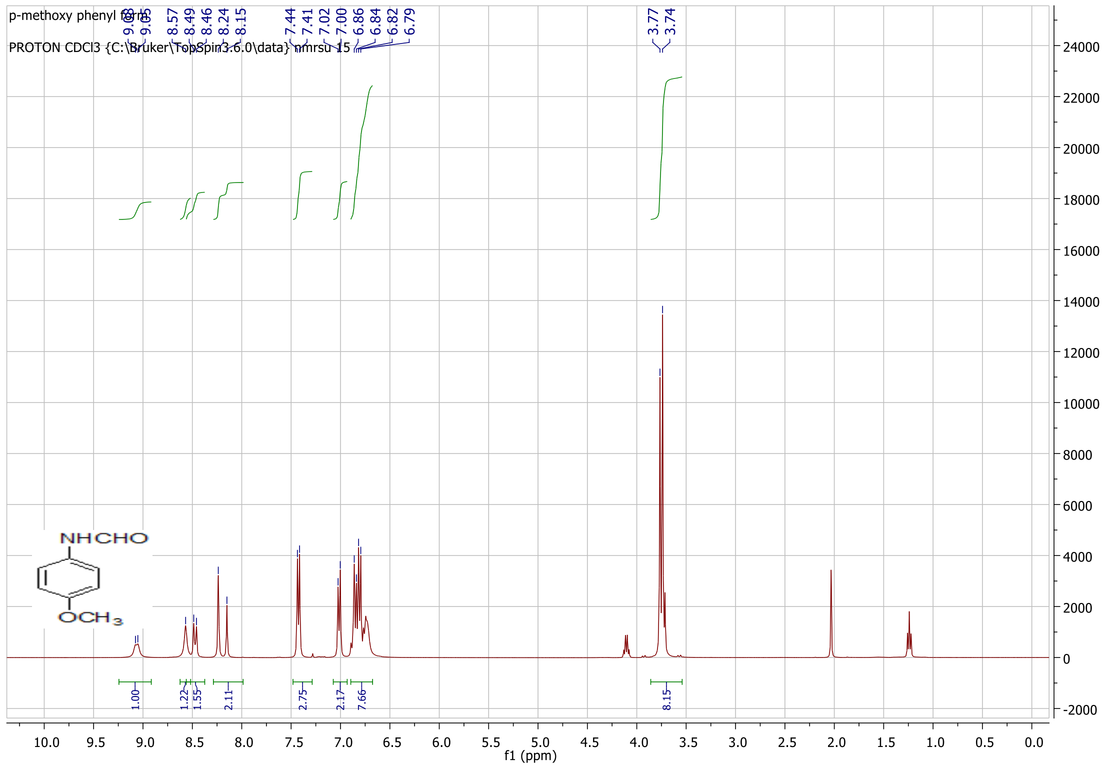

Figure S30.1 ^{1}H NMR (400 MHz, $CDCl_3$) spectrum

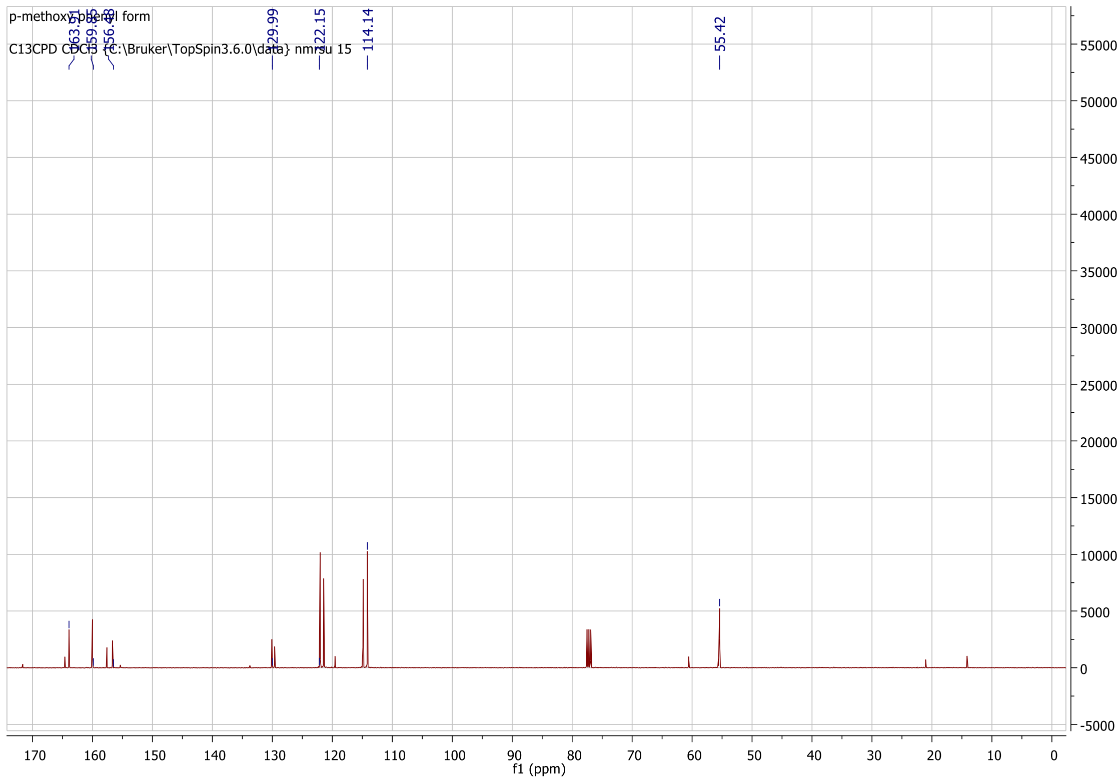

Figure S30.2 ^{13}C NMR (101 MHz, $CDCl_3$) spectrum

2.31. *N*-(3-cyanophenyl)formamide

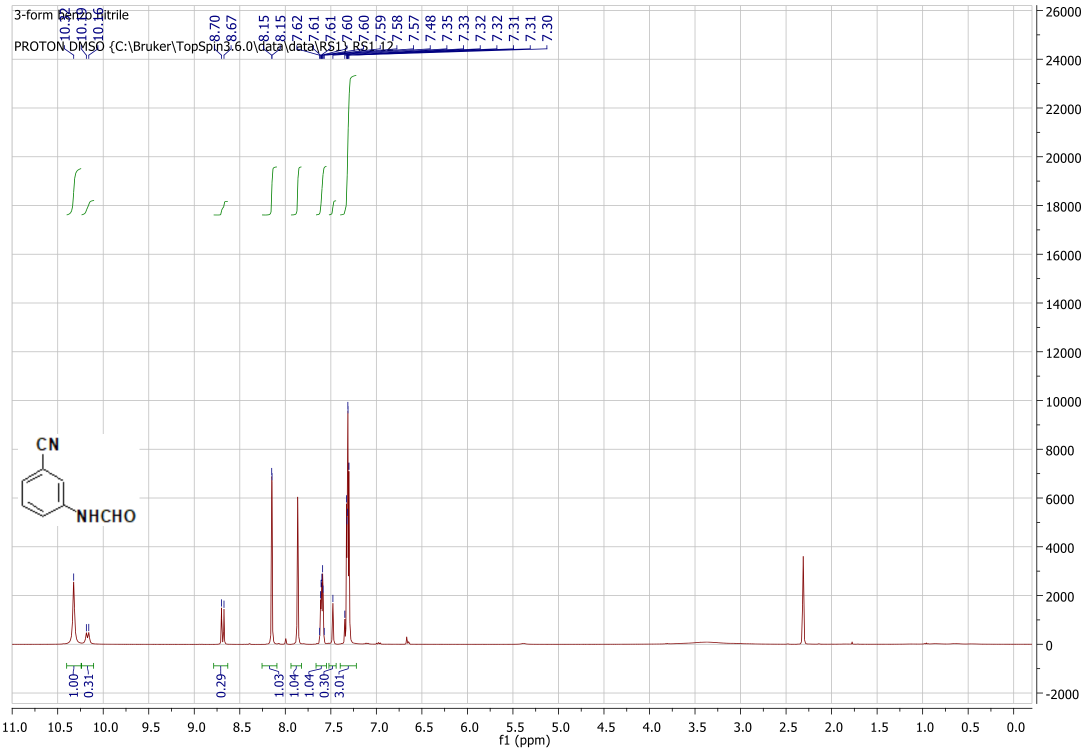

Figure S31.1 ^{1}H NMR (400 MHz, DMSO-d_6) spectrum

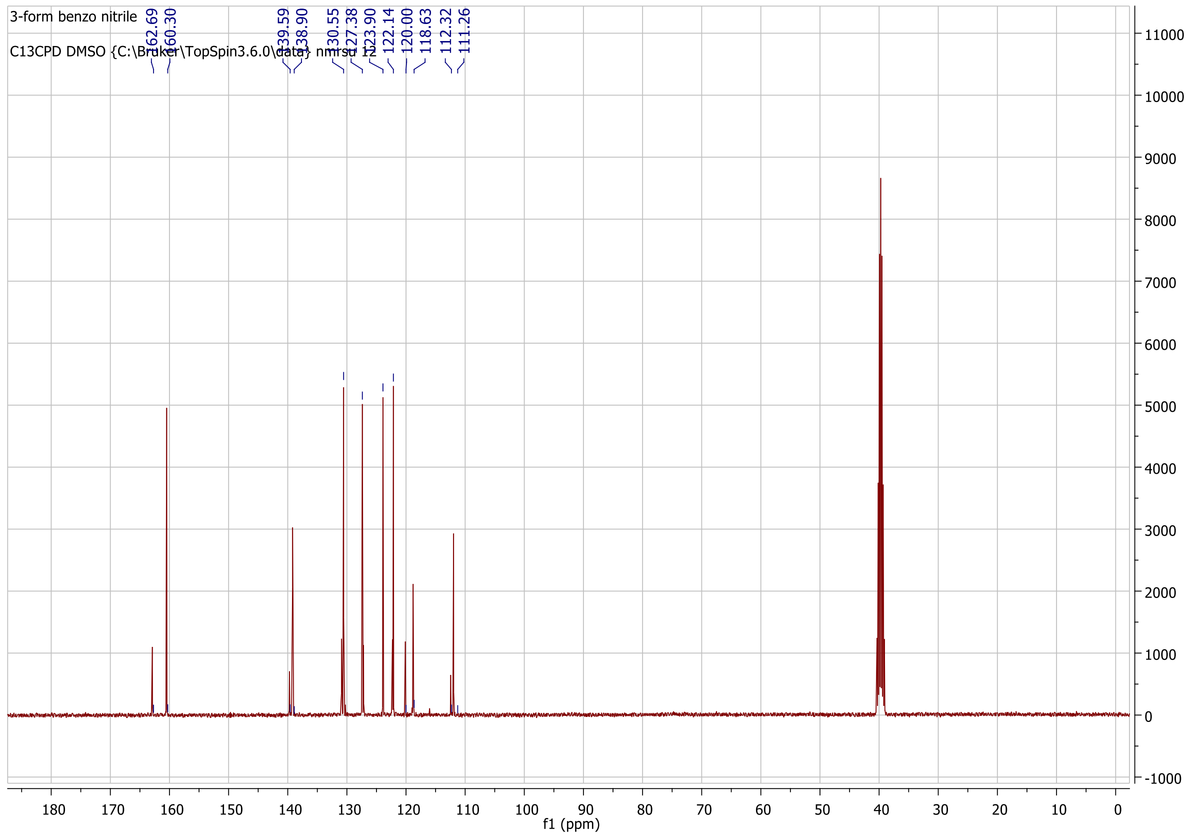

Figure S30.2. ^{13}C NMR (101 MHz, DMSO-d_6) spectrum

2.32. *N*-(2-formamidophenyl)formamide

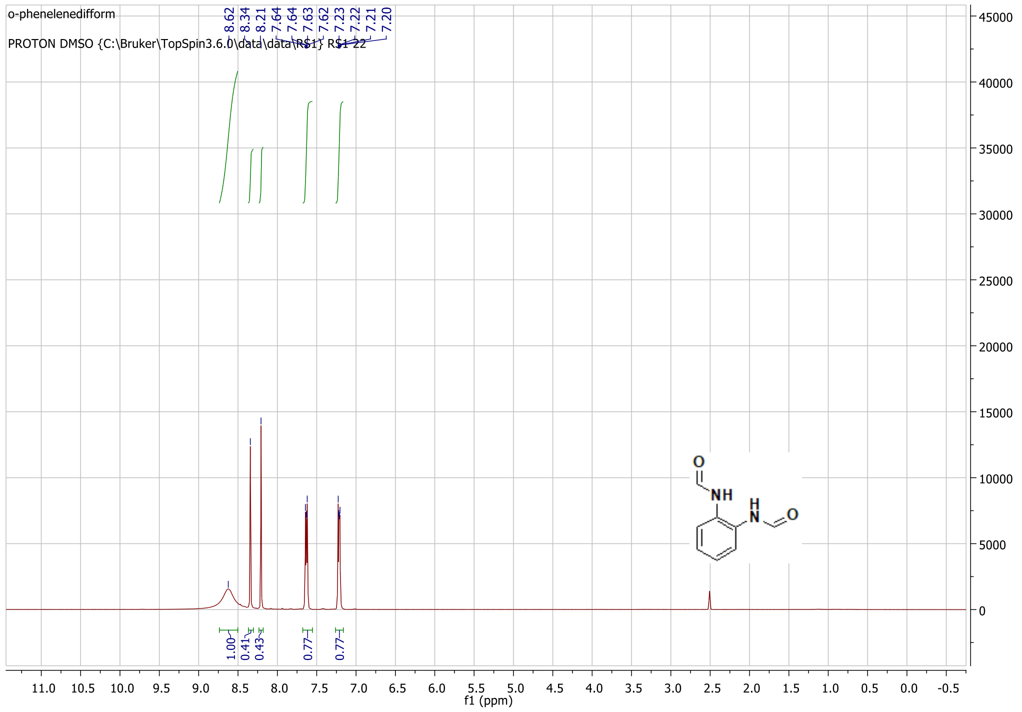

Figure S32.1. ^{1}H NMR (400 MHz, (DMSO-d_6) spectrum

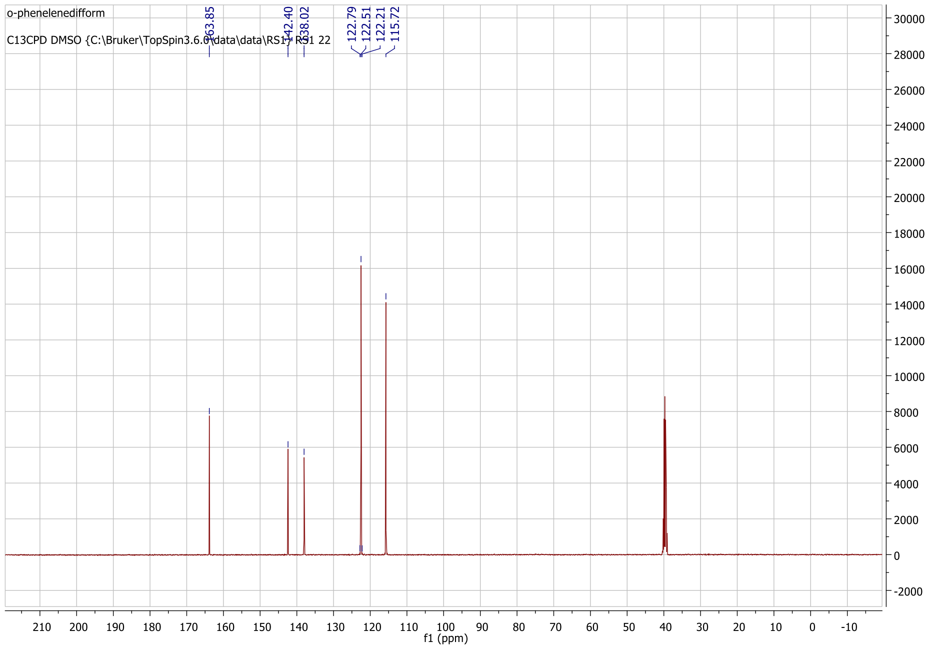

Figure S32.2 ^{13}C NMR spectrum (101 MHz, (DMSO-d_6)

2.33. *N*-(4-acetyl phenyl) formamide

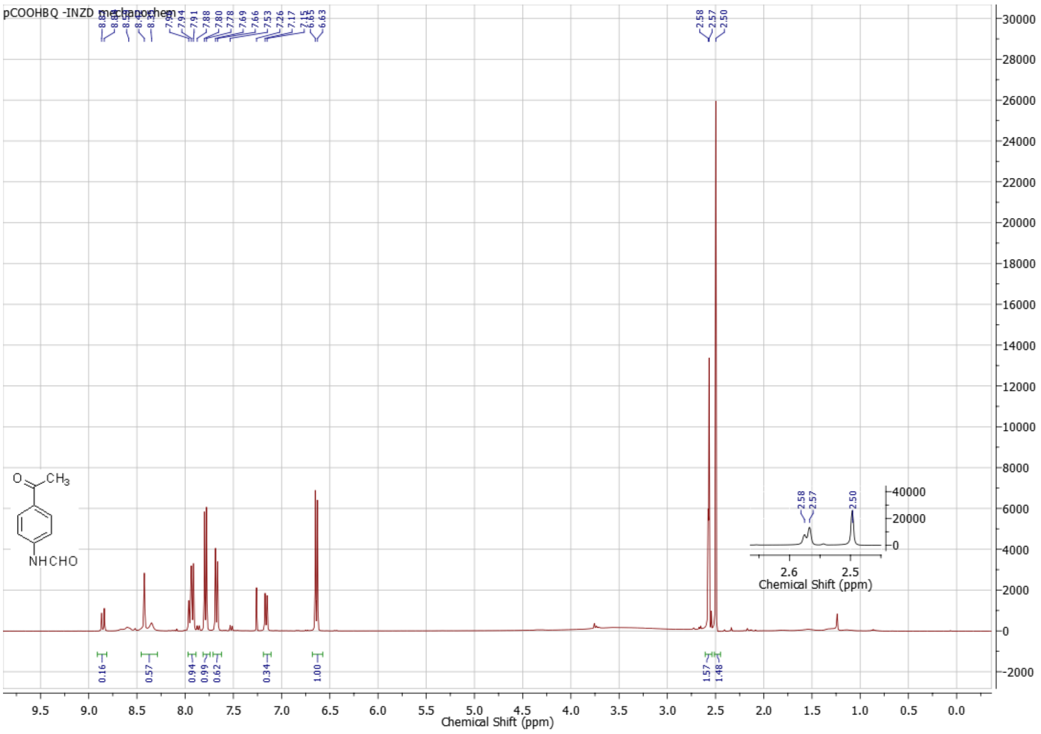

Figure S33.1 ^{1}H NMR (400 MHz, MeOD) spectrum

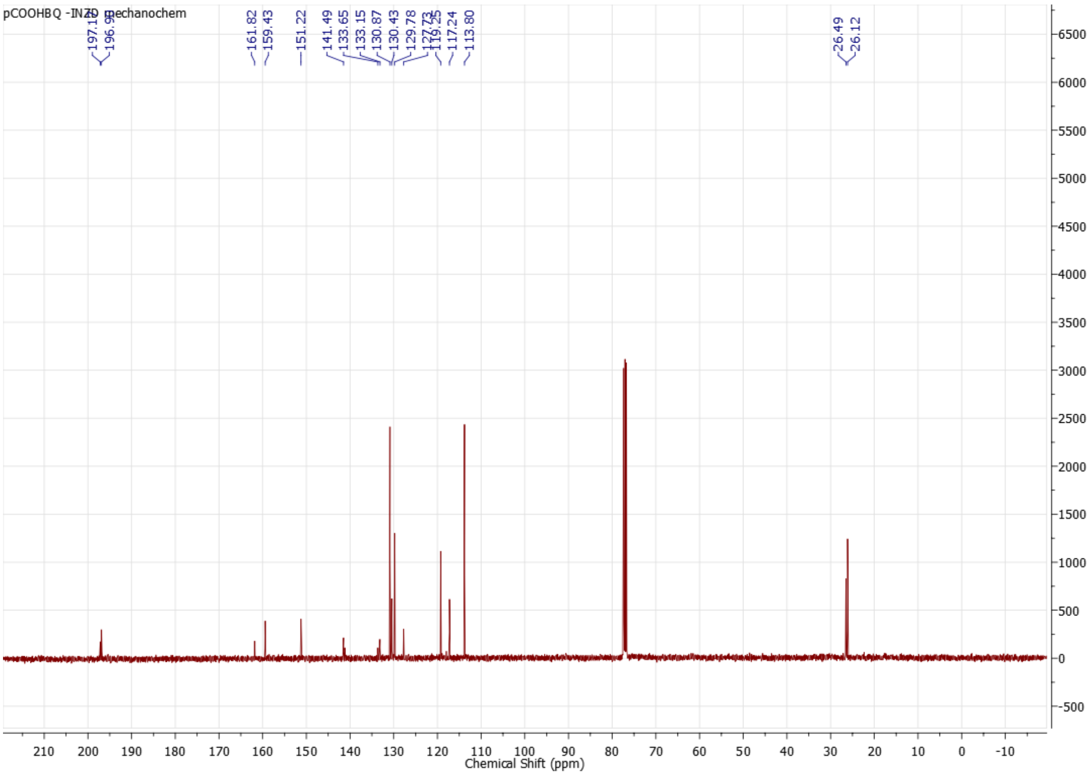

Figure S33.2. ^{13}C NMR (101 MHz, MeOD) spectrum

2.34. *N*-(cyclohexyl)formamide

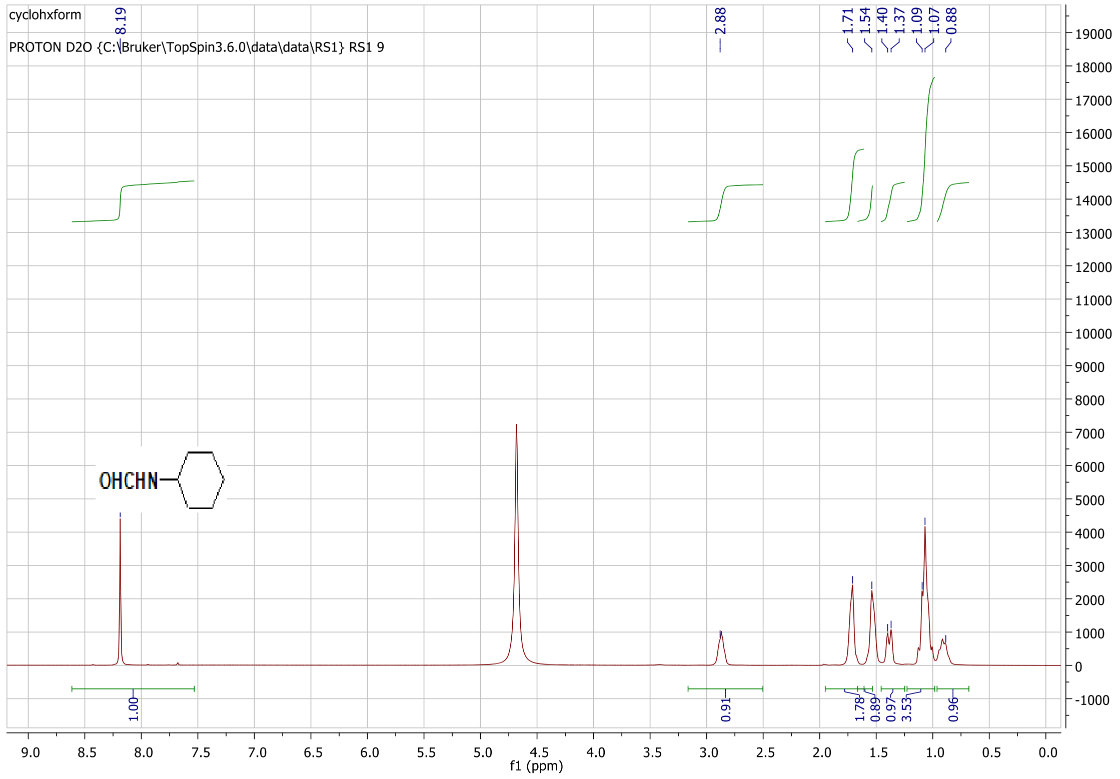

Figure S34.1 ^{1}H NMR (400 MHz, D_2O) spectrum

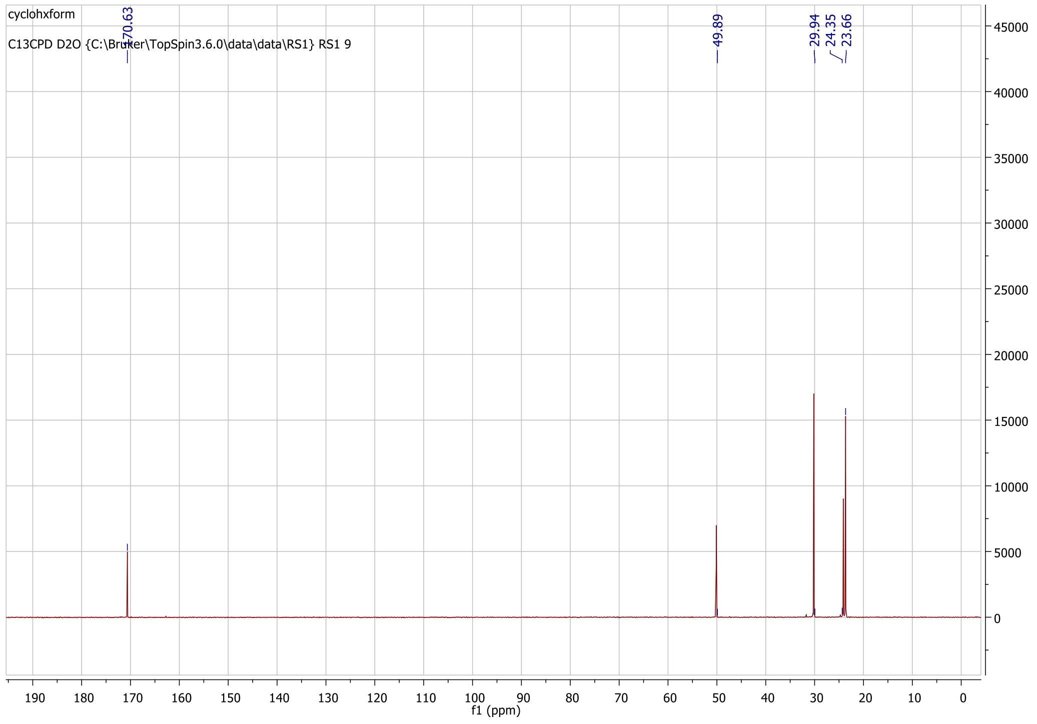

Figure S34.2. ^{13}C NMR (101 MHz, D_2O) spectrum

2.35. Ethyl 2-(formylamino)benzoate

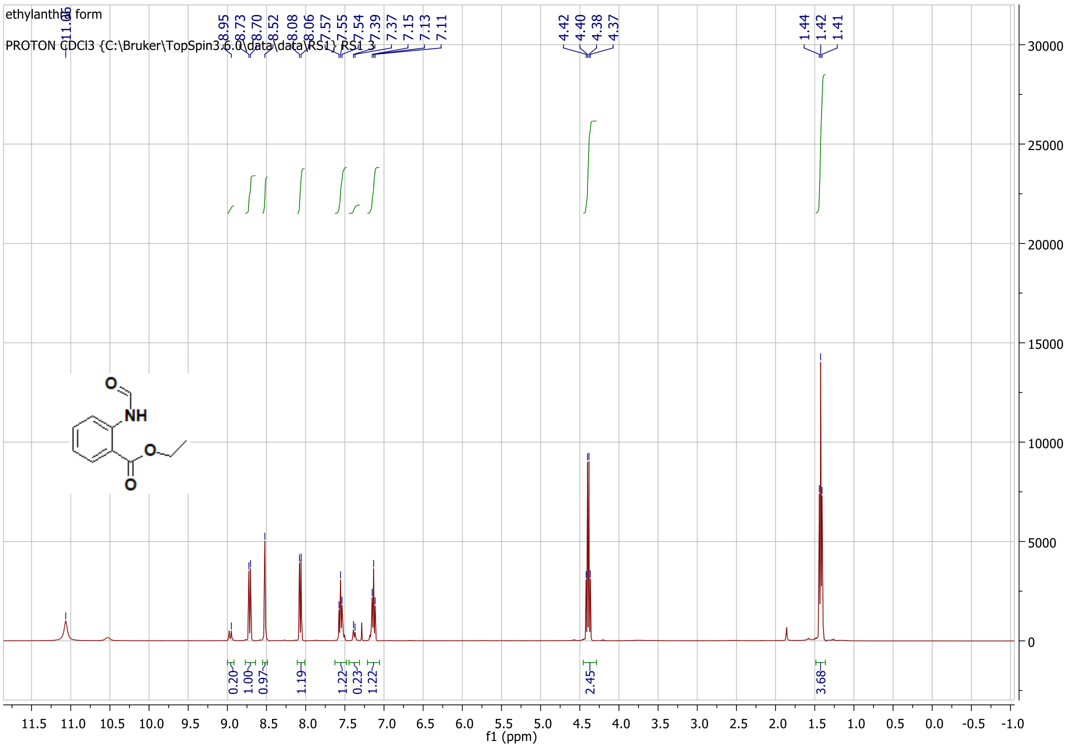

Figure S35.1 ^{1}H NMR (400 MHz, $CDCl_3$) spectrum

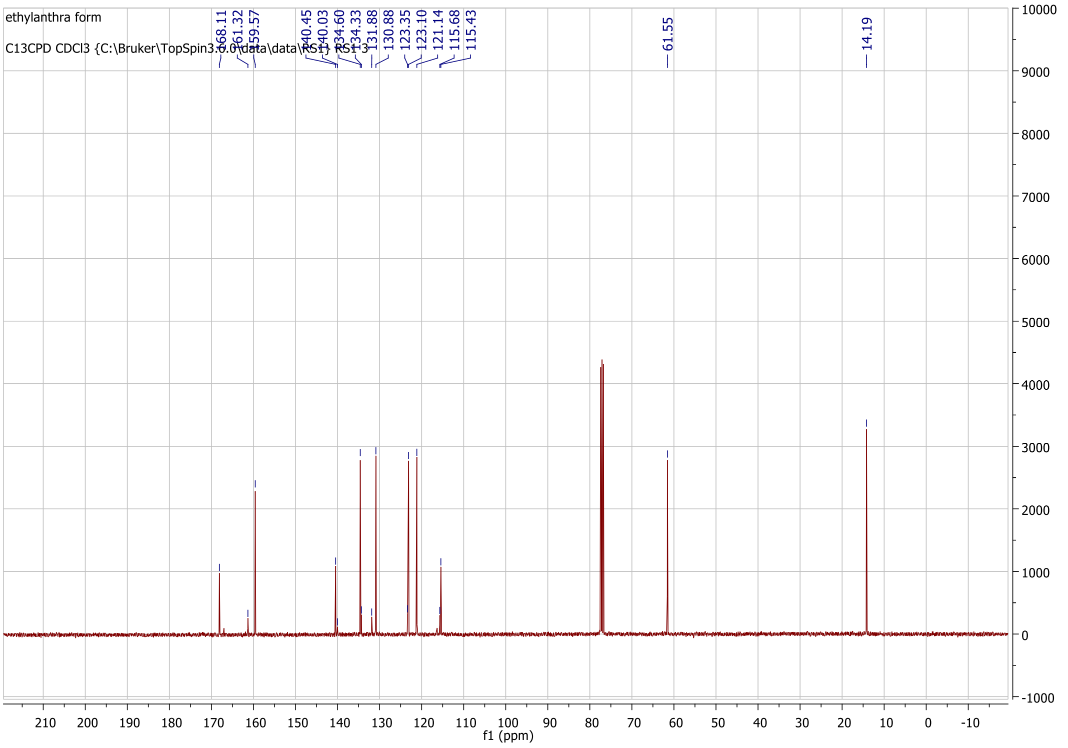

Figure S35.2 ^{13}C NMR (101 MHz, $CDCl_3$) spectrum

2.36. *N*-(naphthalen-1-yl)formamide

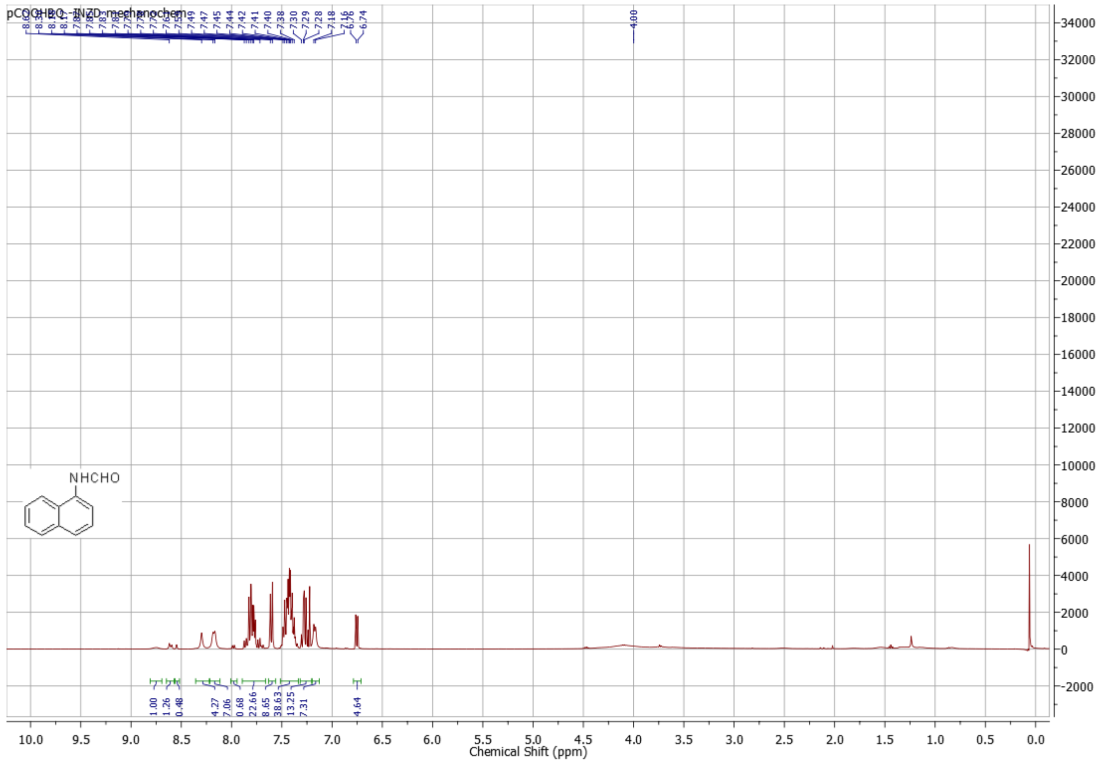

Figure S36.1. ^{1}H NMR (400 MHz, $CDCl_3$) spectrum

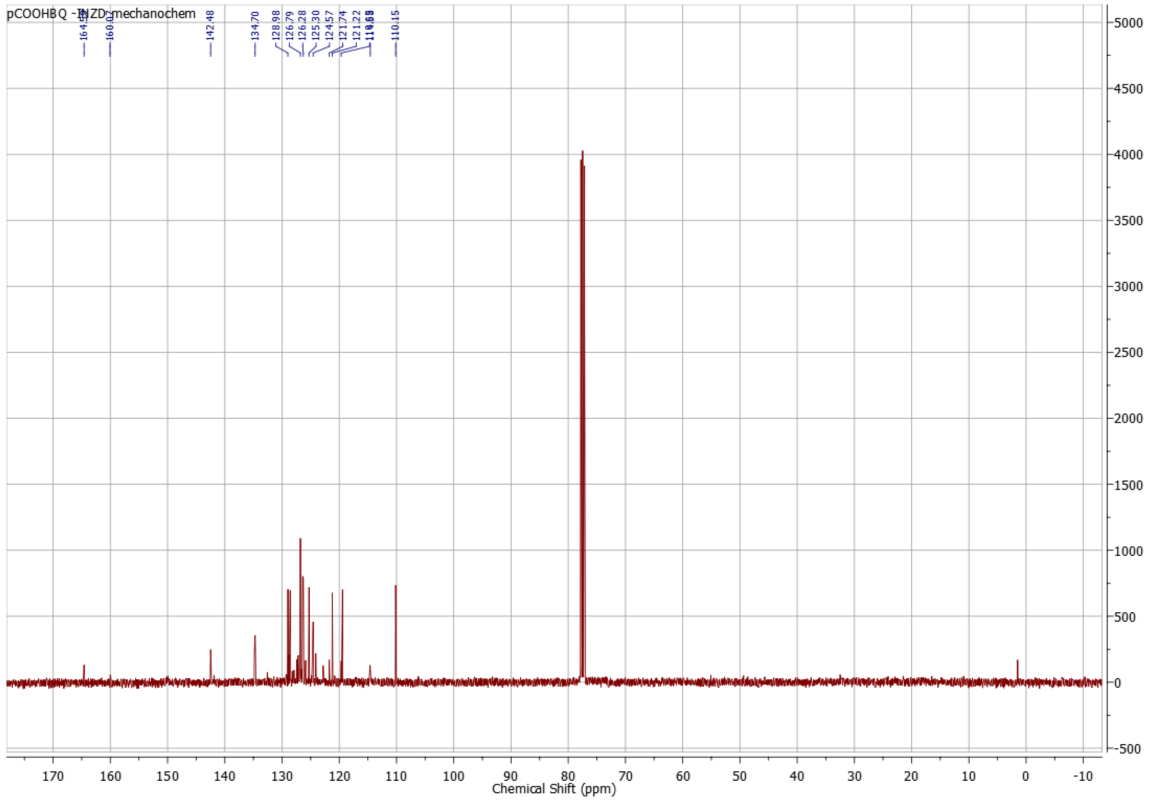

Figure S36.2. ^{13}C NMR (101 MHz, $CDCl_3$) spectrum

2.37. *N*-(5-formamidonaphthalen-1-yl)formamide

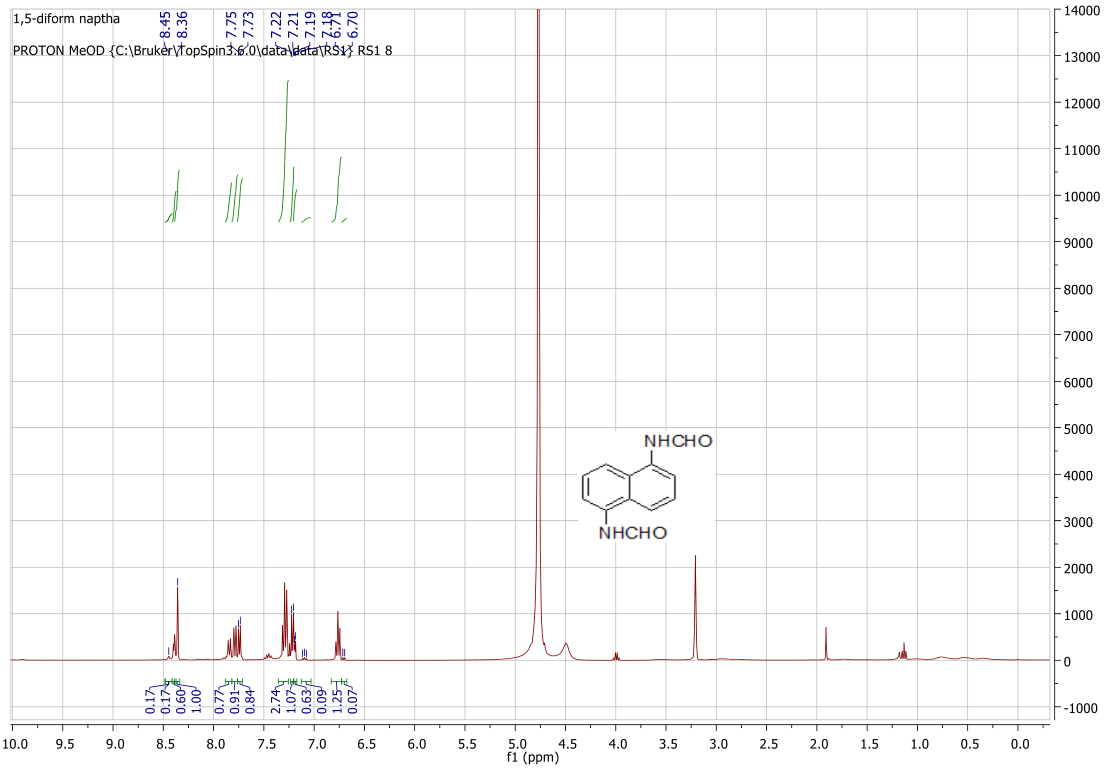

Figure S37.1 ^{1}H NMR (400 MHz, MeOD) spectrum

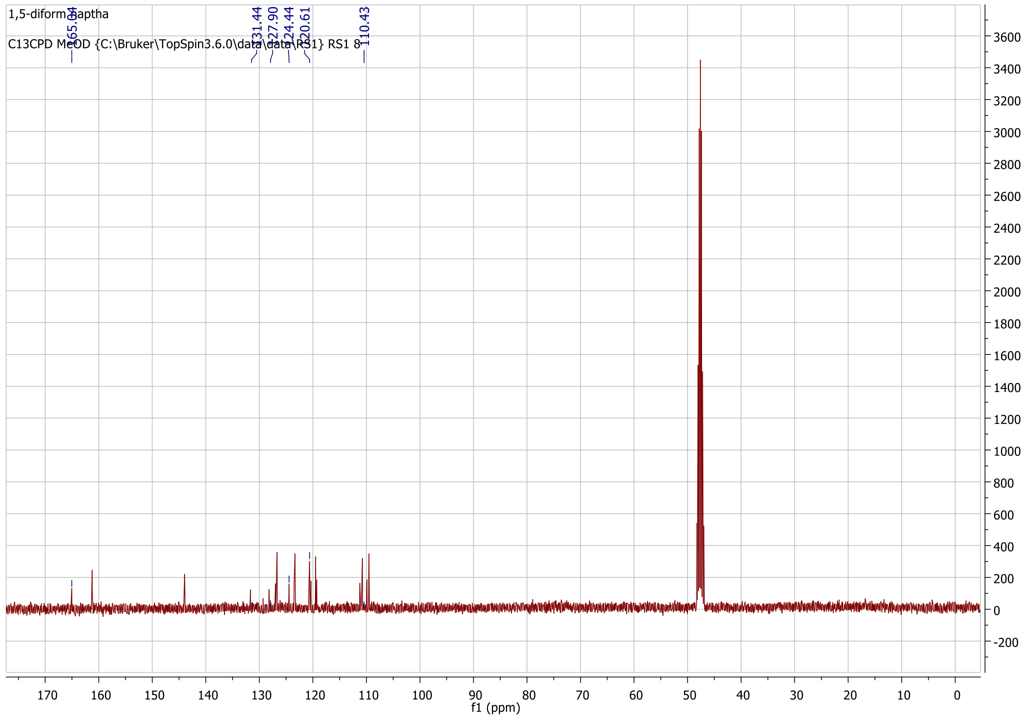

Figure S37.2 ^{13}C NMR (101 MHz, MeOD) spectrum

2.38. *N*-(8-formamidonaphthalen-1-yl)formamide

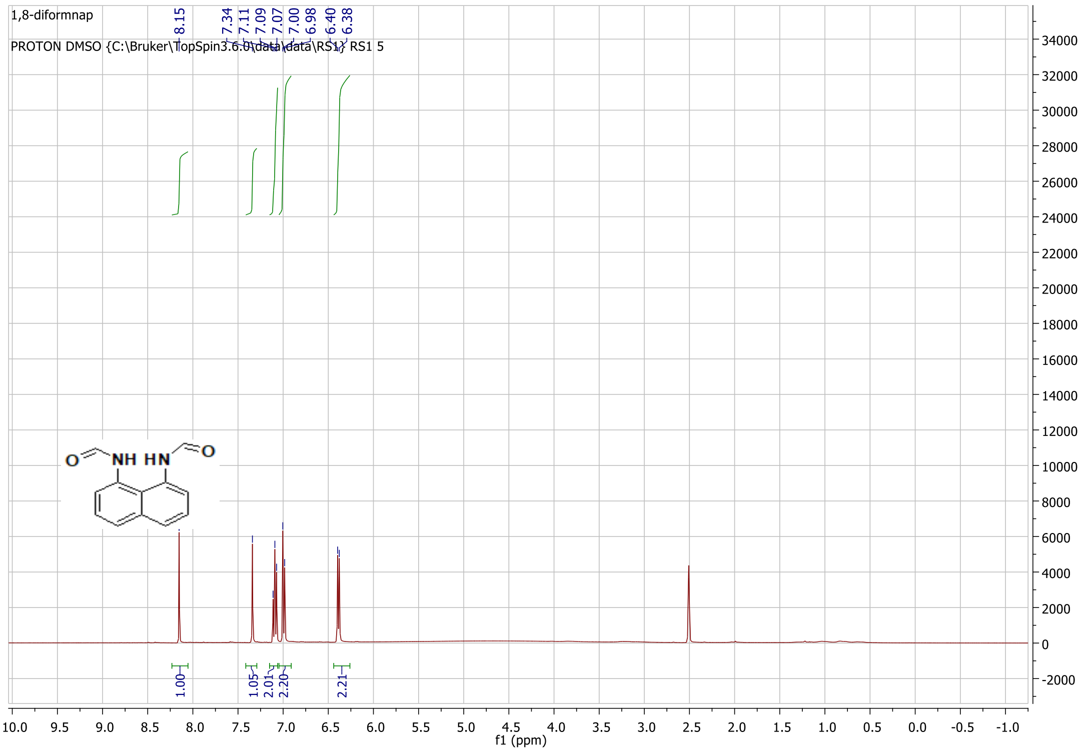

Figure S38.1. ^{1}H NMR (400 MHz, DMSO-d_6) spectrum

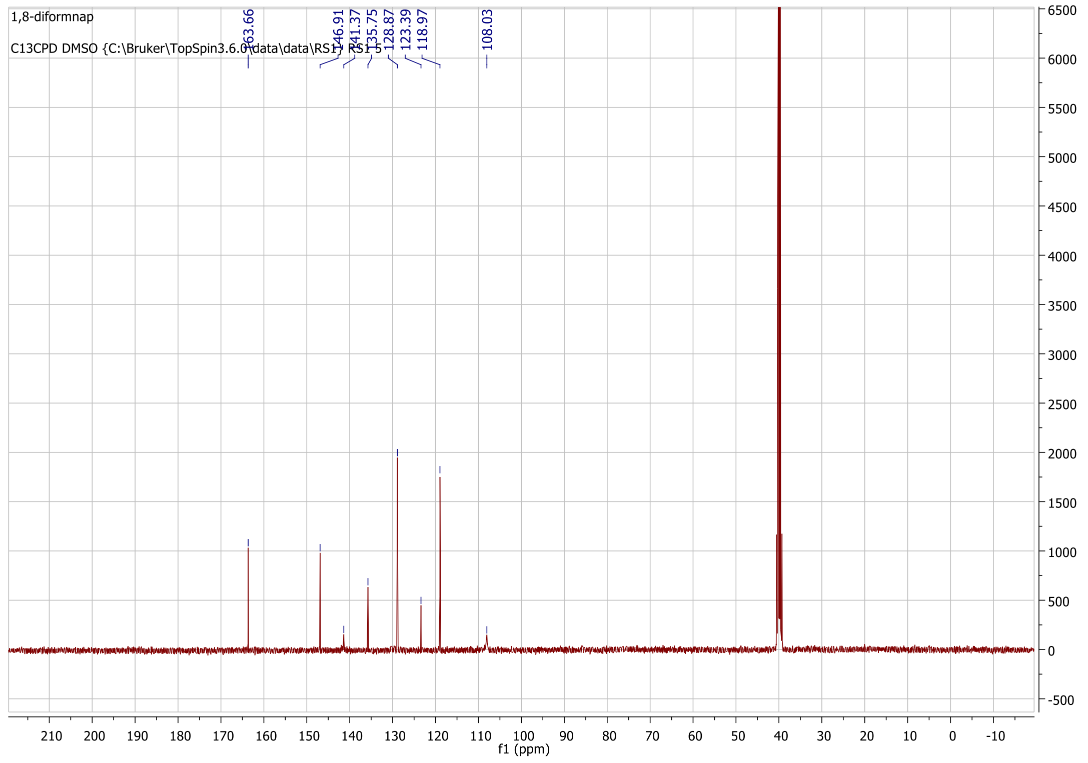

Figure S38.2. ^{13}C NMR (101 MHz, DMSO-d_6) spectrum

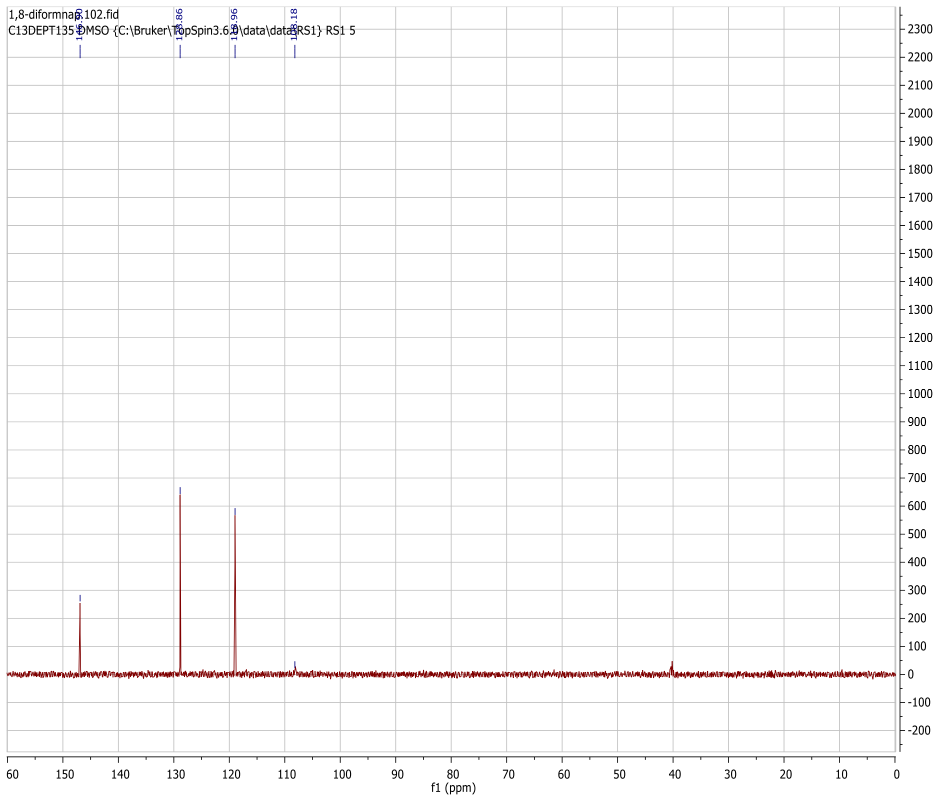

Figure S38.3. ^{13}C DEPT. (102 MHz, DMSO-d_6) spectrum

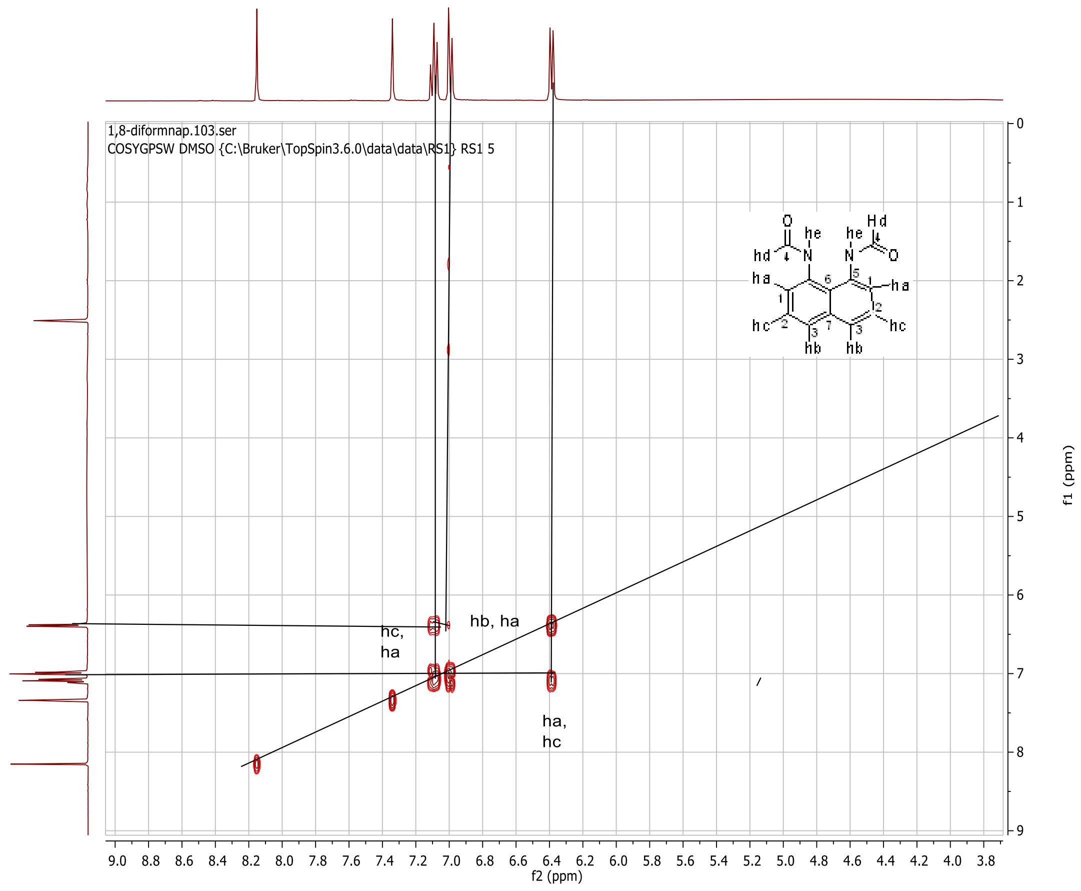

Figure S38.4. COSY (103 MHz, DMSO) spectrum

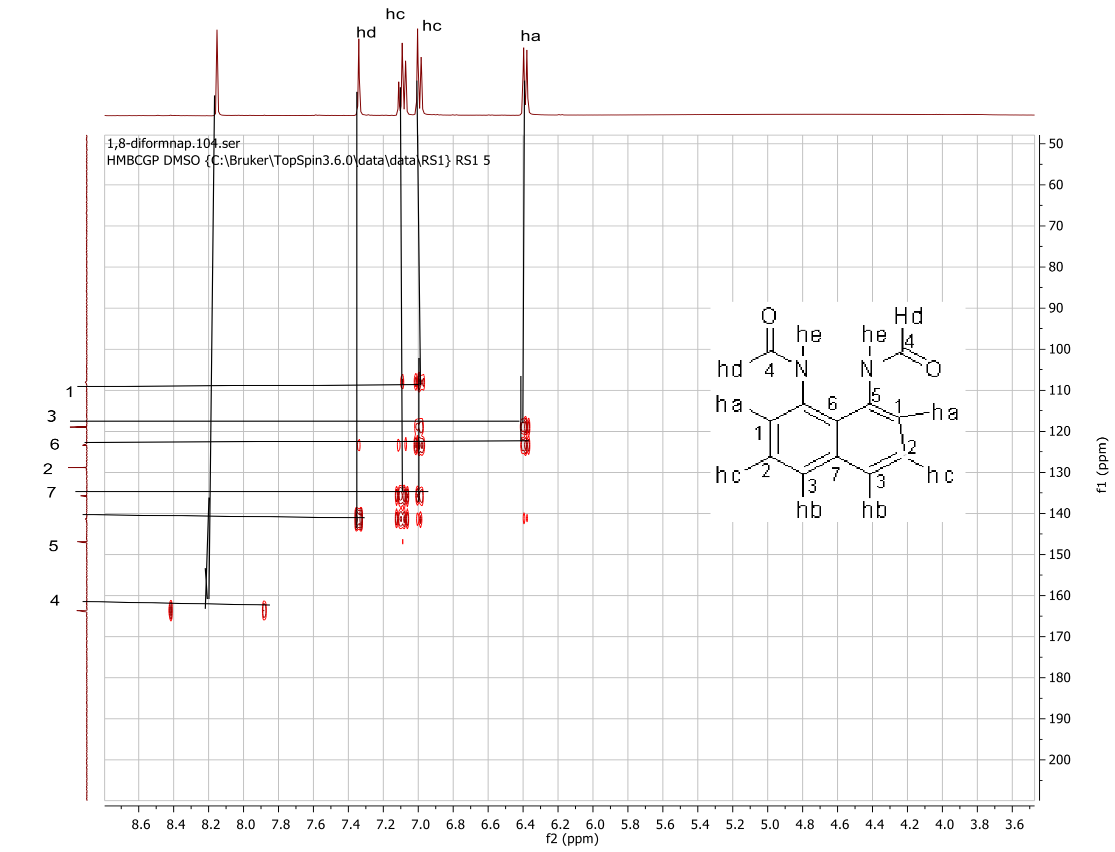

Figure S38.5. HMBC (105 MHz, DMSO-d_6) spectrum

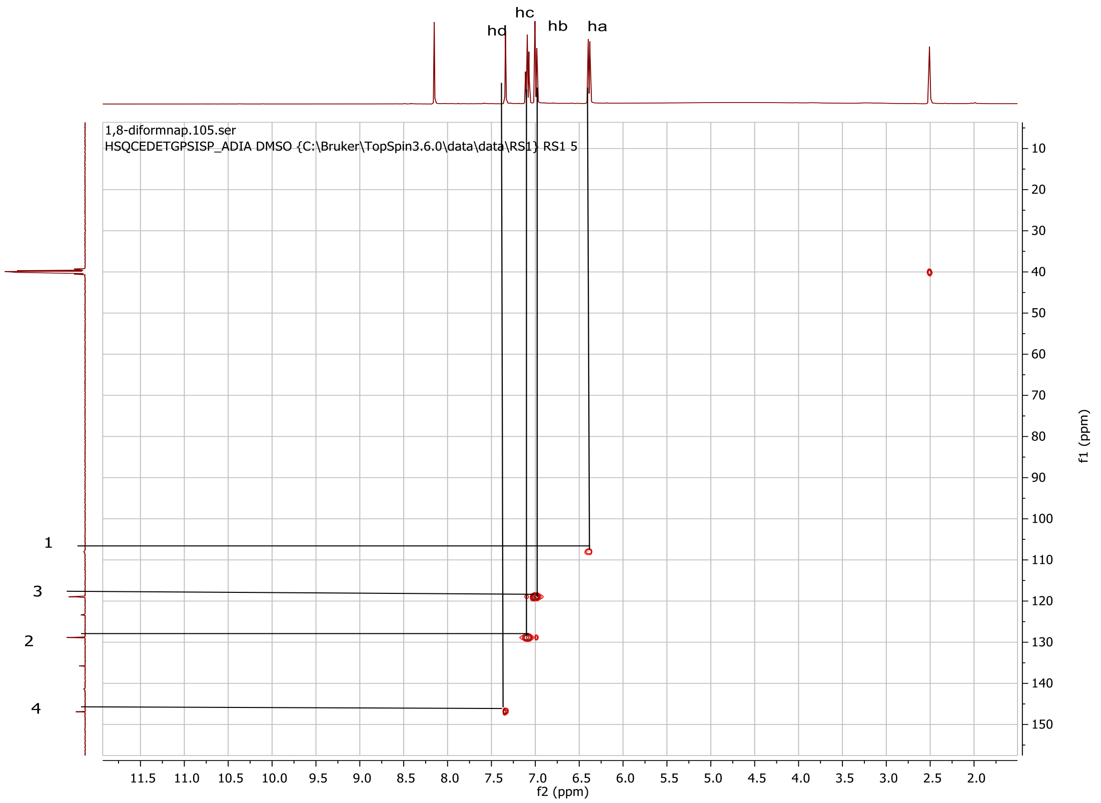

Figure S38.6. HSQC (106 MHz, DMSO-d_6) spectrum

2.39. 2-Formamido anthraquinone

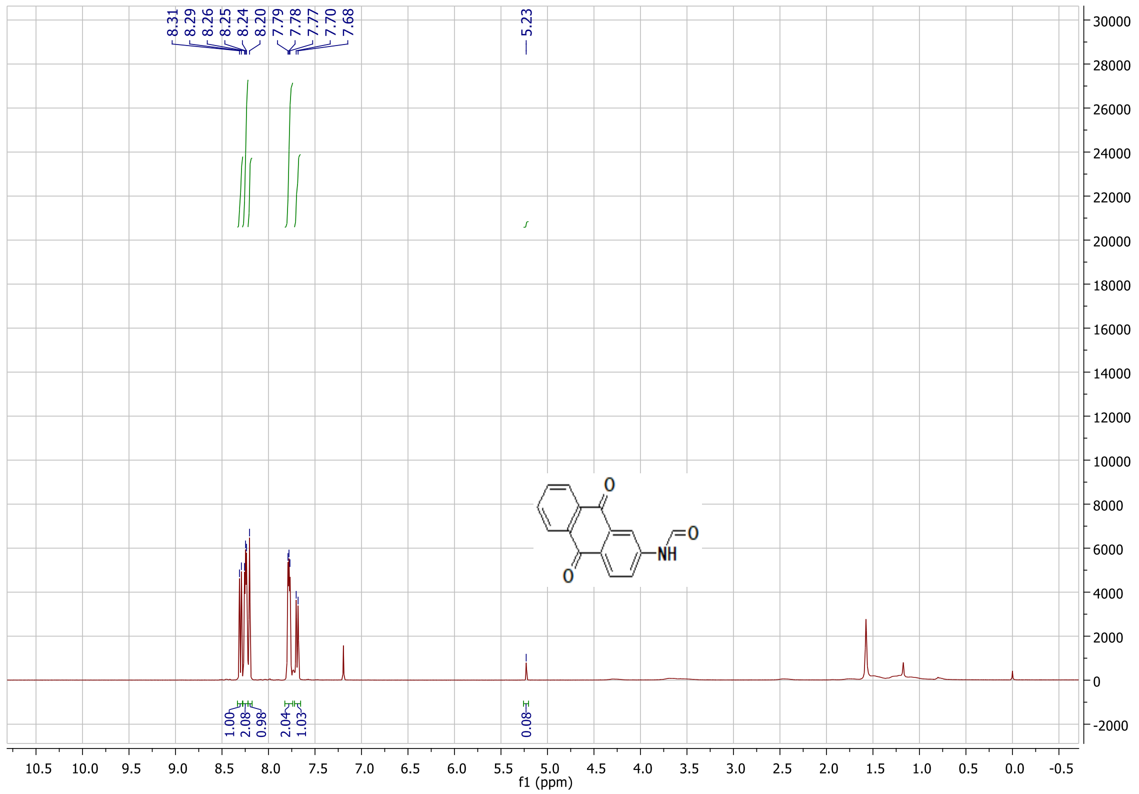

Figure S39.1 ^{1}H NMR (400 MHz, DMSO-d_6) spectrum

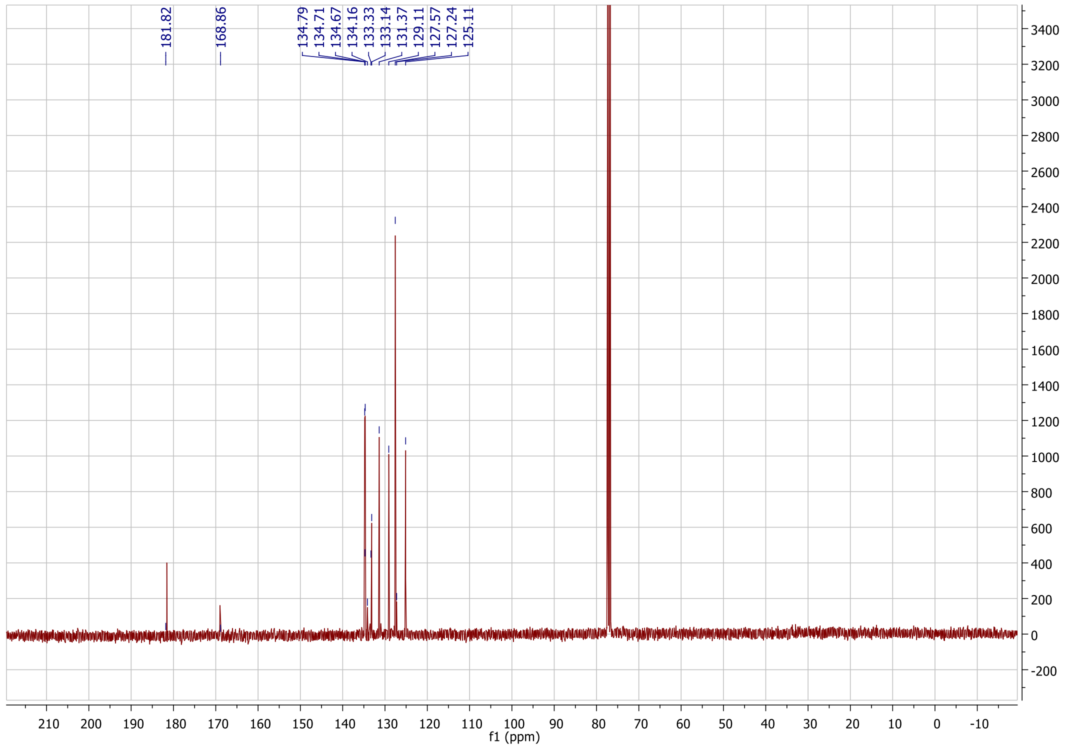

Figure S39.2. ^{13}C NMR (101 MHz, DMSO-d_6) spectrum

2.40. *N*-(5-methylisoxazol-3-yl) formamide

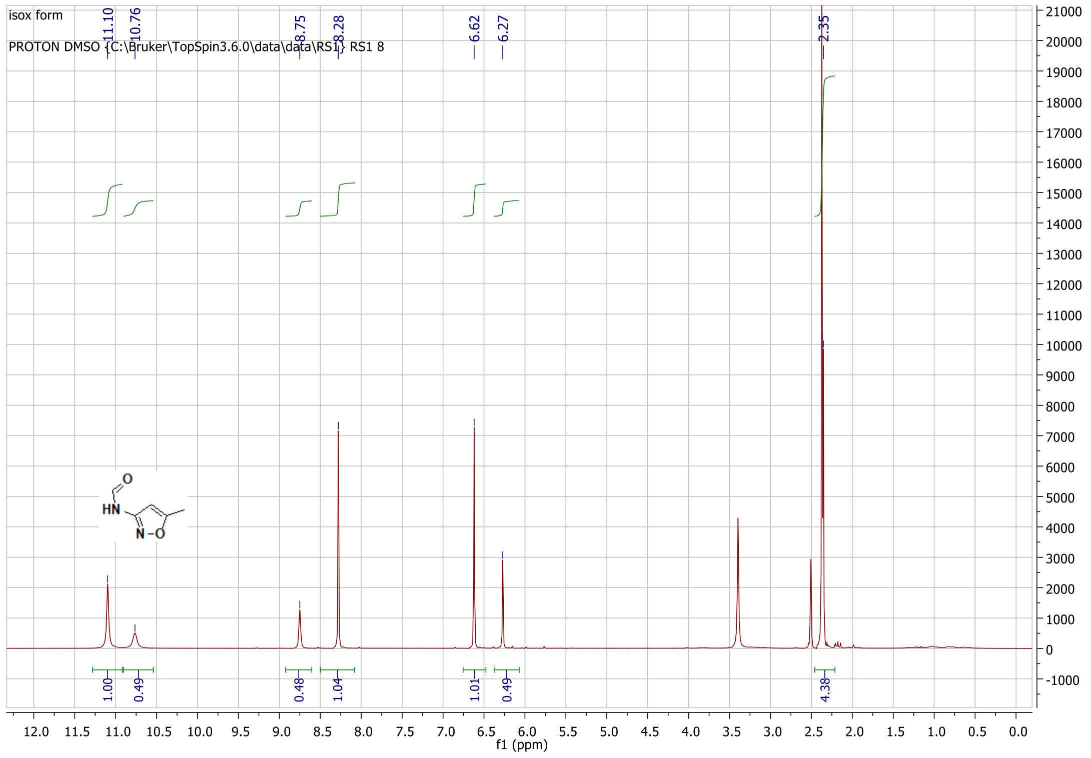

Figure S40.1 ^{1}H NMR (400 MHz, MeOD) spectrum

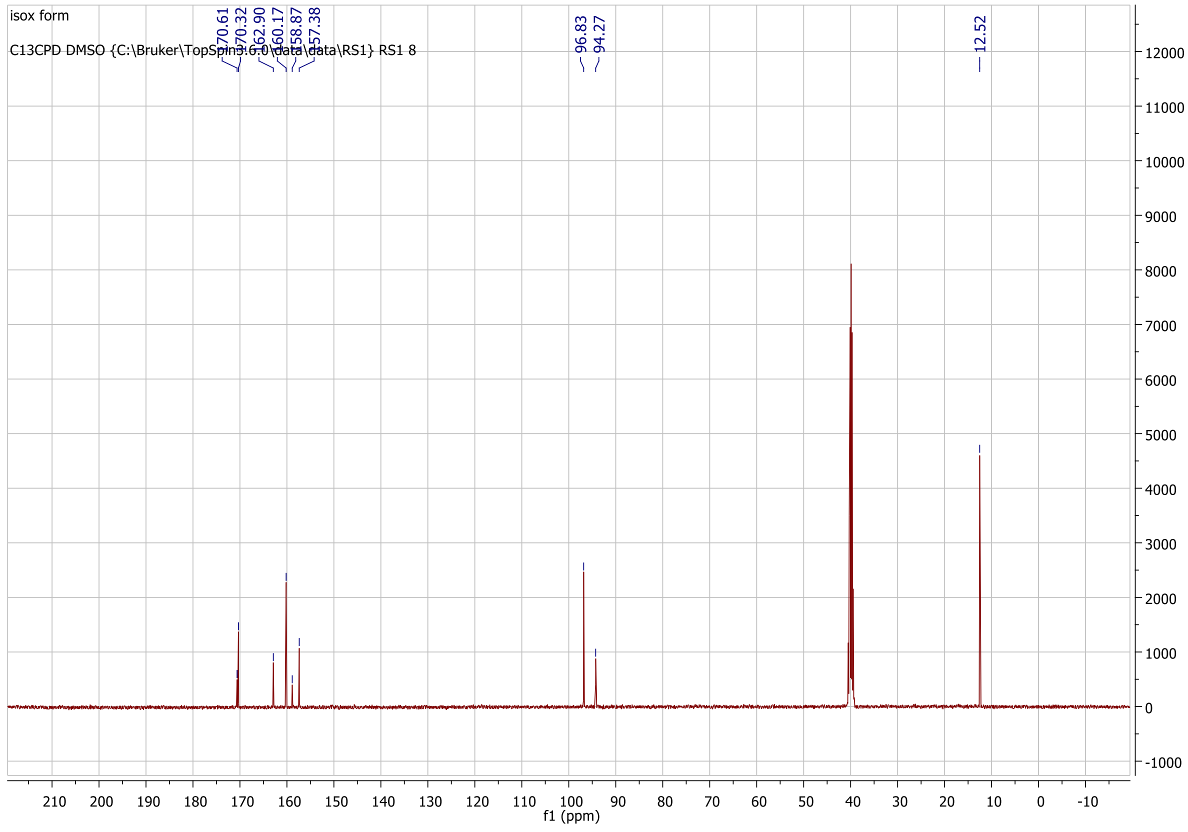

Figure S40.2 ^{13}C NMR (101 MHz, MeOD) spectrum

2.41. *N*-(Furan-2yl)methyl)formamide

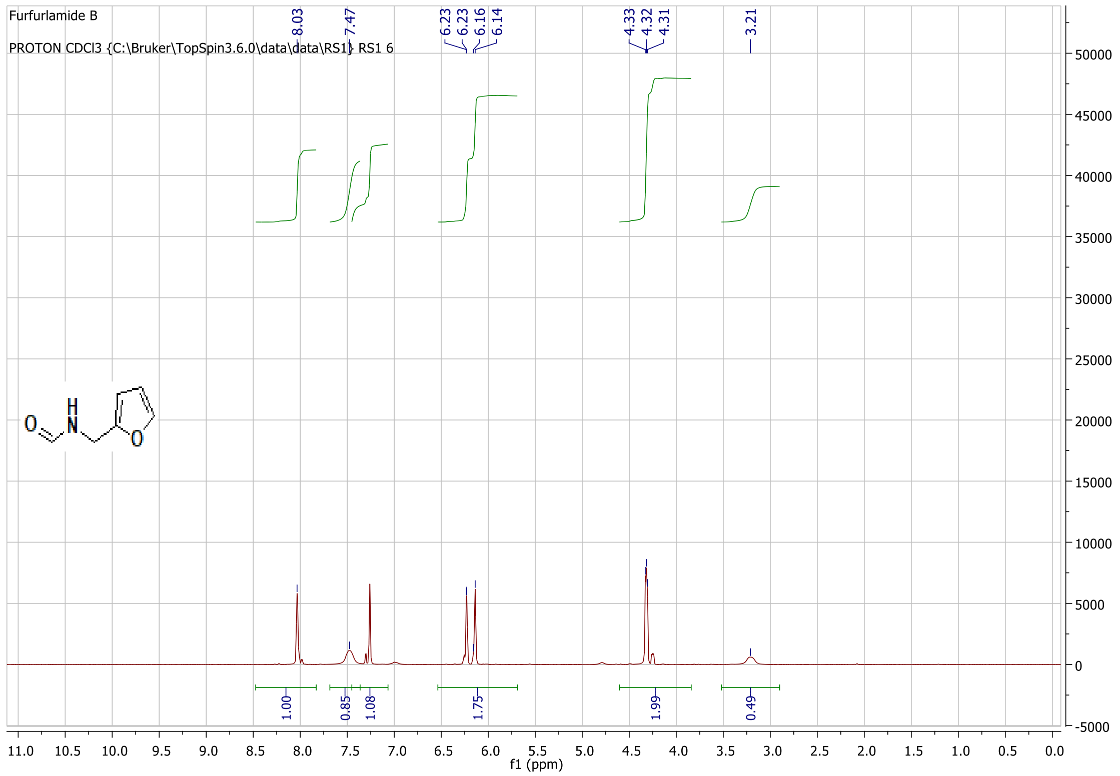

Figure S41.1 ^{1}H NMR (400 MHz, $CDCl_3$) spectrum

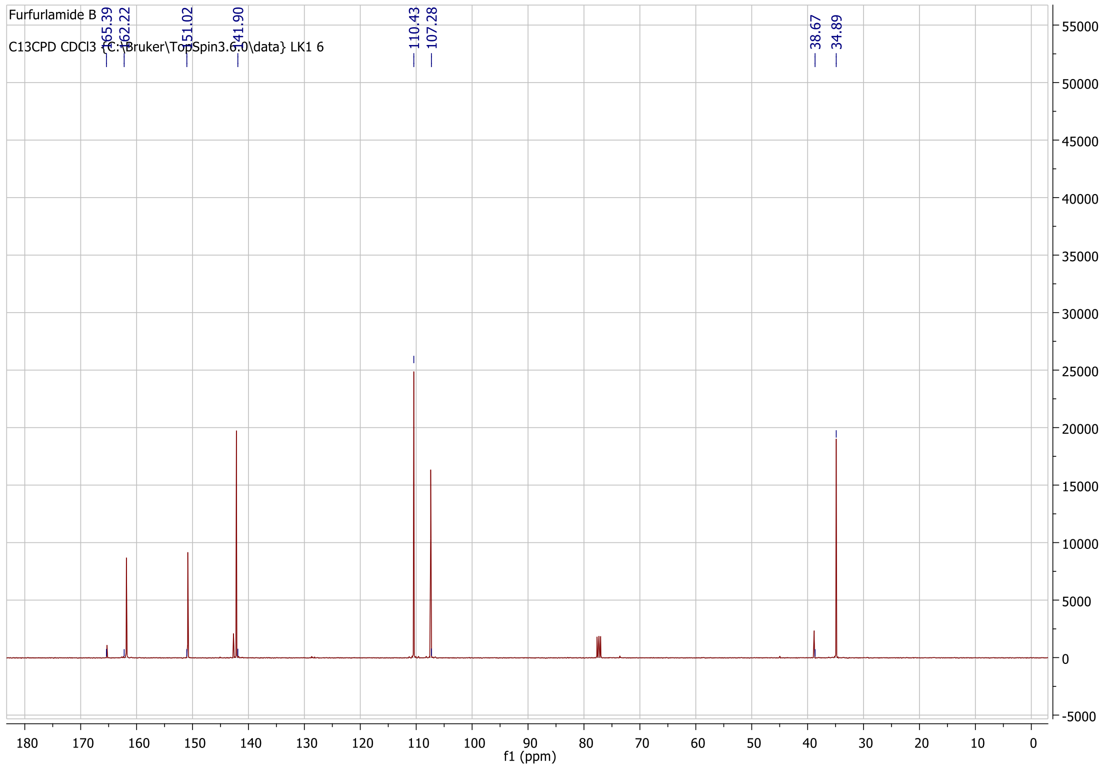

Figure S41.2. ^{13}C NMR (101 MHz, $CDCl_3$) spectrum

2.42. 2-Formamido-3-nitropyridine

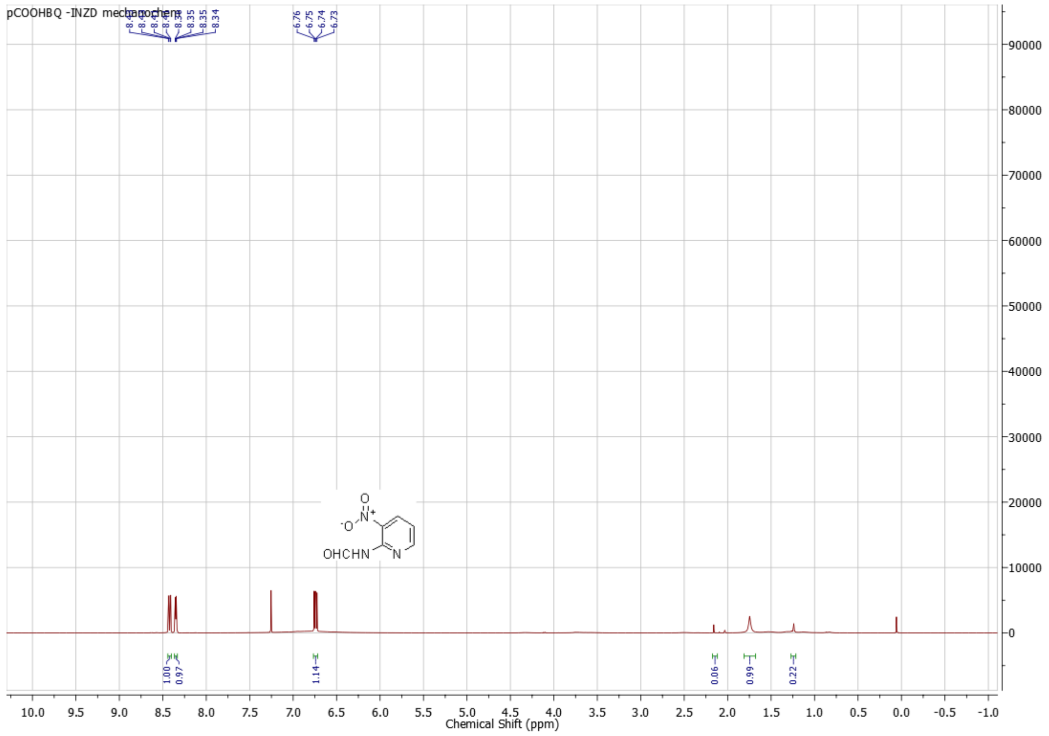

Figure S42.1 ^{1}H NMR (400 MHz, $CDCl_3$) spectrum

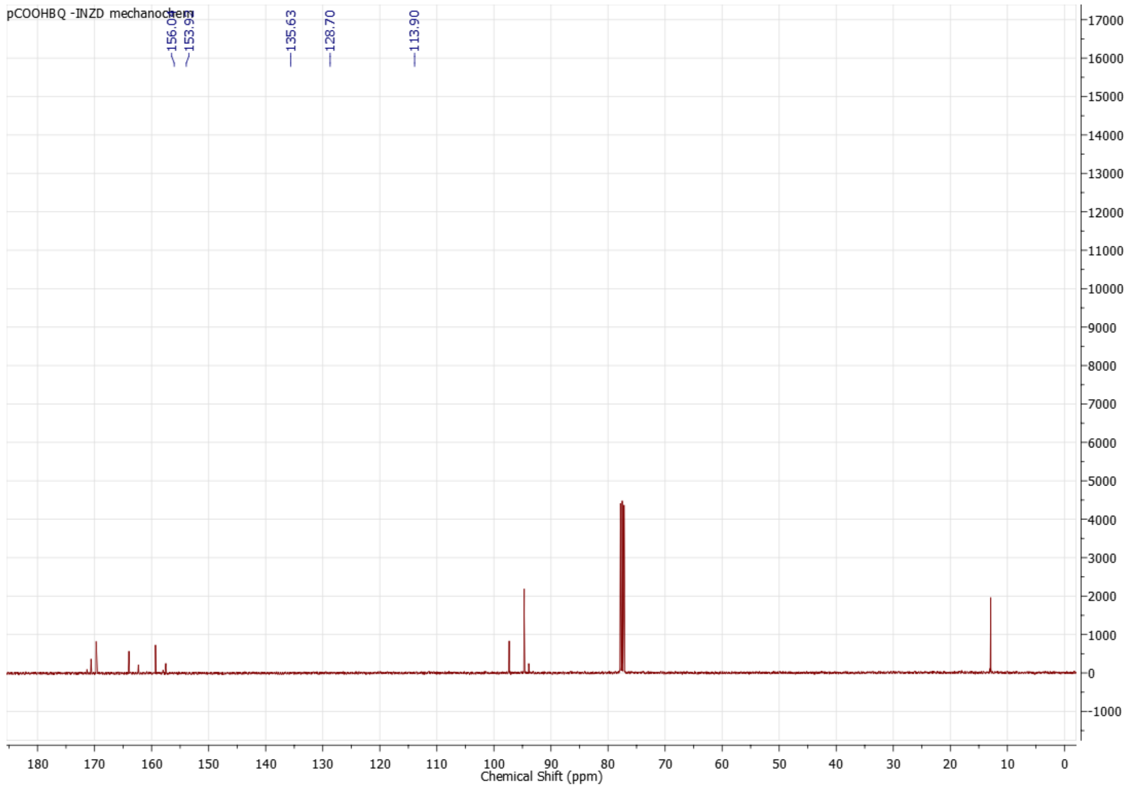

Figure S42.2. ^{13}C NMR (101 MHz, $CDCl_3$) spectrum

2.43. *N*-(thiazol-2-yl)formamide

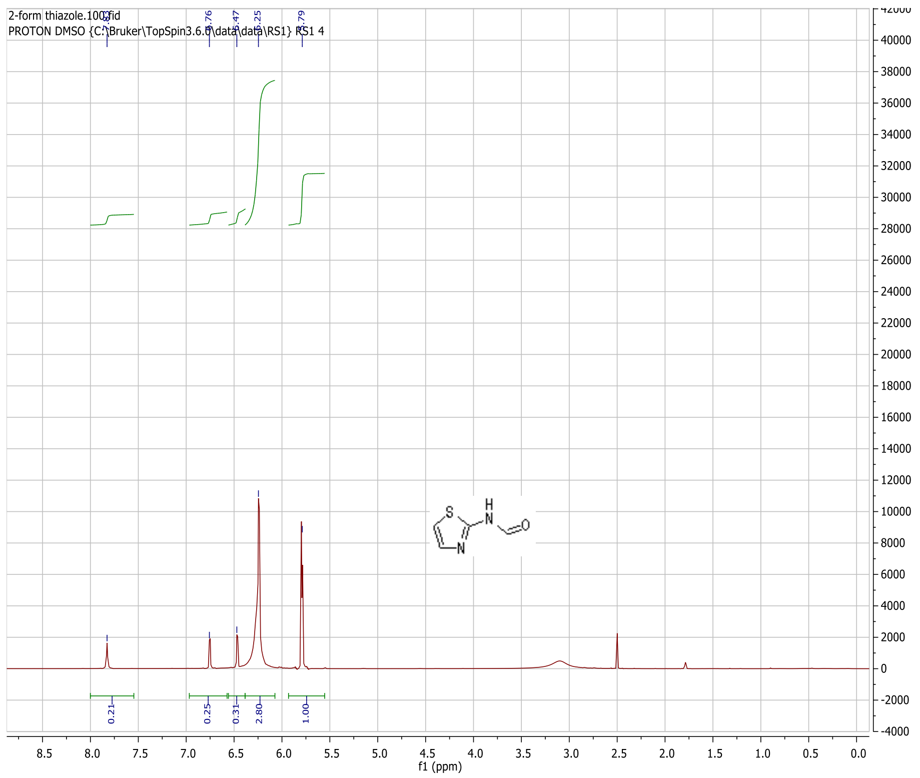

Figure S43.1 ^{1}H NMR (400 MHz, $CDCl_3$) spectrum

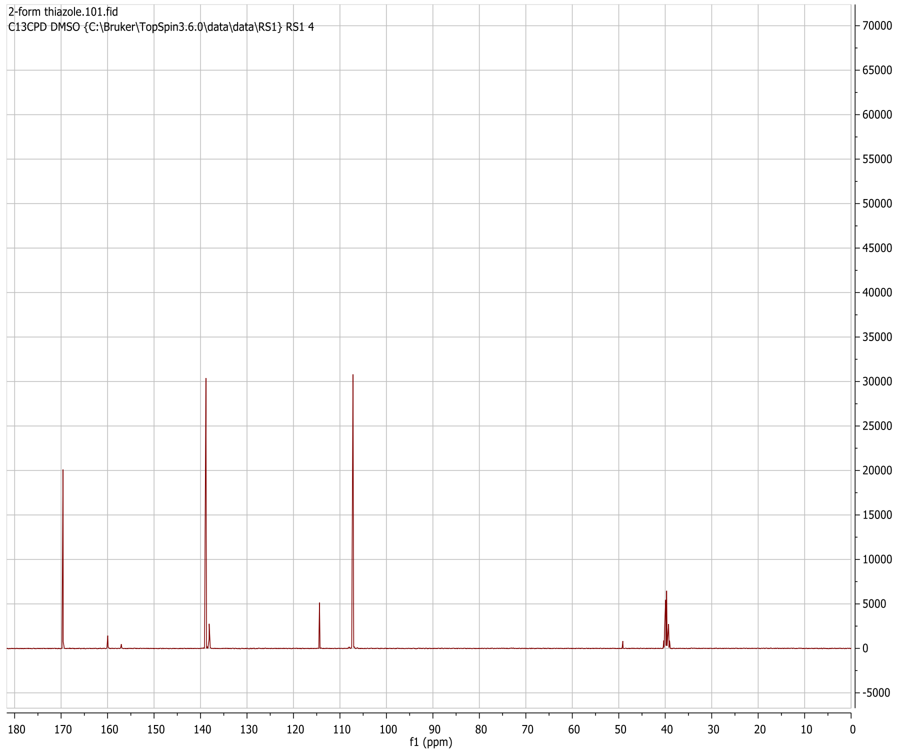

Figure S43.2. ^{13}C NMR (101 MHz, $CDCl_3$) spectrum

2.44. 2-Formamido benzyimidazole

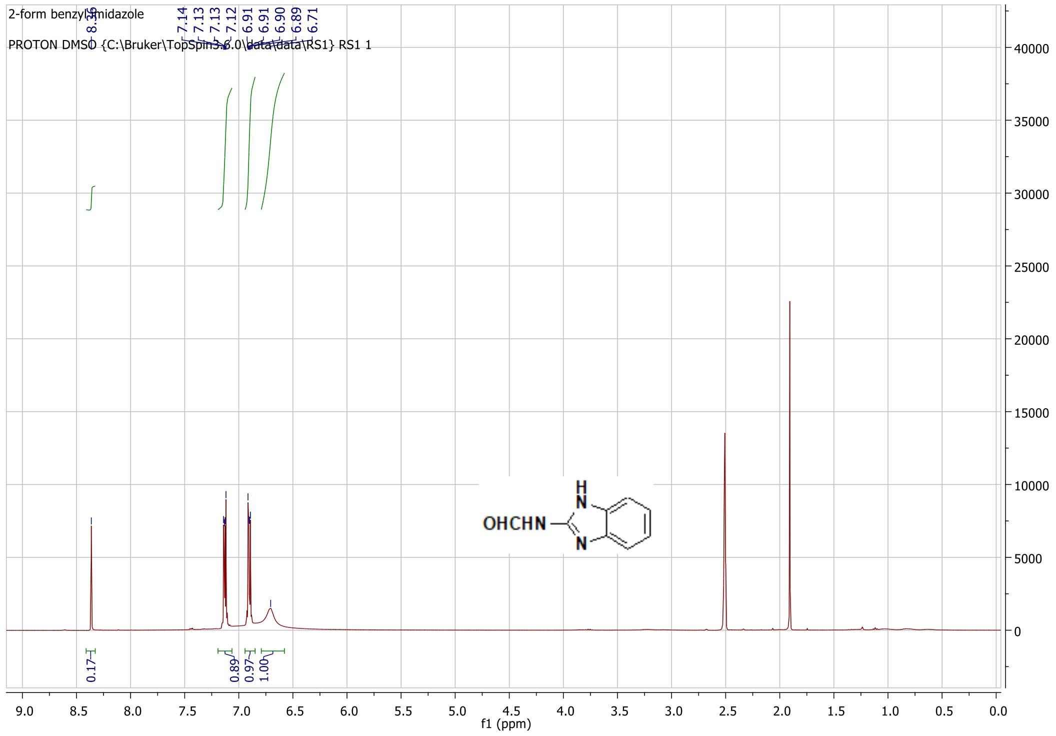

Figure S44.1 ^{1}H NMR (400 MHz, DMSO-d_6) spectrum

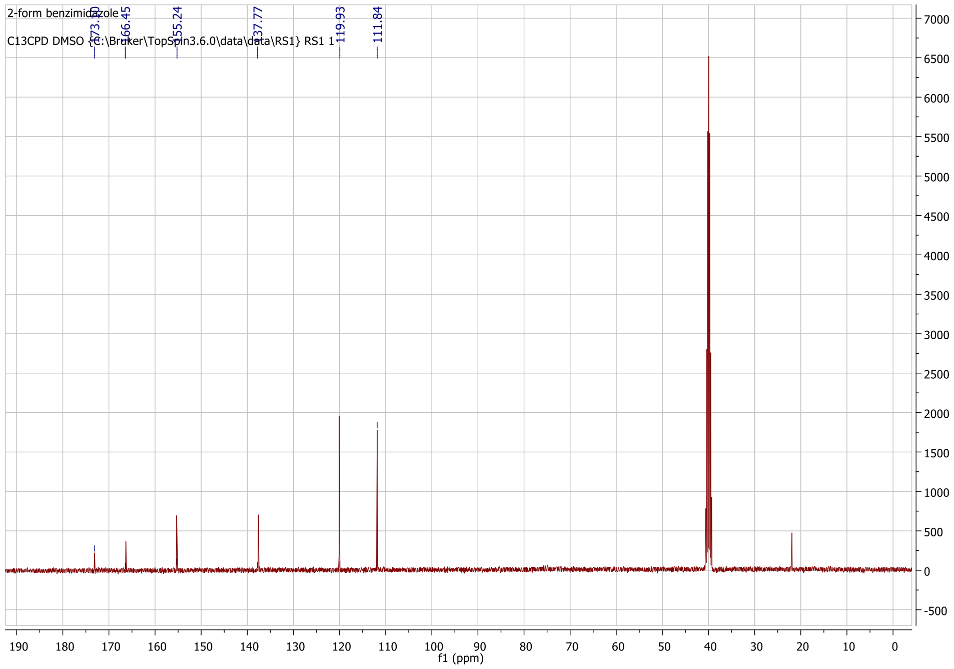

Figure S44.2 ^{13}C NMR (101 MHz, DMSO-d_6) spectrum

2.45. 4-formamido-antipyrine

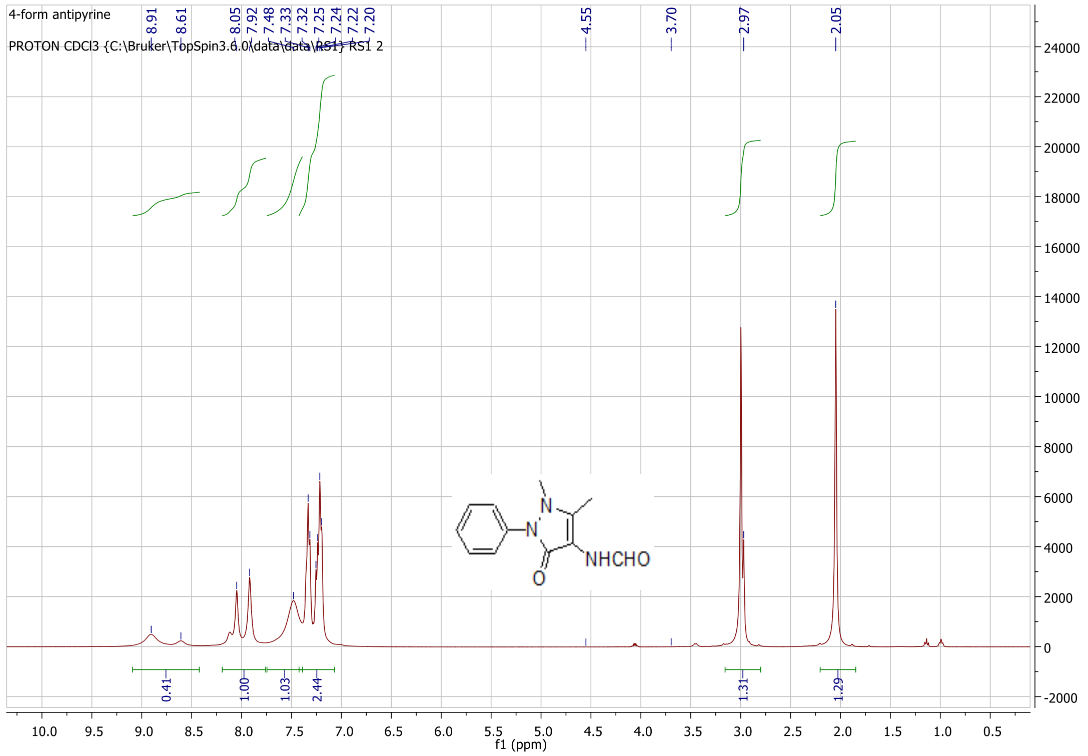

Figure S45.1 ^{1}H NMR (400 MHz, $CDCl_3$) spectrum

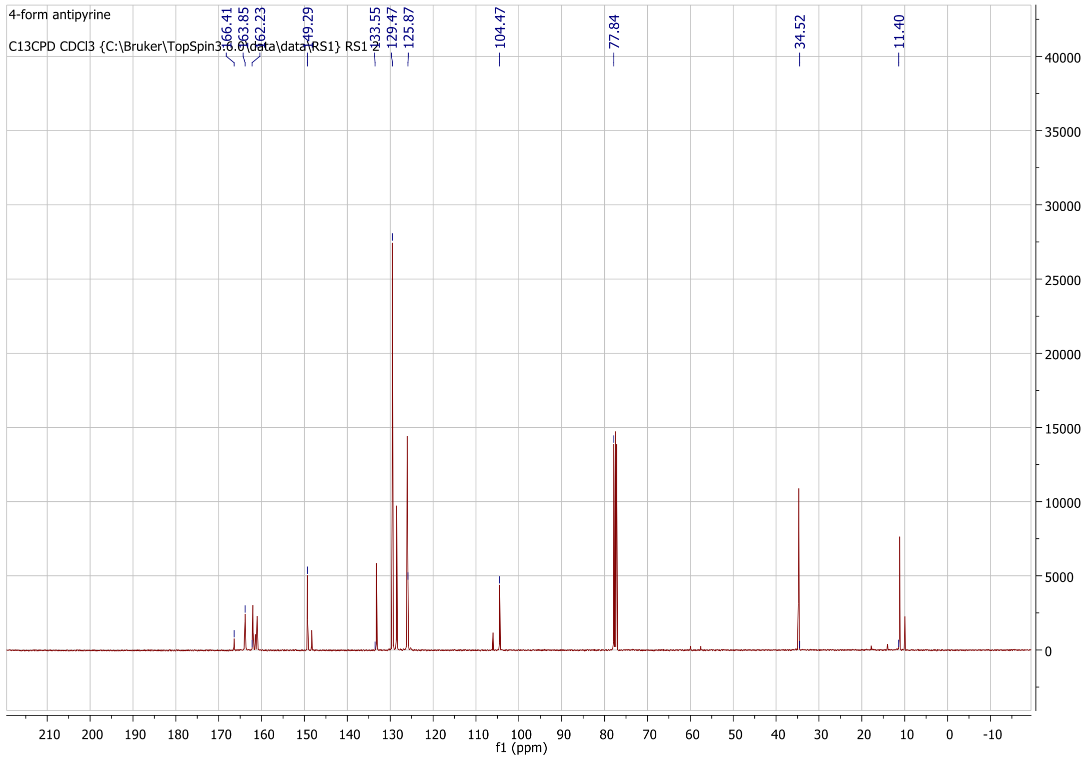

Figure S45.2 ^{13}C NMR (101 MHz, $CDCl_3$) spectrum

2.46. *N*-(Pyridine -2-yl) formamide

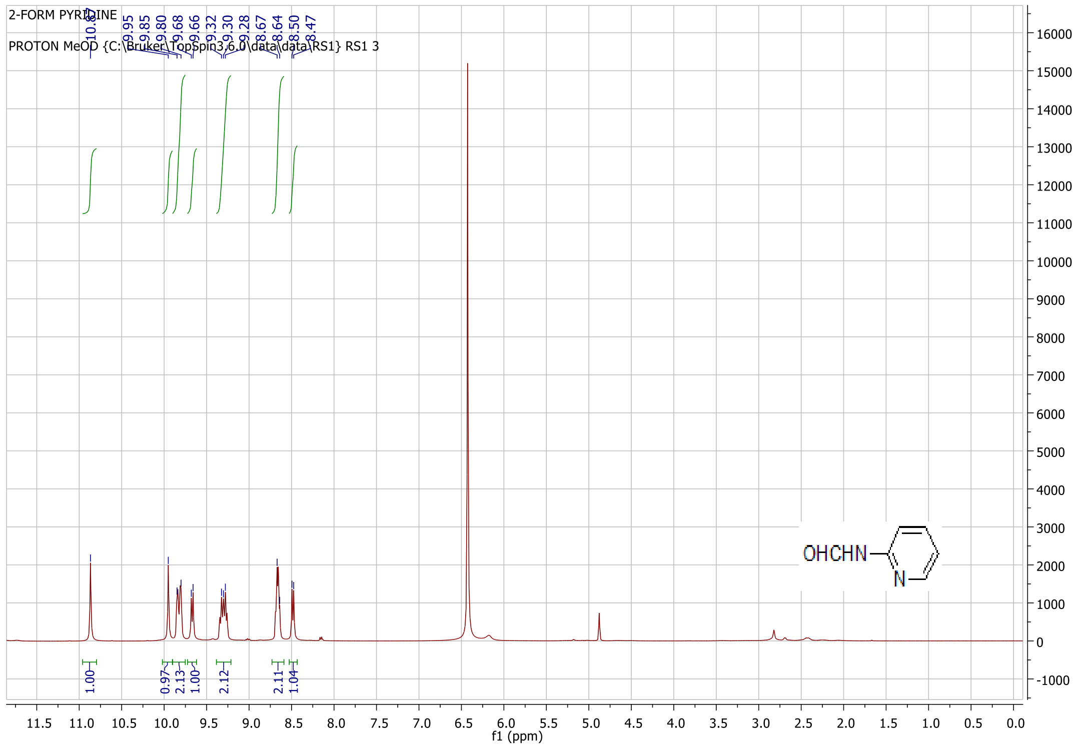

Figure S46.1 ^{1}H NMR (400 MHz, MeOD) spectrum

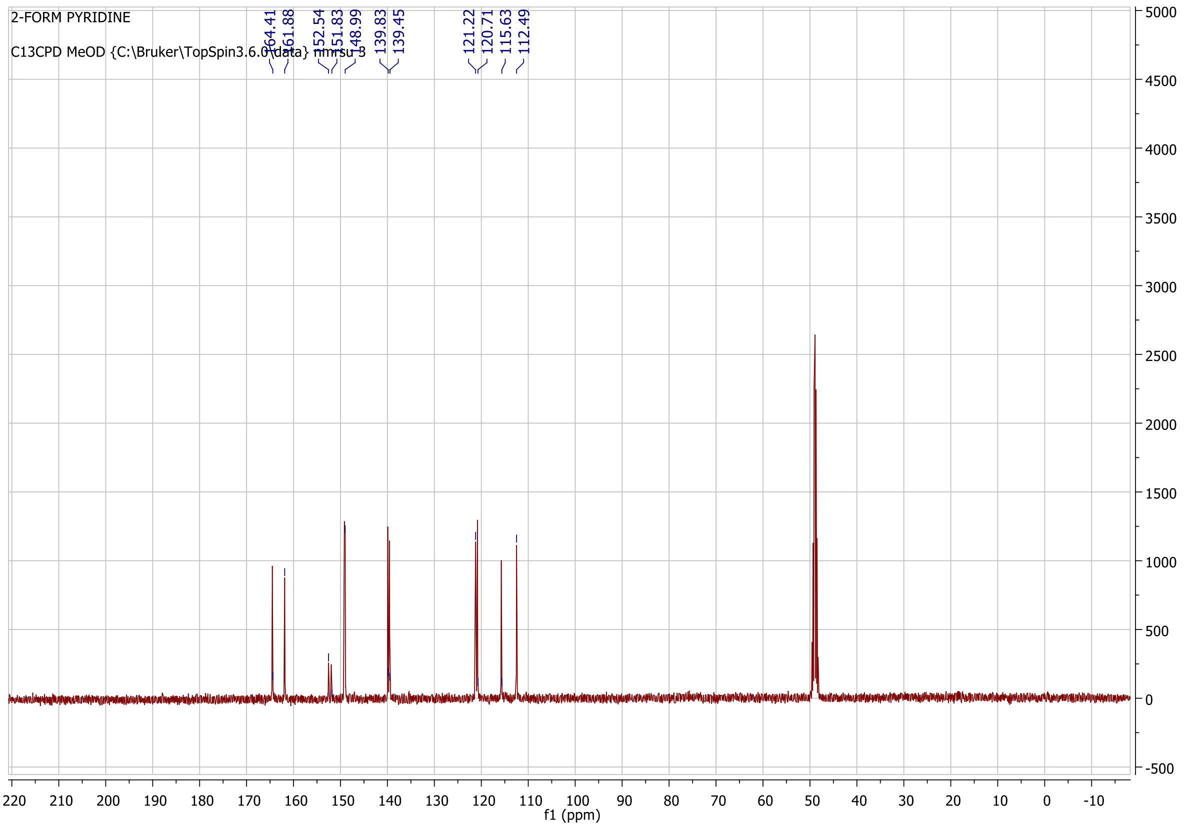

Figure S46.2 ^{13}C NMR (101 MHz, MeOD) spectrum

2.47. *N*-(Pyridine -4-yl) formamide

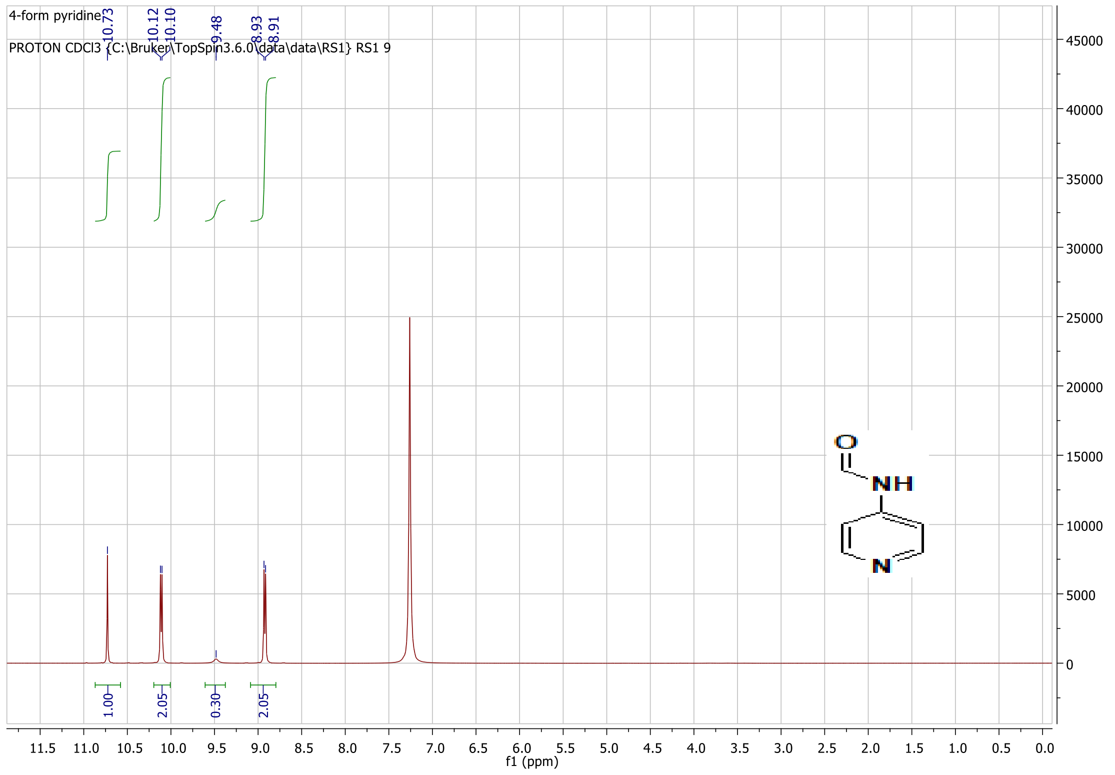

Figure S47.1 ^{1}H NMR (400 MHz, $CDCl_3$) spectrum

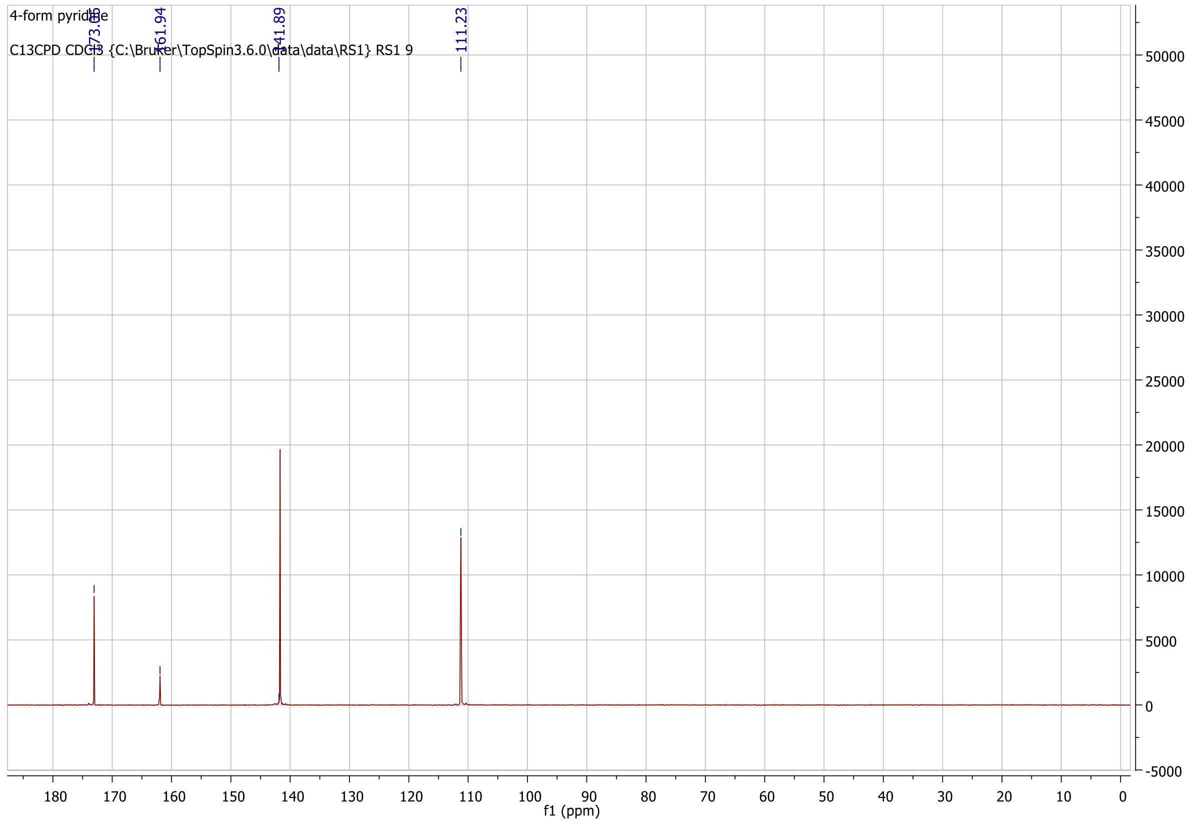

Figure S47.2 ^{13}C NMR (101 MHz, $CDCl_3$) spectrum

2.48. *N*-((pyridine-2-yl)methyl)formamide

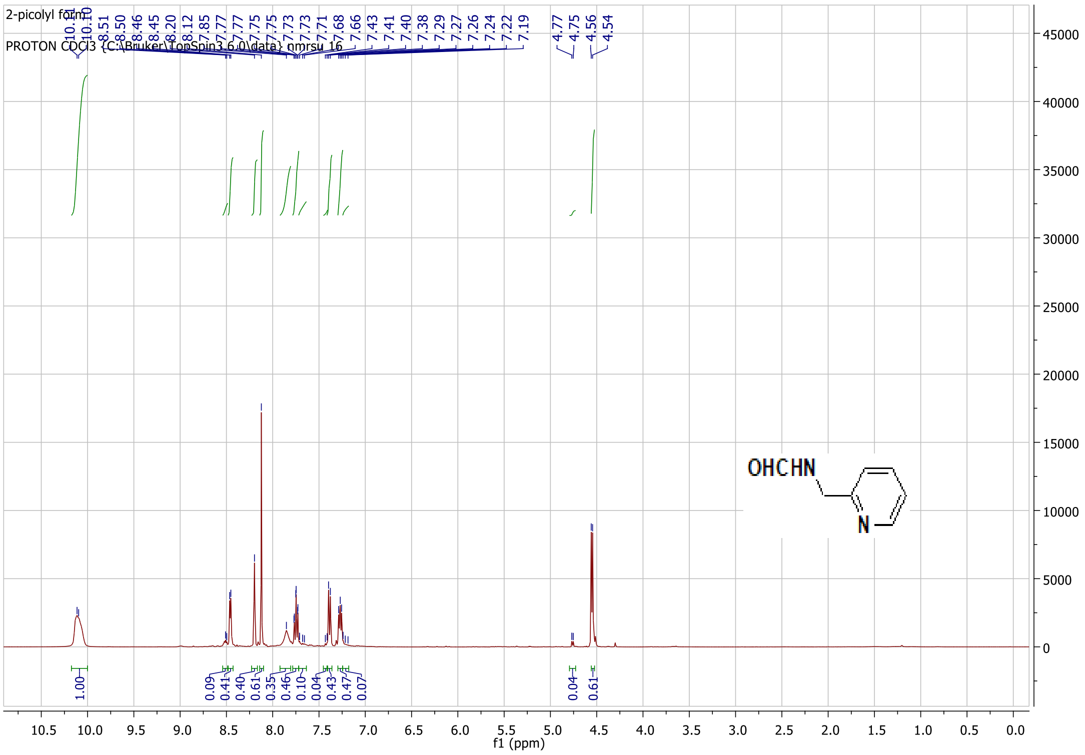

Figure S48.1. ^{1}H NMR (400 MHz, $CDCl_3$) spectrum

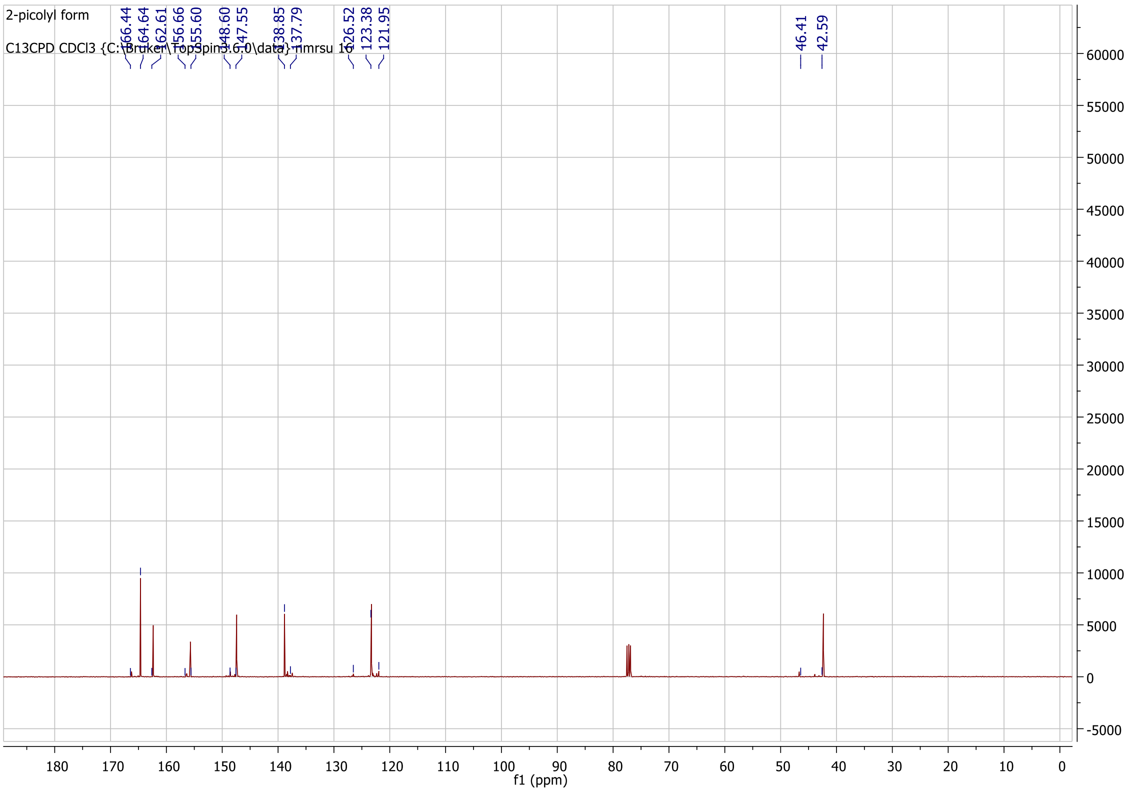

Figure S48.2 ^{13}C NMR (101 MHz, $CDCl_3$) spectrum

2.49. 2-Formamido benzothiazole

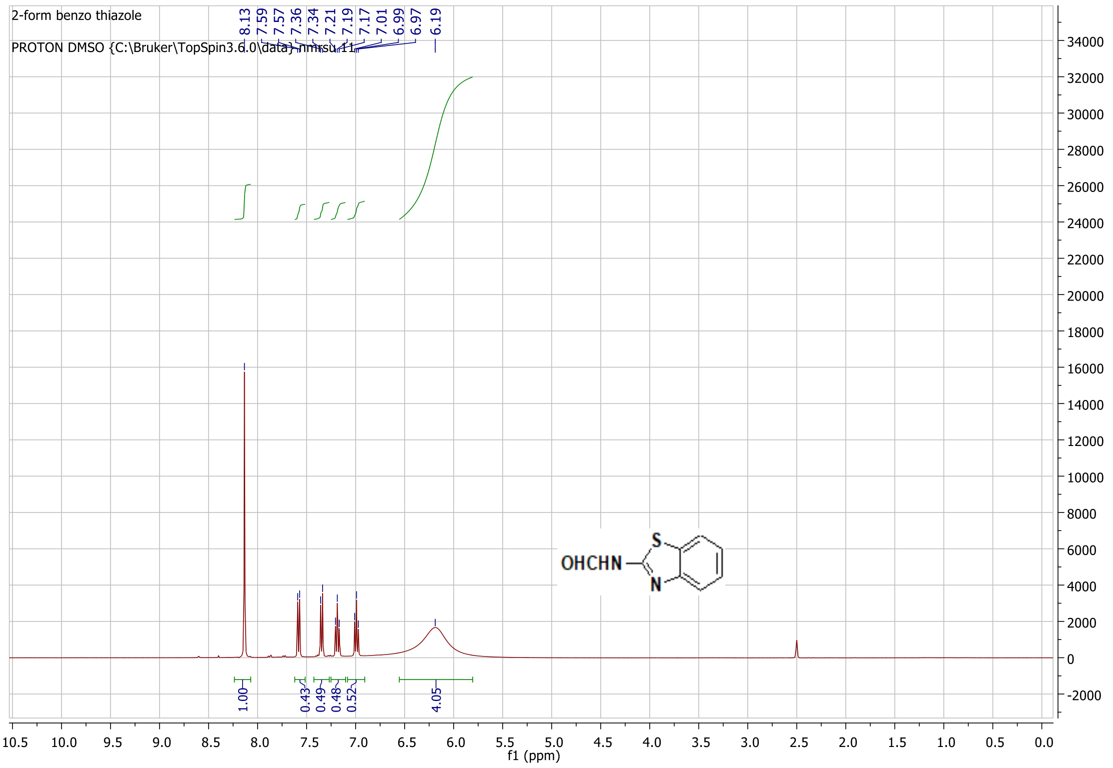

Figure S49.1 ^{1}H NMR (400 MHz, DMSO-d_6) spectrum

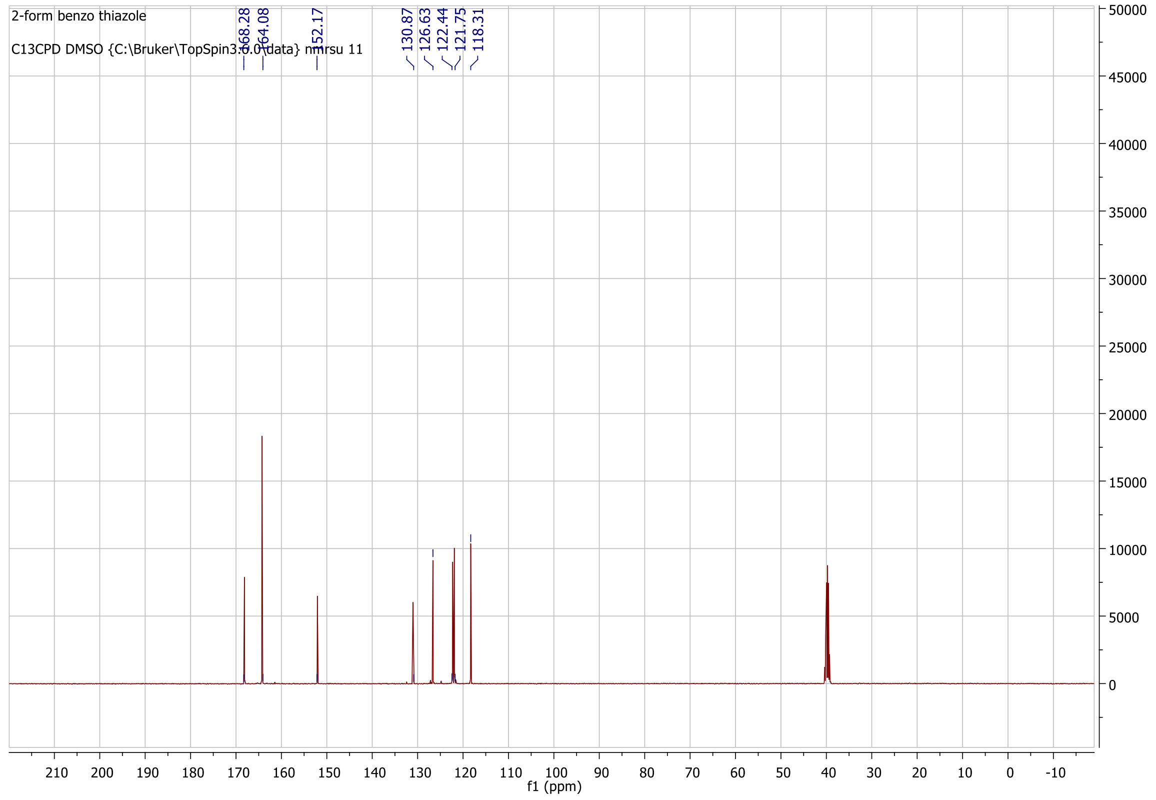

Figure S49.2 ^{13}C NMR (101 MHz, DMSO-d_6) spectrum

2.50. *N*-methyl-N-phenylformamide

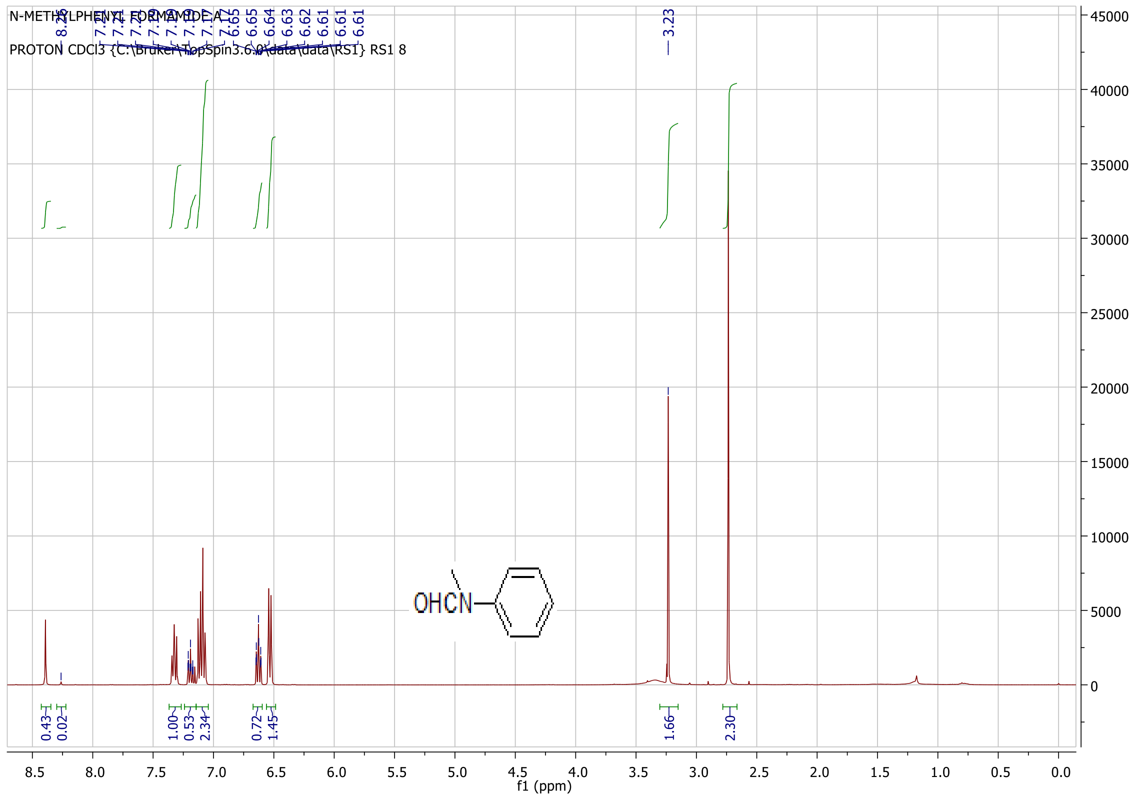

Figure S50.1 ^{1}H NMR (400 MHz, $CDCl_3$) spectrum

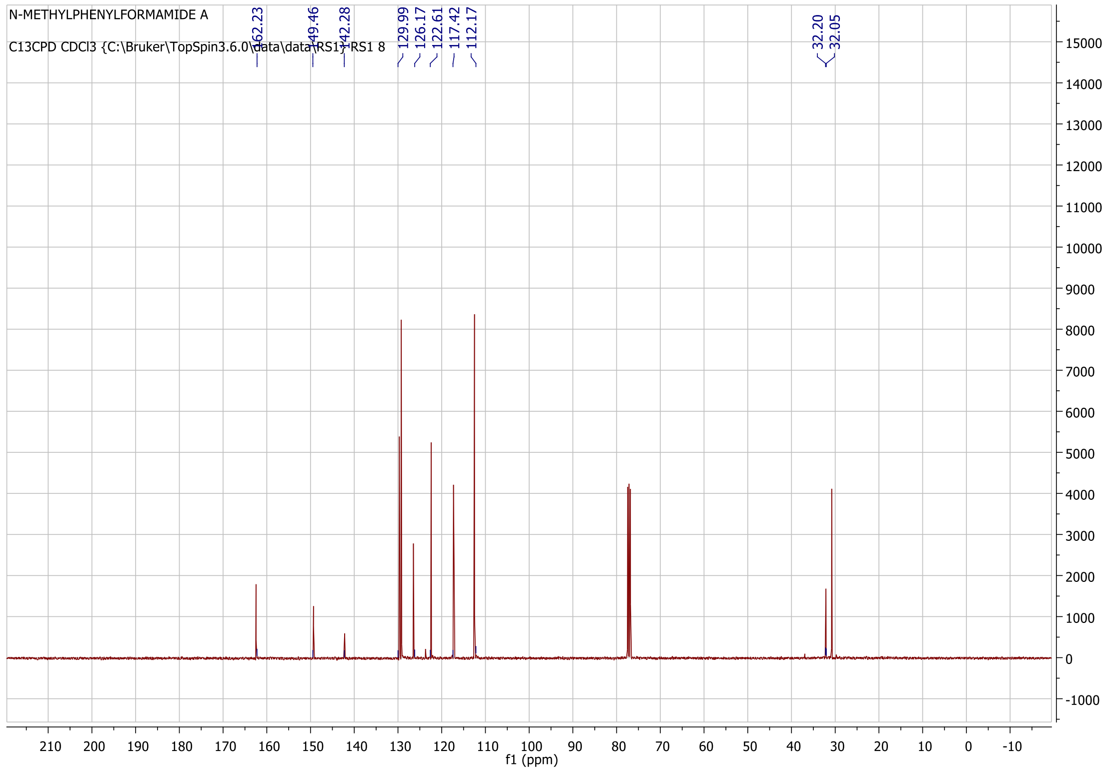

Figure S50.2 ^{13}C NMR (101 MHz, $CDCl_3$) spectrum

2.51. *N*-[4-chloro-2-(2-fluorobenzoyl)phenyl]formamide

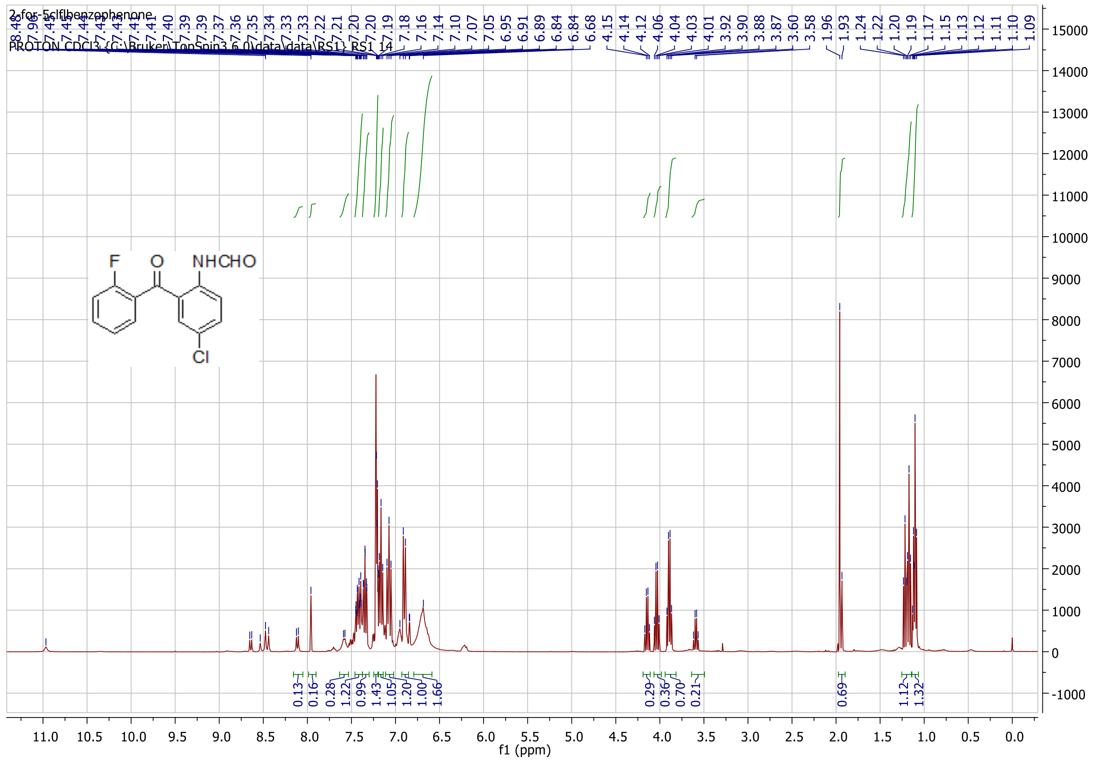

Figure S51.1 ^{1}H NMR (400 MHz, $CDCl_3$) spectrum

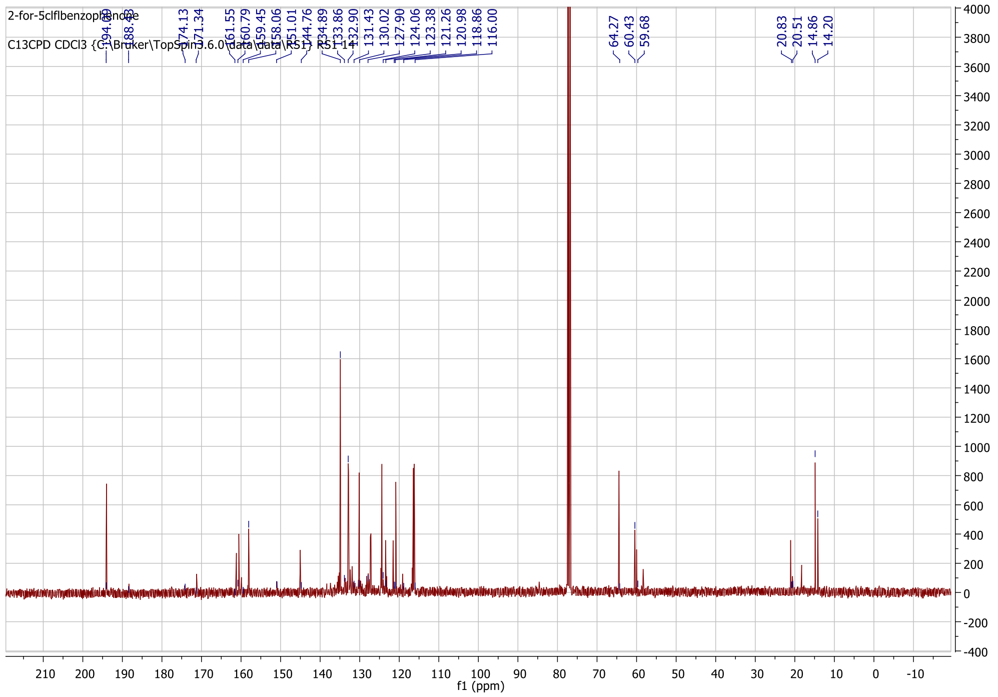

Figure S51.2. ^{13}C NMR (101 MHz, $CDCl_3$) spectrum

2.52. Piperidine-1-carbaldehde

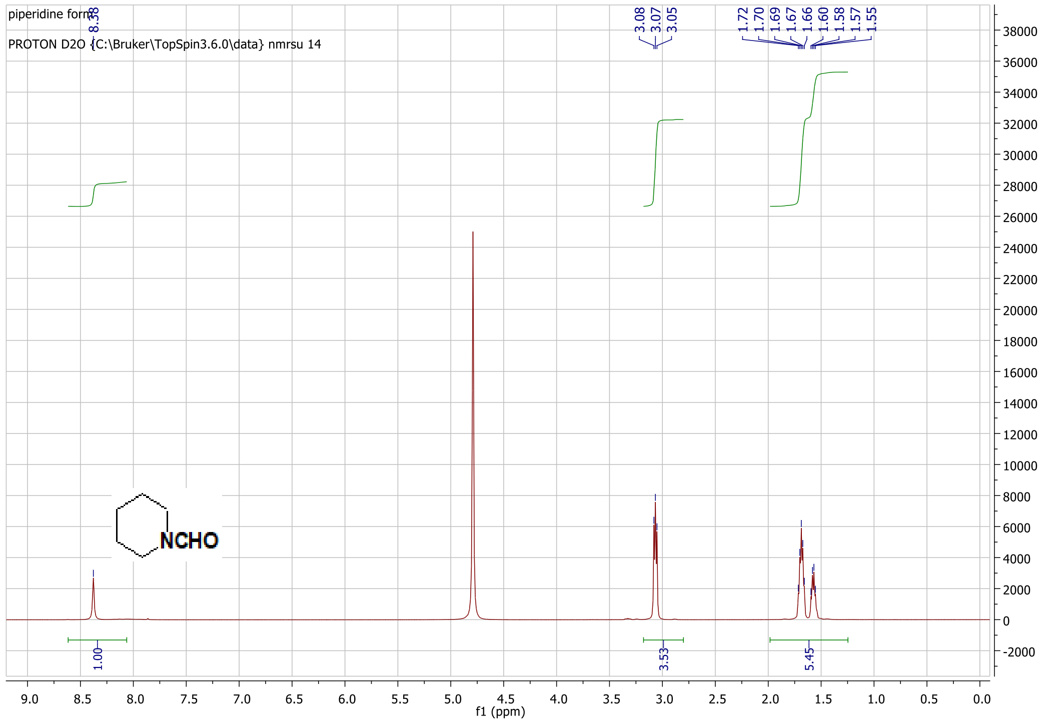

Figure S52.1 ^{1}H NMR (400 MHz, D_2O) spectrum

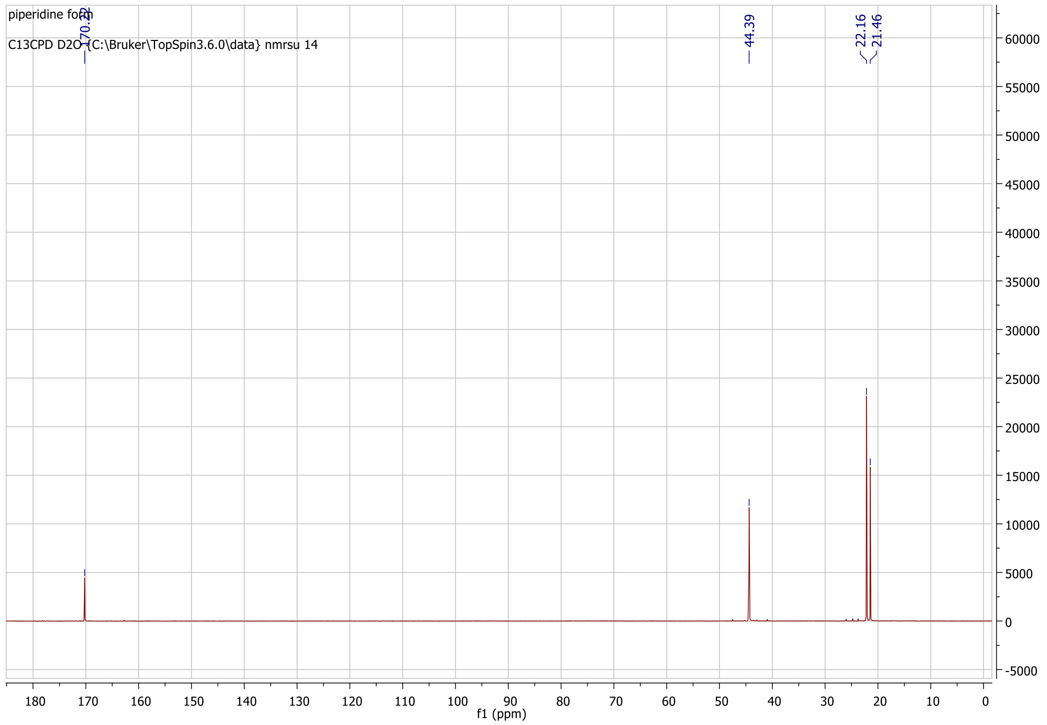

Figure S52.2 ^{13}C NMR (101 MHz, D_2O) spectrum

2.53. 3-Formamido-1,2,4-triazole-5-thiol

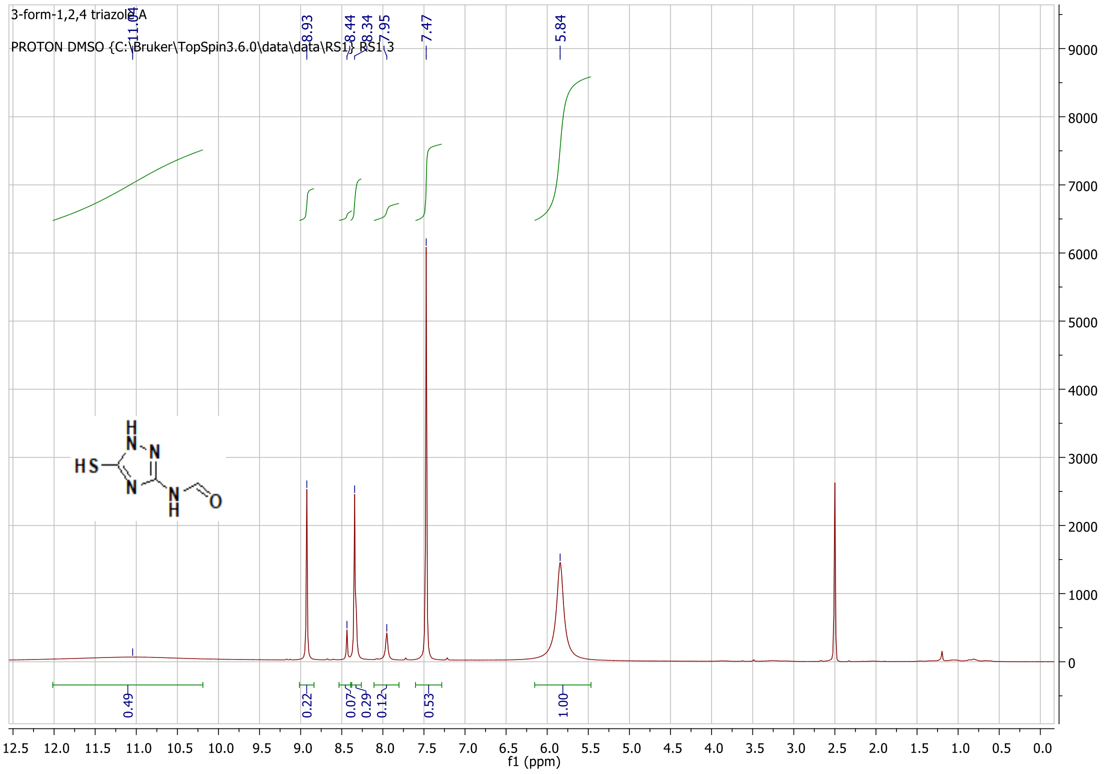

Figure S53.1 ^{1}H NMR (400 MHz, DMSO-d_6) spectrum

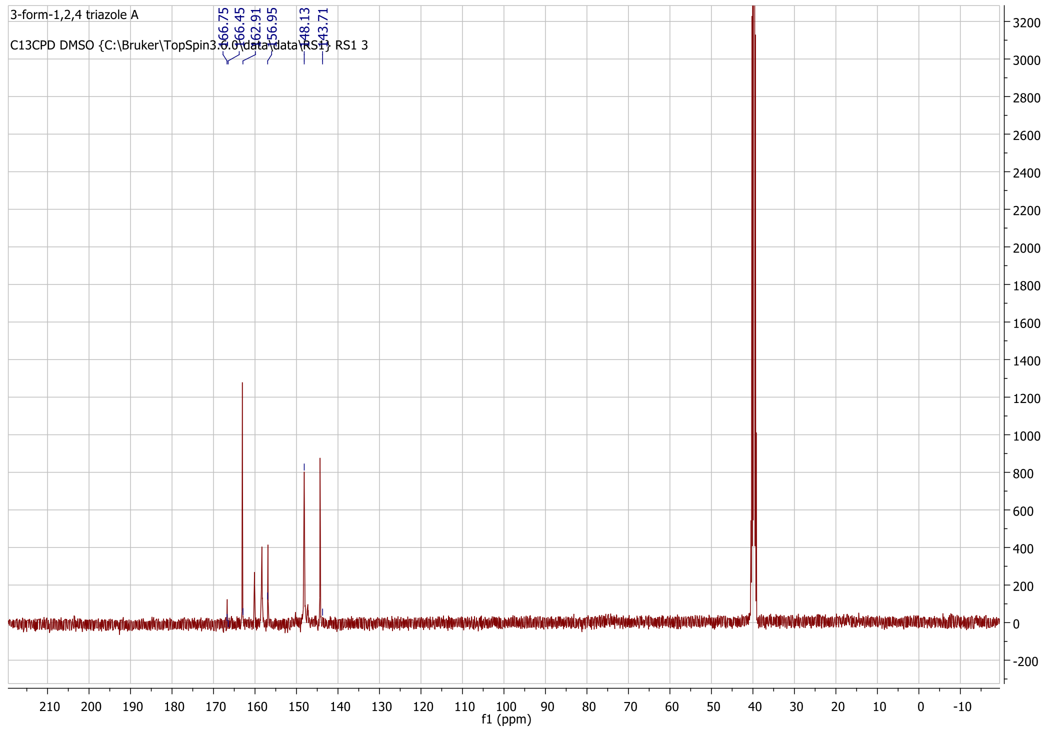

Figure S53.2 ^{13}C NMR (101 MHz, DMSO-d_6) spectrum

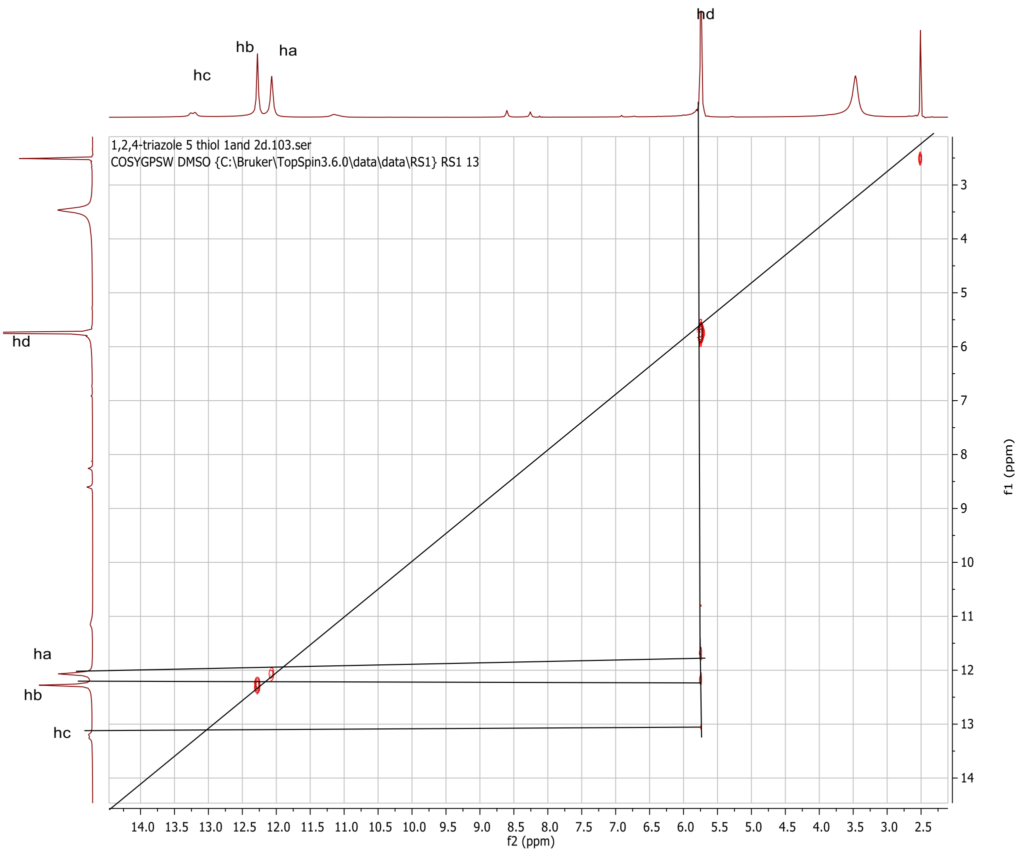

Figure S53.3 COSY (400 MHz, DMSO-d_6) spectrum

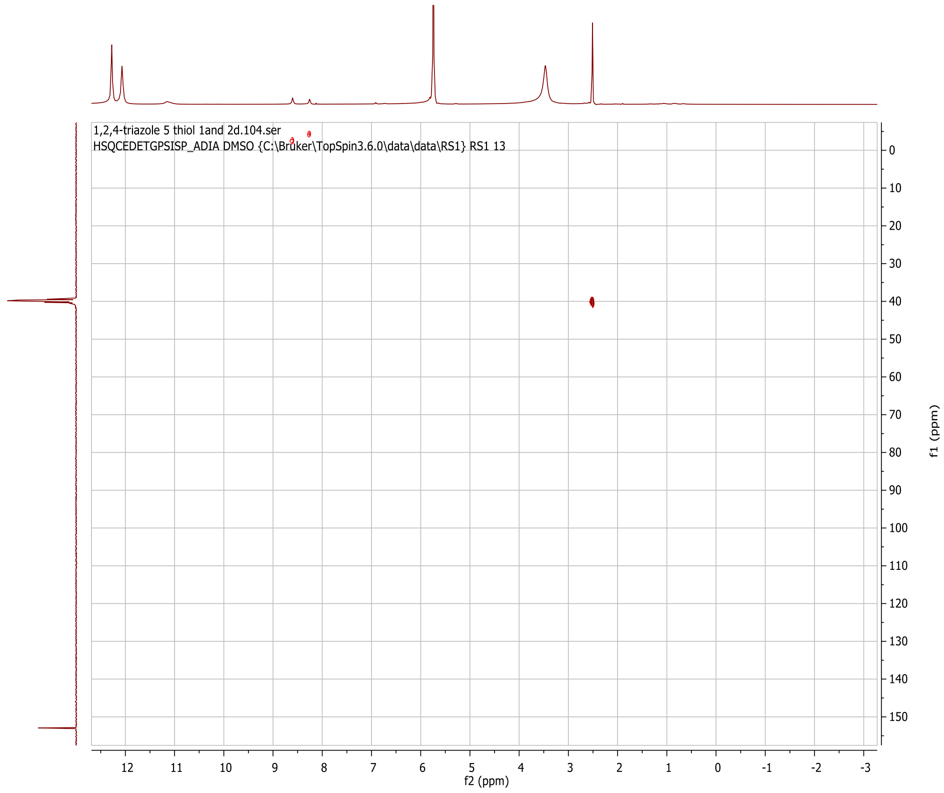

Figure S53.4 HSQC (400 MHz, DMSO-d_6) spectrum

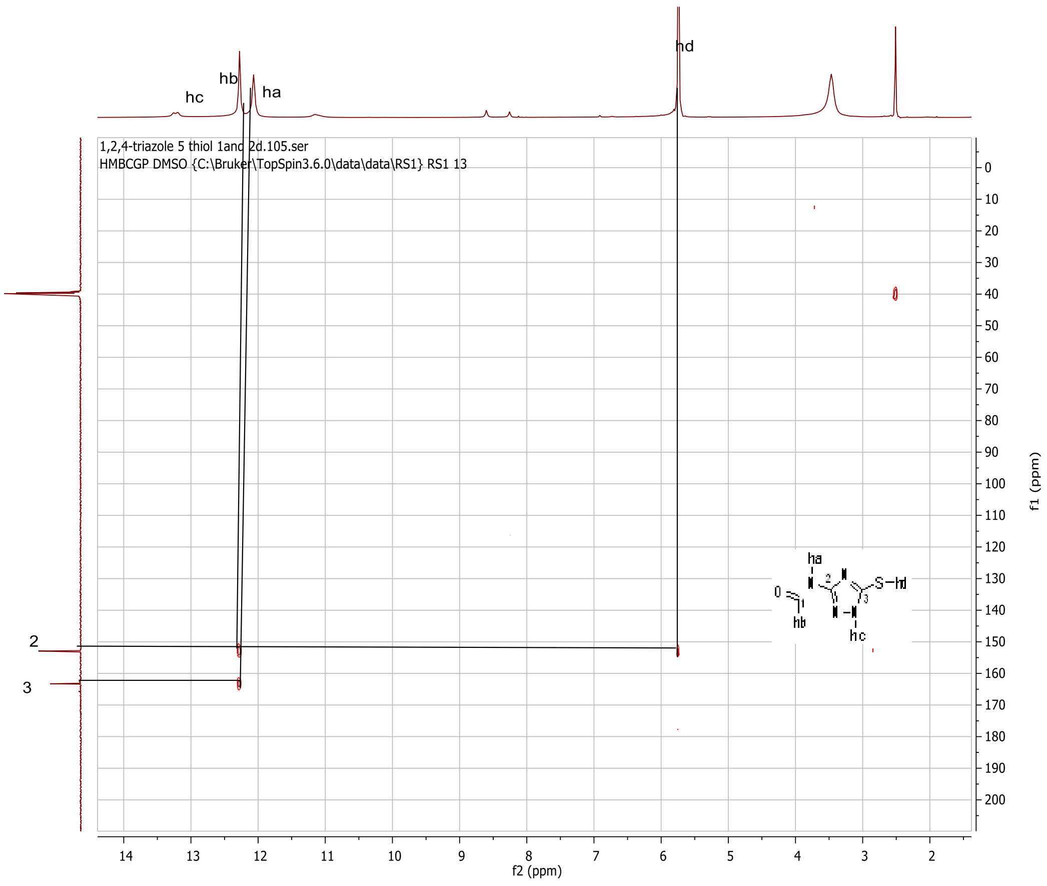

Figure S53.5 HMBC (400 MHz, DMSO-d_6) spectrum

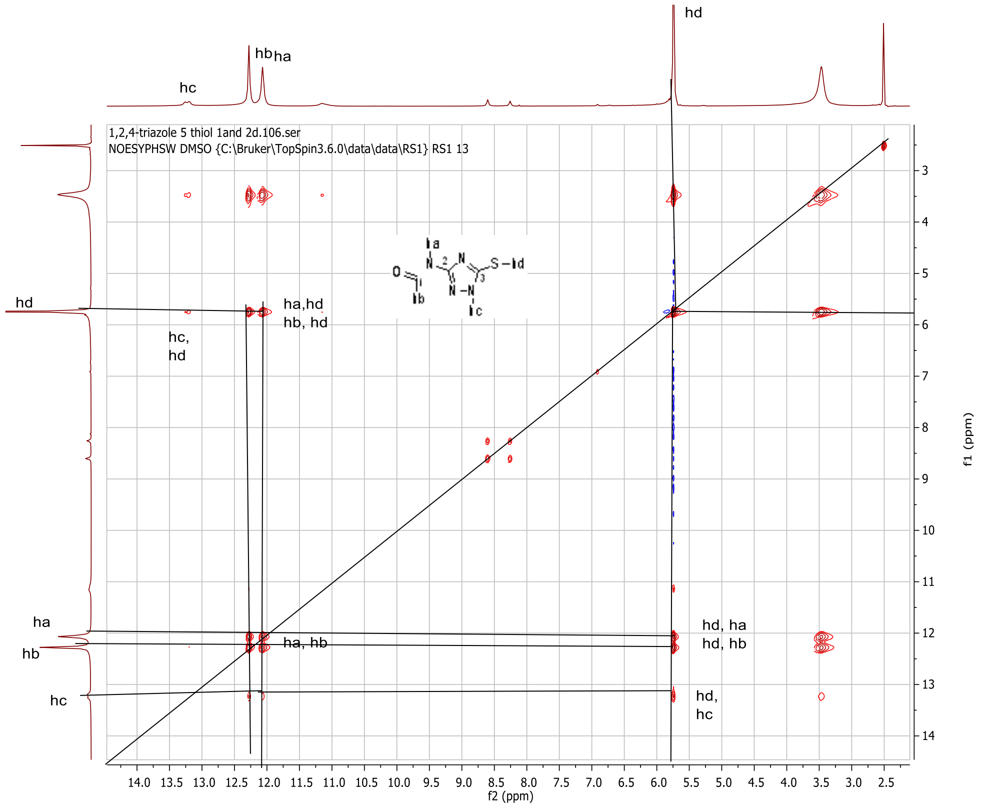

Figure S53.6 NOESI (400 MHz, DMSO-d_6) spectrum

2.54. *N*-[2-(3,4-dihydroxyphenyl)-2-hydroxyethyl]-N- methylformamide

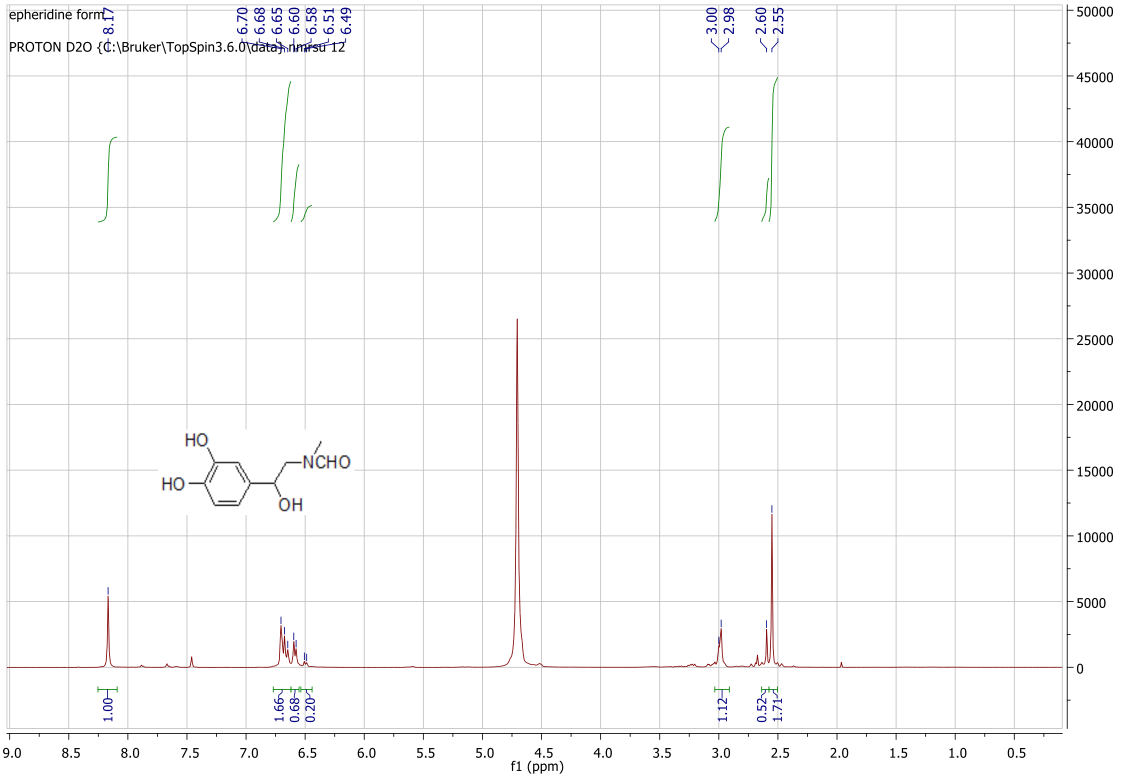

Figure S54.1 ^{1}H NMR (400 MHz, D_2O) spectrum

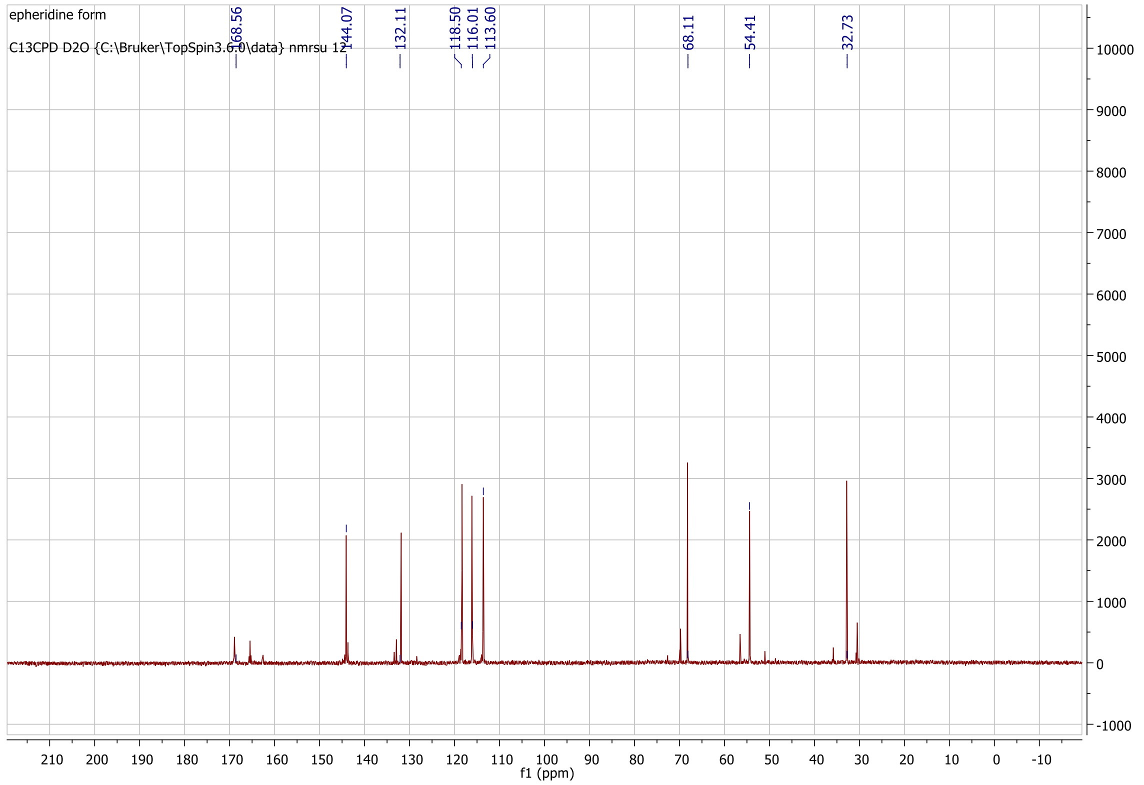

Figure S54.2 ^{13}C NMR (101 MHz, D_2O) spectrum

2.55. *N*-(2-mercaptoethyl)formamide

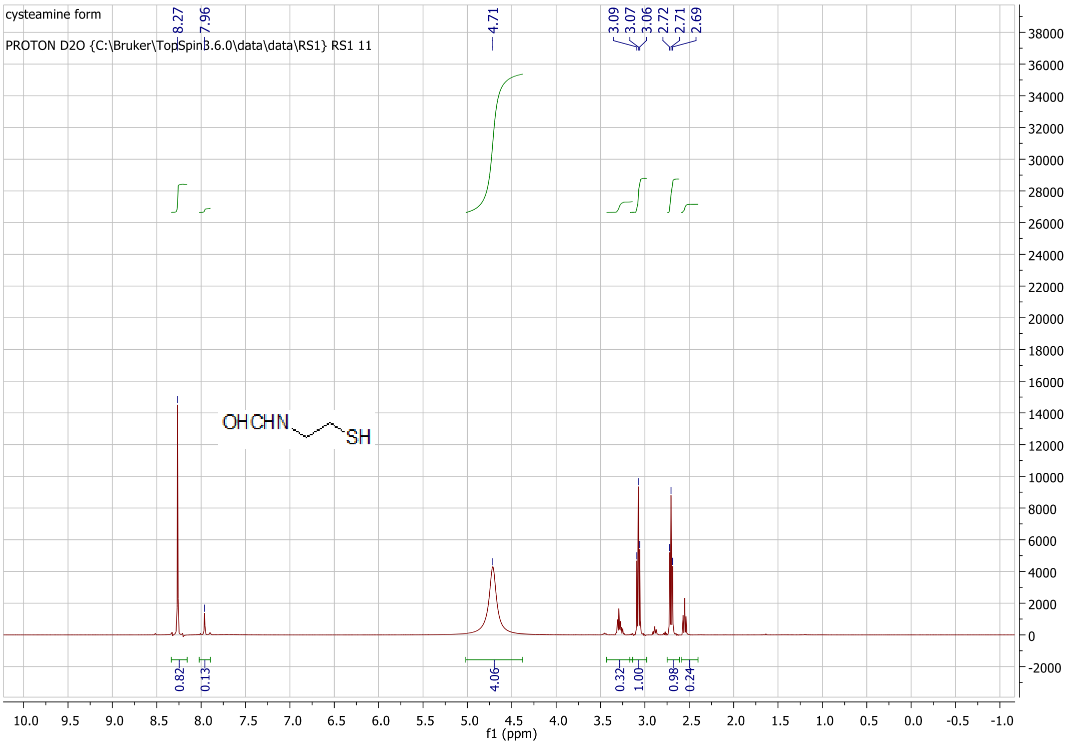

Figure S55.1 ^{1}H NMR (400 MHz, D_2O) spectrum

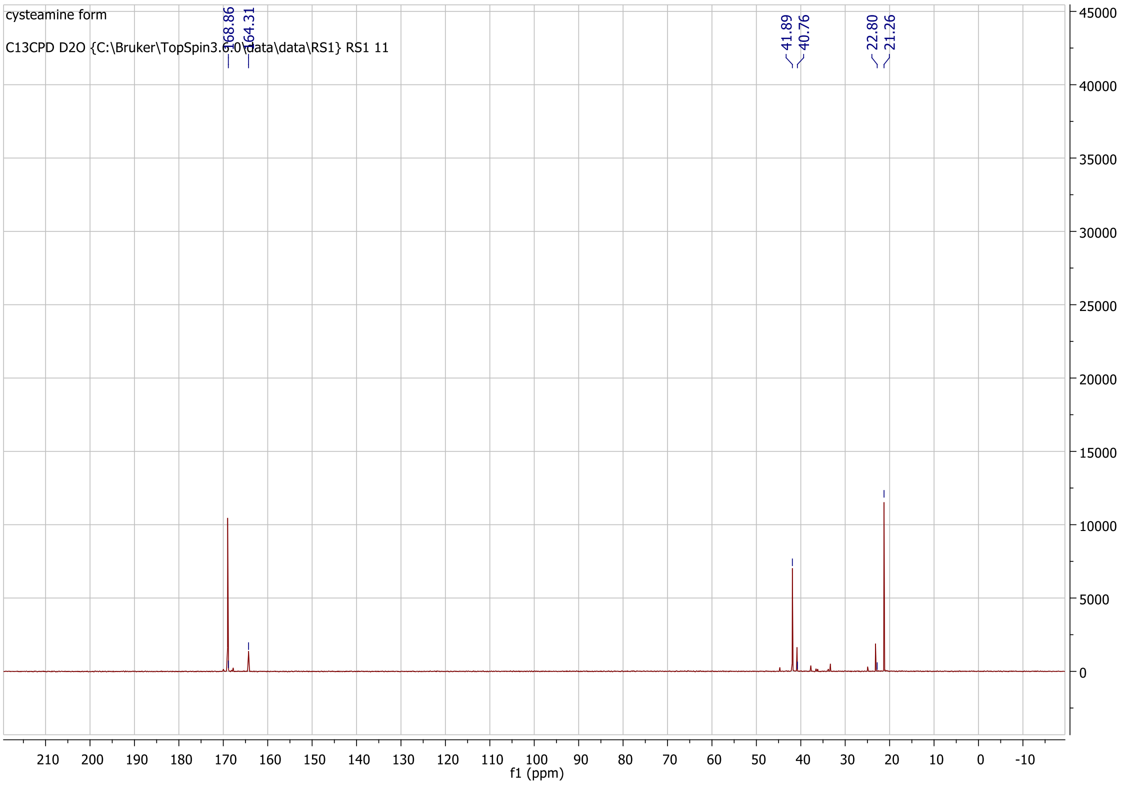

Figure S55.2 ^{13}C NMR (101 MHz, D_2O) spectrum

2.56. *N*-formylpyridine-3-carboxamide

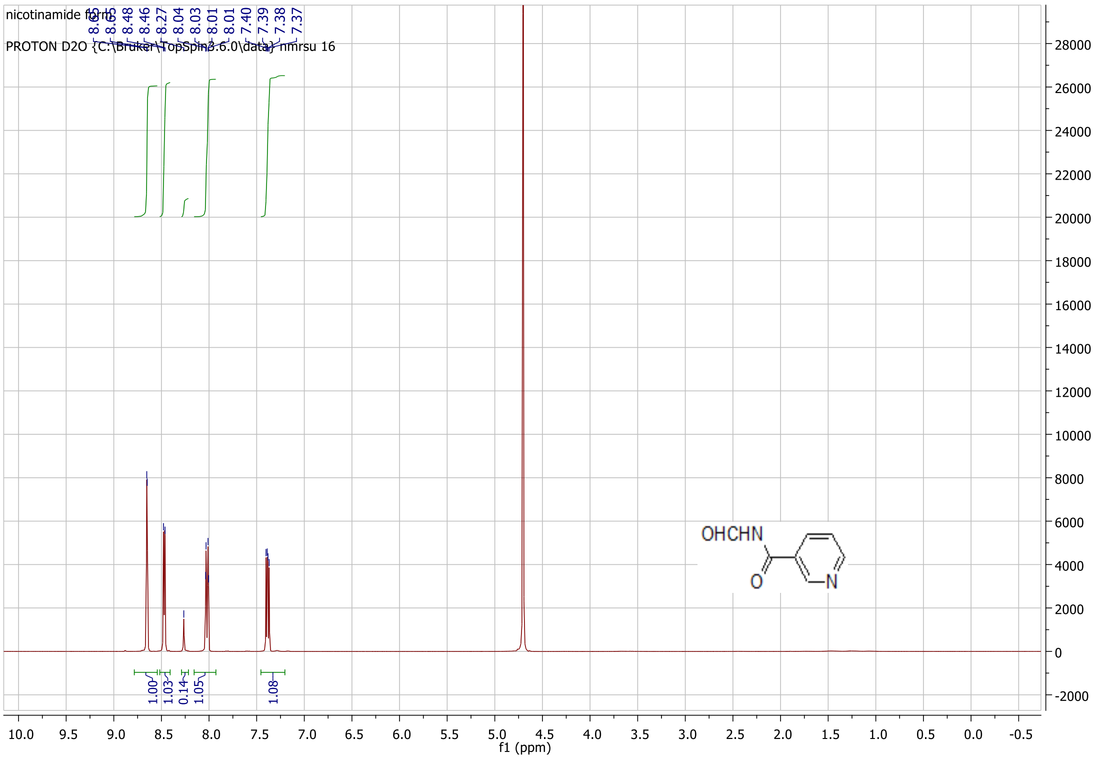

Figure S56.1 ^{1}H NMR (400 MHz, D_2O) spectrum

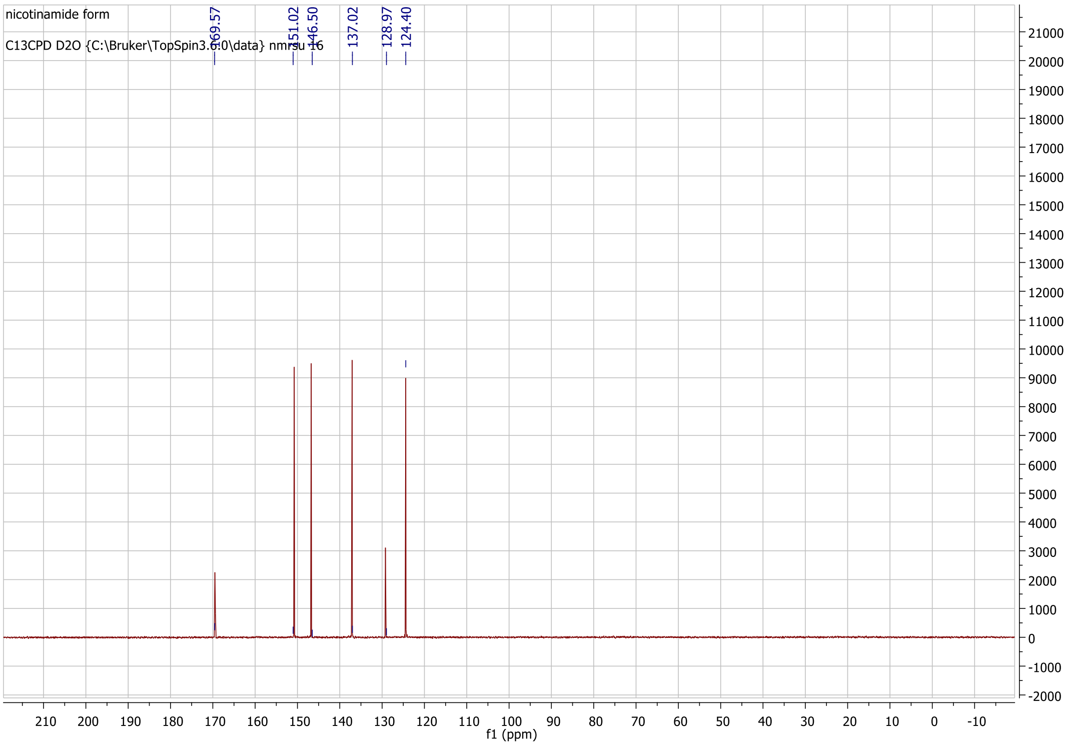

Figure S56.2 ^{13}C NMR (101 MHz, D_2O) spectrum

3. Mass spectroscopy of the new derivatives 38 and 53

LC-MS/MS data were recorded on a Bruker Compact quadrupole time of flight (QToF) mass spectrometer (Bremen, Germany).

3.1 1,8-difformamido naphthalene (38) ($C_{12}H_{10}N_2O_2$)

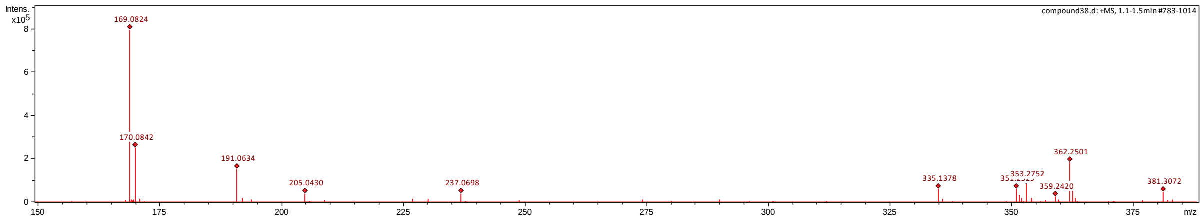

3.2 3-Formamido-1,2,4-triazole-5-thiol. 53 ($C_3H_4N_4OS$)

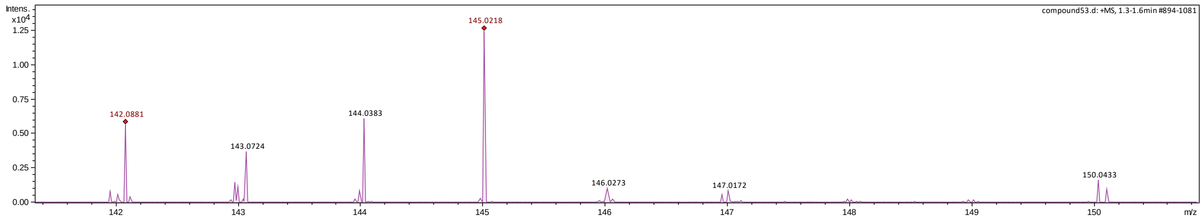

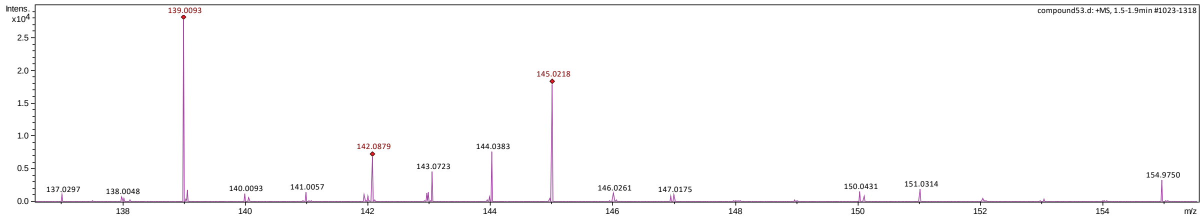

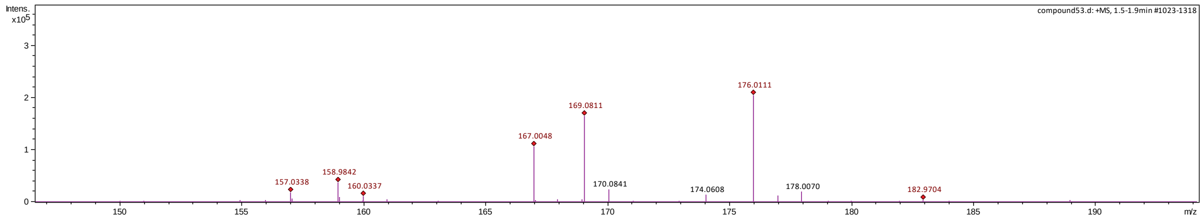

4. Green Metrics

To measure the greenness of our reaction, we evaluate several basic green metrics such as atom economy, carbon economy and Environmental-factor.

Atom economy: The Atom Economy was developed by Barry Trost as a framework for organic chemists to explore "greener" chemistry. The atom economy number shows how much of the reactants are left in the final product [12].

Atom economy = Molecular mass of the desired product/ Molecular masses of the reactants X 100%

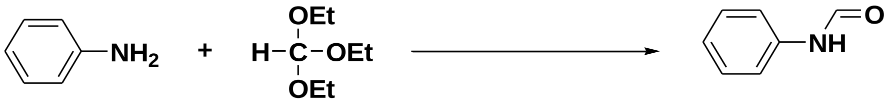

93.06 **148.202** **121.139**

Molecular mass of the product = 121.139

Molecular mass of the reactants = 93.06 + 148.202

Atom economy = 121.139 / 93.06 + 148.202 × 100%

Atom economy = **50.2%**

Carbon Economy: It is how much carbon ends up in the useful product compared to how much carbon was used to create the product.

Carbon economy = number of carbon atoms in desire product/number of carbon atoms in reactant × 100%

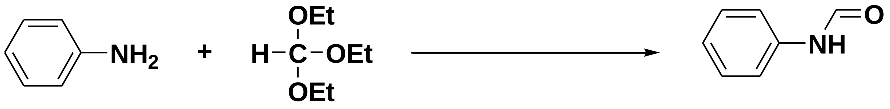

6 **7** **7**

Number of carbon atoms in the product = 7

Number of carbon atoms in the reactants 6 + 7 = 13

Carbon Economy = 7 / 13 × 100%

Carbon Economy = **53.8%**

Environmental factor (E-factor) Calculations:

The E-factor of a process is the ratio of the mass of waste per mass of product:

While calculating the E-factors, we did not consider the amount of silica gel used for column chromatography as it is generally not reported [13].

Calculation of E-factor values for 6 mmol *N*-phenylformamide synthesis by using triethylorthoformate:

Amount of total amount of reactants:

Mass *N*-phenylformamide = 0.548 g

Mass (Triethylorthoformate) = 1.56 g

Mass (Ethyl acetate assuming 90% recovery): 10 mL × (0.92 g/mL) × 10% = 0.92 g

Mass (aq. Na_2SO_4, (calculated by weight) (100 mL)) = 0.7 g

Purification by column chromatography (1:3 ether/EtOAc) for 0.5 g of N-phenylformamide assuming 50 ml of solvent was used and 90% recovery):

Mass (Na_2SO_4 used for drying (assuming 1/8 tea spoon)) = 0.10 g

(Silica have been excluded from this calculation)

Total mass of reactants waste = 0.5 + 1.56 + 0.92 + 0.7 + 0.5 + 0.10 = 3.232 g

Amount of product = 0.52 g

E-Factor = Amount of waste/Amount of product = 3.232/0.52 g = 6.20

E-factor = 6.20

References

[1] Rasheed, S.; Rao, D.N.; Reddy, A.S.; Shankar, R.; Das, P.; Sulphuric acid immobilized on silica gel (H2SO4-SiO2) as an eco-friendly catalyst for transamidation. *RSC Adv.* **2015**, 5, 14, 10567–10574. https://doi.org/10.1039/c4ra16571c.

[2] Khatri C.K.; Chaturbhuj, G.U.; Sulfated polyborate-catalyzed N-formylation of amines: a rapid, green and efficient protocol. *J. Iran. Chem. Soc.* **2017**, 14, 2513–2519. https://doi.org/10.1007/s13738-017-1186-x.

[3] Baltimore, D.; Darnell, J.; Task History Initiating Search, pp. 1–2, 2021.

[4] Ma'mani, L.; Sheykhan, M.; Heydari, A.; Faraji, M.; Yamini, Y.; Sulfonic acid supported on hydroxyapatite-encapsulated-γ-Fe2O3 nanocrystallites as a magnetically Brønsted acid for N-formylation of amines," *Appl. Catal. A Gen.* **2010,** 377, 1–2, 64–69. doi: 10.1016/j.apcata.2010.01.020.

[5] Bose, A.K.; Ganguly, S.N.; Manhas, M.S.; Guha, A.; Pombo-Villars, A.; Microwave promoted energy-efficient N-formylation with aqueous formic acid. *Tetrahedron Lett.* **2006,** 47, 27, 4605–4607. doi: 10.1016/j.tetlet.2006.04.148.

[6] Tumma, H.; Nagaraju, N.; Reddy, K.V.; A facile method for the N-formylation of primary and secondary amines by liquid phase oxidation of methanol in the presence of hydrogen peroxide over basic copper hydroxyl salts, *J. Mol. Catal. A Chem.* **2009**, 310, 1–2, 121–129. doi: 10.1016/j.molcata.2009.06.007.

[7] Lygin, A.V.; De Meijere, A.; ortho-Lithiophenyl isocyanide: A versatile precursor for 3H-quinazolin-4-ones and 3H-quinazolin-4-thiones. *Org. Lett.* **2009**, 11, 2, 389–392. doi: 10.1021/ol802659m.

[8] Habibi, D.; Sahebekhtiari, H.; Nasrollahzadeh, M.; Taghipour, A. A Very Simple, Highly Efficient and Catalyst-free Procedure for the N-Formylation of Amines Using Triethyl orthoformate in Water Under Ultrasound-irradiation. *Lett. Org. Chem*. **2013**, *10*, 209–212. https://doi.org/10.2174/1570178611310030012.

[9] Hosseini-sarvari, M.; Sharghi, H.; ZnO as a New Catalyst for N-Formylation of Amines under Solvent-Free Conditions ZnO under solvent-free conditions brings about highly and in excellent yields. The N-formylation reaction not only formamides . 7 Furthermore , formamides are very useful rea. *Tetrahedron*, **2006**, 8, 6652–6654.

[10] Pathare, S.P.; Sawant, R.V.; Akamanchi, K.G.; Sulfated tungstate catalyzed highly accelerated N-formylation. *Tetrahedron Lett.* **2012**, 53, 26, 3259–3263. doi: 10.1016/j.tetlet.2012.04.058.

[11] Kim, J.J.; Park, Y.D.; Cho, S.D.; Kim, H.K.; Chung, H.A.; Lee, S.G.; Falck, J.R.; Yoon, Y.J.; Efficient N-arylation of pyridazin-3(2H)-ones. *Tetrahedron Lett.* **2004**, 45, 48, 8781–8784. doi: 10.1016/j.tetlet.2004.10.003.

[12] Trost, B.M. The Atom Economy - A Search for Synthetic Efficiency. *Science* **1991**, 254, 1471–1477.

[13] Patil, P.; Ahmadian-Moghaddam, M.; Dömling, A.; Isocyanide 2.0, *Green Chem.* **2020**, 22, 20, 6902–6911. doi: 10.1039/d0gc02722g.
